# Extraction-free LAMP assays for generic detection of Old World Orthopoxviruses and specific detection of Mpox virus

**DOI:** 10.1038/s41598-023-48391-z

**Published:** 2023-11-30

**Authors:** Zhiru Li, Amit Sinha, Yinhua Zhang, Nathan Tanner, Hui-Ting Cheng, Prem Premsrirut, Clotilde K. S. Carlow

**Affiliations:** 1 Molecular Genetics and Genomics Division, New England Biolabs, Ipswich, MA 01938 USA; 2 Mirimus Inc., Brooklyn, NY 11226 USA

**Keywords:** Microbiology, Molecular biology, Diseases, Infectious diseases, Viral infection, Diagnostic markers

## Abstract

Mpox is a neglected zoonotic disease endemic in West and Central Africa. The Mpox outbreak with more than 90,000 cases worldwide since 2022 generated great concern about future outbreaks and highlighted the need for a simple and rapid diagnostic test. The *Mpox virus*, *MPV*, is a member of the *Orthopoxvirus* (*OPV*) genus that also contains other pathogenic viruses including variola virus, vaccinia virus, camelpox virus, and cowpox virus. Phylogenomic analysis of 200 OPV genomes identified 10 distinct phylogroups with the New World OPVs placed on a very long branch distant from the Old World OPVs. Isolates derived from infected humans were found to be distributed across multiple phylogroups interspersed with isolates from animal sources, indicating the zoonotic potential of these viruses. In this study, we developed a simple and sensitive colorimetric LAMP assay for generic detection of Old World OPVs. We also developed an MPV-specific probe that differentiates MPV from other OPVs in the N1R LAMP assay. In addition, we described an extraction-free protocol for use directly with swab eluates in LAMP assays, thereby eliminating the time and resources needed to extract DNA from the sample. Our direct LAMP assays are well-suited for low-resource settings and provide a valuable tool for rapid and scalable diagnosis and surveillance of OPVs and MPV.

## Introduction

Mpox (previously known as Monkeypox) was first described in 1958 in laboratory monkeys shipped from Singapore to Denmark^1^. The first Mpox case in humans was reported in 1970 in the Democratic Republic of the Congo^2^ followed by sporadic outbreaks mainly in West and Central Africa^3^. Even though about 400 confirmed cases of Mpox from these areas were reported in the last two decades, the suspected number of cases has been predicted to be much higher at around 28,000^4^. While Mpox remains a neglected tropical disease, the 2003 outbreak in the United States generated international attention and the global outbreak in 2022 in more than 100 countries led to the declaration of a Public Health Emergency of International Concern by the World Health Organization^5^, with 91,328 cases as of October 26, 2023. Mpox is caused by the *Mpox virus* (*MPV*), which is an enveloped double-stranded DNA virus with an AT-rich (67% on average) genome of about 200 kb encoding 190 open-reading frames^6^. It is a member of the *Orthopoxvirus* (*OPV*) genus that also contains several viruses of great medical relevance, including *Variola virus* (*VARV*), the causative agent of smallpox; *Vaccinia virus* (*VACV*), the virus used in the smallpox vaccine; *Cowpox virus* (*CPXV*), *Camelpox virus* (*CMPV*), and a few other species that could infect humans^7^. Currently, MPV is classified into two major clades, each with distinct geographical, clinical, genomic, and epidemiological differences^8^. Viruses from Clade I (former Congo Basin clade) cause more clinically severe disease in humans, with higher mortality rates and transmissibility, while those from Clade II (former West African clade) have a milder clinical presentation with lower mortality rates and transmissibility. Clade II is further divided into Clade IIa and Clade IIb. The isolates from the most recent 2022 outbreak are placed in Clade IIb and closely related to the virus in the same clade responsible for the 2017–2019 outbreaks^8–10^.

Human-to-human transmission of MPV can occur by direct contact, respiratory secretions, vertical transmission, or indirect contact through fomites. Direct contact with infectious sores or lesions on mucous membranes was the primary mode of transmission during the 2022 outbreak^11^. Mpox diagnosis is based on suspected epidemiological and clinical symptoms and confirmed by nucleic acid amplification testing, mainly real-time PCR. The recommended diagnostic specimens are collected directly from skin lesions or biopsies^12,13^. Viral DNA can also be detected in saliva^14^, semen, blood, or urine samples, though usually less frequently and with a lower viral load^15–17^ but could potentially help early detection of Mpox before the development of skin lesions. At present, detecting viral DNA by quantitative polymerase chain reaction (qPCR) is the recommended laboratory test for Mpox^18^; indeed, a positive PCR result is considered definitive, regardless of associated symptoms. However, the qPCR method requires complex and expensive equipment. In rural areas or low-resource settings without access to high-precision PCR instruments, a faster and simpler method for Mpox testing is needed as a viable alternative. Loop-mediated isothermal amplification (LAMP) is a nucleic acid amplification method that uses a Bst DNA polymerase with strand displacement activity. The assay is conducted under isothermal conditions ranging from 60 to 66 °C and is fast and simple to use, making it ideal for the diagnosis and surveillance of neglected tropical diseases^19^. LAMP diagnostic assays have also been successfully used as a point-of-care diagnostic tool during the SARS-CoV-2 pandemic^20–22^.

In this study, we performed a comprehensive phylogenomic analysis of OPV genomes and developed a colorimetric LAMP assay that can detect all Old World OPVs, as well as a fluorescent probe-based LAMP assay for specific detection of MPV. In addition, we described a protocol to detect viral DNA directly from the swab samples without the need for a DNA extraction step. Given the constant threat and continued transmission of MPV in different parts of the world^23^, accessibility to testing is critical for worldwide control efforts. Our simple and quick colorimetric LAMP assay can improve the testing capability, especially in remote and low-resource areas.

## Results

### Phylogenomic analysis of Orthopoxvirus genomes

**Figure 1:**
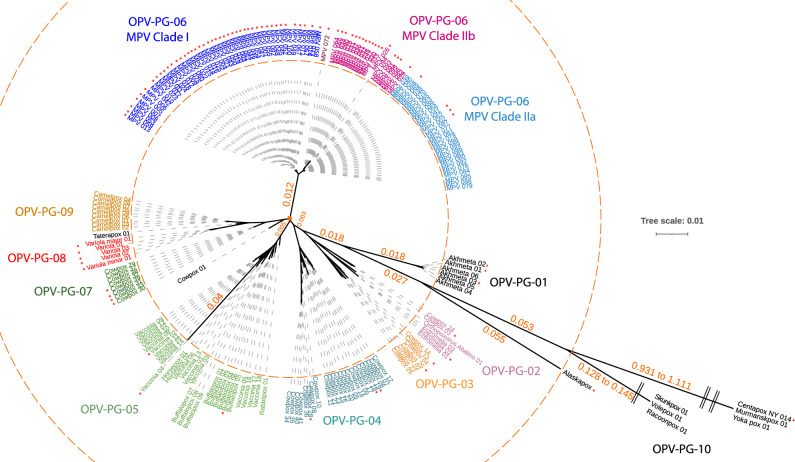
Phylogenomic analysis of *Orthopoxvirus* species. An unrooted phylogenomic tree of 200 Orthopoxviruses, including 100 genomes from various MPV was constructed based on 38 single copy orthologs, with a combined supermatrix sequence length of 17,205 nucleotides. The *Centapoxvirus* species were used as an outgroup. The nucleotide substitution model GTR + F + I + G4 that best fit the sequences was used. The phylogroups are color coded and indicated by corresponding labels. The virus isolates derived from infected humans are marked with red asterisks. The branches are drawn proportional to the computed branch length, except when marked by pair of parallel bars across the branch. The inner dotted circle marks the maximum branch lengths observed within the Old World OPVs. The outer dotted circle marks the Alaskapox virus branch length. Branch length values are displayed along the paths to some long branches.

A phylogenomic analysis of 200 Orthopoxvirus genomes, identified 10 distinct clades within this genus, which are here named as phylogroups 1 to 10 and are denoted as OPV-PG-01 to OPV-PG-10 (Fig. 1) based on their nesting patterns (Supplementary Fig. S1), where the 100 MPV isolates included in this analysis are placed in OPV-PG-06. Two distinct clades were observed within OPV-PG-06, which correspond to the recently described MPV Clade I and Clade II^4^, with isolates from the 2022 global outbreak placed in Clade IIb (Fig. 1). The phylogroup most closely related to MPV is OPV-PG-05 which contains all vaccinia virus isolates, as well rabbitpox, buffalopox and horsepox viruses (Supplementary Fig. S1). The next closest virus was a so-called cowpox virus which was isolated from a cat^24^ and does not cluster within any of the phylogroups. Other “cowpox” viruses have been isolated from a diverse set of host animals other than cows, such as humans, cats, alpaca, and rodents (S1 Table). These viruses do not form a monophyletic clade but rather are seen as 4 distinct clades PG-02, PG-03, PG-04, and PG-07, each containing a mixture of both human and non-human derived isolates (Fig. 1). The New World OPVs^25^ namely the *Raccoonpox virus* (*RAPV*), *Volepox virus* (*VPXV*), and *Skunkpox virus* (*SKPV*), were placed in OPV-PG-10, the most distant members observed within the Orthopoxvirus genus (Fig. 1). The Alaskapox virus (AKPV) did not cluster within any phylogroup, with a branch length between those of the Old World OPVs and New World OPVs, consistent with previous analysis^26^. The branch length of AKPV (outer dotted circle, Fig. 1) was found to be more than twice the maximum branch length observed within all Old World OPVs (inner dotted circle, Fig. 1). The New World OPVs were placed even farther out. These extreme branch lengths in the phylogenomic tree suggest that the taxonomic classification of AKPV and New World OPVs within the Orthopoxvirus genus needs to be reconsidered.

Analysis of pan-phylogroup proteomes, formed by combining protein sequences from each isolate within a phylogroup, revealed common and distinct orthogroups present in various phylogroups (Supplementary Fig. S2). A total of 160 orthogroups were present across the Orthopoxvirus genus as well as in the Centapox outgroup, and 12 orthogroups were present in the Orthopoxvirus genus but absent from the Centapox genus. This analysis also showed 39 orthogroups that were present only in the pan-MPV proteome, which potentially could be used as MPV specific biomarker. However, none of these orthogroups were present across all MPV isolates, precluding their use as MPV specific biomarkers. Analysis of the hosts of Orthopoxviruses from which they were isolated (S1 Table) revealed that viruses isolated from human were distributed throughout the tree (red asterisk in Fig. 1) and interspersed with closely related isolates from various animal hosts. This distribution pattern indicates the dramatic zoonotic potential and disease risk of these viruses, highlighting the urgency for a pan-Orthopoxvirus detection assay.

### Colorimetric LAMP assay development and sensitivity

A total of 14 sets of primers (S2 Table) targeting 8 different genes were tested in the colorimetric LAMP assays with fluorescent dye. The time to reach the signal threshold (Tt) determined from the real-time fluorescence signal indicated the speed of the LAMP reaction. Since shorter Tt could correlate with faster primer sets and increased sensitivity, the Tt from each primer set was recorded. Among the 14 sets of primers evaluated, 10 showed consistent amplification signals using MPV DNA as the template, however, primer sets (Table 1) targeting the A4L (set 2) gene and the N1R (set 3) gene were chosen as they showed the best performance with the earliest signal detection and without any background signal. The optimal LAMP reaction temperature was found to be 65 °C with 40 mM GuHCl included as an additive to speed up the reaction^27^.

**Table 1:** LAMP primer sequences targeting A4L and N1R.

Primer	Sequence 5′–3′
A4L_F3	CAAGGATATTTATTCTATGGCATTC
A4L_B3	CCAAACATATTCTTATTCTGACGT
A4L_FIP	TCCAGAACATCTTCCATAGCCTAGATGGCAATAGTGGAAGAGTG
A4L_BIP	TACACACATTGATCCATTGGGAACCATCATTGCTCCATTAACGATA
A4L_LF	TGTTAGGAGGAGCGAACAC
A4L_LB	TAATGTGATGGGTAGTGCTGT
N1R_F3	GAATTGATGCAATGGAGCTA
N1R_B3	GCAGCATAAGTAGTATGTCG
N1R_FIP	TCTCCACGCAATTGTCGATATTGGTAGCGAGTTGAAGGAGTT
N1R_BIP	ACTCCATGAAAACCGCCAAAGAAGACTCTTCCAGTGACA
N1R_LF	CCACGGAAGTGAATTCGAG
N1R_LB	TGGACTTTGTACTCAATCAGCT

**Figure 2:**
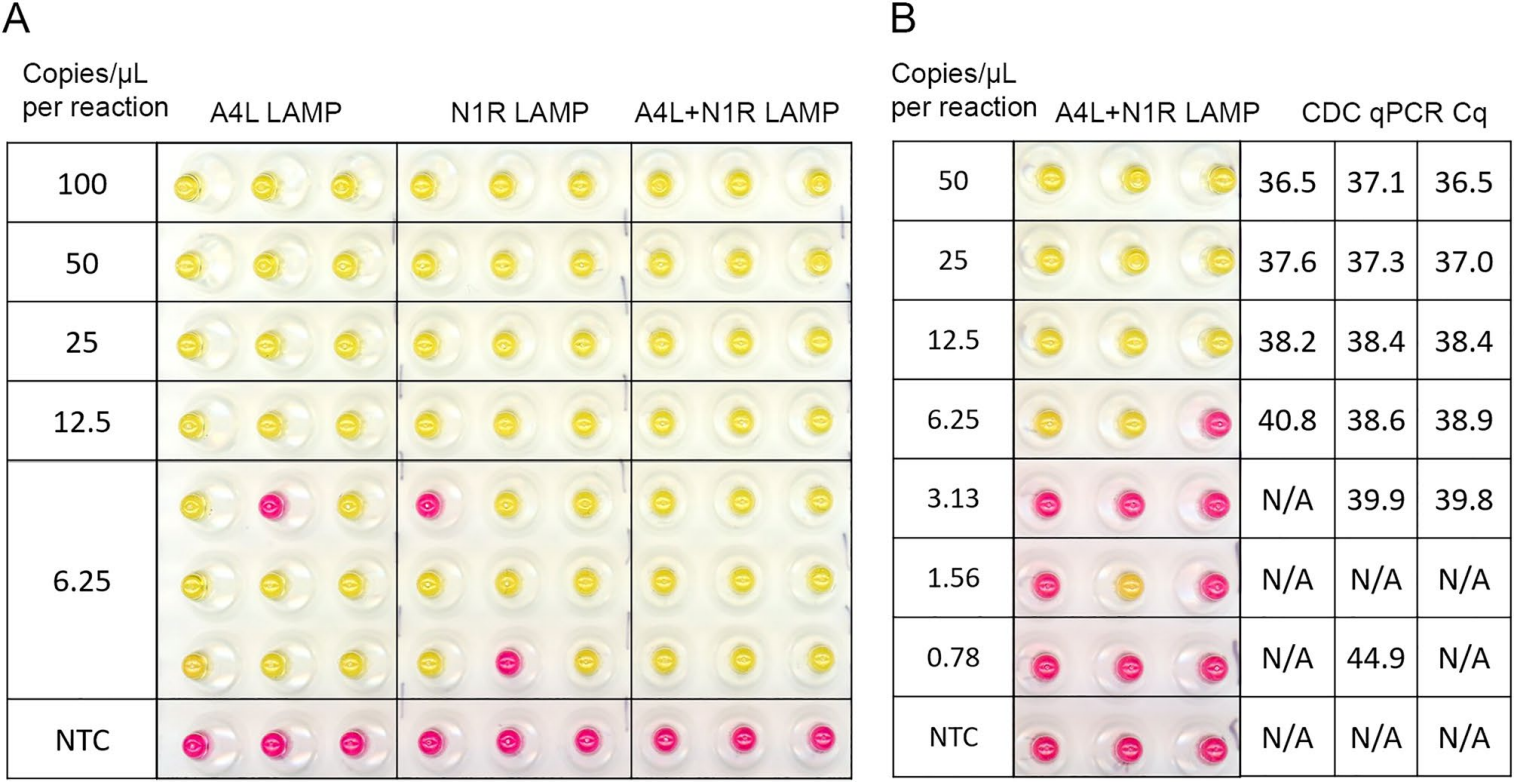
A4L and N1R LAMP assays can detect MPV DNA. (**A**) MPV synthetic partial genome DNA from Twist was diluted from 500 to 31.25 copies/µL in 0.1X TE buffer containing 1 ng/µL human DNA. 4 µL of DNA was tested in 20 µL LAMP reactions with either A4L primer set or N1R primer set or both. The copy number in each µL LAMP reaction was indicated. Scanned images of the post-amplification plate showing the colorimetric (pink = negative, yellow = positive) readouts are shown. (**B**) MPV genomic DNA from BEI was diluted from 250 to 3.125 copies/µL and 4 µL of DNA was tested in colorimetric LAMP or CDC qPCR assays. The copy number in each µL LAMP reaction was indicated. The scanned image of the post-LAMP amplification plate and Cq number of CDC qPCR are shown. No amplification is denoted N/A. All reactions were performed in triplicates.

The sensitivity of the colorimetric A4L and N1R LAMP assays was first evaluated using a serial dilution of synthetic partial genomic MPV DNA. As shown in Fig. 2A, synthetic DNA can be detected at 12.5 copies/µL in all triplicate reactions with either A4L or N1R LAMP primers in 20 µL LAMP reactions. At 6.25 copies/µL, 8 out of 9 A4L LAMP reactions and 7 out of 9 N1R LAMP reactions showed a positive color change. As expected, the LAMP reaction containing both sets of LAMP primers improved the sensitivity and enabled the detection of all 9 reactions at 6.25 copies/µL.

Genomic DNA from MPV_USA2003 strain was also tested in the A4L + N1R colorimetric LAMP assay. The CDC Non-variola Orthopoxvirus Generic Real-Time PCR Test was performed as a control for the quality of the DNA. As shown in Fig. 2B, all triplicate samples tested positive in the LAMP assay down to 12.5 copies/µL. At 6.25 copies/µL, 2 out of 3 reactions showed a positive color change in the LAMP assay, consistent with an average late Cq value of 39.4 observed in the CDC qPCR assay. While qPCR assay can detect 2/3 reactions at even lower concentrations, no LAMP reactions showed positive color change, indicating the LAMP assay is slightly less sensitive than the current gold standard qPCR assay.

To evaluate the limit of detection, DNA templates that match the MPV A4L and N1R targets were synthesized as gBlock gene fragments and tested (Supplementary Fig. S3). For both A4L and N1R LAMP, at 10 copies/µL of gBlock DNA, all 21 replicate LAMP reactions tested positive, while the 3 negative controls tested negative. At 5 copies/µL, of the 24 LAMP replicate reactions, 20 (83.3%) A4L LAMP replicates and 18 (75%) N1R LAMP replicates showed a positive color change from pink to yellow.

### Specificity of A4L LAMP assay

**Figure 3:**
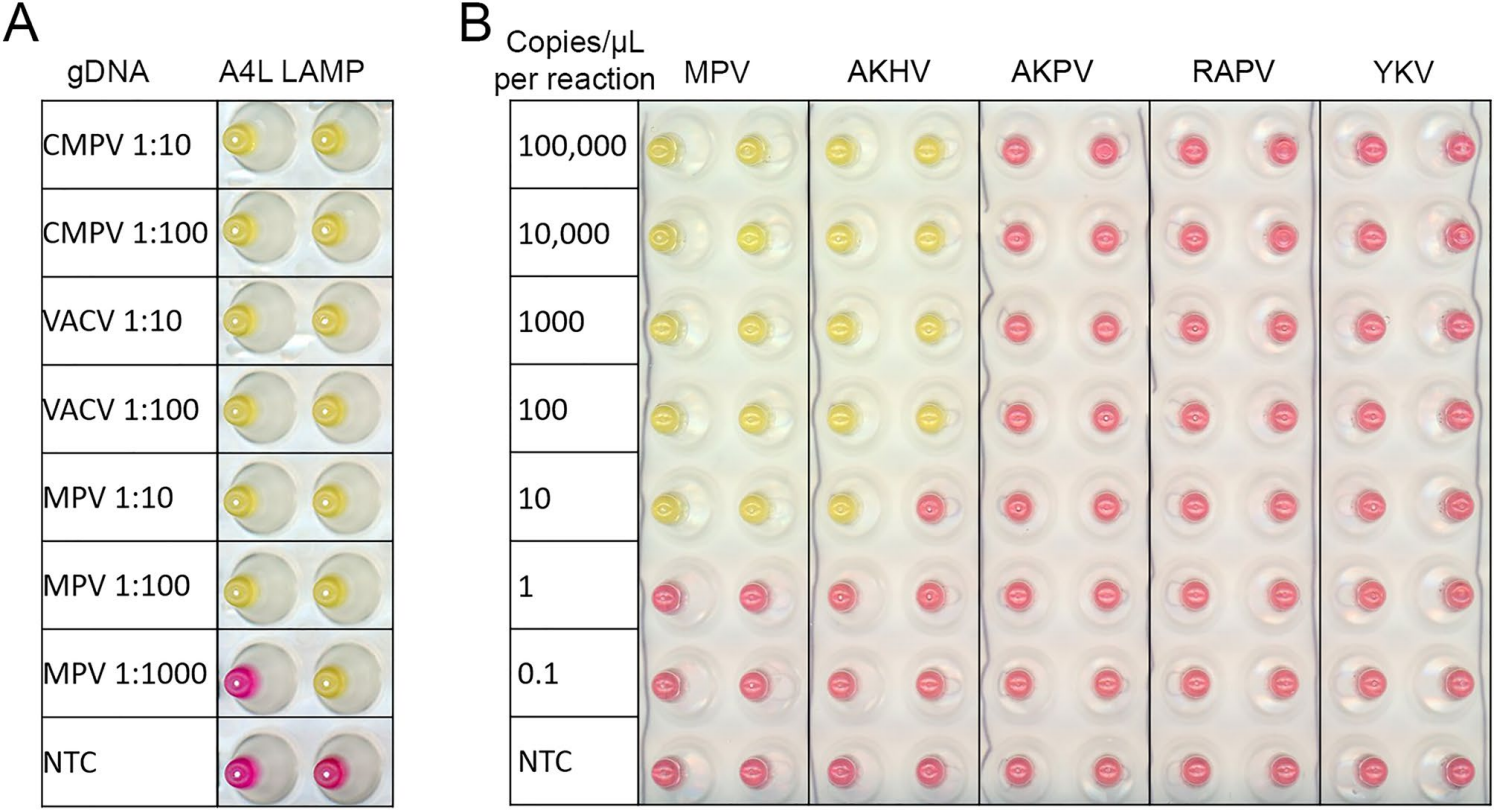
A4L assays on genomic DNA and A4L gBlock DNA from various Orthopoxviruses. (**A**) Genomic DNA from Camelpox virus (CMPV), Vaccinia virus (VACV), and *Mpox virus* (MPV) diluted 10 or 100-fold were tested in A4L colorimetric LAMP assay. (**B**) gBlocks that match the A4L LAMP regions from MPV, Akhmeta virus (AKHV), Alaskapox virus (AKPV), Raccoonpox virus (RAPV), and Yokapox virus (YKV) were serial diluted and tested. The colorimetric (pink = negative, yellow = positive) readouts were obtained, and the scanned image of the post-amplification plates are shown. No amplification is denoted N/A. Experiments were performed with two replicates.

The A4L gene is located in the more conserved central region of the viral genome and encodes the precursor of the essential major virion core protein p4b within the Orthopoxvirus. The A4L amplicon region and primer binding sites were found to be highly conserved within all MPV isolates available in GISAID EpiPox™ database (Supplementary Fig. S4). Among 7370 MPV isolates, 99.8% (7352) contain identical A4L amplicon sequence. A single nucleotide difference was observed at various positions in 17 isolates. The A4L gene sequence was also found to be highly conserved in Old World OPVs with most of the orthologs showing 1 to 3 nucleotide differences in the LAMP targeting region (Supplementary Fig. S5). The most common A to C substitution does not impact the performance of the LAMP reaction since it is located between the LB and B2 primer regions. The effect of nucleotide differences located within the LAMP primer binding sites such as single nucleotide differences located in either the F2 or F1c primer region (Supplementary Fig. S5) were tested with genomic DNA from CMPV or VACV, which harbor these substitutions. As shown in Fig. 3A, these differences did not impact the ability of the A4L LAMP to detect the CMPV and VACV genomic DNAs, as successful amplifications were observed with a positive color change from pink to yellow. Even A4L gBlock gene fragment from Akhmeta virus (AKHV) that contains 4 nucleotide mismatches can still be detected (Fig. 3B) consistent with the previous report on LAMP tolerance of sequence variation^28^. Among the more distantly related AKPV and New World OPVs (Fig. 1) including VPXV, SKPV, and RAPV, the number of mismatched nucleotides is ranging from 12 to 33, indicating that these viruses probably could not be detected by the A4L LAMP. Consistently, when A4L gBlock gene fragments (Supplementary Note) from AKPV and RAPV were tested, no amplification signal could be detected (Fig. 3B). Similarly, the A4L gBlock gene fragment of the Yokapox virus (Centapoxvirus genus within the Poxviridae family) also showed no amplification signal with A4L LAMP primers. Taken together, these results indicate that the A4L LAMP can be used to detect all the Old World OPVs including MPV, but not AKPV, or the New World OPVs, or other genus within the Poxviridae family.

### Probe-based N1R LAMP is specific for MPV

**Figure 4:**
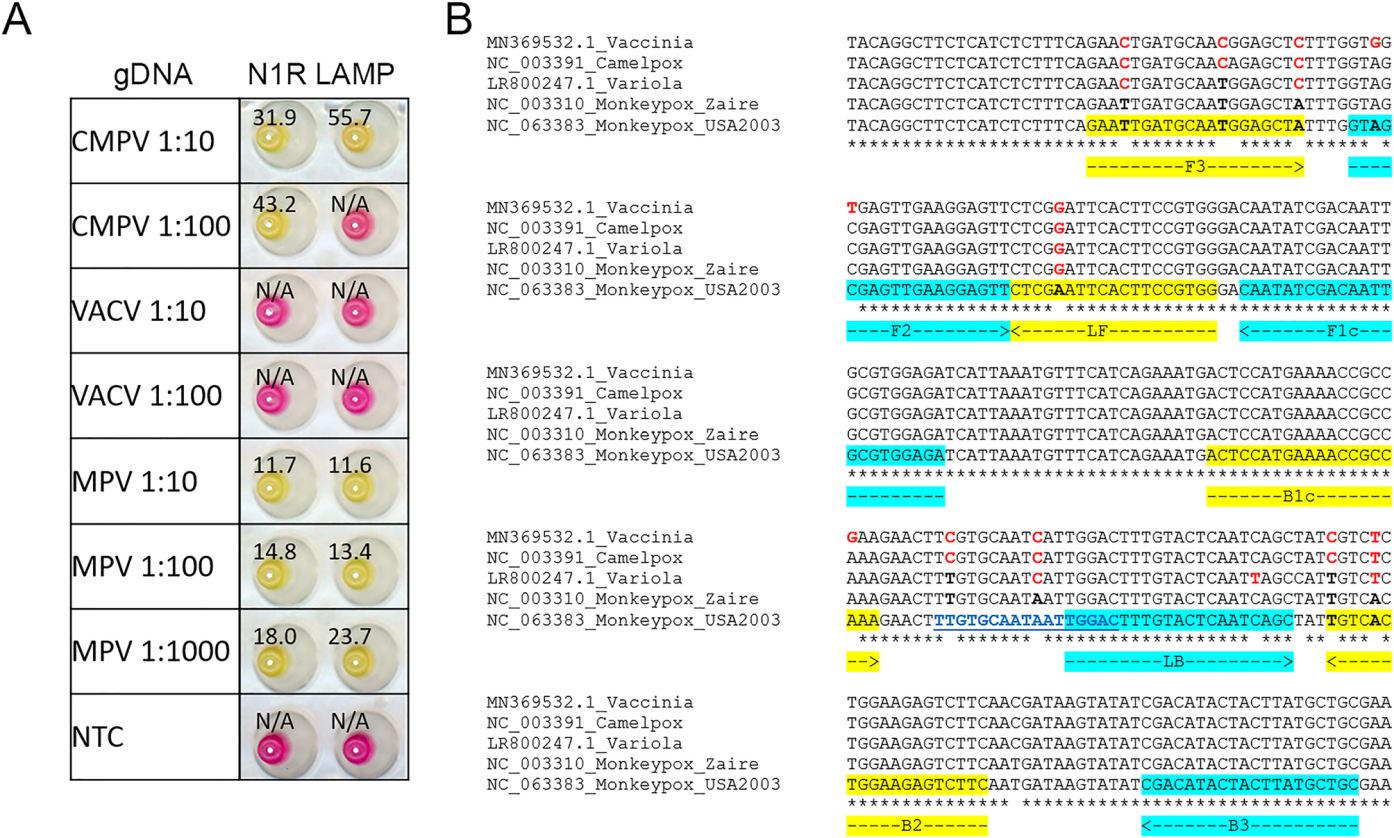
N1R LAMP and target sequence alignment from various Orthopoxvirus. (**A**) Genomic DNA from Camelpox virus (CMPV), Vaccinia virus (VACV), and Mpox virus (MPV) diluted 10 or 100-fold were tested in N1R colorimetric LAMP assay. The colorimetric (pink = negative, yellow = positive) readouts were obtained, and the scanned image of the post-amplification plates with overlaid Tt values defined as the time (min) to reach threshold fluorescence values during the amplification are shown. (**B**) N1R sequences were downloaded from NCBI and aligned with ClustalW. Nucleotides different from the MPV sequence are indicated in bold red. Primer regions are highlighted in yellow or cyan. Nucleotide sequences used for the detection probe are shown in bold blue and underlined.

The N1R gene is located towards the termini of the genome and encodes a Poxvirus Bcl-1-like protein, predicted to be involved in evading the host’s innate immune response. It is specific for OPVs since no N1R homologs could be found outside of this genus based on the NCBI Blast search. Among different MPV isolates, the N1R region is highly conserved, with 99% N1R sequences available in GISAID EpiPox™ database having identical sequence in the N1R amplicon region (Supplementary Fig. S6). In other OPVs, the gene is more variable (Fig. 4 and Supplementary Fig. S7). For example, the CMPV N1R gene contains multiple nucleotide mismatches in the primer binding regions, including 3 in F3, 2 in B2, and 1 in LF. The effect of these differences was tested using genomic DNA from CMPV and VACV. While 100-fold diluted MPV DNA can be detected with a Tt value of 14 min, only 1 out of 2 reactions showed a positive result for CMPV DNA at that dilution, and with a significant delay of the amplification as indicated by a higher Tt value of 43 min (Fig. 4) reflecting decreased amplification efficiency from mismatched nucleotides. No positive amplification was observed for VACV DNA because the corresponding sequence of this genomic DNA (NC_006998) lacks the N1R gene, different from other VACV isolates which contain the N1R gene with a similar number of mutations as CMPV (Supplementary Fig. S7). These results indicate that the N1R assay is better suited to detect MPV compared with CMPV, but it is not specific for MPV.

**Figure 5:**
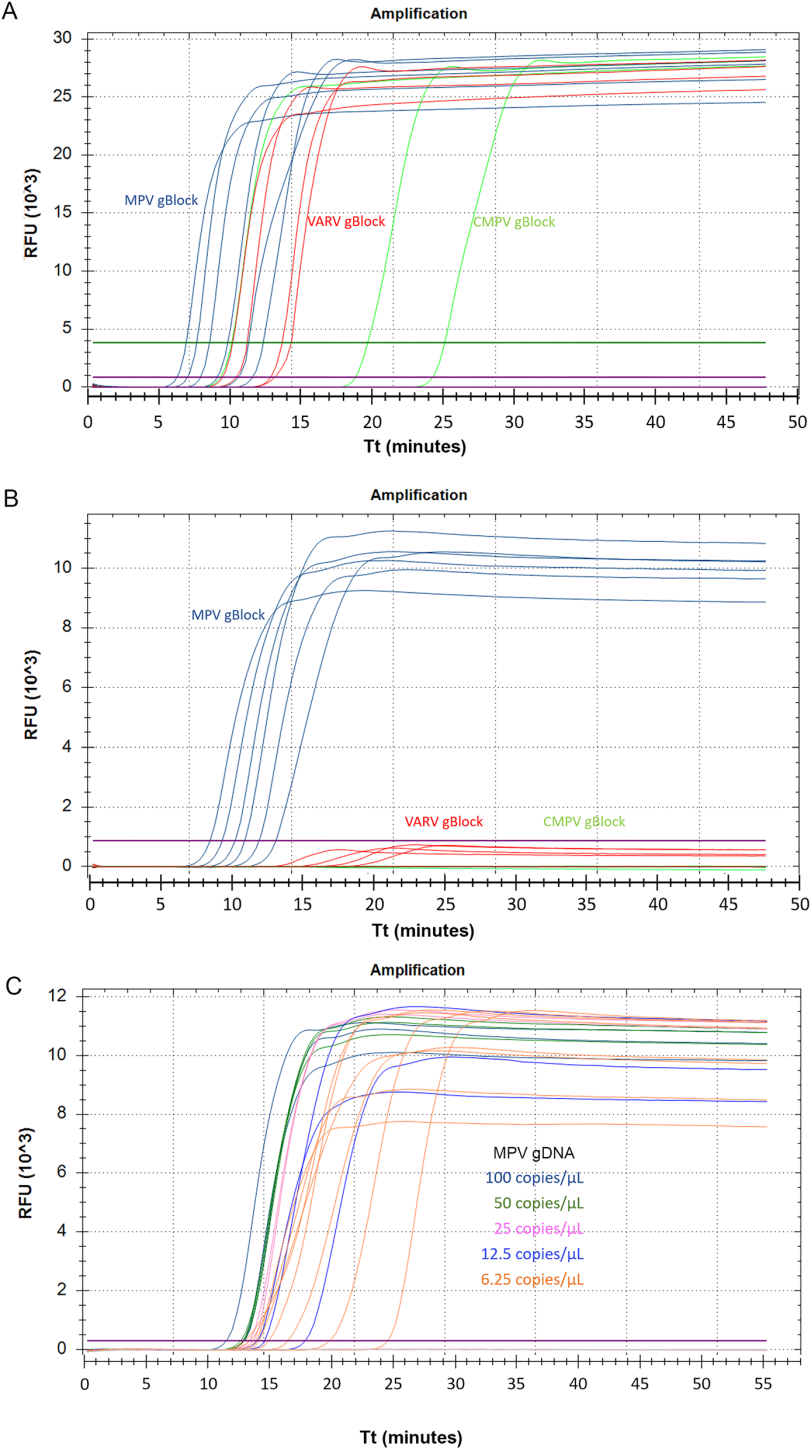
Specificity and sensitivity of probe-based LAMP assay. A tenfold dilution series of N1R gBlocks from MPV (blue), CMPV (green), and the VARV (red) ranging from 5,000,000 to 5 copies/µL and 2 µL were tested in duplicate in fluorescent LAMP reactions containing either a DNA-binding fluorescent dye (**A**) or Cy5 labelled MPV specific probe (**B**). (**C**) MPV genomic DNA was serial diluted over a range of 100 to 6.25 copies/µL and tested in 20 µL of N1R LAMP reaction contain MPV specific probe. All reactions were performed in a Bio-Rad Opus qPCR machine. Amplification signal was acquired every 15 s (total incubation time was less than 1 h). The fluorescent amplification curves over time are shown. The Tt values measuring the incubation time in minutes to reach the fluorescent threshold were calculated using 10% fluorescent level as the baseline.

To improve the N1R LAMP specificity toward MPV, a detection probe labelled with a fluorophore and quencher was designed. Fluorescent detection probes with locked nucleic acid (LNA) bases have been successfully used to distinguish SARS-CoV-2 variants in LAMP assays^29^. The probe was designed to take advantage of the 2 nucleotide differences in the N1R region between B1c and LB primers (Fig. 4B and Supplementary Fig. S7). In N1R LAMP reactions containing a DNA-binding fluorescent dye, amplification signals were observed for both MPV and CMPV templates (Supplementary Fig. S8A). In contrast, in reactions using the MPV-specific N1R probe, amplification signals were only observed for MPV DNA, but not for CMPV or VACV DNA (Supplementary Fig. S8B), indicating the probe can distinguish amplicons with a 2-nucleotide difference in the probe binding region. While most of the N1R homologs were found to contain several nucleotide differences in the probe binding region, an exception was found in some variants of the VARV, for example, in the genome of LR800247.1 (Fig. 4) there is only 1 nucleotide mismatch in the probe region. To test if the probe can differentiate a single nucleotide difference for this VARV isolate, a synthetic N1R gBlock gene fragment mimicking the variola N1R gene was designed by mutating 6 nucleotide positions within the primer binding sites in the MPV N1R LAMP targeted region (Supplementary Note). A range of 1 to 10,000,000 copies of gBlock DNA from MPV, VARV, and CMPV were tested in fluorescent N1R LAMP reactions. When a DNA-binding fluorescent dye was used for amplification detection, 6 dilutions of MPV gBlock DNA down to 10 copies, 4 dilutions of VARV gBlock DNA down to 1000 copies, and 3 dilutions of CMPV gBlock DNA down to 10,000 copies could be detected (Fig. 5A). In contrast, when the MPV-specific probe was used, no signals were detected for CMPV N1R gBlock DNA, even at the highest copy number of 10,000,000. For the Variola mimic gBlock DNA, the addition of the probe resulted in strong suppression of the amplification signal (Fig. 5B). When compared to the MPXV positive control, the fluorescent signal was less than the baseline threshold (1/10 of the maximum fluorescence signal of positive control). In addition, the Tt from the lowest dilution of MPV gBlock DNA was still smaller than Tt of the highest dilution of VARV gBlock DNA. In practice, a positive control for MPV amplification should always be included to set the baseline threshold. These experiments demonstrated that the probe-based LAMP assay can be used for specific detection of MPV.

To determine the sensitivity of probe based N1R LAMP assay, a serial dilution of MPV genomic DNA was tested. In each 20 µL of reaction, a reliable detection down to 12.5 copies/µL of template was observed around 20 min, at 6.25 copies/µL, 8 of 9 reactions scored positive (Fig. 5C). Overall, this probe-based LAMP assay is both specific and sensitive.

### An extraction-free method for rapid detection of MPV in clinical samples

**Figure 6:**
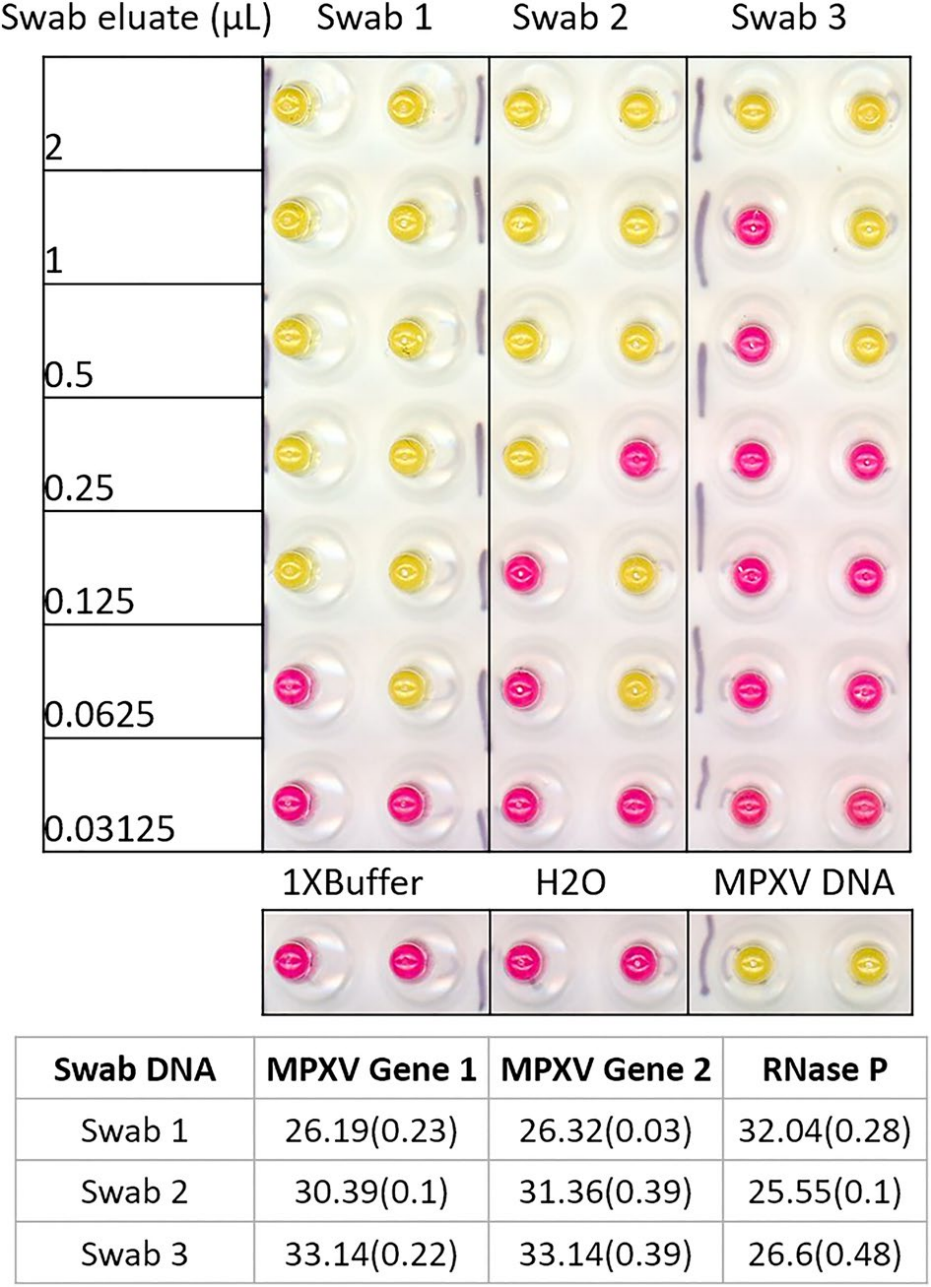
Direct virus detection of clinical swab eluate. Elute from the swab was mixed with an equal volume of 2× SLB buffer and heated at 95 °C for 5 min, then cooled to 4 °C. For each LAMP reaction, 4 µL of swab or serially diluted swab in 1× SLB was used. Water or 1×SLB buffer was used as the negative control, and MPV Twist DNA was used as a positive control. The experiment was done in duplicate.

Most molecular diagnostic protocols for MPV detection involve DNA extraction from lesion material, such as lesion fluid on a dry swab. However, DNA extraction is a time-consuming and expensive process. An extraction-free colorimetric LAMP method has been successfully applied for SARS-CoV-2 detection in clinical samples^20^. This approach was evaluated and found to work well for MPV detection directly from swab eluate when compared with the standard CDC qPCR method using purified DNA. After the eluates of lesion swabs from 3 patients were mixed with sample prep buffer and heated at 95 °C for 5 min, all three treated crude samples generated positive color change in the colorimetric LAMP assay (Fig. 6). Consistent with the viral titer demonstrated by the qPCR assay, as little as 0.0625 µL of the eluate from the high viral containing Swab 1 is sufficient to generate a positive signal in the colorimetric LAMP assay, while 2 µL eluate from the low viral containing Swab 3 was needed. Therefore, the extraction-free LAMP method displayed comparable performance to qPCR on purified DNA.

## Discussion

Members of the genus *Orthopoxvirus* are highly prevalent, zoonotic viruses that infect many mammals, and are particularly relevant to human health. While smallpox has been eradicated, the recent global outbreak in 2022 of Mpox caused by MPV underscores the constant threat from these viruses and highlights the need for diagnostic tools for continued surveillance. The design and development of such assays require an understanding of the relationships between various OPVs. Here we have utilized ~ 200 genome sequences available in public databases to construct a comprehensive phylogenomic tree covering all known OPVs. This analysis showed the presence of 10 distinct clades, denoted as phylogroups OPV-PG-01 to OPV-PG-10. No clear correspondence between the viruses within a phylogroup and their host species was found. For example, the so-called cowpox virus has been isolated from diverse animals, and these isolates were placed in multiple phylogroups, consistent with previous reports^24^. The phylogenomic analysis also demonstrated that the viruses SKPV, RAPV, and VPXV which are together considered as New World OPVs were very distant from all the Old World OPVs. AKPV was placed in between New World and Old World OPVs but was only distantly related to either group. This new information indicates the current taxonomic classification of AKPV, SKPV, RAPV and VPXV within OPVs needs to be re-evaluated. Further, as more novel and diverse viruses continue to be discovered^25,26^, the use of their genetic sequence and phylogenetic analysis should be more extensively adopted for informative nomenclature and accurate classification rather than naming them after the first host species or broad phenotypic properties^24^. Interestingly, various OPVs that have been isolated from human subjects do not cluster together in a particular phylogroup but are distributed across the tree, illustrating that viruses from any of the phylogroups could be a threat to human health and agents of global outbreaks^7,30^. A pan-OPV assay would be ideal for monitoring animal reservoirs as well as new outbreaks in humans, while a species-specific viral test would be required for accurate diagnosis and treatment during a particular outbreak. However, the large genetic distance of AKPV and New World OPVs highlights the fact that within the current classification scheme, the design of a pan-OPV assay would be challenging due to high divergence in nucleotide sequences across the tree. This study focused on generic detection of Old World OPVs which are more closely related to each other and contain the majority of human OPV pathogens currently known.

MPV has been responsible for multiple outbreaks in recent decades. There are numerous ways to detect MPV, including the detection of viral particles, virus-specific antigens, and many PCR-based methods^31^. The goals of the present study were to develop an isothermal, simple, rapid, and robust workflow for both OPVs and MPV specific diagnosis. In resource-limited settings, where access to thermocyclers and a stable electricity supply is challenging, LAMP-based assays are attractive. Several isothermal amplification-based assays, including LAMP and recombinase polymerase amplification, have been developed for diagnosing MPV infection^32,33^ and used turbidimeters or gel electrophoresis instruments for readout, whereas our colorimetric LAMP test provides a simple visual readout.

One of the CDC’s clade-specific assays targets the tumor necrosis factor (TNF) receptor gene (G2R)^34,35^ which is located within the terminal inverted-repeat region and contains many single nucleotide polymorphisms and insertions/deletions specific for Clade II. However, in the recent outbreak, new deletions in the G2R target have negatively impacted the performance of the test resulting in dropouts. This led to a CDC advisory on September 2nd, 2022, urging caution in the interpretation of negative results returned from the G2R assay if clinical suspicion for Mpox was high^36^. The rapid accumulation of mutations in the 2022 MPV isolates^37^ necessitates the selection of more conserved targets. In our colorimetric LAMP assay, the high conservation of the A4L target in Old World OPVs and the high conservation of the N1R target within all MPV isolates, combined with the tolerance of mismatches in LAMP reactions^28^, are expected to result in fewer false negative results. The A4L LAMP region is less conserved in the AKPV and SKPV, RAPV, and VPXV with 12 to 30 mismatches in the primer binding region. These viruses are placed on very long branches in the phylogenomic tree indicating they are only distantly related to Old World OPVs and cannot be detected with A4L LAMP.

To achieve specific MPV detection, a hybridization detection probe was designed and included in the N1R LAMP assay. The probe-based detection method requires no modification to the LAMP primers and can be used for the detection of single-nucleotide polymorphisms and small sequence changes^29^. The LAMP probe in our assay contains a 5′ end fluorophore and a 3’ end dark quencher. The probe only generates a fluorescent signal upon binding to N1R amplicons due to the increased structural rigidity of dsDNA and subsequent separation of the fluorophore and quencher. To enable using a short probe with high Tm, Locked Nucleic Acid (LNA) bases were incorporated into the probe, which is important to achieve specificity. The probe was designed to target the loop region of the LAMP amplicons to provide greater availability for probe annealing and less frequent displacement by the polymerase and showed little inhibition of the probe to the LAMP reaction. In our assay, a real-time PCR instrument was used for probe-based fluorescence detection. However, in the field, portable isothermal fluorimeters, endpoint plate readers, simple illumination instruments, or lateral flow can potentially be used for signal detection. In a recently published colorimetric LAMP assay targeting two different genes namely A27L and F3L, high specificity was claimed based on in silico analysis^38^. Using the published primer sets for A27L and F3L and their recommended reaction temperature, we found both CMPV and VACV DNA were detected (Supplementary Fig. S9) indicating that these assays lack specificity to MPV. This result is also consistent with our findings from a comprehensive in silico analysis of this sequence region across all OPV genomes available in NCBI revealing that these regions are highly conserved across OPVs, with only a couple of nucleotide differences in the primer binding regions (Supplementary Figs. S10 and S11).

Most MPV detection assays in use, including the CDC-recommended assays, utilize purified DNA in qPCR reactions. In a rapidly expanding viral outbreak, testing can be limited by the time and cost of nucleic acid extraction steps, as witnessed during the SARS-CoV-2 pandemic. In our study, we used a simple extraction-free method involving a sample lysis buffer and heat treatment to release the DNA for detection directly from swab eluates. The heat treatment also inactivates the virus for quick and safe handling^39^. Detection of MPV in saliva has been demonstrated to have a sensitivity of 88% compared to skin lesions^40^. Importantly, a saliva-based PCR test identified asymptomatic cases^41^ before the appearance of rash or lesion, indicating that using a saliva test could help detect Mpox earlier in the time course of the illness. Earlier identification would likely reduce transmission. We expect our extraction-free colorimetric LAMP method could also be used to detect MPV in saliva as demonstrated previously for LAMP-based SARS-CoV-2 detection in saliva^20^.

## Materials and methods

### Ethical compliance

We have complied with all ethical regulations related to this study. Informed consent was obtained from all participants. Each specimen was anonymized prior to use to maintain confidentiality and the samples were labeled using a barcode for identification purposes only. The de-identified (barcoded) samples (swabs from lesions) were submitted to Mirimus Clinical Laboratory and collected within the interim guidelines for laboratory testing established by the WHO (May 23rd, 2022). Sample collection and submission methods for use in the Mirimus Clinical Laboratory study protocol were reviewed and approved by Advarra Institutional Review Board Pro00065623. No minors were included in the study.

### Phylogenomic and homolog analysis

Genome assemblies for all OPVs available as of May 18th, 2022, were downloaded from GenBank, and protein-coding genes in each assembly were annotated using PROKKA version 1.14.6 with the parameters specific for viral gene prediction^42^. Genomes of Centapoxviruses namely Yokapox virus, Murmansk virus, and Centapox NY_014 were downloaded for use as outgroups. Metadata on the source of isolation of each virus was compiled (S1 Table). Orthology analysis and identification of single-copy orthologs for phylogenomic analysis were performed using Orthofinder^43^ version 2.4.0. The gene sequences corresponding to the identified SCOs (n = 38) were concatenated together to generate a supermatrix sequence for each species, and their multiple sequence alignment was obtained using mafft^44^ v7.149b. The Centapoxviruses were used as an outgroup. Phylogenetic analysis of the resulting supermatrix of 31,857 nucleotides was trimmed using Gblocks^45^ v0.91b to remove poorly aligned positions or positions with gaps across multiple species. The final supermatrix of 17,205 nucleotides was used for phylogenetic tree construction using W-IQ-TREE^46^ where the best-fit substitution model was chosen automatically using ModelFinder^47^ and bootstrap support values were calculated based on ultrafast bootstrap^48^ with 1000 replicates. The OPV clades were named phylogroups 1 to 10 and denoted as OPV-PG-1 to OPV-PG-10. To identify orthogroups that are present or absent across various phylogroups, protein sequences from each isolate within a phylogroup were first combined into a pan-phylogroup proteome and were then used for orthology analysis using OrthoFinder^43^ version 2.4.0. The conservation of orthogroups across various phylogroups was visualized as an UpSet plot^49^.

### Analysis of sequence variation in LAMP amplicons

All available OPV genomes were downloaded from NCBI Virus database (https://www.ncbi.nlm.nih.gov/labs/virus, n = 6812 sequences in November 2023). Additionally, all MPV genomes available in the GISAID EpiPox™ database^50^, excluding low coverage sequences were also downloaded (n = 7513 in November 2023). The complete list of all GISAID sequences and their contributors is available in Supplementary Tables S3 and S4 (sequences up to 1st November 2022 and 2nd November 2022 to 02nd November 2023 respectively).

The sequences of various LAMP amplicons were first extracted from all available genomes and collected into species-specific bins, and each bin was clustered using cd-hit-est^51^ at 100% global identity threshold to produce representative sequences of all potential variants. Each representative sequence cluster was named as the virus species abbreviation and the cluster number within the species followed by the number of sequences comprising that cluster. Multiple sequence alignments of these clustered amplicons and the reference amplicon with annotated primers were performed using ClustalW^52^ in Geneious Prime 2023.2.1 (https://www.geneious.com).

### Control virus DNA and MPV samples

Genomic DNA from MPV USA-2003 (BEI, #NR-4928, Lot 70053399, at least 98% identical to GenBank accession number NC_063383) and synthetic hMPV control 2 (Twist Bioscience, # 106059, Lot 2000006347 identical to GenBank accession number NC_063383) were used as the template for LAMP assays. Genomic DNA from camelpox virus strain V78-2379 (BEI, #NR-50076, Lot 64108452, 99% identical to GenBank accession number NC_003391) and vaccinia virus WR (BEI #NR-2640, Lot 60981371, GenBank: NC_006998) were also used for specificity analysis. gBlock gene fragments that match to A4L and N1R LAMP target sequences from various poxviruses were synthesized by IDT. Three clinical MPV DNA samples and their corresponding swab eluates samples were donated by Mirimus Inc. Human genomic DNA (Promega #G3041, Lot 0000531679) was used as the negative control. Viral DNA was aliquoted into small volumes and stored at − 80 °C until use.

### Real-time PCR

Real-Time PCR was performed using the Luna^®^ Universal Probe qPCR Master (NEB #M3004) following the manufacturer’s instructions. Each 20 μL reaction contained 2 μL template DNA. CDC-Non-Variola Orthopoxvirus Forward primer (5′-TCAACTGAAAAGGCCATCTAT GA-3′), Reverse primer (5′-GAGTATAGAGCACTATTTCTAAATCCCA-3′) and dual quencher modified probe (5′-FAM-CCATGCAAT/ZEN/ATACGTACAAGATAGTAGCCAAC-3′IABkFQ) were used^53^. The reactions were performed on a Bio-Rad CFX Opus instrument using the following cycling conditions: initial denaturation (95 °C for 1 min) followed by 45 cycles of alternating denaturation (95 °C for 10 s) with annealing/elongation (60 °C for 30 s) plus a plate read step.

### Colorimetric and probe-based LAMP assay

LAMP primer design was based on MPV reference genomes NC_003310 and NC_063383. Multiple genes were chosen for LAMP primer design using the NEB Primer Design Tool (https://lamp.neb.com/). Among the 14 sets of LAMP primers (S2 Table) tested targeting various genes, 2 sets of primers showed the best performance and were chosen for this study (Table 1). Each primer set included an outer forward primer (F3), outer backward primer (B3), forward inner primer (FIP), backward inner primer (BIP), forward loop primer (LF), and backward loop primer (LB). Primers were synthesized by Integrated DNA Technologies™ (Coralville, IA, USA).

The colorimetric LAMP assay was performed using the WarmStart® Colorimetric LAMP 2X Master Mix with UDG (NEB #M1804). Each 20 μL reaction contained 10 µL 2X Master Mix, 2 µL 10X primer mix to their final concentration F3/B3 0.2 µM each; FIP/BIP 1.6 µM each; LF/LB 0.4 µM each), 2 µL 10X guanidine hydrochloride (400 mM), 2–4 µL of DNA or crude swab eluate, and DNase/RNase free water. Reactions were assembled in 96-well plates on ice followed by incubation at 65 °C for up to 1 h. Samples were considered positive for the presence of the virus if the reaction had a color change from pink to yellow, or negative if the reaction remained pink. To record color changes, 96-well plates were imaged using an Epson Perfection V600 Photo Scanner before and after the LAMP reaction. To enable reaction dynamics to be monitored in real-time, 1 µM of SYTO™ 9 Green Fluorescent Nucleic Acid Stain (Invitrogen) was included in the LAMP reaction and the reactions performed in a qPCR machine (Bio-Rad CFX Opus) for 1 h. The Cq number was converted to Tt which represents the time in minutes to reach the fluorescence detection threshold, set to be 10% of the maximum fluorescence level, using a conversion factor of 22 s per cycle. No amplification is denoted N/A. Experiments were conducted using at least two replicates.

Probe-based LAMP assays were performed with a fluorescent-based LAMP kit (NEB #E1700) with 0.25 µM MPV_Probe (/5Cy5/T + T + GTGCAA + T + AAT + TGGAC/3IAbRQSp/) instead of the fluorescent dye included in the kit. The hybridization probe contains a 5′ Cy5 fluorophore and a 3′ end dark quencher and locked nucleic acid (LNA) bases indicated by a preceding + symbol. The probe was synthesized by Integrated DNA Technologies™ (Coralville, IA, USA). All probe-based reactions were performed in a qPCR machine (Bio-Rad CFX Opus).

### Molecular testing of clinical swab samples

Clinical swab samples were collected as part of the IRB study with either paper or electronically written consent. Each specimen was anonymized prior to use. Dry swab specimens were rehydrated in 1 mL TE buffer (ThermoFisher, Catalog#J75793-AP). For the standard qPCR test, 100 µL of eluate was subjected to nucleic acid extraction and purification using the KingFisher Flex automated nucleic acid extraction (ThermoFisher, Catalog A48383), and 2.5 µL of purified DNA was amplified and detected using 4X Luna MasterMIx (NEB, Catalog#M3019B) and CDC-defined primer/probe sets (S5 Table) in a total of 5 µL of reaction. For the extraction-free LAMP test, swab eluates were treated similarly to saliva as previously described^20^. Briefly, an equal volume of eluates was mixed with an equal volume of 2X SLB buffer containing 5 mM tris(2-carboxyethyl) phosphine (TCEP, Millipore Cat# 580567), 22 mM sodium hydroxide (Sigma 72068), 2 mM Ethylenediaminetetraacetic acid (EDTA, Invitrogen 15575-038) and 0.4% Pluronic F-68 (Gibco 24040-032). Mixed samples were heated in a thermocycler at 95 °C for 5 min, then cooled to 4 °C. For each LAMP reaction, 4 μL of the treated sample, corresponding to 2 μL of swab eluate, was used in the 20 μL reaction.

### Supplementary Information

Supplementary Figure S1.

Supplementary Figure S2.

Supplementary Figure S3.

Supplementary Figure S4.

Supplementary Figure S5.

Supplementary Figure S6.

Supplementary Figure S7.

Supplementary Figure S8.

Supplementary Figure S9.

Supplementary Figure S10.

Supplementary Figure S11.

Supplementary Information.

Supplementary Table 1.

Supplementary Table 2.

Supplementary Table 3.

Supplementary Table 4.

Supplementary Table 5.

## Data Availability

All data generated or analyzed during this study are included in this published article (and its Supplementary Information files).
