# Supplementary material for: Extraction-free LAMP assays for generic detection of Old World Orthopoxviruses and specific detection of Mpox virus
Source: Sci Rep. 2023 Nov 30;13:21093. doi: 10.1038/s41598-023-48391-z (PMC10689478; doi:10.1038/s41598-023-48391-z)

Supplementary Figure S1: A mid-point rooted phylogenomic tree of 200 Orthopoxviruses, rendered in a rectangular layout.
The tree was constructed based on 38 single copy orthologs. The multiple sequence alignment supermatrix of 31,857 nucleotides was trimmed using Gblocks to remove poorly aligned to produce a final supermatrix of 17,205 nucleotides. The Centapoxviruses were used as an outgroup. The substitution model GTR+F+I+G4 was found to be the best fit for the sequences. Branch length values are indicated on respective branches. Branches are not drawn to scale. Bootstrap support values are shown at each node.
The phylogroups are color-coded and indicated by corresponding labels.

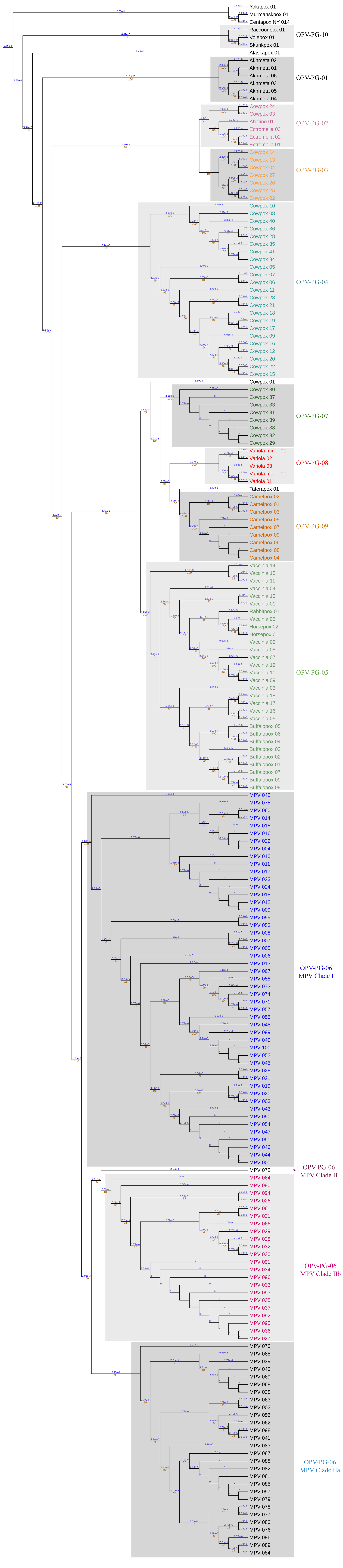

Supplement: Supplementary file 1 — Supplementary Figure S1. [file 41598_2023_48391_MOESM1_ESM.pdf]
