# Supplementary material for: Extraction-free LAMP assays for generic detection of Old World Orthopoxviruses and specific detection of Mpox virus
Source: Sci Rep. 2023 Nov 30;13:21093. doi: 10.1038/s41598-023-48391-z (PMC10689478; doi:10.1038/s41598-023-48391-z)

Supplementary Figure S2: Analysis of orthogroups shared across various pan-phylogroup proteomes.

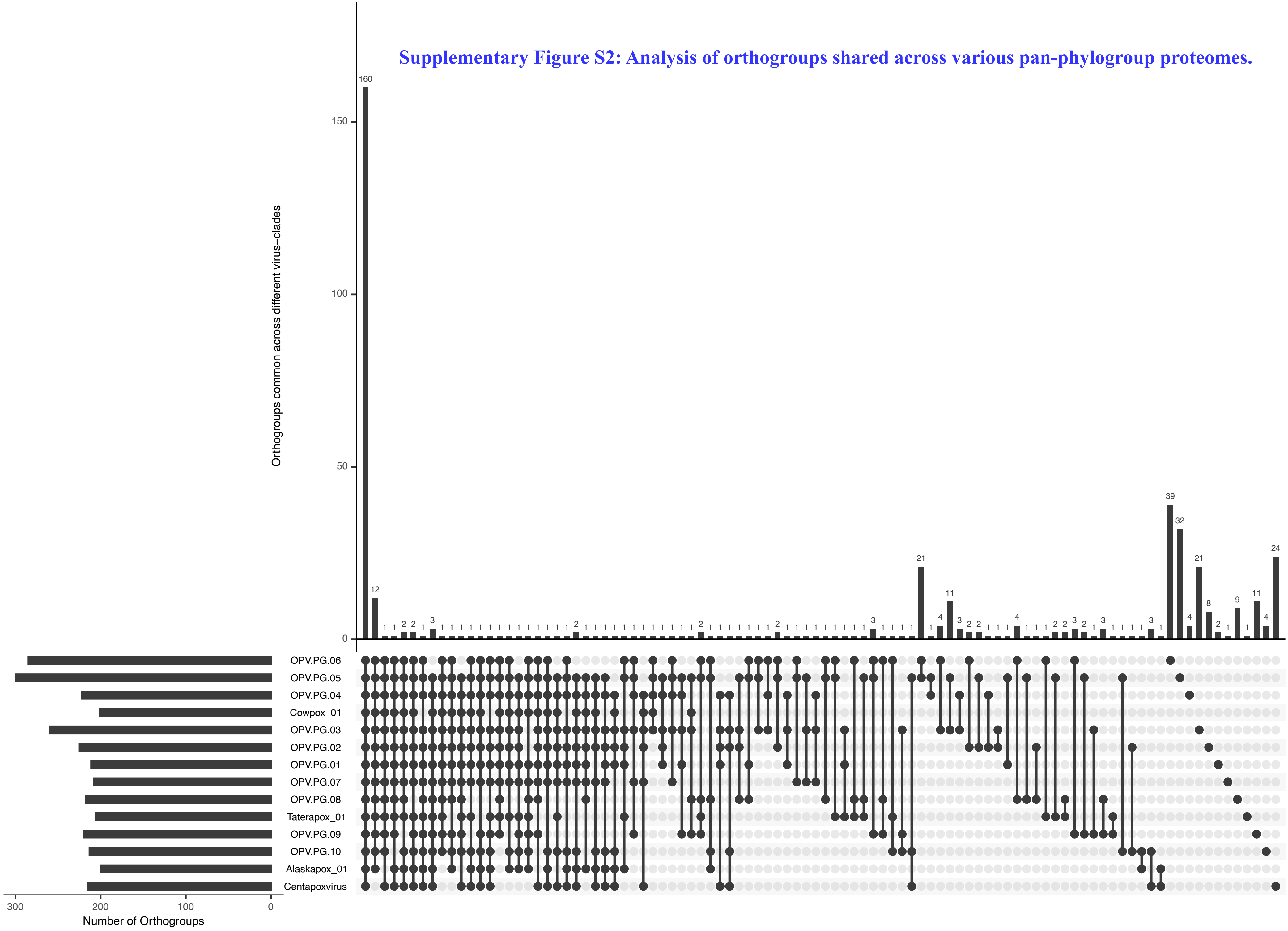

Supplement: Supplementary file 2 — Supplementary Figure S2. [file 41598_2023_48391_MOESM2_ESM.pdf]
