# Supplementary material for: Extraction-free LAMP assays for generic detection of Old World Orthopoxviruses and specific detection of Mpox virus
Source: Sci Rep. 2023 Nov 30;13:21093. doi: 10.1038/s41598-023-48391-z (PMC10689478; doi:10.1038/s41598-023-48391-z)

Supplementary Figure S3. Sensitivity of colorimetric LAMP assay. MPV A4L or N1R gBlocks were tested at 10 copies/µL (21 replicates) with 3 negative control reactions or 5 copies/µL(24 replicates) in A4L or N1R colorimetric LAMP. Scanned images of the post-amplification plate showing the colorimetric (pink = negative, yellow = positive) readouts are shown.

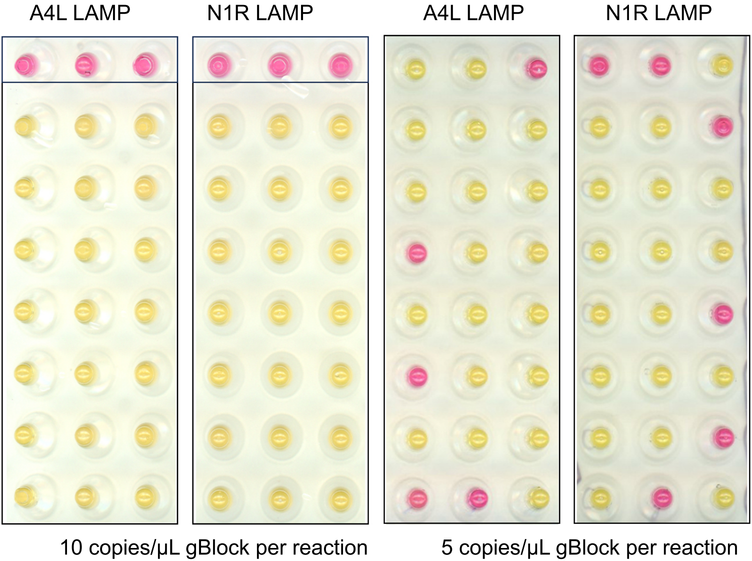

Supplement: Supplementary file 3 — Supplementary Figure S3. [file 41598_2023_48391_MOESM3_ESM.pdf]
