# Supplementary material for: Extraction-free LAMP assays for generic detection of Old World Orthopoxviruses and specific detection of Mpox virus
Source: Sci Rep. 2023 Nov 30;13:21093. doi: 10.1038/s41598-023-48391-z (PMC10689478; doi:10.1038/s41598-023-48391-z)

Supplementray Figure S4. Multiple sequence alignment of the A4L LAMP amplicon across all MPV sequences in GISAID EpiPox(TM) database

The representative variants of the A4L LAMP region were obtained by clustering of identical sequences from all MPV sequences.

The number of sequences represented by a variant are indicated after the "_n" suffix at the end of each sequence cluster name.

LAMP primers are marked in the reference amplicon at the top.

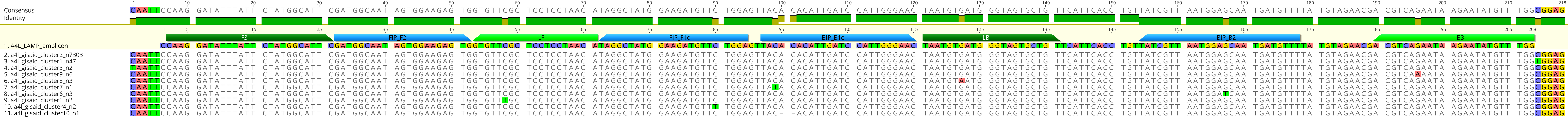

Supplement: Supplementary file 4 — Supplementary Figure S4. [file 41598_2023_48391_MOESM4_ESM.pdf]
