# Supplementary material for: Extraction-free LAMP assays for generic detection of Old World Orthopoxviruses and specific detection of Mpox virus
Source: Sci Rep. 2023 Nov 30;13:21093. doi: 10.1038/s41598-023-48391-z (PMC10689478; doi:10.1038/s41598-023-48391-z)

Supplementary Figure S5: Multiple sequence alignment of the A4L LAMP amplicon across all OPVs in NCBI Virus database

The A4 LAMP region was extracted from all 6,812 genomes available in NCBI Virus database in November 2023.
Species-specific representative sequences were generated by clustering identical sequences form each species.
Each representative cluster is named with a suffix indicating the species abbreviation followed by the serial number of the cluster within that species,
and ending with the number of sequences represented by each cluster after the "_n" suffix.
LAMP primers are marked in the reference amplicon at the top.

Abbreviations: ABMPV = Orthopoxvirus Abatino; AKHV = Ahkmeta virus; AKPV = Alaskapox virus; BPXV = Buffalopox virus; CMPV = Camelpox virus; CPV = Cowpox virus; ECTV = Ectromelia virus;
HSPV = Horsepox virus; MMPV = Murmansk poxvirus; MPV = Mpox virus; RAPV = Raccoonpox virus; RPXV = Rabbitpox virus; SKPV = Skunkpox virus; TATPV = Taterapox virus;
VACV = Vaccinia virus; VARV = Variola virus; VPXV = Volepox virus; YKV = Yokapox virus.

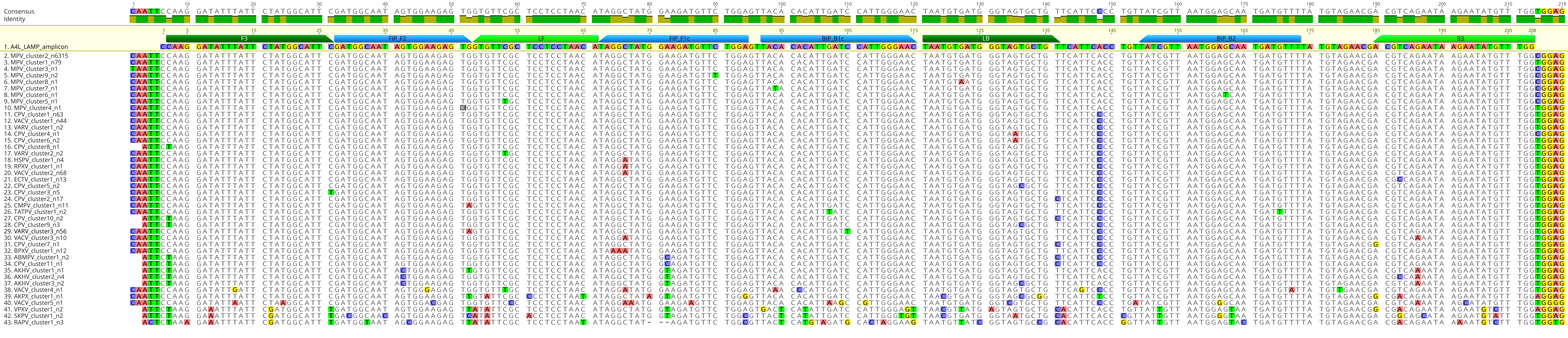

Supplement: Supplementary file 5 — Supplementary Figure S5. [file 41598_2023_48391_MOESM5_ESM.pdf]
