# Supplementary material for: Extraction-free LAMP assays for generic detection of Old World Orthopoxviruses and specific detection of Mpox virus
Source: Sci Rep. 2023 Nov 30;13:21093. doi: 10.1038/s41598-023-48391-z (PMC10689478; doi:10.1038/s41598-023-48391-z)

Supplementray Figure S6. Multiple sequence alignment of the N1R LAMP amplicon across all MPV sequences in GISAID EpiPox(TM) database

The representative variants of the N1R LAMP region were obtained by clustering of identical sequences from all MPV sequences.

The number of sequences represented by a variant are indicated after the "_n" suffix at the end of each sequence cluster name.

LAMP primers and the LNA probe are marked in the reference amplicon at the top.

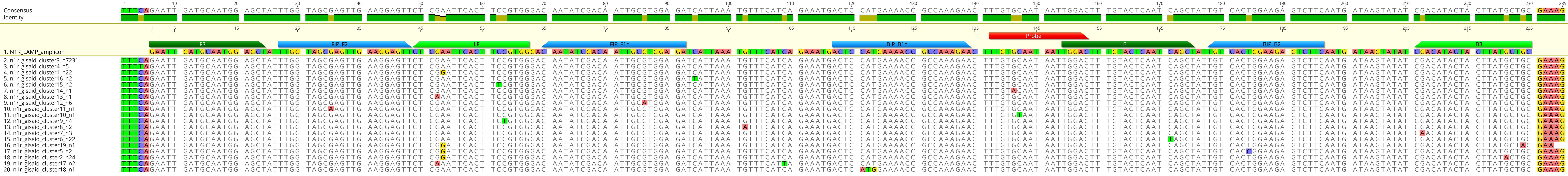

Supplement: Supplementary file 6 — Supplementary Figure S6. [file 41598_2023_48391_MOESM6_ESM.pdf]
