# Supplementary material for: Extraction-free LAMP assays for generic detection of Old World Orthopoxviruses and specific detection of Mpox virus
Source: Sci Rep. 2023 Nov 30;13:21093. doi: 10.1038/s41598-023-48391-z (PMC10689478; doi:10.1038/s41598-023-48391-z)

Supplementary Figure S7: Multiple sequence alignment of the N1R LAMP amplicon across all OPVs in NCBI Virus database

The N1R LAMP region was extracted from all 6,812 genomes available in NCBI Virus database in November 2023.
Species-specific representative sequences were generated by clustering identical sequences form each species.
Each representative cluster is named with a suffix indicating the species abbreviation followed by the serial number of the cluster within that species, and ending with the number of sequences represented by each cluster after the "_n" suffix.
LAMP primers and the LNA probe are marked in the reference amplicon at the top.

Abbreviations: ABMPV = Orthopoxvirus Abatino; AKHV = Ahkmeta virus; AKPV = Alaskapox virus; BPXV = Buffalopox virus; CMPV = Camelpox virus; CPV = Cowpox virus; ECTV = Ectromelia virus; HSPV = Horsepox virus; MMPV = Murmansk poxvirus; MPV = Mpox virus; RAPV = Raccoonpox virus; RPXV = Rabbitpox virus; SKPV = Skunkpox virus; TATPV = Taterapox virus; VACV = Vaccinia virus; VARV = Variola virus; VPXV = Volepox virus; YKV = Yokapox virus.

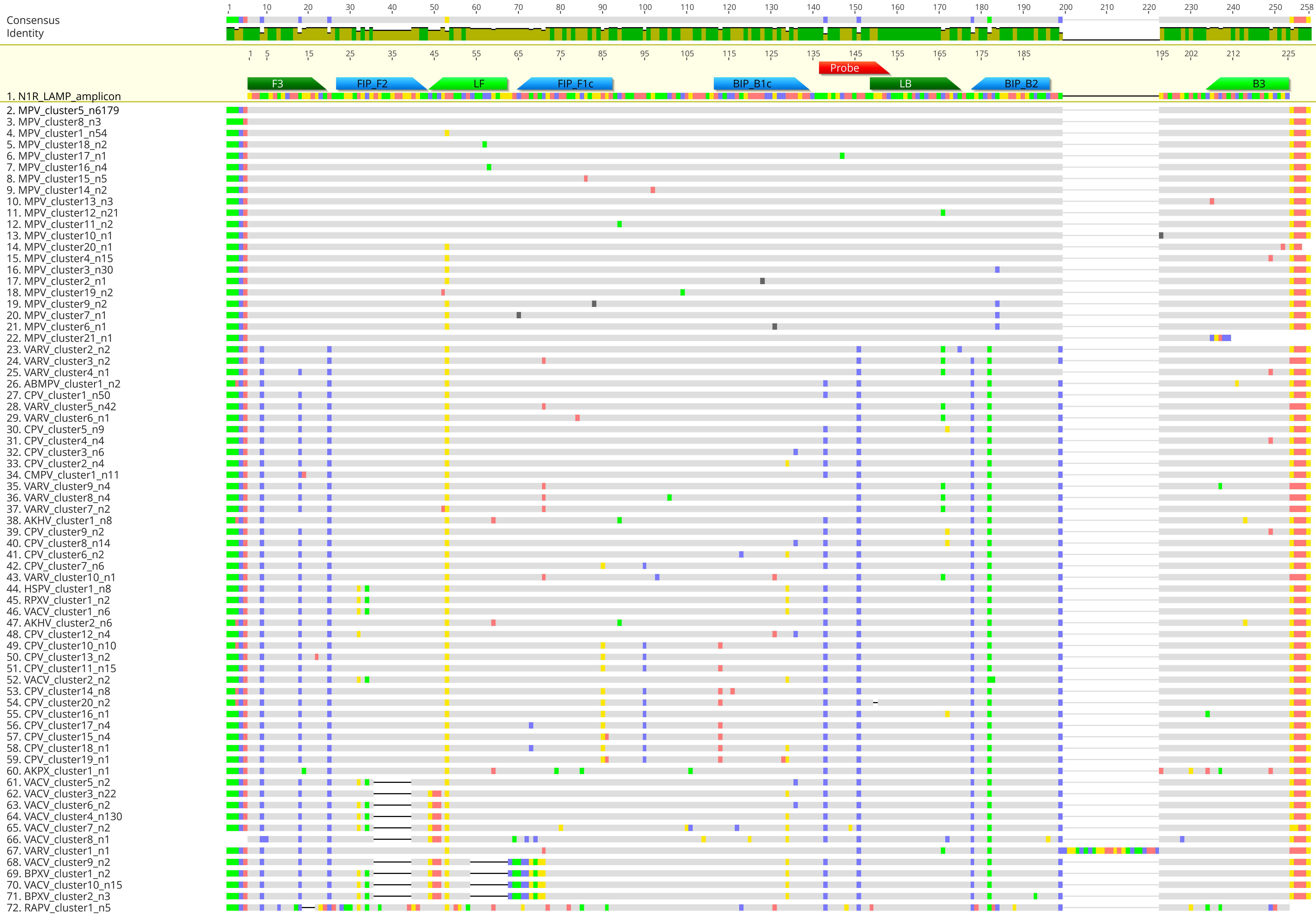

Supplement: Supplementary file 7 — Supplementary Figure S7. [file 41598_2023_48391_MOESM7_ESM.pdf]
