# Supplementary material for: Extraction-free LAMP assays for generic detection of Old World Orthopoxviruses and specific detection of Mpox virus
Source: Sci Rep. 2023 Nov 30;13:21093. doi: 10.1038/s41598-023-48391-z (PMC10689478; doi:10.1038/s41598-023-48391-z)

Supplementary Figure S8. Specificity of N1R probe-based LAMP assay. Genomic DNA from MPV (blue), CMPV (orange), and VACV (purple) were diluted 10-fold in 0.1X TE and tested in triplicate in fluorescent LAMP reactions with either generic fluorescent dye **(A)** or Cy5 labelled MPV specific probe **(B)** in a Bio-Rad Opus qPCR machine. Amplification signal was acquired every 15 seconds (total incubation time was less than 1 hour). The fluorescent amplification curves over time are shown.

A

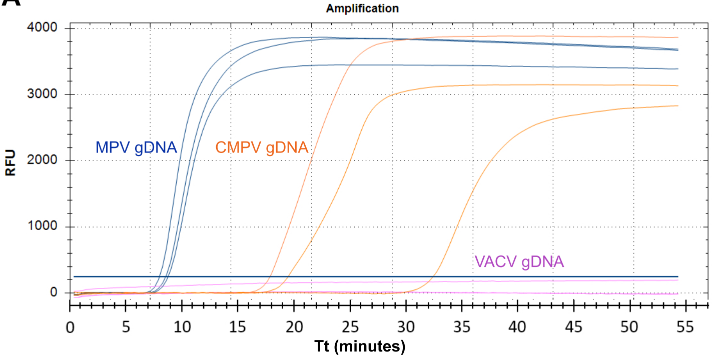

Lorem Ipsum

B

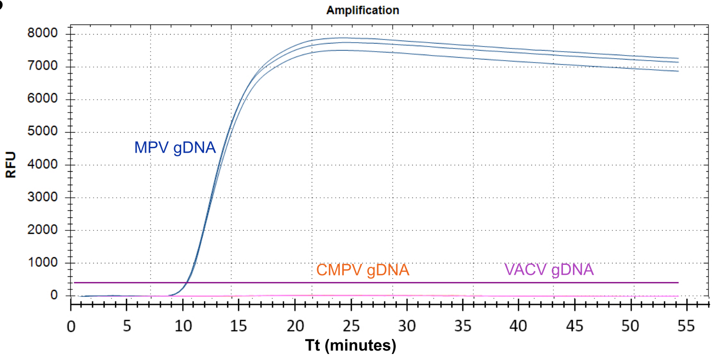

Supplement: Supplementary file 8 — Supplementary Figure S8. [file 41598_2023_48391_MOESM8_ESM.pdf]
