# Supplementary material for: Extraction-free LAMP assays for generic detection of Old World Orthopoxviruses and specific detection of Mpox virus
Source: Sci Rep. 2023 Nov 30;13:21093. doi: 10.1038/s41598-023-48391-z (PMC10689478; doi:10.1038/s41598-023-48391-z)

Supplementary Figure S9. A27L and F3L colorimetric. Synthetic human MPV control DNA from Twist was diluted in 1ng/µL of human DNA and tested in 20 µL of LAMP reactions with either A27L or F3L LAMP primers. LAMP reactions were performed at 63°C as described in the published reference. Genomic DNA from camelpox virus (CMPV), vaccinia virus (VACV), and human (NTC) were also tested. NEB WarmStart Colorimetric LAMP mix was used. Scanned images of the post-amplification plate showing the colorimetric (pink/negative, yellow/positive) readouts were shown. Experiments were performed in triplicates.

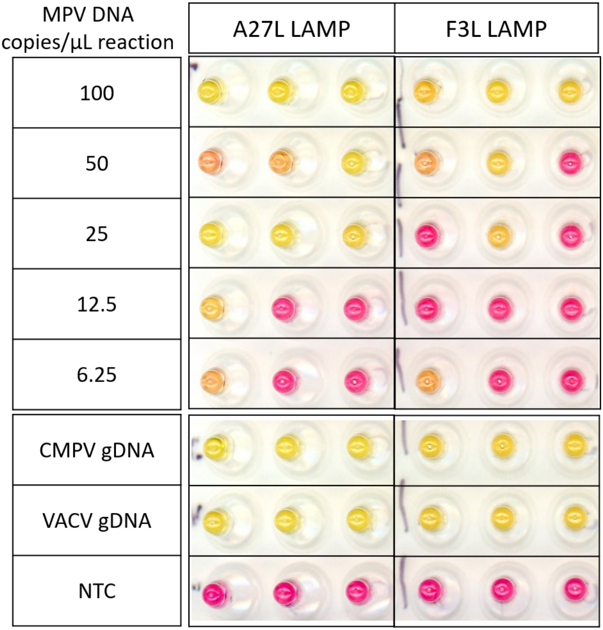

Supplement: Supplementary file 9 — Supplementary Figure S9. [file 41598_2023_48391_MOESM9_ESM.pdf]
