# Supplementary material for: Extraction-free LAMP assays for generic detection of Old World Orthopoxviruses and specific detection of Mpox virus
Source: Sci Rep. 2023 Nov 30;13:21093. doi: 10.1038/s41598-023-48391-z (PMC10689478; doi:10.1038/s41598-023-48391-z)

Supplementary Figure S9: Multiple sequence alignment of the A27L LAMP amplicon from Feng et al. 2022 across all OPVs

The representative variants of the A27L LAMP region (Feng et al. Microbiology Spectrum 2022) were obtained by clustering of identical sequences within each OPV species.
The number of sequences represented by a variant are indicated after the "_n" suffix at the end of each sequence cluster name. LAMP primers are marked in the reference amplicon at the top.

Abbreviations: ABMPV = Orthopoxvirus Abatino; AKHV = Ahkmeta virus; AKPV = Alaskapox virus; BPXV = Buffalopox virus; CMPV = Camelpox virus; CPV = Cowpox virus; ECTV = Ectromelia virus;
HSPV = Horsepox virus; MMPV = Murmansk poxvirus; MPV = Mpox virus; RAPV = Raccoonpox virus; RPXV = Rabbitpox virus; SKPV = Skunkpox virus; TATPV = Taterapox virus;
VACV = Vaccinia virus; VARV = Variola virus; VPXV = Volepox virus; YKV = Yokapox virus.

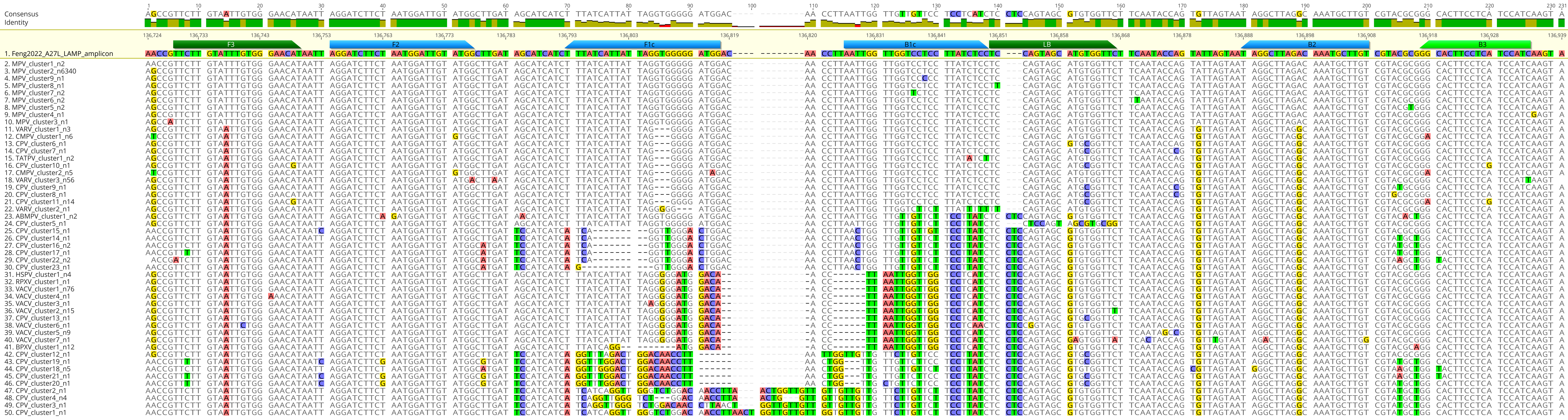

Supplement: Supplementary file 10 — Supplementary Figure S10. [file 41598_2023_48391_MOESM10_ESM.pdf]
