# Supplementary material for: Extraction-free LAMP assays for generic detection of Old World Orthopoxviruses and specific detection of Mpox virus
Source: Sci Rep. 2023 Nov 30;13:21093. doi: 10.1038/s41598-023-48391-z (PMC10689478; doi:10.1038/s41598-023-48391-z)
Supplement: Supplementary file 12 — Supplementary Information. [file 41598_2023_48391_MOESM12_ESM.docx]

**Supplementary note:**

**A4L gBlock sequences and alignment**

MPV GTTGGATTTAGACCTACGTTGGTTTCTGTGGCTAGACCTATGAACGGCATCAGTTACGAT

Alaskapox GTTGGATTTAGACCTACGTTGGTTTCTGTGGCTAGACCTATGAACGGCATCAGTTACGAT

Volepox GTTGGATTTAGACCTACGTTGGTTTCTGTGGCTAGACCTATGAACGGCATCAGTTACGAT

Skunkpox GTTGGATTTAGACCTACGTTGGTTTCTGTGGCTAGACCTATGAACGGCATCAGTTACGAT

Raccoonpox GTTGGATTTAGACCTACGTTGGTTTCTGTGGCTAGACCTATGAACGGCATCAGTTACGAT

Yokapox GTTGGATTTAGACCTACGTTGGTTTCTGTGGCTAGACCTATGAACGGCATCAGTTACGAT

************************************************************

MPV ATGAAACTTCAGGCTGCACCATACATCGTTGTTAATCCTATGAAGATGATAACAACATCC

Alaskapox ATGAAACTTCAGGCTGCACCATACATCGTTGTTAATCCTATGAAGATGATAACAACATCC

Volepox ATGAAACTTCAGGCTGCACCATACATCGTTGTTAATCCTATGAAGATGATAACAACATCC

Skunkpox ATGAAACTTCAGGCTGCACCATACATCGTTGTTAATCCTATGAAGATGATAACAACATCC

Raccoonpox ATGAAACTTCAGGCTGCACCATACATCGTTGTTAATCCTATGAAGATGATAACAACATCC

Yokapox ATGAAACTTCAGGCTGCACCATACATCGTTGTTAATCCTATGAAGATGATAACAACATCC

************************************************************

------------F3---------->-----F2-----

MPV GACAGTCCGATTTCTATCAATTCCAAGGATATTTATTCTATGGCATTCGATGGCAATAGT

Alaskapox GACAGTCCGATTTCTATCAATTCCAAGGATATTTATTCTATGGCATTCGATGGCAATAGT

Volepox GACAGTCCGATTTCTATCAATTCTAAGGAAATTTATTCGATGGCATTTGATGGCAATAGT

Skunkpox GACAGTCCGATTTCTATCAATTCTAAGGAAATTTATTCGATGGCATTTGACGGCAACAGT

Raccoonpox GACAGTCCGATTTCTATCAATTCTAAAGAAATTTATTCGATGGCATTTGATGGTAATAGC

Yokapox GACAGTCCGATTTCTATCAATTCTAGAGATATTTATTCTATGGCATTTGATAACAATAGT

*********************** * ** ******** ******** ** ** **

--F2----><------LF----------<-------F1c------------ -------

MPV GGAAGAGTGGTGTTCGCTCCTCCTAACATAGGCTATGGAAGATGTTCTGGAGTTACACAC

Alaskapox GGAAGAGTTGTATTCGCCCCTCCTAATATAGGCTATAGTAGATGTTCTGGGGTTACACAC

Volepox GGAAGAGTTATATTCGCTCCTCCTAACATAGGCTATGGTAGATGTTCTGGAGTGACTCAT

Skunkpox GGAAGAGTCATATTCGCACCTCCTAACATAGGCTATGGTAGATGTTCTGGCGTTACTCAT

Raccoonpox GGAAGAGTTATATTCGCTCCTCCTAATATAGGCTAT---AGATGTTCTGGCGTTACTCAT

Yokapox GGTAGAGTAGTCTTTGCTCCTCCAAATATTGGATATAGCGGATGTTCAGGTATAACACAT

** ***** * ** ** ***** ** ** ** *** ******* ** * ** **

-------B1c------>---------LB---------> <-----B2--

MPV ATTGATCCATTGGGAACTAATGTGATGGGTAGTGCTGTTCATTCACCTGTTATCGTTAAT

Alaskapox ATTGATCCATTGGGAACTAACGTGATGGGTAGCGCGGTTCATTCTCCTGTTATCGTTAAT

Volepox ATTGATCCATTGGGAGTTAACGTTATGAGTAGTGCTGCACATTCACCTGTTATTGTTAAT

Skunkpox ATTGATCCATTGGGTGTTAACGTGATGGGTAATGCTGCACATTCACCCGTTATTGTTAAT

Raccoonpox GTAGATGCACTAGGAAGTAATGTTATCGGTAGTGCCGCACATTCACCGGTTATTGTTAAT

Yokapox GTAGAACCGGCGGGTAGTTCTATTTTTGGTAACGCAATGAATTCACCAGTTATTGTTAAC

* ** * ** * * * *** ** **** ** ***** *****

----B2------ <----------B3-----------

MPV GGAGCAATGATGTTTTATGTAGAACGACGTCAGAATAAGAATATGTTTGGCGGAGAATGT

Alaskapox GGAGCAATGATGTTTTATGTAGAACGGCGACAGAATAAGAATATGTTTGGCGGAGAATGT

Volepox GGAGTAATGATGTTTTATGTAGAACGGCGACAGAATAAGAATGTCTTTGGCGGAGAATGT

Skunkpox GGGGTAATGATGTTTTATGTAGAACGACGGCAGCATAAGAATGTATTTGGCGGAGAATGT

Raccoonpox GGAGTACTGATGTTTTATGTAGAACGACGACAGAATAAAAATGTCTTTGGCGGAGAATGT

Yokapox GGAGTTCTAATGTTTTATGTAGAACGGCGACAGAACAAAAATGTTTTTGGCGGAGAATGT

** * * ***************** ** *** * ** *** * ***************

MPV TACACCGGCTTTAGATCTCTAATAGATGATACTCCGATTGACGTATCACCAGAAATCATG

Alaskapox TACACCGGCTTTAGATCTCTAATAGATGATACTCCGATTGACGTATCACCAGAAATCATG

Volepox TACACCGGCTTTAGATCTCTAATAGATGATACTCCGATTGACGTATCACCAGAAATCATG

Skunkpox TACACCGGCTTTAGATCTCTAATAGATGATACTCCGATTGACGTATCACCAGAAATCATG

Raccoonpox TACACCGGCTTTAGATCTCTAATAGATGATACTCCGATTGACGTATCACCAGAAATCATG

Yokapox TACACCGGCTTTAGATCTCTAATAGATGATACTCCGATTGACGTATCACCAGAAATCATG

************************************************************

MPV CTAAACGGTATCATGTATAGGTTAAAGTCCGCAGTCTGTTACAAACTCGGAGACCAATTC

Alaskapox CTAAACGGTATCATGTATAGGTTAAAGTCCGCAGTCTGTTACAAACTCGGAGACCAATTC

Volepox CTAAACGGTATCATGTATAGGTTAAAGTCCGCAGTCTGTTACAAACTCGGAGACCAATTC

Skunkpox CTAAACGGTATCATGTATAGGTTAAAGTCCGCAGTCTGTTACAAACTCGGAGACCAATTC

Raccoonpox CTAAACGGTATCATGTATAGGTTAAAGTCCGCAGTCTGTTACAAACTCGGAGACCAATTC

Yokapox CTAAACGGTATCATGTATAGGTTAAAGTCCGCAGTCTGTTACAAACTCGGAGACCAATTC

************************************************************

**gBlock sequence of variola N1R mimic and its alignment with N1R gene from MPXV and variola**

NC_063383_Monkeypox_USA2003 ATGGCCTCTCCTTGTGCCCAGTTCAGTCCCTGTCATTGCCACGCTACTAA

NC_003310_Monkeypox_Zaire ATGGCCTTTCCTTGTGCCCAGTTCAGTCCCTGTCATTGCCACGCTACTAA

MPXV_N1R_Variola_Mimic_New ATGGCCTTTCCTTGTGCCCAGTTCAGTCCCTGTCATTGCCACGCTACTAA

LR800247.1_Variola ATGGCCTCTCCTTGTGCCCAGTTCAGTCCCTGTCATTGCCACGCTACTAA

******* ******************************************

NC_063383_Monkeypox_USA2003 GGACTCCCTGAATACCGTGACTGACGTCAGACATTGTCTGACTGAATACA

NC_003310_Monkeypox_Zaire GGACTCCCTGAATACCGTGACTGACGTCAGACATTGTCTGACTGAATACA

MPXV_N1R_Variola_Mimic_New GGACTCCCTGAATACCGTGACTGACGTCAGACATTGTCTGACTGAATACA

LR800247.1_Variola GGACTCCCTGAATACCGTGGCCGACGTCAGACATTGTCTGACTGAATACA

******************* * ****************************

NC_063383_Monkeypox_USA2003 TCCTGTGGGTTTCTCATAGATGGACCCATAGAGAAAGCGCAGGGCCTCTC

NC_003310_Monkeypox_Zaire TCCTGTGGGTTTCTCATAGATGGACCCATAGAGAAAGCGCAGGGCCTCTC

MPXV_N1R_Variola_Mimic_New TCCTGTGGGTTTCTCATAGATGGACCCATAGAGAAAGCGCAGGGCCTCTC

LR800247.1_Variola TCCTGTGGGTTTCTCATAGATGGACCCATAGAGAAAGCGCAGGGTCTCTC

******************************************** *****

NC_063383_Monkeypox_USA2003 TACAGGCTTCTCATCTCTTTCAGAA**T**TGATGCAATGGAGCT**A**TTTGGTAG

NC_003310_Monkeypox_Zaire TACAGGCTTCTCATCTCTTTCAGAA**T**TGATGCAATGGAGCT**A**TTTGGTAG

MPXV_N1R_Variola_Mimic_New TACAGGCTTCTCATCTCTTTCAGAA**C**TGATGCAATGGAGCT**C**TTTGGTAG

LR800247.1_Variola TACAGGCTTCTCATCTCTTTCAGAA**C**TGATGCAATGGAGCT**C**TTTGGTAG

************************* *************** ********

---------F3--------> ----

NC_063383_Monkeypox_USA2003 CGAGTTGAAGGAGTTCTCG**A**ATTCACTTCCGTGGGACAATATCGACAATT

NC_003310_Monkeypox_Zaire CGAGTTGAAGGAGTTCTCG**G**ATTCACTTCCGTGGGACAATATCGACAATT

MPXV_N1R_Variola_Mimic_New CGAGTTGAAGGAGTTCTCG**G**ATTCACTTCCGTGGGACAATATCGACAATT

LR800247.1_Variola CGAGTTGAAGGAGTTCTCG**G**ATTCACTTCCGTGGGACAATATCGACAATT

******************* ******************************

----F2--------><------LF---------- <-------F1c---

NC_063383_Monkeypox_USA2003 GCGTGGAGATCATTAAATGTTTCATCAGAAATGACTCCATGAAAACCGCC

NC_003310_Monkeypox_Zaire GCGTGGAGATCATTAAATGTTTCATCAGAAATGACTCCATGAAAACCGCC

MPXV_N1R_Variola_Mimic_New GCGTGGAGATCATTAAATGTTTCATCAGAAATGACTCCATGAAAACCGCC

LR800247.1_Variola GCGTGGAGATCATTAAATGTTTCATCAGAAATGACTCCATGAAAACCGCC

**************************************************

--------- -------B1c-------

NC_063383_Monkeypox_USA2003 AAAGAACT**TTGTGCAATAATTGGAC**TTTGTACTCAAT**C**AGCTATTGTC**A**C

NC_003310_Monkeypox_Zaire AAAGAACTTTGTGCAAT**A**ATTGGACTTTGTACTCAAT**C**AGCTATTGTC**A**C

MPXV_N1R_Variola_Mimic_New AAAGAACTTTGTGCAAT**C**ATTGGACTTTGTACTCAAT**T**AGCTATTGTC**T**C

LR800247.1_Variola AAAGAACTTTGTGCAAT**C**ATTGGACTTTGTACTCAAT**T**AGCCATTGTC**T**C

***************** ******************* *** ****** *

--> ---------LB---------> <-----

NC_063383_Monkeypox_USA2003 TGGAAGAGTCTTCAATGATAAGTATATCGACATACTACTTATGCTGCGAA

NC_003310_Monkeypox_Zaire TGGAAGAGTCTTCAATGATAAGTATATCGACATACTACTTATGCTGCGAA

MPXV_N1R_Variola_Mimic_New TGGAAGAGTCTTCAATGATAAGTATATCGACATACTACTTATGCTGCGAA

LR800247.1_Variola TGGAAGAGTCTTCAACGATAAGTATATCGACATACTACTTATGCTGCGAA

*************** **********************************

-----B2------ <-------B3----------

NC_063383_Monkeypox_USA2003 AGATTCTGAACGAGAACGACTATCTCACCCTCTTGGATCATATCCTCACT

NC_003310_Monkeypox_Zaire AGATTCTGAATGAGAACGACTATCTCACCCTCTTGGATCATATCCTCACT

MPXV_N1R_Variola_Mimic_New AGATTCTGAATGAGAACGACTATCTCACCCTCTTGGATCATATCCTCACT

LR800247.1_Variola AGATTCTGAACGAGAACGACTATCTCACCCTCTTGGATCATATCCGCACT

********** ********************************** ****
