# Supplementary material for: Extraction-free LAMP assays for generic detection of Old World Orthopoxviruses and specific detection of Mpox virus
Source: Sci Rep. 2023 Nov 30;13:21093. doi: 10.1038/s41598-023-48391-z (PMC10689478; doi:10.1038/s41598-023-48391-z)
Supplement: Supplementary file 15 — Supplementary Table 3. [file 41598_2023_48391_MOESM15_ESM.pdf]

We gratefully acknowledge the following Authors from the Originating laboratories responsible for obtaining the specimens, as well as the Submitting laboratories where the genome data were generated and shared via GISAID, on which this research is based.

All Submitters of data may be contacted directly via www.gisaid.org

Authors are sorted alphabetically.

Accession ID	Originating Laboratory	Submitting Laboratory	Authors
EPI_ISL_13052263	Microbiol Genomics and Bioinformatics, Bundeswehr Institute of Microbiology	Microbiol Genomics and Bioinformatics, Bundeswehr Institute of Microbiology	Antwerpen,M.H., Lang,D., Zange,S., Walter,M.C. and Woelfel,R.
EPI_ISL_13052266, EPI_ISL_13052267, EPI_ISL_13052268, EPI_ISL_13052269, EPI_ISL_13052270, EPI_ISL_13052272, EPI_ISL_13052273	Instituto Nacional de Saude Doutor Ricardo Jorge (INSA)	Instituto Nacional de Saude Doutor Ricardo Jorge (INSA)	Joana Isidro, Vítor Borges, Miguel Pinto, Daniel Sobral, João Dourado Santos, Alexandra Nunes, Verónica Mixão, Rita Ferreira, Daniela Santos, Silvia Duarte, Luís Vieira, Maria José Borrego, Sofia Núncio, Isabel Lopes de Carvalho, Ana Pelerito, Rita Cordeiro, João Paulo Gomes
EPI_ISL_13052274	Laboratory of Virology, University Hospitals of Geneva	Laboratory of Virology, University Hospitals of Geneva	Laubscher,F., Chudzinski,V., Schibler,M., Kaiser,L. and Renzoni,A.
EPI_ISL_13052275	IHAP, VIRAL, Universite de Toulouse, INRAE, ENVT	IHAP, VIRAL, Universite de Toulouse, INRAE, ENVT	Croville,G., Walch,M., Guerin,J.-L., Mansuy,J.-M., Pasquier,C. and Izopet,J.
EPI_ISL_13052276	Laboratory of Virology, INMI Lazzaro Spallanzani IRCCS	Laboratory of Virology, INMI Lazzaro Spallanzani IRCCS	Gruber,C.E.M., Rueca,M., Gramigna,G., Carletti,F., Butera,O., Fabeni,L., Specchiarello,E., Meschi,S., Colavita,F., Minosse,C., Francalancia,M., Lapa,D., Garbuglia,A.R. and Giombini,E.
EPI_ISL_13052277	Public Health Virology, Erasmus Medical Centre	Public Health Virology, Erasmus Medical Centre	Oude Munnink,B.B., Boter,M., Wellers,B., Molenkamp,R., Sikkema,R.S. and Koopmans,M.
EPI_ISL_13052278	Research and Evaluation, UKHSA	Research and Evaluation, UKHSA	Osman,K.L., Lewandowski,K.S., Pullan,S.T., Carter,D.P., Crook,J.M., Vipond,R. and Chand,M.
EPI_ISL_13052279, EPI_ISL_13052280, EPI_ISL_13052281	Research and Evaluation, UKHSA	Research and Evaluation, UKHSA	Osman,K.L., Lewandowski,K.S., Carter,D.P., Crook,J.M., Pullan,S.T., Vipond,R. and Chand,M.
EPI_ISL_13052282	Microbiology, Immunology and Transplantation, KU Leuven, Rega Institute	Microbiology, Immunology and Transplantation, KU Leuven, Rega Institute	Vanmechelen,B., Wawina-Bokalanga,T., Logist,A.-S., Sinnesael,R., Ysebaert,L., Verlinden,J., Bloemen,M. and Maes,P.
EPI_ISL_13052283	Microbiology, Immunology and Transplantation, KU Leuven, Rega Institute	Microbiology, Immunology and Transplantation, KU Leuven, Rega Institute	Wawina-Bokalanga,T., Vanmechelen,B., Logist,A.-S., Sinnesael,R., Ysebaert,L., Verlinden,J., Bloemen,M. and Maes,P.
EPI_ISL_13052284	Microbiology, Hospital Universitari Germans Trias i Pujol	Microbiology, Hospital Universitari Germans Trias i Pujol	Martinez-Puchol,S., Coello,A., Bordoy,A.E., Soler,L., Panisello,D., Gonzalez-Gomez,S., Clara,G., Paris de Leon,A., Not,A., Hernandez,A., Bofill-Mas,S., Saludes,V., Blanco,I., Martro,E. and Cardona,P.-J.
EPI_ISL_13052285	Laboratory of Virology, University Hospitals of Geneva	Laboratory of Virology, University Hospitals of Geneva	Laubscher,F., Schibler,M., Kaiser,L. and Renzoni,A.
EPI_ISL_13052287	Virology, GENomique EPIdemiologique des maladies Infectieuses	Virology, GENomique EPIdemiologique des maladies Infectieuses	unknown
EPI_ISL_13052288	Department of Health, Utah Public Health Laboratory	Department of Health, Utah Public Health Laboratory	Young,E.L., Hergert,J. and Oakeson,K.F.
EPI_ISL_13052289	Centers for Disease Control & Prevention (CDC), Division of High Consequence Pathogens and Pathology (DHCPP-PRB)	Centers for Disease Control & Prevention (CDC), Division of High Consequence Pathogens and Pathology (DHCPP-PRB)	Gigante,C.M., Smole,S., Seabolt,M.H., Wilkins,K., McCollum,A., Hutson,C., Davidson,W., Rao,A., Brown,C. and Li,Y.
EPI_ISL_13052290	Laboratory for Diagnostics of Zoonoses and WHO Centre, Institute of Microbiology and Immunology, Faculty of Medicine, University of Ljubljana	Laboratory for Diagnostics of Zoonoses and WHO Centre, Institute of Microbiology and Immunology, Faculty of Medicine, University of Ljubljana	Zakotnik,S., Vlaj,D., Suljic,A., Zorec,T.M., Korva,M., Poljak,M. and Avsic Zupanc,T.
EPI_ISL_13052291	Laboratory for Diagnostics of Zoonoses and WHO Centre, Institute of Microbiology and Immunology, Faculty of Medicine, University of Ljubljana	Laboratory for Diagnostics of Zoonoses and WHO Centre, Institute of Microbiology and Immunology, Faculty of Medicine, University of Ljubljana	Zakotnik,S., Vlaj,D., Suljic,A., Zorec,T.M., Skubic,C., Rozman,D., Korva,M., Poljak,M. and Avsic Zupanc,T.
EPI_ISL_13052292	Victorian Infectious Diseases Reference Laboratory, Doherty Institute	Victorian Infectious Diseases Reference Laboratory, Doherty Institute	Hammerschlag,Y., MacLeod,G., Papadakis,G., Adan-Sanchez,A., Druce,J.D., Williamson,D.A., Cheng,A.C. and McMahon,J.H.
EPI_ISL_13052293, EPI_ISL_13052294	Centre for Biological Threats, Highly Pathogenic Viruses, Robert Koch Institute	Centre for Biological Threats, Highly Pathogenic Viruses, Robert Koch Institute	Brinkmann,A., Kohl,C., Uddin,S., Pape,K., Schrick,L., Michel,J., Schaade,L. and Nitsche,A.
EPI_ISL_13052295	SC (UCO) Igiene e Sanità Pubblica, ASUGI, Trieste	Genomics and Epigenomics, AREA Science Park	Licastro,D., DeGasperi,M., Negri,C., Piscianz,E., Koncan,R., Dal Monego,S., Segat,L. and D'Agaro,P.
EPI_ISL_13053218	National Center for Infectious Diseases, Centers for Disease Control and Prevention	National Center for Infectious Diseases, Centers for Disease Control and Prevention	Likos,A.M., Sammons,S.A., Olson,V.A., Frace,A.M., Li,Y., Olsen-Rasmussen,M., Davidson,W., Galloway,R., Khristova,M.L., Reynolds,M.G., Zhao,H., Carroll,D.S., Curns,A., Formenty,P., Esposito,J.J., Regnery,R.L. and Damon,I.K.
EPI_ISL_13056233, EPI_ISL_13056234, EPI_ISL_13056235, EPI_ISL_13056236, EPI_ISL_13056237, EPI_ISL_13056238, EPI_ISL_13056239, EPI_ISL_13056240, EPI_ISL_13056241, EPI_ISL_13056242, EPI_ISL_13056243, EPI_ISL_13056244, EPI_ISL_13056245, EPI_ISL_13056246, EPI_ISL_13056247, EPI_ISL_13056248, EPI_ISL_13056249, EPI_ISL_13056250, EPI_ISL_13056251, EPI_ISL_13056252, EPI_ISL_13056253, EPI_ISL_13056254, EPI_ISL_13056255			
see above	USAMRIID, Center for Genome Sciences, United States Army Medical Research Institute of Infectious Diseases	USAMRIID, Center for Genome Sciences, United States Army Medical Research Institute of Infectious Diseases	Kugelman,J.R., Johnston,S.C., Mulembakani,P.M., Kisalu,N., Lee,M.S., Koroleva,G., McCarthy,S.E., Gestole,M.C., Wolfe,N.D., Fair,J.N., Schneider,B.S., Wright,L.L., Huggins,J., Whitehouse,C.A., Wemakoy,E.O., Muyembe-Tamfum,J.J., Hensley,L.E., Palacios,G.F. and Rimoin,A.W.
EPI_ISL_13056256, EPI_ISL_13056257, EPI_ISL_13056258, EPI_ISL_13056259, EPI_ISL_13056260, EPI_ISL_13056261, EPI_ISL_13056262, EPI_ISL_13056263, EPI_ISL_13056264, EPI_ISL_13056265, EPI_ISL_13056266, EPI_ISL_13056267, EPI_ISL_13056268			
see above	PRB, US Centers for Disease Control and Prevention, 1600 Clifton Rd, Atlanta, GA 30333, USA	PRB, US Centers for Disease Control and Prevention, 1600 Clifton Rd, Atlanta, GA 30333, USA	Mccollum,A.M., Nakazawa,Y., Ndongala,G.M., Pukuta,E., Karhemere,S., Muyembe,J.-J., Lushima,R.S., Ilunga,B.K., Kabamba,J., Wilkins,K., Gao,J., Li,Y., Damon,I.K., Carroll,D.S., Malekani,J. and Reynolds,M.G.
EPI_ISL_13056269	Centers for Disease Control and Prevention	Centers for Disease Control and Prevention	Mccollum,A.M., Nakazawa,Y., Ndongala,G.M., Pukuta,E., Karhemere,S., Muyembe,J.-J., Lushima,R.S., Ilunga,B.K., Kabamba,J., Wilkins,K., Gao,J., Li,Y., Damon,I.K., Carroll,D.S., Malekani,J. and Reynolds,M.G.
EPI_ISL_13056270	PRB, US Centers for Disease Control and Prevention	PRB, US Centers for Disease Control and Prevention	Mccollum,A.M., Nakazawa,Y., Ndongala,G.M., Pukuta,E., Karhemere,S., Muyembe,J.-J., Lushima,R.S., Ilunga,B.K., Kabamba,J., Wilkins,K., Gao,J., Li,Y., Damon,I.K., Carroll,D.S., Malekani,J. and Reynolds,M.G.
EPI_ISL_13056271, EPI_ISL_13056272, EPI_ISL_13056273, EPI_ISL_13056274	Centers for Disease Control and Prevention	Centers for Disease Control and Prevention	Mauldin,M.R., McCollum,A.M., Nakazawa,Y.J., Mandra,A., Whitehouse,E.R., Davidson,W., Zhao,H., Gao,J., Li,Y., Doty,J., Yinka-Ogunleye,A., Akinpelu,A., Aruna,O., Naidoo,D., Lewandowski,K., Afrough,B., Graham,V., Aarons,E., Hewson,R., Vipond,R., Dunning,J., Chand,M., Brown,C., Cohen-Gihon,I., Erez,N., Shifman,O., Israeli,O., Sharon,M., Schwartz,E., Beth-Din,A., Zvi,A., Mak,T.M., Ng,Y.K., Cui,L., Lin,R.T.P., Olson,V.A., Brooks,T., Paran,N., Ihekweazu,C. and Reynolds,M.G.
EPI_ISL_13056275, EPI_ISL_13056276, EPI_ISL_13056277, EPI_ISL_13056278, EPI_ISL_13056279, EPI_ISL_13056280, EPI_ISL_13056281	Centers for Disease Control and Prevention	Centers for Disease Control and Prevention	Yinka-Ogunleye,A., Aruna,O., Dalhat,M., Ogoina,D., McCollum,A., Disu,Y., Mamadu,I., Akinpelu,A., Ahmad,A., Burga,J., Ndoreraho,A., Nkunzimana,E., Manneh,L., Mohammed,A., Adeoye,O., Tom-Aba,D., Silenou,B., Ipadeola,O., Saleh,M., Adeyemo,A., Nwadiutor,I., Aworabhi,N., Uke,P., John,D., Wakama,P., Reynolds,M., Mauldin,M., Doty,J., Wilkins,K., Musa,J., Khalakdina,A., Adedeji,A., Mba,N., Ojo,O., Krause,G. and Ihekweazu,C.
EPI_ISL_13056282, EPI_ISL_13056283, EPI_ISL_13056284, EPI_ISL_13056285, EPI_ISL_13056286	Centers for Disease Control and Prevention	Centers for Disease Control and Prevention	Mauldin,M.R., McCollum,A.M., Nakazawa,Y.J., Mandra,A., Whitehouse,E.R., Davidson,W., Zhao,H., Gao,J., Li,Y., Doty,J., Yinka-Ogunleye,A., Akinpelu,A., Aruna,O., Naidoo,D., Lewandowski,K., Afrough,B., Graham,V., Aarons,E., Hewson,R., Vipond,R., Dunning,J., Chand,M., Brown,C., Cohen-Gihon,I., Erez,N., Shifman,O., Israeli,O., Sharon,M., Schwartz,E., Beth-Din,A., Zvi,A., Mak,T.M., Ng,Y.K., Cui,L., Lin,R.T.P., Olson,V.A., Brooks,T., Paran,N., Ihekweazu,C. and Reynolds,M.G.
EPI_ISL_13056287, EPI_ISL_13056288	Institut Pasteur of Bangui	Department of virology, Institut Pasteur	Nakoune,E., Lampaert,E., Ndjapou,S.G., Janssens,C., Zuniga,I., Van Herp,M., Fongbia,J.P., Koyazegbe,T.D., Selekon,B., Komoyo,G.F., Garba-Ouangole,S.M., Manengu,C., Manuguerra,J.-C., Kazanji,M., Gessain,A. and Berthet,N.
EPI_ISL_13056289	Biochemistry and Molecular Biology, Israel Institute for Biological Research	Biochemistry and Molecular Biology, Israel Institute for Biological Research	Cohen Gihon,I., Israeli,O., Shifman,O., Erez,N., Melamed,S., Paran,N., Beth-Din,A. and Zvi,A.
EPI_ISL_13056556	Walter Reed Army Institute of Research	Biochemistry and Microbiology, University of Victoria	Chen,N., Li,G., Liszewski,M.K., Atkinson,J.P., Jahrling,P.B., Feng,Z., Schriewer,J., Buck,C., Wang,C., Lefkowitz,E.J., Esposito,J.J., Harms,T., Damon,I.K., Roper,R.L., Upton,C. and Buller,R.M.
EPI_ISL_13056891	Centre for Clinical Infection & Diagnostics Research, King's College London, St Thomas Hospital	Centre for Clinical Infection & Diagnostics Research	Alcolea-Medina,A., Charalampous,T., Snell,L.B., Batra,R. and Edgeworth,J.D.
EPI_ISL_13056892, EPI_ISL_13056893, EPI_ISL_13056894, EPI_ISL_13056895, EPI_ISL_13056896, EPI_ISL_13056897, EPI_ISL_13056899, EPI_ISL_13056900, EPI_ISL_13056901, EPI_ISL_13056902, EPI_ISL_13056903, EPI_ISL_13056904, EPI_ISL_13056905, EPI_ISL_13056906, EPI_ISL_13056907, EPI_ISL_13056908, EPI_ISL_13056909			
see above	Instituto Nacional de Saude Doutor Ricardo Jorge (INSA)	Instituto Nacional de Saude Doutor Ricardo Jorge (INSA)	Joana Isidro, Vítor Borges, Miguel Pinto, Daniel Sobral, João Dourado Santos, Alexandra Nunes, Verónica Mixão, Rita Ferreira, Daniela Santos, Silvia Duarte, Luís Vieira, Maria José Borrego, Sofia Núncio, Isabel Lopes de Carvalho, Ana Pelerito, Rita Cordeiro, João Paulo Gomes
EPI_ISL_13056910	Biochemistry and Molecular Genetics, Israel Institute for Biological Research	Biochemistry and Molecular Genetics, Israel Institute for Biological Research	Israeli,O., Guedj-Dana,Y., Lazar,S., Shifman,O., Erez,N., Weiss,S., Paran,N., Israely,T., Schuster,O., Zvi,A., Beth-Din,A. and Cohen Gihon,I.
EPI_ISL_13058395, EPI_ISL_13058396, EPI_ISL_13058397, EPI_ISL_13058398, EPI_ISL_13058399, EPI_ISL_13058400, EPI_ISL_13058401, EPI_ISL_13058402, EPI_ISL_13058403	Poxvirus Section, VEHB, DVRD, NCID, Centers for Disease Control and Prevention	Poxvirus Section, VEHB, DVRD, NCID, Centers for Disease Control and Prevention	Esposito,J.J., Ropp,S.L., Jin,Q., Cai,B., Knight,J.C., Yu,L., Taubenberger,J.K., Tsai,M.M., Nowotny,N., Meyer,H. and Cavallaro,K.F.
EPI_ISL_13058404, EPI_ISL_13058405	National Center for Infectious Diseases, Centers for Disease Control and Prevention	National Center for Infectious Diseases, Centers for Disease Control and Prevention	Likos,A.M., Sammons,S.A., Olson,V.A., Frace,A.M., Li,Y., Olsen-Rasmussen,M., Davidson,W., Galloway,R., Khristova,M.L., Reynolds,M.G., Zhao,H., Carroll,D.S., Curns,A., Formenty,P., Esposito,J.J., Regnery,R.L. and Damon,I.K.
EPI_ISL_13058406, EPI_ISL_13058407, EPI_ISL_13058408, EPI_ISL_13058409, EPI_ISL_13058410, EPI_ISL_13058411, EPI_ISL_13058412, EPI_ISL_13058413, EPI_ISL_13058414, EPI_ISL_13058415	Bundeswehr Institute of Microbiology	Bundeswehr Institute of Microbiology	Rimoin,A.W., Kisalu,N., Kebela-Ilunga,B., Mukaba,T., Wright,L.L., Formenty,P., Wolfe,N.D., Shongo,R.L., Tshioko,F., Okitolonda,E., Muyembe,J.J., Ryder,R.W. and Meyer,H.
EPI_ISL_13058416	Bundeswehr Institute of Microbiology	Bundeswehr Institute of Microbiology	Rimoin,A., Kisalu,N., Mukaba,T., Ryder,R., Formenty,P., Kebela,B., Okitolonda,E., Muyembe,J. and Meyer,H.

EPI_ISL_13058417	Bundeswehr Institute of Microbiology	Bundeswehr Institute of Microbiology	Rimoin,A.W., Kisalu,N., Kebela-Ilunga,B., Mukaba,T., Wright,L.L., Formenty,P., Wolfe,N.D., Shongo,R.L., Tshioko,F., Okitolonda,E., Muyembe,J.J., Ryder,R.W. and Meyer,H.
EPI_ISL_13058418	Bundeswehr Institute of Microbiology	Bundeswehr Institute of Microbiology	Rimoin,A., Kisalu,N., Mukaba,T., Ryder,R., Formenty,P., Kebela,B., Okitolonda,E., Muyembe,J. and Meyer,H.
EPI_ISL_13058419, EPI_ISL_13058420, EPI_ISL_13058421, EPI_ISL_13058422, EPI_ISL_13058423, EPI_ISL_13058424, EPI_ISL_13058425, EPI_ISL_13058426, EPI_ISL_13058427, EPI_ISL_13058428, EPI_ISL_13058429, EPI_ISL_13058430, EPI_ISL_13058431, EPI_ISL_13058432, EPI_ISL_13058433, EPI_ISL_13058434, EPI_ISL_13058435, EPI_ISL_13058436, EPI_ISL_13058437, EPI_ISL_13058438, EPI_ISL_13058439, EPI_ISL_13058440, EPI_ISL_13058441, EPI_ISL_13058442, EPI_ISL_13058443, EPI_ISL_13058444, EPI_ISL_13058445, EPI_ISL_13058446, EPI_ISL_13058447, EPI_ISL_13058448, EPI_ISL_13058449, EPI_ISL_13058450, EPI_ISL_13058451, EPI_ISL_13058452, EPI_ISL_13058453, EPI_ISL_13058454, EPI_ISL_13058455			
see above	Bundeswehr Institute of Microbiology	Bundeswehr Institute of Microbiology	Rimoin,A.W., Kisalu,N., Kebela-Ilunga,B., Mukaba,T., Wright,L.L., Formenty,P., Wolfe,N.D., Shongo,R.L., Tshioko,F., Okitolonda,E., Muyembe,J.J., Ryder,R.W. and Meyer,H.
EPI_ISL_13058456	Vaccine and Gene Therapy Institute, Oregon Health and Science University	Vaccine and Gene Therapy Institute, Oregon Health and Science University	Estep,R.D., Messaoudi,I., O'Connor,M.A., Li,H., Sprague,J., Barron,A., Engelmann,F., Yen,B., Powers,M.F., Jones,J.M., Robinson,B.A., Orzechowska,B.U., Manoharan,M., Legasse,A., Planer,S., Wilk,J., Axthelm,M.K. and Wong,S.W.
EPI_ISL_13058457, EPI_ISL_13058458	Department of Virology, Institut Pasteur	Department of Virology, Institut Pasteur	Berthet,N., Nakoune,E., Whist,E., Selekon,B., Burguiere,A.M., Manuguerra,J.C., Gessain,A. and Kazanji,M.
EPI_ISL_13058459, EPI_ISL_13058460, EPI_ISL_13058461	Biochemistry and Microbiology, University of Victoria	Biochemistry and Microbiology, University of Victoria	Nakazawa,Y., Emerson,G.L., Carroll,D.S., Zhao,H., Li,Y., Reynolds,M.G., Karem,K.L., Olson,V.A., Lash,R.R., Davidson,W.B., Smith,S.K., Levine,R.S., Regnery,R.L., Sammons,S.A., Frace,M.A., Mutasim,E.M., Karsani,M.E., Muntasir,M.O., Babiker,A.A., Opoka,L., Chowdhary,V. and Damon,I.K.
EPI_ISL_13058462, EPI_ISL_13058463	Center for Genome Sciences, USAMRIID	Center for Genome Sciences, USAMRIID	Faye,O., Pratt,C.B., Faye,M., Fall,G., Chitty,J.A., Diagne,M.M., Wiley,M.R., Yinka-Ogunleye,A.F., Aruna,S., Etebu,E.N., Aworabhi,N., Ogoina,D., Numbere,W., Mba,N., Palacios,G., Sall,A.A. and Ihekweazu,C.
EPI_ISL_13058465, EPI_ISL_13058466, EPI_ISL_13058467, EPI_ISL_13058468, EPI_ISL_13058469, EPI_ISL_13058470, EPI_ISL_13058471, EPI_ISL_13058472, EPI_ISL_13058473, EPI_ISL_13058474	Virology, Centre International de Recherches Medicales de Franceville	Virology, Centre International de Recherches Medicales de Franceville	Selekon,B., Labouba,I.L., Gonofio,E.C., Sem Ouilibona,R., Simo Tchetgna,H., Besombes,C., Feher,M., Fontanet,A., Kazanji,M., Manugerra,J.-C., Gessain,A., Nakoune,E. and Berthet,N.
EPI_ISL_13058475	National Public Health Laboratory, National Centre for Infectious Diseases	National Public Health Laboratory, National Centre for Infectious Diseases	Yong,S.E.F., Ng,O.T., Ho,Z.J.M., Mak,T.M., Marimuthu,K., Vasoo,S., Yeo,T.W., Ng,Y.K., Cui,L., Ferdous,Z., Chia,P.Y., Aw,B.J.W., Manauis,C.M., Low,C.K.K., Chan,G., Peh,X., Lim,P.L., Chow,L.P.A., Chan,M., Lee,V.J.M., Lin,R.T.P., Heng,M.K.D. and Leo,Y.S.
EPI_ISL_13069002	Erasmus Medical Center Department of Virology	Erasmus Medical Center Department of Virology	Bas Oude Munnink, Marjan Boter, Babette Weller, Richard Molenkamp, Janette Rahamat-Langendoen, Reina Sikkema, Marion Koopmans
EPI_ISL_13089461	Hospital General Universitario Gregorio Marañón	Hospital General Universitario Gregorio Marañón	Sergio Buenestado Serrano, Rosalía Palomino Cabrera, Daniel Peñas Utrilla, Jorge Rodríguez-Grande, Laura Pérez-Lago, Cristina Rodriguez-Grande, Marta Herranz Martin, Julia Suárez, Pilar Catalán, Patricia Muñoz, Darío García de Viedma
EPI_ISL_13090993	Virology, Instituto Nacional de Enfermedades Infecciosas	Virology, Instituto Nacional de Enfermedades Infecciosas	Lewis,A., Josiowicz,A., Bonaventura,R., Basiletti,J., Hirmas Riade,S.M., Tous,M. and Cisterna,D.M.
EPI_ISL_13094227	Centers for Disease Control & Prevention (CDC), Division of High Consequence Pathogens and Pathology (DHCPP-PRB)	Centers for Disease Control & Prevention (CDC), Division of High Consequence Pathogens and Pathology (DHCPP-PRB)	Gigante,C.M., Lee,P., Seabolt,M.H., Wilkins,K., McCollum,A., Hutson,C., Davidson,W., Rao,A., Mendoza,R. and Li,Y.
EPI_ISL_13096615	Centers for Disease Control & Prevention (CDC), Division of High Consequence Pathogens and Pathology (DHCPP-PRB)	Centers for Disease Control & Prevention (CDC), Division of High Consequence Pathogens and Pathology (DHCPP-PRB)	Gigante,C.M., Griffin-Thomas,L.A., Seabolt,M.H., Wilkins,K., McCollum,A., Hutson,C., Davidson,W., Rao,A., Crain,J. and Li,Y.
EPI_ISL_13100618	Centers for Disease Control & Prevention (CDC), Division of High Consequence Pathogens and Pathology (DHCPP-PRB)	Centers for Disease Control & Prevention (CDC), Division of High Consequence Pathogens and Pathology (DHCPP-PRB)	Gigante,C.M., Ventura,J., Seabolt,M.H., Wilkins,K., McCollum,A., Hutson,C., Davidson,W., Rao,A., Nash,J. and Li,Y.
EPI_ISL_13100619	Centers for Disease Control & Prevention (CDC), Division of High Consequence Pathogens and Pathology (DHCPP-PRB)	Centers for Disease Control & Prevention (CDC), Division of High Consequence Pathogens and Pathology (DHCPP-PRB)	Gigante,C.M., Lee,P., Seabolt,M.H., Wilkins,K., McCollum,A., Hutson,C., Davidson,W., Rao,A., Mendoza,R. and Li,Y.
EPI_ISL_13100620	Centers for Disease Control & Prevention (CDC), Division of High Consequence Pathogens and Pathology (DHCPP-PRB)	Centers for Disease Control & Prevention (CDC), Division of High Consequence Pathogens and Pathology (DHCPP-PRB)	Gigante,C.M., Atkinson,A., Seabolt,M.H., Wilkins,K., McCollum,A., Hutson,C., Davidson,W., Rao,A., Murray,J. and Li,Y.
EPI_ISL_13100621	Centers for Disease Control & Prevention (CDC), Division of High Consequence Pathogens and Pathology (DHCPP-PRB)	Centers for Disease Control & Prevention (CDC), Division of High Consequence Pathogens and Pathology (DHCPP-PRB)	Gigante,C.M., Stringer,J., Seabolt,M.H., Wilkins,K., McCollum,A., Hutson,C., Davidson,W., Rao,A., Schulte,J. and Li,Y.
EPI_ISL_13100622	Centers for Disease Control & Prevention (CDC), Division of High Consequence Pathogens and Pathology (DHCPP-PRB)	Centers for Disease Control & Prevention (CDC), Division of High Consequence Pathogens and Pathology (DHCPP-PRB)	Gigante,C.M., Myers,R., Seabolt,M.H., Wilkins,K., McCollum,A., Hutson,C., Davidson,W., Rao,A., Blythe,D. and Li,Y.
EPI_ISL_13100719	Centers for Disease Control & Prevention (CDC), Division of High Consequence Pathogens and Pathology (DHCPP-PRB)	Centers for Disease Control & Prevention (CDC), Division of High Consequence Pathogens and Pathology (DHCPP-PRB)	Gigante,C.M., Ventura,J., Seabolt,M.H., Wilkins,K., McCollum,A., Hutson,C., Davidson,W., Rao,A., Nash,J. and Li,Y.
EPI_ISL_13106454	Hospital General Universitario Gregorio Marañón	Hospital General Universitario Gregorio Marañón	Sergio Buenestado Serrano, Rosalía Palomino Cabrera, Daniel Peñas Utrilla, Jorge Rodríguez-Grande, Pedro Sola Campoy, Laura Pérez-Lago, Cristina Rodriguez-Grande, Marta Herranz Martin, Julia Suárez, Pilar Catalán, Patricia Muñoz, Darío García de Viedma
EPI_ISL_13117291	Centre for Biological Threats, Highly Pathogenic Viruses, Robert Koch Institute	Centre for Biological Threats, Highly Pathogenic Viruses, Robert Koch Institute	Brinkmann,A., Kohl,C., Uddin,S., Pape,K., Schrick,L., Michel,J., Jessen,H., Schaade,L. and Michel,A.
EPI_ISL_13117292, EPI_ISL_13117293	Centre for Biological Threats, Highly Pathogenic Viruses, Robert Koch Institute	Centre for Biological Threats, Highly Pathogenic Viruses, Robert Koch Institute	Brinkmann,A., Kohl,C., Uddin,S., Pape,K., Schrick,L., Michel,J., Stocker,H., Schaade,L. and Nitsche,A.
EPI_ISL_13117294, EPI_ISL_13117295, EPI_ISL_13117296, EPI_ISL_13117297, EPI_ISL_13117298	Centre for Biological Threats, Highly Pathogenic Viruses, Robert Koch Institute	Centre for Biological Threats, Highly Pathogenic Viruses, Robert Koch Institute	Brinkmann,A., Kohl,C., Uddin,S., Pape,K., Schrick,L., Michel,J., Schaade,L. and Nitsche,A.
EPI_ISL_13148263, EPI_ISL_13148264, EPI_ISL_13148265	Centre for Biological Threats, Highly Pathogenic Viruses, Robert Koch Institute	Centre for Biological Threats, Highly Pathogenic Viruses, Robert Koch Institute	Brinkmann,A., Kohl,C., Uddin,S., Pape,K., Schrick,L., Michel,J., Jessen,H., Schaade,L. and Nitsche,A.
EPI_ISL_13148266, EPI_ISL_13148267, EPI_ISL_13148268, EPI_ISL_13148269	Centre for Biological Threats, Highly Pathogenic Viruses, Robert Koch Institute	Centre for Biological Threats, Highly Pathogenic Viruses, Robert Koch Institute	Brinkmann,A., Kohl,C., Uddin,S., Pape,K., Schrick,L., Michel,J., Stocker,H., Schaade,L. and Nitsche,A.
EPI_ISL_13148270, EPI_ISL_13148271, EPI_ISL_13148272, EPI_ISL_13148273, EPI_ISL_13148274, EPI_ISL_13148275, EPI_ISL_13148276	Centre for Biological Threats, Highly Pathogenic Viruses, Robert Koch Institute	Centre for Biological Threats, Highly Pathogenic Viruses, Robert Koch Institute	Brinkmann,A., Kohl,C., Uddin,S., Pape,K., Schrick,L., Michel,J., Schaade,L. and Nitsche,A.
EPI_ISL_13157812	Laboratorio di Microbiologia e Virologia, Università Vita-Salute San Raffaele, Milano	Laboratorio di Microbiologia e Virologia, Università Vita-Salute San Raffaele, Milano	Benedetta Giuliani, Sofia Sisti, Michela Sampaolo, Elena Criscuolo, Matteo Castelli, Roberto Ferrarese, Roberta Antonia Diotti, Massimo Locatelli, Massimo Clementi, Nicasio Mancini, Nicola Clementi
EPI_ISL_13158444	Laboratorio di Microbiologia e Virologia, Università Vita-Salute San Raffaele, Milano	Laboratorio di Microbiologia e Virologia, Università Vita-Salute San Raffaele, Milano	Sofia Sisti, Michela Sampaolo, Elena Criscuolo, Benedetta Giuliani, Matteo Castelli, Roberto Ferrarese, Martina Libera, Massimo Locatelli, Massimo Clementi, Nicasio Mancini, Nicola Clementi
EPI_ISL_13159759	Laboratorio di Microbiologia e Virologia, Università Vita-Salute San Raffaele, Milano	Laboratorio di Microbiologia e Virologia, Università Vita-Salute San Raffaele, Milano	Roberto Ferrarese, Benedetta Giuliani, Elena Criscuolo, Sofia Sisti, Michela Sampaolo, Matteo Castelli, Roberta Antonia Diotti, Massimo Locatelli, Massimo Clementi, Nicasio Mancini, Nicola Clementi
EPI_ISL_13191424	Virology, INEI-ANLIS Dr. Carlos G. Malbran,	Virology, INEI-ANLIS Dr. Carlos G. Malbran,	Lewis,A., Josiowicz,A., Hirmas Riade,S.M., Tous,M. and Cisterna,D.M.
EPI_ISL_13191438	Instituto de Infectologia Emilio Ribas	Instituto Adolfo Lutz Strategic Laboratory	Claudio Tavares Sacchi, Karoline Rodrigues Campos, Marlon Benedito Nascimento Santos, Alex Domingos Reis, Ariadne Ferreira Amarante, Adriano Abbud, Adriana Bugno, Walkiria Delnoro Almeida Prado, Regiane Cardoso de Paula
EPI_ISL_13194516	Alberta Precision Laboratories	Alberta Precision Laboratories	Matthew Croxen, Ashwin Deo, Paul Dieu, Xiaoli Dong, Kara Gill, David Granger, Christina Ferrato, Vanipriyadarsini Ikkurti, Jamil Kanji, Petya Koleva, Vincent Li, Colin Lloyd, Tarah Lynch, Raymond Ma, Kanti Pabbaraju, Silas Rotich, Hilary Sergeant, Steven Shideler, Todd Skitsko, Sandy Shokopoles, Graham Tipples, Johanna Thayer, Anita Wong
EPI_ISL_13234112	Laboratório Central de Saúde Pública do Estado do Rio Grande do Sul	Instituto Adolfo Lutz Strategic Laboratory	Claudio Tavares Sacchi, Karoline Rodrigues Campos, Adriano Abbud, Adriana Bugno
EPI_ISL_13242738	Hospital General Universitario Gregorio Marañón	Hospital General Universitario Gregorio Marañón	Sergio Buenestado Serrano, Rosalía Palomino Cabrera, Daniel Peñas Utrilla, Jorge Rodríguez-Grande, Pedro Sola Campoy, Laura Pérez-Lago, Cristina Rodriguez-Grande, Marta Herranz Martin, Julia Suárez, Pilar Catalán, Patricia Muñoz, Darío García de Viedma
EPI_ISL_13244349, EPI_ISL_13244610	Erasmus Medical Center Department of Virology	Erasmus Medical Center Department of Virology	Bas Oude Munnink, Marjan Boter, Babette Weller, Richard Molenkamp, Janette Rahamat-Langendoen, Reina Sikkema, Marion Koopmans
EPI_ISL_13251120	Laboratory of Virology, INMI Lazzaro Spallanzani IRCCS	Laboratory of Virology, INMI Lazzaro Spallanzani IRCCS	Giombini,E., Gruber,C.E.M., Rueca,M., Gramigna,G., VIta,S., Carletti,F., D'Abramo,A., Lapa,D., Puro,V., Fabeni,L., Butera,O., Colavita,F., Meschi,S., Matusali,G., Specchiarello,E., Vairo,F., Vaia,F., Garbuglia,A.R., Nicastri,E., Antinori,A., GIrardi,E. and Maggi,F.
EPI_ISL_13251157	checkin Zollhaus	Institute of Medical Virology, University of Zurich	Verena Kufner, Gabriela Ziltener, Maryam Zaheri, Stefan Schmutz, Annette Audigé, Odette Bernasconi, Kevin Steiner, Jon Huder, Cyril Shah, Riccarda Capaul, Guido Bloemberg, Jürg Böni, Michael Huber, Alexandra Trkola
EPI_ISL_13251584	Division of Infectious Diseases, University Hospital Zürich	Institute of Medical Virology, University of Zurich	Verena Kufner, Gabriela Ziltener, Maryam Zaheri, Stefan Schmutz, Annette Audigé, Odette Bernasconi, Kevin Steiner, Jon Huder, Cyril Shah, Riccarda Capaul, Guido Bloemberg, Jürg Böni, Michael Huber, Alexandra Trkola
EPI_ISL_13251723	checkin Zollhaus	Institute of Medical Virology, University of Zurich	Verena Kufner, Gabriela Ziltener, Maryam Zaheri, Stefan Schmutz, Annette Audigé, Odette Bernasconi, Kevin Steiner, Jon Huder, Cyril Shah, Riccarda Capaul, Guido Bloemberg, Jürg Böni, Michael Huber, Alexandra Trkola
EPI_ISL_13269478	Alberta Precision Laboratories	Alberta Precision Laboratories	Matthew Croxen, Ashwin Deo, Paul Dieu, Xiaoli Dong, Kara Gill, David Granger, Christina Ferrato, Vanipriyadarsini Ikkurti, Jamil Kanji, Petya Koleva, Vincent Li, Colin Lloyd, Tarah Lynch, Raymond Ma, Kanti Pabbaraju, Silas Rotich, Hilary Sergeant, Steven Shideler, Todd Skitsko, Sandy Shokopoles, Graham Tipples, Johanna Thayer, Anita Wong
EPI_ISL_13270980	Instituto de Infectologia Emilio Ribas	Imperial College London, School of Public Health	Claro,I.M., de Lima,E.L., Romano,C.M., Candido,D.S., Lindoso,J.A.L., Barra,L.A.C., Borges,L.M.S., Medeiros,L.A., Tomishige,M.Y.S., Ramundo,M.S., Moutinho,T., da Silva,A.J.D., Rodrigues,C.C.M., de Azevedo,L.C.F., Villas-Boas,L.S., da Silva,C.A.M., Coletti,T.M., O'Toole,A., Quick,J., Loman,N., Rambaut,A., Faria,N.R., Figueiredo-Mello,C. and Sabino,E.C.
EPI_ISL_13302316	Laboratory of Clinical Microbiology, Virology and Bioemergencies. ASST-Fatebenefratelli-Sacco, L.Sacco	Army Medical and Veterinary Research Center	Silvia Fillo, Riccardo De Sanctis, Giovanni Faggioni, Andrea Ciammaruconi, Anna Anselmo, Vanessa Vera Fain, Simone Di Sabatino, Francesco Giordani, Antonella Fortunato, Rossella Brandi, Giulia Campoli, Marzia Cavalli, Anella Monte, Martina Lipari, Maria Di Spirito, Giorgia Grilli, Silvia Chimienti, Giandomenico Cerreto, Filippo Molinari, Giancarlo Petralito, Davide Mileto, Valeria Micheli, Maria Rita Gismondo, Florigio Lista

	University Hospital		
EPI_ISL_13304977	National Public Health Center, National Biosafety Laboratory	National Public Health Center, National Biosafety Laboratory	Judit Henczkó, Dániel Déri, Lili Jármi, Bernadett Pályi, Zoltán Kis,
EPI_ISL_13308117, EPI_ISL_13308118, EPI_ISL_13308119, EPI_ISL_13308121, EPI_ISL_13308122, EPI_ISL_13308124, EPI_ISL_13308125, EPI_ISL_13308127, EPI_ISL_13308129, EPI_ISL_13308131, EPI_ISL_13308133, EPI_ISL_13308135, EPI_ISL_13308137, EPI_ISL_13308139, EPI_ISL_13308140, EPI_ISL_13308142, EPI_ISL_13308144, EPI_ISL_13308145, EPI_ISL_13308146, EPI_ISL_13308147, EPI_ISL_13308148, EPI_ISL_13308150, EPI_ISL_13308151, EPI_ISL_13308153, EPI_ISL_13308155, EPI_ISL_13308157			
see above	Centre for Biological Threats, Highly Pathogenic Viruses, Robert Koch Institute	Centre for Biological Threats, Highly Pathogenic Viruses, Robert Koch Institute	Brinkmann,A., Kohl,C., Uddin,S., Pape,K., Schrick,L., Michel,J., Schaade,L. and Nitsche,A.
EPI_ISL_13308158, EPI_ISL_13308160	IRBA Research Institute Biomédicale Des Armées	IRBA Research Institute Biomédicale Des Armées	Jarjaval,F., Nolent,F., Criqui,A., Chapus,C., Lamer,O., Ferraris,O. and Gorge,O.
EPI_ISL_13308162, EPI_ISL_13308163, EPI_ISL_13308165, EPI_ISL_13308167	Laboratory for Diagnostics of Zoonoses and WHO Centre, Institute of Microbiology and Immunology, Faculty of Medicine, University of Ljubljana	Laboratory for Diagnostics of Zoonoses and WHO Centre, Institute of Microbiology and Immunology, Faculty of Medicine, University of Ljubljana	Zakotnik,S., Vlaj,D., Suljic,A., Zorec,T.M., Korva,M., Poljak,M. and Avsic Zupanc,T.
EPI_ISL_13314740	Laboratorio de Vigilancia em Saude de Vinhedo	Instituto Adolfo Lutz Strategic Laboratory	Claudio Tavares Sacchi, Karoline Rodrigues Campos, Adriano Abbud, Adriana Bugno
EPI_ISL_13331598	Department for Virology, Molecular Biology and Genome Research, R. G. Lugar Center for Public Health Research, National Center for Disease Control and Public Health (NCDC) of Georgia	Department for Virology, Molecular Biology and Genome Research, R. G. Lugar Center for Public Health Research, National Center for Disease Control and Public Health (NCDC) of Georgia	Giorgi Tomashvili, Salome Javashvili, Meri Pantsulaia, Gvantsa Brachveli, Ana Papkiauri, Giorgi Gogoladze, Gvantsa Chanturia, Adam Kotorashvili, Maia Alkhazashvili, Khatuna Zakhashvili, Paata Imnadze, Amiran Gamkrelidze.
EPI_ISL_13331712	Laboratory of Virology, INMI Lazzaro Spallanzani IRCCS	Laboratory of Virology, INMI Lazzaro Spallanzani IRCCS	Rueca,M., Giombini,E., Gruber,C.E.M., Gramigna,G., Mazzotta,V., Carletti,F., Lapa,D., Pittalis,S., Puro,V., Fabeni,L., Butera,O., Colavita,F., Meschi,S., Matusali,G., Specchiarello,E., Vairo,F., Vaia,F., Nicastri,E., Antinori,A., Girardi,E. and Maggi,F.
EPI_ISL_13331713	Laboratory of Virology, INMI Lazzaro Spallanzani IRCCS	Laboratory of Virology, INMI Lazzaro Spallanzani IRCCS	Gramigna,G., Giombini,E., Gruber,C.E.M., Rueca,M., Carletti,F., Cicalini,S., Lapa,D., Puro,V., Marani,A., Fabeni,L., Butera,O., Colavita,F., Meschi,S., Matusali,G., Rivano Capparuccia,M., Specchiarello,E., Vairo,F., Vaia,F., Nicastri,E., Antinori,A., Girardi,E. and Maggi,F.
EPI_ISL_13331714	Department of Virology, Faculty of Medicine, University of Helsinki, Hartmaninkatu 3	Department of Virology, Faculty of Medicine, University of Helsinki, Hartmaninkatu 3	Kant,R., Smura,T., Vauhkonen,H. and Vapalahti,O.
EPI_ISL_13331715	Department of Virology, Faculty of Medicine, University of Helsinki, Hartmaninkatu 3	Department of Virology, Faculty of Medicine, University of Helsinki, Hartmaninkatu 3	Kant,R., Smura,T., Vauhkonen,H., Vapalahti,O. and Sironen,T.
EPI_ISL_13331716	Genomics Division, Instituto Tecnologico y de Energias Renovables (ITER), Poligono Industrial de Granadilla	Genomics Division, Instituto Tecnologico y de Energias Renovables (ITER), Poligono Industrial de Granadilla	Alcoba-Florez,J., Munoz-Barrera,A., Ciuffreda,L., Rodriguez-Perez,H., Rubio-Rodriguez,L.A., Gil-Campesino,H., Garcia-Martinez de Artola,D., Inigo-Campos,A., Diez-Gil,O., Gonzalez-Montelongo,R., Valenzuela-Fernandez,A., Lorenzo-Salazar,J.M. and Flores,C.
EPI_ISL_13331717	Genomics Division, Instituto Tecnologico y de Energias Renovables (ITER), Poligono Industrial de Granadilla	Genomics Division, Instituto Tecnologico y de Energias Renovables (ITER), Poligono Industrial de Granadilla,	Alcoba-Florez,J., Munoz-Barrera,A., Ciuffreda,L., Rodriguez-Perez,H., Rubio-Rodriguez,L.A., Gil-Campesino,H., Garcia-Martinez de Artola,D., Inigo-Campos,A., Diez-Gil,O., Gonzalez-Montelongo,R., Valenzuela-Fernandez,A., Lorenzo-Salazar,J.M. and Flores,C.
EPI_ISL_13338028	Clinical Virology Unit, Department of Clinical Sciences, Institute of Tropical Medicine of Antwerp	Clinical Virology Unit, Department of Clinical Sciences, Institute of Tropical Medicine of Antwerp	Antonio Mauro Rezende*, Tessa de Block*, Sandra Coppens, Eric Florence, Maartje van Frankenhuijsen, Stefanie Bracke, Isabel Brosius, Laurens Liesenborghs, Patrick Soentjens, Kevin Ariën, Marjan Van Esbroeck, Philippe Selhorst*, Koen Vercauteren* *equal contribution
EPI_ISL_13339105	Microbiology Service, Hospital Universitario Clinico San Cecilio, Granada	Microbiology Service, Hospital Universitario Clinico San Cecilio, Granada	Chueca N, de Salazar A, Viñuela L, Fuentes A, Casimiro-Soriguer CS, Perez-Florido J, Dopazo J, Garcia F
EPI_ISL_13342823	Clinical Virology Unit, Department of Clinical Sciences, Institute of Tropical Medicine of Antwerp	Clinical Virology Unit, Department of Clinical Sciences, Institute of Tropical Medicine of Antwerp	Philippe Selhorst, Antonio Mauro Rezende, Tessa de Block, Sandra Coppens, Eric Florence, Isabel Brosius, Laurens Liesenborghs, Kevin Ariën, Marjan Van Esbroeck, Chris Kenyon, Koen Vercauteren
EPI_ISL_13343634	Instituto de Infectologia Emilio Ribas	Instituto Adolfo Lutz Strategic Laboratory	Claudio Tavares Sacchi, Karoline Rodrigues Campos, Adriano Abbud, Adriana Bugno
EPI_ISL_13343697	Fleury Medicina Dignóstica	Instituto Adolfo Lutz Strategic Laboratory	Claudio Tavares Sacchi, Karoline Rodrigues Campos, Adriano Abbud, Adriana Bugno
EPI_ISL_13343718	Hospital Santa Ignes	Instituto Adolfo Lutz Strategic Laboratory	Claudio Tavares Sacchi, Karoline Rodrigues Campos, Adriano Abbud, Adriana Bugno
EPI_ISL_13351002	B.C. Centre for Disease Control Public Health Laboratory	B.C. Centre for Disease Control Public Health Laboratory	John Tyson, Tracy Lee, Anthea Lam, Josh Quick, Agatha Jassem, Natalie Prystajecky, Linda Hoang, Inna Sekirov, Catherine Hogn, Frankie Tsang, Mel Krajden
EPI_ISL_13362760, EPI_ISL_13362764	Laboratorio di Epidemiologia Molecolare e Sanità Pubblica-Policlinico Bari	Istituto Zooprofilattico Sperimentale della Puglia e della Basilicata	Parisi A, Simone D, Capozzi L, Del Sambro L, Bianco A, Chironna M, Loconsole D, Sallustio F, Galante D, Pace L, Manzulli V, Fasanella A.
EPI_ISL_13363142	Hospital Universitari Vall d'Hebron	Hospital Universitari Vall d'Hebron	Maria Piñana, Cristina Andrés, Alejandra González-Sánchez, Damir Garcia-Cehic, Ariadna Rando, Juliana Esperalba, Maria Gema Codina, Maria Carmen Martin, Carla Castillo, Karen García, Rodrigo Vásquez, Maria Piquer, Tomàs Pumarola, Josep Quer, Andrés Antón
EPI_ISL_13374487	National Public Health Center, National Biosafety Laboratory	National Public Health Center, National Biosafety Laboratory	Judit Henczkó, Dániel Déri, Fruzsina Petrovay, Lili Jármi, Bernadett Pályi, Eszter Balla, Zoltán Kis
EPI_ISL_13408797	Virology, Instituto Nacional de Enfermedades Infecciosas	Virology, Instituto Nacional de Enfermedades Infecciosas	Lewis,A., Josiowicz,A., Hirmas Riade,S.M., Tous,M. and Cisterna,D.M.
EPI_ISL_13408799, EPI_ISL_13408801, EPI_ISL_13408803	Public Health Agency of Canada, National Microbiology Laboratory	Public Health Agency of Canada, National Microbiology Laboratory	Knox,N., Hole,D., Duggan,A., Yadav,C., Haidl,E., Chapel,M., Graham,M., Domselaar,G.V., Jolly,G., Audet,J., Fernando,L., Antonation,K., Hagan,M., Griffiths,E., Leung,A., Safronetz,D., Eshaghi,A., Gubbay,J.B., Hasso,M., Marchand-Austin,A., Olsha,R. and Patel,S.N.
EPI_ISL_13408805, EPI_ISL_13408807, EPI_ISL_13408809, EPI_ISL_13408811, EPI_ISL_13408813, EPI_ISL_13408815, EPI_ISL_13408817, EPI_ISL_13408819, EPI_ISL_13408821, EPI_ISL_13408823, EPI_ISL_13408825, EPI_ISL_13408827, EPI_ISL_13408829, EPI_ISL_13408831, EPI_ISL_13408833, EPI_ISL_13408835			
see above	Public Health Agency of Canada, National Microbiology Laboratory	Public Health Agency of Canada, National Microbiology Laboratory	ncknox
EPI_ISL_13408837, EPI_ISL_13408839, EPI_ISL_13408841, EPI_ISL_13408843, EPI_ISL_13408845, EPI_ISL_13408847, EPI_ISL_13408849, EPI_ISL_13408851, EPI_ISL_13408853, EPI_ISL_13408855, EPI_ISL_13408857, EPI_ISL_13408859, EPI_ISL_13408861			
see above	Public Health Agency of Canada, National Microbiology Laboratory	Public Health Agency of Canada, National Microbiology Laboratory	Knox,N., Duggan,A., Yadav,C., Hole,D., Haidl,E., Chapel,M., Jolly,G., Domselaar,G.V., Antonation,K., Leung,A., Fernando,L., Audet,J., Hagan,M., Graham,M., Griffiths,E., Safronetz,D., Charest,H., Levade,I. and Fafard,J.
EPI_ISL_13409177, EPI_ISL_13409178, EPI_ISL_13409179, EPI_ISL_13409180, EPI_ISL_13409181	Viral Genomics and Bioinformatics, MRC University of Glasgow Centre for Virus Research	Viral Genomics and Bioinformatics, MRC University of Glasgow Centre for Virus Research	Filipe,A., Tong,L., Vattipally,S.B., Maclean,A., Gunson,R., Holden,M.T.G., Barr,D., Ho,A., Palmarini,M., Rambaut,A., Robertson,D.L. and Thomson,E.C.
EPI_ISL_13411153, EPI_ISL_13411154, EPI_ISL_13411155, EPI_ISL_13411156, EPI_ISL_13411157, EPI_ISL_13411158	Centre for Biological Threats, Highly Pathogenic Viruses, Robert Koch Institute	Centre for Biological Threats, Highly Pathogenic Viruses, Robert Koch Institute	Brinkmann,A., Kohl,C., Uddin,S., Pape,K., Schrick,L., Michel,J., Schaade,L. and Nitsche,A.
EPI_ISL_13411159, EPI_ISL_13411160, EPI_ISL_13411161, EPI_ISL_13411162	Centre for Biological Threats, Highly Pathogenic Viruses, Robert Koch Institute	Centre for Biological Threats, Highly Pathogenic Viruses, Robert Koch Institute	Brinkmann,A., Kohl,C., Uddin,S., Pape,K., Schrick,L., Michel,J., Stocker,H., Schaade,L. and Nitsche,A.
EPI_ISL_13411163, EPI_ISL_13411164, EPI_ISL_13411165	Centre for Biological Threats, Highly Pathogenic Viruses, Robert Koch Institute	Centre for Biological Threats, Highly Pathogenic Viruses, Robert Koch Institute	Brinkmann,A., Kohl,C., Uddin,S., Pape,K., Schrick,L., Michel,J., Schaade,L. and Nitsche,A.
EPI_ISL_13411166, EPI_ISL_13411167	Centre for Biological Threats, Highly Pathogenic Viruses, Robert Koch Institute	Centre for Biological Threats, Highly Pathogenic Viruses, Robert Koch Institute	Brinkmann,A., Kohl,C., Uddin,S., Pape,K., Schrick,L., Michel,J., Jessen,H., Schaade,L. and Nitsche,A.
EPI_ISL_13411168	Centre for Biological Threats, Highly Pathogenic Viruses, Robert Koch Institute	Centre for Biological Threats, Highly Pathogenic Viruses, Robert Koch Institute	Brinkmann,A., Kohl,C., Uddin,S., Pape,K., Schrick,L., Michel,J., Pfaefflin,F., Schaade,L. and Nitsche,A.
EPI_ISL_13436658	Coordenadoria de Vigilancia em Saude - Sao Paulo	Instituto Adolfo Lutz Strategic Laboratory	Claudio Tavares Sacchi, Karoline Rodrigues Campos, Ariadne Fereira Amarante, Adriano Abbud, Adriana Bugno
EPI_ISL_13436792	Hospital Santa Ignes	Instituto Adolfo Lutz Strategic Laboratory	Claudio Tavares Sacchi, Karoline Rodrigues Campos, Adriano Abbud, Adriana Bugno
EPI_ISL_13437056	Hosp. Alemao Oswaldo Cruz	Instituto Adolfo Lutz Strategic Laboratory	Claudio Tavares Sacchi, Karoline Rodrigues Campos, Ariadne Ferreira Amarante, Adriano Abbud, Adriana Bugno
EPI_ISL_13445553	Laboratory for Diagnostics of Zoonoses and WHO Centre, Institute of Microbiology and Immunology, Faculty of Medicine, University of Ljubljana	Laboratory for Diagnostics of Zoonoses and WHO Centre, Institute of Microbiology and Immunology, Faculty of Medicine, University of Ljubljana	Zakotnik,S., Vlaj,D., Suljic,A., Zorec,T.M., Korva,M., Poljak,M. and Avsic Zupanc,T.
EPI_ISL_13449965, EPI_ISL_13449966	Hospital Universitario La Paz, Microbiology	Hospital Universitario La Paz, Microbiology	de la Hoz-Sanchez,B., Lopez-Ortiz,M., Gutierrez-Arroyo,A., Roces-Alvarez,P., Lazaro-Peona,F., Dahdouh,E., Bloise,I., Garcia-Rodriguez,J. and Mingorance,J.
EPI_ISL_13459346	CRT-DST-AIDS	Instituto Adolfo Lutz Strategic Laboratory	Claudio Tavares Sacchi, Karoline Rodrigues Campos, Ariadne Ferreira Amarante, Adriano Abbud, Adriana Bugno
EPI_ISL_13459347, EPI_ISL_13459482, EPI_ISL_13459483	Instituto de Infectologia Emilio Ribas	Instituto Adolfo Lutz Strategic Laboratory	Claudio Tavares Sacchi, Karoline Rodrigues Campos, Ariadne Ferreira Amarante, Adriano Abbud, Adriana Bugno
EPI_ISL_13466447, EPI_ISL_13466448, EPI_ISL_13466449, EPI_ISL_13466450, EPI_ISL_13466451, EPI_ISL_13466452, EPI_ISL_13466453, EPI_ISL_13466455, EPI_ISL_13466456, EPI_ISL_13466457, EPI_ISL_13466458, EPI_ISL_13466459, EPI_ISL_13466460, EPI_ISL_13466461, EPI_ISL_13466462, EPI_ISL_13466463, EPI_ISL_13466464, EPI_ISL_13466465			
see above	Department of Infectious Diseases, National Institute of Health Doutor Ricardo Jorge, Portugal (INSA)	Department of Infectious Diseases, National Institute of Health Doutor Ricardo Jorge, Portugal (INSA)	Isidro,J., Borges,V., Pinto,M., Sobral,D., Santos,J., Nunes,A., Mixao,V., Ferreira,R., Santos,D., Duarte,S., Vieira,L., Borrego,M.J., Nuncio,S., Lopes de Carvalho,I., Pelerito,A., Cordeiro,R., Gomes,J.P.
EPI_ISL_13472080	National Institute of Public Health NIH - NRI	National Institute of Public Health NIH - NRI	Wołkowicz Tomasz, Zacharczuk Katarzyna, Gierczyński Rafał
EPI_ISL_13472250	Medical University of Vienna Center for Virology	Medical University of Vienna Center for Virology	Jeremy V. Camp, Monika Redlberger-Fritz, Stephan W. Aberle
EPI_ISL_13483155, EPI_ISL_13483157, EPI_ISL_13483159, EPI_ISL_13483161, EPI_ISL_13483162, EPI_ISL_13483163, EPI_ISL_13483164	Centre for Biological Threats, Highly Pathogenic Viruses, Robert Koch Institute	Centre for Biological Threats, Highly Pathogenic Viruses, Robert Koch Institute	Brinkmann,A., Kohl,C., Uddin,S., Pape,K., Schrick,L., Michel,J., Jessen,H., Schaade,L. and Nitsche,A.
EPI_ISL_13483165, EPI_ISL_13483167, EPI_ISL_13483168, EPI_ISL_13483170, EPI_ISL_13483171, EPI_ISL_13483173, EPI_ISL_13483175, EPI_ISL_13483177, EPI_ISL_13483178, EPI_ISL_13483180, EPI_ISL_13483182, EPI_ISL_13483183, EPI_ISL_13483185, EPI_ISL_13483187, EPI_ISL_13483188, EPI_ISL_13483190, EPI_ISL_13483191, EPI_ISL_13483193, EPI_ISL_13483195, EPI_ISL_13483196,			

EPI_ISL_13483198, EPI_ISL_13483200, EPI_ISL_13483201, EPI_ISL_13483203, EPI_ISL_13483205, EPI_ISL_13483206, EPI_ISL_13483208			
see above	Centre for Biological Threats, Highly Pathogenic Viruses, Robert Koch Institute	Centre for Biological Threats, Highly Pathogenic Viruses, Robert Koch Institute	Brinkmann,A., Kohl,C., Uddin,S., Pape,K., Schrick,L., Michel,J., Schaade,L. and Nitsche,A.
EPI_ISL_13484458	Laboratorio de Enterovirus, Instituto Oswaldo Cruz, Fiocruz	Instituto Oswaldo Cruz FIOCRUZ - Laboratory of Respiratory Viruses and Measles (LVRS)	Paola Resende, Elisa Calvalcante Pereira, Bruna Mendonça da Silva, Jéssica Graça Macedo de Carvalho, Larissa Macedo Pinto, Victor Guimaraes, Marilda Siqueira, Renan da Silva Faustino, Marilia Santini, Edson Elias da Silva on behalf of the Fiocruz Genomic Surveillance Network
EPI_ISL_13498265	National Institute for Communicable Diseases of the National Health Laboratory Service	National Institute for Communicable Diseases of the National Health Laboratory Service	Chan WY, Mtshali PS, Grobbelaar A, Moolla N, Mohale T, Du Plessis MG, Ismail A, Weyer J
EPI_ISL_13502582	Laboratory of Microbiology and Virology, Ospedale Amedeo di Savoia, ASL "Città di Torino"	Laboratory of Microbiology and Virology, Ospedale Amedeo di Savoia, ASL "Città di Torino"	Francesco Cerutti, Antonella Bottoni, Marisa Cazzadore, Tiziano Allice, Maria Grazia Milia, Gabriella Gregori, Elisa Burdino, Valeria Ghisetti
EPI_ISL_13508393	Hosp. Itacolomy Butanta	Instituto Adolfo Lutz Strategic Laboratory	Claudio Tavares Sacchi, Karoline Rodrigues Campos, Ariadne Ferreira Amarante, Adriano Abbud, Adriana Bugno
EPI_ISL_13508471	Instituto de Infectologia Emilio Ribas	Instituto Adolfo Lutz Strategic Laboratory	Claudio Tavares Sacchi, Karoline Rodrigues Campos, Ariadne Ferreira Amarante, Adriano Abbud, Adriana Bugno
EPI_ISL_13511312	Laboratorio de Salud Pública de Antioquia	Instituto Nacional de Salud- Dirección de Investigación en Salud Pública	Katherine Laiton-Donato, Diego A. Álvarez-Díaz, Carlos Franco-Muñoz, Héctor A. Ruiz-Moreno, Paola Rojas-Estevez, Andres Prada, Alicia Rosales, Marcela Mercado-Reyes
EPI_ISL_13530881	Laboratorio de Referencia Nacional de Virus Respiratorios. Centro Nacional de Salud Publica. Instituto Nacional de Salud Peru.	Laboratorio de Referencia Nacional de Virus Respiratorios. Centro Nacional de Salud Publica. Instituto Nacional de Salud Peru.	Carlos Padilla Rojas, Veronica Hurtado Vela, Iris Silva Molina, Luren Sevilla Castañeda, Victor Jimenez Vasquez, Orson Mestanza Millones, Luis Barcena Flores, Wendy Lizarraga Olivares, Alicia Nuñez Llanos, Steve Acedo Lazo, Francisco Ascue Orosco, Kelly Izarra Rojas, Princesa Medrano Alhuay, Karla Vasquez Cajachahua, Estela Huaman Angeles, Jorge Giraldo Chavez, Lilian Huarca Balbin, Lisbet Roxana Inga Angulo, Maria Sandra Villar Saavedra, Henri Bailon Calderon, Lely Solari Zerpa, Gloria Arotinco Garayar. Equipo de vigilancia genomica del Instituto Nacional de Salud.
EPI_ISL_13537922	Instituto de Medicina Tropical de Sao Paulo (IMT-USP)	School of Public Health, Imperial College London	Coletti,T.M., Ghilardi,F., khan,M.J., Claro,I.M., Valenca,I.N., Faria,N.R. and Sabino,E.C.
EPI_ISL_13537923	Microbiology, Immunology and Transplantation, KU Leuven, Rega Institute	Microbiology, Immunology and Transplantation, KU Leuven, Rega Institute	Wawina-Bokalanga,T., Vanmechelen,B., Logist,A.-S., Sinnesael,R., Ysebaert,L., Bloemen,M. and Maes,P.
EPI_ISL_13537924, EPI_ISL_13537925, EPI_ISL_13537926	Microbiology, Immunology and Transplantation, KU Leuven, Rega Institute	Microbiology, Immunology and Transplantation, KU Leuven, Rega Institute	Vanmechelen,B., Wawina-Bokalanga,T., Logist,A.-S., Sinnesael,R., Ysebaert,L., Verlinden,J., Van Holm,B., Bloemen,M. and Maes,P.
EPI_ISL_13544223, EPI_ISL_13544224, EPI_ISL_13544225, EPI_ISL_13544226, EPI_ISL_13544227, EPI_ISL_13544228, EPI_ISL_13544229, EPI_ISL_13544230, EPI_ISL_13544231, EPI_ISL_13544232, EPI_ISL_13544233, EPI_ISL_13544234, EPI_ISL_13544235			
see above	Public Health Agency of Canada, National Microbiology Laboratory	Public Health Agency of Canada, National Microbiology Laboratory	Duggan,A., Hole,D., Knox,N., Yadav,C., Haidl,E., Chapel,M., Domselaar,G.V., Jolly,G., Audet,J., Fernando,L., Antonation,K., Safronetz,D., Hagan,M., Griffiths,E., Leung,A., Graham,M., Peters,G., Go,A., Laminman,V., Kaplen,B., Eshaghi,A., Gubbay,J.B., Hasso,M., Marchand-Austin,A., Olsha,R. and Patel,S.N.
EPI_ISL_13544237, EPI_ISL_13544238, EPI_ISL_13544239, EPI_ISL_13544240, EPI_ISL_13544241, EPI_ISL_13544243, EPI_ISL_13544244, EPI_ISL_13544245, EPI_ISL_13544246, EPI_ISL_13544247, EPI_ISL_13544248, EPI_ISL_13544249, EPI_ISL_13544250, EPI_ISL_13544251, EPI_ISL_13544252, EPI_ISL_13544253, EPI_ISL_13544254, EPI_ISL_13544255, EPI_ISL_13544256, EPI_ISL_13544257, EPI_ISL_13544258, EPI_ISL_13544259, EPI_ISL_13544260, EPI_ISL_13544261, EPI_ISL_13544262, EPI_ISL_13544263, EPI_ISL_13544264, EPI_ISL_13544265, EPI_ISL_13544266, EPI_ISL_13544267			
see above	Public Health Agency of Canada, National Microbiology Laboratory	Public Health Agency of Canada, National Microbiology Laboratory	Duggan,A., Hole,D., Knox,N., Yadav,C., Haidl,E., Chapel,M., Domselaar,G.V., Fernando,L., Graham,M., Antonation,K., Audet,J., Hagan,M., Safronetz,D., Leung,A., Peters,G., Go,A., Laminman,V., Kaplen,B., Jolly,G., Charest,H., Levade,I. and Fafard,J.
EPI_ISL_13573943	Center for Virology, Medical University of Vienna	Medical University of Vienna Center for Virology	Jeremy V. Camp, Monika Redlberger-Fritz, Stephan W. Aberle
EPI_ISL_13584854, EPI_ISL_13586184	Institute for Virology, Philipps-University Marburg	Institute for Virology, Philipps-University Marburg	Eickmann, M., Lier, C., Kowalski, K., Kraft, F., Becker, S.
EPI_ISL_13607904	Servicio de Infectologia, Hospital Universitario Dr. José Eleuterio Gonzalez, Universidad Autonoma de Nuevo Leon	Centro de Investigacion e Innovacion en Virologia Medica, Departamento de Bioquimica y Medicina Molecular, Facultad de Medicina, Universidad Autonoma de Nuevo Leon	Kame A. Galan-Huerta, Manuel Paz Infanzon, Ali F. Ruiz Higareda, Laura Nuzzolo-Shihadeh, Adrian Camacho-Ortiz, Paola Bocanegra-Ibarias, Ana M. Rivas-Estilla, Daniel Zacarias-Villarreal, Luis A. Yamallel-Ortega, Maria D. Guerrero-Putz, Jorge Ocampo-Candiani
EPI_ISL_13624509	Instituto de Diagnóstico y Referencia Epidemiológicos/Jurisdicción Sanitaria Cuauhtémoc/Hospital Ángeles Roma	Instituto de Diagnóstico y Referencia Epidemiológicos/Instituto de Biotecnología UNAM	Adnan Araiza-Rodríguez, Adriana Salvador-Patiño, Alejandro Sánchez-Flores, América del Pilar Mandujano-Martínez, Blanca Taboada, Carlos Eduardo Hernández-Sánchez, Carlos F. Arias, Claudia Elena Wong-Arámbula, Daniel José Regalado-Santiago, David Esaú Fragoso-Fonseca, Elizabeth Andrade-Montiel, Fabiola Garcés-Ayala, Fernando González-Dominguez, Gabriel García-Rodríguez, Gloria Vázquez-Castro, Hugo López Gatell Ramírez, Irma López-Martínez, Jerome Verleyen, Jesús Trujillo, Jorge Ochoa, José Ernesto Ramírez-González, Karel Estrada-Guerra, Lucía Hernández-Rivas, Magaly Guadalupe Landa-Flores, Maribel González-Villa, Mireya Mederos-Michel, Nancy Martínez-Velázquez, Noé Escobar-Escamilla, Oliva López, Ricardo Cortés-Alcalá, Ricardo Grande, Verónica Jiménez-Jacinto
EPI_ISL_13632071	Center of Diagnostics and Vaccine Development, Centers for Disease Control, Taiwan	Center of Diagnostics and Vaccine Development, Centers for Disease Control, Taiwan	Jih-Hui Lin, Shu-Chun Chiu, Hsin-I, Huang, Wei-Lun Huang, Wen-Bin, Fann, Pei-Yu, Hsieh, Jyh-Yuan Yang
EPI_ISL_13632288	National Institute for Communicable Diseases of the National Health Laboratory Service	National Institute for Communicable Diseases of the National Health Laboratory Service	Chan WY, Mtshali PS, Grobbelaar A, Moolla N, Mohale T, Lowe M, Du Plessis MG, Ismail A, Weyer J
EPI_ISL_13651348, EPI_ISL_13651349, EPI_ISL_13651350	Laboratorio de Referencia Nacional de Virus Respiratorio. Centro Nacional de Salud Publica. Instituto Nacional de Salud.	Laboratorio de Referencia Nacional de Virus Respiratorio. Centro Nacional de Salud Publica. Instituto Nacional de Salud.	Carlos Padilla Rojas, Veronica Hurtado Vela, Iris Silva Molina, Luren Sevilla Castañeda, Victor Jimenez Vasquez, Orson Mestanza Millones, Luis Barcena Flores, Wendy Lizarraga Olivares, Alicia Nuñez Llanos, Steve Acedo Lazo, Francisco Ascue Orosco, Kelly Izarra Rojas, Princesa Medrano Alhuay, Karla Vasquez Cajachahua, Estela Huaman Angeles, Jorge Giraldo Chavez, Lilian Huarca Balbin, Lisbet Roxana Inga Angulo, Maria Sandra Villar Saavedra, Henri Bailon Calderon, Lely Solari Zerpa, Gloria Arotinco Garayar. Equipo de vigilancia genomica del Instituto Nacional de Salud.
EPI_ISL_13658019, EPI_ISL_13658021	Erasmus Medical Center Department of Virology	Erasmus Medical Center Department of Virology	Bas Oude Munnink, Marjan Boter, Babette Weller, Richard Molenkamp, Janette Rahamat-Langendoen, Reina Sikkema, Marion Koopmans
EPI_ISL_13660191	Hospital Center Luxembourg	Laboratoire National de Santé Microbiology	Eric Hugoson, Ines Kozar, Sibel Berger, Anke Wienecke-Baldacchino, Bas Oude Munnink, Michel Kohnen, Jean-Hugues Francois, Tamir Abdelrahman
EPI_ISL_13705358	Hosp. Alemao Oswaldo Cruz	Instituto Adolfo Lutz Strategic Laboratory	Claudio Tavares Sacchi, Karoline Rodrigues Campos, Ariadne Ferreira Amarante, Marlon Benedito Nascimento Santos, Alex Domingos Reis, Adriano Abbud, Adriana Bugno
EPI_ISL_13705407	Hosp. Sirio-Libanes	Instituto Adolfo Lutz Strategic Laboratory	Claudio Tavares Sacchi, Karoline Rodrigues Campos, Ariadne Ferreira Amarante, Marlon Benedito Nascimento Santos, Alex Domingos Reis, Adriano Abbud, Adriana Bugno
EPI_ISL_13717674	Hospital Center Luxembourg	Laboratoire National de Santé Microbiology	Eric Hugoson, Ines Kozar, Sibel Berger, Anke Wienecke-Baldacchino, Bas Oude Munnink, Michel Kohnen, Jean-Hugues Francois, Tamir Abdelrahman
EPI_ISL_13728303	Department of Medical Microbiology & Infection prevention, Amsterdam University Medical Centers location AMC	Department of Medical Microbiology & Infection prevention, Amsterdam University Medical Centers location AMC	Matthijs Welkers, Jelle Koopsen, Robin van Houdt, Marcel Jonges, Sebastien Matamoros, Sjoerd Rebers, Fokla Zorgdrager, Sylvia Bruisten, Judith den Uil, Akke Cornelissen, Janke Schinkel, Menno de Jong, Gini van Rijckevorsel and Mariken van der Lubben on behalf of the Amsterdam Regional Genomic epidemiology and Outbreak Surveillance (ARGOS) consortium
EPI_ISL_13732932	Hosp. Sao Joaquim - Beneficiencia Portuguesa	Instituto Adolfo Lutz Strategic Laboratory	Claudio Tavares Sacchi, Karoline Rodrigues Campos, Ariadne Ferreira Amarante, Marlon Benedito Nascimento Santos, Alex Domingos Reis, Adriano Abbud, Adriana Bugno
EPI_ISL_13734230, EPI_ISL_13734231, EPI_ISL_13734232	UK Health Security Agency	UK Health Security Agency	Atkinson,B., Pottage,T., Ngabo,D., Crook,A., Pitman,J., Summers,S., Pullan,S., Lewandowski,K., Furneaux,J., Davies,K. and Brooks,T.
EPI_ISL_13734233	Microbial Genomics, Hospital General Universitario Gregorio Maranon	Microbial Genomics, Hospital General Universitario Gregorio Maranon	Palomino-Cabrera,R., Penas-Utrilla,D., Buenestado-Serrano,S., Perez-Lago,L., Herranz Martin,M., Veintimilla,C., Catalan,P., Munoz,P. and Garcia de Viedma,D.
EPI_ISL_13734237, EPI_ISL_13734238, EPI_ISL_13734239, EPI_ISL_13734240, EPI_ISL_13734241, EPI_ISL_13734242, EPI_ISL_13734243, EPI_ISL_13734244, EPI_ISL_13734245, EPI_ISL_13734246, EPI_ISL_13734247, EPI_ISL_13734248, EPI_ISL_13734249, EPI_ISL_13734250, EPI_ISL_13734251, EPI_ISL_13734252, EPI_ISL_13734253, EPI_ISL_13734254, EPI_ISL_13734255, EPI_ISL_13734256, EPI_ISL_13734257, EPI_ISL_13734258, EPI_ISL_13734259, EPI_ISL_13734260, EPI_ISL_13734261, EPI_ISL_13734262, EPI_ISL_13734263, EPI_ISL_13734264, EPI_ISL_13734265, EPI_ISL_13734266, EPI_ISL_13734267, EPI_ISL_13734268			
see above	Centre for Biological Threats, Highly Pathogenic Viruses, Robert Koch Institute	Centre for Biological Threats, Highly Pathogenic Viruses, Robert Koch Institute	Brinkmann,A., Kohl,C., Pape,K., Uddin,S., Schrick,L., Michel,J., Schaade,L. and Nitsche,A.
EPI_ISL_13734269	Department of Clinical Sciences, Institute of Tropica Medicine	Department of Clinical Sciences, Institute of Tropica Medicine	De Baetselier,I., Van Dijck,C., Kenyon,C., Coppens,J., Smet,H., de Block,T., Coppens,S., Vanroye,F., Bugert,J., Girl,P., Liesenborghs,L., Selhorst,P., Arien,K., Van den Bossche,D., Florence,E., Rezende,A.M., Vercauteren,K. and Van Esbroeck,M.
EPI_ISL_13734270	Centers for Disease Control & Prevention (CDC), Division of High Consequence Pathogens and Pathology (DHCPP-PRB)	Centers for Disease Control & Prevention (CDC), Division of High Consequence Pathogens and Pathology (DHCPP-PRB)	Gigante,C.M., Ventura,J., Seabolt,M.H., Zhao,H., Wilkins,K., Respress,J., Howard,D., Batra,D., McCollum,A., Hutson,C., Davidson,W., Rao,A., Nash,J. and Li,Y.
EPI_ISL_13744896	Centers for Disease Control & Prevention (CDC), Division of High Consequence Pathogens and Pathology (DHCPP-PRB)	Centers for Disease Control & Prevention (CDC), Division of High Consequence Pathogens and Pathology (DHCPP-PRB)	Gigante,C.M., Ghinai,I., Seabolt,M.H., Zhao,H., Wilkins,K., Respress,J., Howard,D., Batra,D., McCollum,A., Hutson,C., Davidson,W., Rao,A., Kerins,J. and Li,Y.
EPI_ISL_13744897, EPI_ISL_13744898	Centers for Disease Control & Prevention (CDC), Division of High Consequence Pathogens and Pathology (DHCPP-PRB)	Centers for Disease Control & Prevention (CDC), Division of High Consequence Pathogens and Pathology (DHCPP-PRB)	Gigante,C.M., Hughes,S., Seabolt,M.H., Zhao,H., Wilkins,K., Respress,J., Howard,D., Batra,D., McCollum,A., Hutson,C., Davidson,W., Rao,A., Baumgartner,J. and Li,Y.
EPI_ISL_13744899	Centers for Disease Control & Prevention (CDC), Division of High Consequence Pathogens and Pathology (DHCPP-PRB)	Centers for Disease Control & Prevention (CDC), Division of High Consequence Pathogens and Pathology (DHCPP-PRB)	Gigante,C.M., Ghinai,I., Seabolt,M.H., Zhao,H., Wilkins,K., Respress,J., Howard,D., Batra,D., McCollum,A., Hutson,C., Davidson,W., Rao,A., Kerins,J. and Li,Y.
EPI_ISL_13744900, EPI_ISL_13744901	Centers for Disease Control & Prevention (CDC), Division of High Consequence Pathogens and Pathology (DHCPP-PRB)	Centers for Disease Control & Prevention (CDC), Division of High Consequence Pathogens and Pathology (DHCPP-PRB)	Gigante,C.M., Hughes,S., Seabolt,M.H., Zhao,H., Wilkins,K., Respress,J., Howard,D., Batra,D., McCollum,A., Hutson,C., Davidson,W., Rao,A., Baumgartner,J. and Li,Y.
EPI_ISL_13744902	Department of Virology, Faculty of Medicine, University of Helsinki	Department of Virology, Faculty of Medicine, University of Helsinki	Kant,R., Smura,T., Vauhkonen,H. and Vapalahti,O.
EPI_ISL_13744903, EPI_ISL_13744904, EPI_ISL_13744905	Centre for Biological Threats, Highly Pathogenic Viruses, Robert Koch Institute	Centre for Biological Threats, Highly Pathogenic Viruses, Robert Koch Institute	Brinkmann,A., Kohl,C., Pape,K., Uddin,S., Schrick,L., Michel,J., Jessen,H., Schaade,L. and Nitsche,A.
EPI_ISL_13744906, EPI_ISL_13744907, EPI_ISL_13744908, EPI_ISL_13744909, EPI_ISL_13744910, EPI_ISL_13744911, EPI_ISL_13744912, EPI_ISL_13744913, EPI_ISL_13744914, EPI_ISL_13744915, EPI_ISL_13744916, EPI_ISL_13744917, EPI_ISL_13744918, EPI_ISL_13744919, EPI_ISL_13744920, EPI_ISL_13744921, EPI_ISL_13744922, EPI_ISL_13744923, EPI_ISL_13744924, EPI_ISL_13744925, EPI_ISL_13744926, EPI_ISL_13744927, EPI_ISL_13744928, EPI_ISL_13744929, EPI_ISL_13744930, EPI_ISL_13744931			
see above	Centre for Biological Threats, Highly Pathogenic Viruses, Robert Koch Institute	Centre for Biological Threats, Highly Pathogenic Viruses, Robert Koch Institute	Brinkmann,A., Kohl,C., Pape,K., Uddin,S., Schrick,L., Michel,J., Schaade,L. and Nitsche,A.
EPI_ISL_13817808	New York University Langone Health	New York University Langone Health	Adriana Heguy, Dacia Dimartino, Emily Guzman, Christian Marier, Peter Meyn, Sitharam Ramaswami, Gael Westby, Paul Zappile, Yutong Zhang, Guiqing Wang
EPI_ISL_13822667, EPI_ISL_13822668, EPI_ISL_13822669, EPI_ISL_13822718	Erasmus Medical Center Department of Virology	Erasmus Medical Center Department of Virology	Bas Oude Munnink, Marjan Boter, Babette Weller, Richard Molenkamp, Janette Rahamat-Langendoen, Reina Sikkema, Marion Koopmans

EPI_ISL_13827273, EPI_ISL_13827274, EPI_ISL_13827275, EPI_ISL_13827277, EPI_ISL_13827278, EPI_ISL_13827279, EPI_ISL_13827280, EPI_ISL_13827281, EPI_ISL_13827282	Public Health Agency of Canada, National Microbiology Laboratory	Public Health Agency of Canada, National Microbiology Laboratory	Duggan,A., Hole,D., Yadav,C., Knox,N., Haidl,E., Chapel,M., Domselaar,G.V., Fernando,L., Graham,M., Antonation,K., Audet,J., Hagan,M., Safronetz,D., Leung,A., Peters,G., Go,A., Laminman,V., Kaplen,B., Jolly,G., Marchand-Austin,A., Eshaghi,A., Patel,S.N., Hasso,M., Gubbay,J.B. and Olsha,R.
EPI_ISL_13827283	Military Heatlh Institute in Prague, Military Heatlh Institute	Military Heatlh Institute in Prague, Military Heatlh Institute	Chmel,M., Pajer,P., Nagy,A., Zlamal,M., Jirincova,H., Dresler,J. and Bartos,O.
EPI_ISL_13833194, EPI_ISL_13833195, EPI_ISL_13833196, EPI_ISL_13833197	Laboratorio de Referencia Nacional de Virus Respiratorio. Centro Nacional de Salud Publica. Instituto Nacional de Salud.	Laboratorio de Referencia Nacional de Virus Respiratorio. Centro Nacional de Salud Publica. Instituto Nacional de Salud.	Carlos Padilla Rojas, Veronica Hurtado Vela, Iris Silva Molina, Luren Sevilla Castañeda, Victor Jimenez Vasquez, Orson Mestanza Millones, Luis Barcena Flores, Wendy Lizarraga Olivares, Alicia Nuñez Llanos, Steve Acedo Lazo, Francisco Ascue Orosco, Kelly Izarra Rojas, Princesa Medrano Alhuay, Karla Vasquez Cajachahua, Estela Huaman Angeles, Jorge Giraldo Chavez, Lilian Huarca Balbin, Lisbet Roxana Inga Angulo, Maria Sandra Villar Saavedra, Henri Bailon Calderon, Lely Solari Zerpa, Gloria Arotinco Garayar. Equipo de vigilancia genomica del Instituto Nacional de Salud.
EPI_ISL_13842269, EPI_ISL_13842548	Center for Virology, Medical University of Vienna	Medical University of Vienna Center for Virology	Jeremy V. Camp, Monika Redlberger-Fritz, Stephan W. Aberle
EPI_ISL_13889435, EPI_ISL_13889436, EPI_ISL_13889437, EPI_ISL_13889438, EPI_ISL_13889439, EPI_ISL_13889440, EPI_ISL_13889441, EPI_ISL_13889442, EPI_ISL_13889443, EPI_ISL_13889444, EPI_ISL_13889445, EPI_ISL_13889446, EPI_ISL_13889447, EPI_ISL_13889448, EPI_ISL_13889449, EPI_ISL_13889450, EPI_ISL_13889515, EPI_ISL_13889590, EPI_ISL_13889660, EPI_ISL_13889729, EPI_ISL_13889796, EPI_ISL_13889908, EPI_ISL_13889977, EPI_ISL_13890048, EPI_ISL_13890135, EPI_ISL_13890204, EPI_ISL_13890273, EPI_ISL_13890338, EPI_ISL_13890408, EPI_ISL_13890464, EPI_ISL_13890465, EPI_ISL_13890466, EPI_ISL_13890467, EPI_ISL_13890468, EPI_ISL_13890469, EPI_ISL_13890470, EPI_ISL_13890471, EPI_ISL_13890472, EPI_ISL_13890473, EPI_ISL_13890474, EPI_ISL_13890475, EPI_ISL_13890476, EPI_ISL_13890477, EPI_ISL_13890478, EPI_ISL_13890479, EPI_ISL_13890480, EPI_ISL_13890481, EPI_ISL_13890482			
see above	Charité Universitätsmedizin Berlin, Institut für Virologie/Labor Berlin	Charité Universitätsmedizin Berlin, Institut für Virologie	Terry C. Jones, Julia Schneider, Barbara Mühlemann, Talitha Veith, Jörn Beheim-Schwarzbach, Julia Tesch, Marie Luisa Schmidt, Felix Walper, Tobias Bleicker, Caroline Isner, Frieder Pfäfflin, Ricardo Niklas Werner, Victor M. Corman, Christian Drosten
EPI_ISL_13891126	Ministry of Health Turkey	Ministry of Health Turkey	Fatma Bayrakdar, Suleyman Yalcin, Gulay Korukluoglu
EPI_ISL_13908328	Center of Diagnostics and Vaccine Development, Centers for Disease Control	Center of Diagnostics and Vaccine Development, Centers for Disease Control	Lin,J.-H., Chiu,S.-C., Huang,H.-I., Huang,W.-L., Fann,W.-B., Hsieh,P.-Y., Hsu,S.-C., Liu,P.-C., Chang,T.-Y. and Yang,J.-Y.
EPI_ISL_13908329, EPI_ISL_13908332, EPI_ISL_13908333, EPI_ISL_13908334, EPI_ISL_13908335, EPI_ISL_13908336, EPI_ISL_13908337, EPI_ISL_13908338, EPI_ISL_13908339, EPI_ISL_13908340, EPI_ISL_13908341, EPI_ISL_13908342, EPI_ISL_13908343, EPI_ISL_13908345			
see above	Public Health Agency of Canada, National Microbiology Laboratory	Public Health Agency of Canada, National Microbiology Laboratory	Duggan,A., Hole,D., Yadav,C., Knox,N., Chapel,M., Tyler,A., Haidl,E., Domselaar,G.V., Antonation,K., Audet,J., Fernando,L., Hagan,M., Safronetz,D., Graham,M., Peters,G., Go,A., Laminman,V., Kaplen,B., Leung,A., Jolly,G., Fafard,J., Charest,H. and Levade,I.
EPI_ISL_13908346, EPI_ISL_13908347, EPI_ISL_13908348, EPI_ISL_13908349	Centre for Biological Threats, Highly Pathogenic Viruses, Robert Koch Institute	Centre for Biological Threats, Highly Pathogenic Viruses, Robert Koch Institute	Brinkmann,A., Kohl,C., Pape,K., Uddin,S., Schrick,L., Michel,J., Jessen,H., Schaade,L. and Nitsche,A.
EPI_ISL_13908350, EPI_ISL_13908351, EPI_ISL_13908352, EPI_ISL_13908353, EPI_ISL_13908354, EPI_ISL_13908355, EPI_ISL_13908356, EPI_ISL_13908357, EPI_ISL_13908358, EPI_ISL_13908359, EPI_ISL_13908360, EPI_ISL_13908361, EPI_ISL_13908362, EPI_ISL_13908363, EPI_ISL_13908364, EPI_ISL_13908365			
see above	Centre for Biological Threats, Highly Pathogenic Viruses, Robert Koch Institute	Centre for Biological Threats, Highly Pathogenic Viruses, Robert Koch Institute	Brinkmann,A., Kohl,C., Pape,K., Uddin,S., Schrick,L., Michel,J., Schaade,L. and Nitsche,A.
EPI_ISL_13953610, EPI_ISL_13953611	Indian Council of Medical Research-National Institute of Virology	Indian Council of Medical Research-National Institute of Virology	Pragya Yadav, Rima Sahay, Anita Aich Shete, Sreelekshmy Mohandas, Priya Abraham
EPI_ISL_13955501	Public Health Authority of the Slovak Republic	Laboratory of Genomics and Bioinformatics, Comenius University Science Park	Tomáš Szemes, Edita Staroňová, Elena Tichá, Lucia Ševčíková, Terézia Vrabľová, Tatiana Sedláčková, Miroslav Böhmer, Jaroslav Budiš, Pavol Mišenko
EPI_ISL_13958697	Research and Evaluation, UKHSA	Research and Evaluation, UKHSA	Groves,N., Osman,K.L., Lewandowski,K.S., Carter,D.P., Pullan,S.T., Myers,R., Vipond,R. and Chand,M.
EPI_ISL_13983354, EPI_ISL_13983355	Instituto de Infectologia Emilio Ribas	Instituto Adolfo Lutz Strategic Laboratory	Claudio Tavares Sacchi, Karoline Rodrigues Campos, Ariadne Ferreira Amarante, Marlon Benedito Nascimento Santos, Alex Domingos Reis, Adriano Abbud, Adriana Bugno
EPI_ISL_13983356	INSPI-Centro de Referencia Nacional de Virus Exantemáticos, Gastroentéricos y Transmitido por Vectores.	INSPI-Dirección Técnica de Investigación, Desarrollo e Innovación INSPI-Centro de Referencia Nacional de Genómica, Secuenciación y Bioinformática	Andrés Carrazco-Montalvo, Diana Gutiérrez, Naomi Mora, Silvia Salgado-Cisneros, Johana Parrales-Valdiviezo, Martha Sánchez-Domenech, Diego Morales, Gulnara Borja-Cabrera, Leandro Patiño*.
EPI_ISL_13983888	Bangkok Hospital Phuket	National Institute of Health, Department of Medical Sciences, Ministry of Public Health, Thailand	Pilailuk Okada; Siripaporn Phuygun; Nuttida Thongpramul; Thanutsapa Thanadachakul; Kazuhisa Okada; Archawin Rojanawiwat; Chakkarat Pitayawonganon; Supakit Sirilak
EPI_ISL_13993734, EPI_ISL_13993735, EPI_ISL_13993737, EPI_ISL_13993738, EPI_ISL_13993739	California Department of Public Health	California Department of Public Health	Viral and Rickettsial Disease Laboratory
EPI_ISL_14003930	University of Rochester Medical Center	University of Rochester Medical Center	Andrew Cameron, Mondraya Howard, Sara Connelly, Dwight Hardy, Kelly DeLary
EPI_ISL_14011193	Bangkok Hospital Phuket	Thai Red Cross Emerging Infectious Diseases Clinical Center and Faculty of Medicine, Chulalongkorn University	Kusak Kukiattikoon, Waritta Dararattanaroj, Rome Buathong, Supaporn Wacharapluesadee, Sininat Petcharat, Ananporn Supataragul, Stefan Fernandez, Achawin Rojanaviwat, Chonticha Klungthong, Pilailuk Okada, Khajohn Joonlasak, Chakkarat Pitayawonganon, Opass Putcharoen
EPI_ISL_14021725	Hosp. Municipal Enf. Antonio Policarpo de Oliveira	Instituto Adolfo Lutz Strategic Laboratory	Claudio Tavares Sacchi, Karoline Rodrigues Campos, Ariadne Ferreira Amarante, Marlon Benedito Nascimento Santos, Alex Domingos Reis, Adriano Abbud, Adriana Bugno
EPI_ISL_14033203	Hanimal Health, Istituto Zooprofilattico Sperimentale del Mezzogiorno	Hanimal Health, Istituto Zooprofilattico Sperimentale del Mezzogiorno	Viscardi,M., Cardillo,L., De Martinis,C., Cozzolino,L. and Fusco,G.
EPI_ISL_14033204, EPI_ISL_14033205, EPI_ISL_14033206, EPI_ISL_14033207, EPI_ISL_14033208, EPI_ISL_14033209, EPI_ISL_14033210, EPI_ISL_14033211, EPI_ISL_14033212, EPI_ISL_14033213	Laboratory Medicine, UW Virology	Laboratory Medicine, UW Virology	Sereewit,J., Xie,H., Pavitra,R. and Greninger,A.
EPI_ISL_14049244, EPI_ISL_14049245	Indian Council of Medical Research-National Institute of Virology	Indian Council of Medical Research-National Institute of Virology	Pragya Yadav, Rima Sahay, Anita Aich Shete, Sreelekshmy Mohandas, Priya Abraham
EPI_ISL_14050451, EPI_ISL_14050453, EPI_ISL_14050454, EPI_ISL_14050455, EPI_ISL_14050456, EPI_ISL_14050458	Public Health Agency of Canada, National Microbiology Laboratory	Public Health Agency of Canada, National Microbiology Laboratory	Duggan,A., Hole,D., Yadav,C., Knox,N., Tyler,A., Haidl,E., Chapel,M., Domselaar,G.V., Graham,M., Audet,J., Fernando,L., Hagan,M., Safronetz,D., Leung,A., Peters,G., Go,A., Laminman,V., Kaplen,B., Antonation,K., Jolly,G., Griffiths,E., Charest,H., Levade,I. and Fafard,J.
EPI_ISL_14056410	Azienda Sanitaria dell'Alto Adige - Laboratorio Aziendale di Microbiologia e Virologia	Azienda Sanitaria dell'Alto Adige - Laboratorio Aziendale di Microbiologia e Virologia	Teresa Fortini, Elisabetta Incrocci, Elisabetta Giacobazzi, Elisa Masi, Irene Bianconi, Elisabetta Pagani
EPI_ISL_14070493, EPI_ISL_14070852, EPI_ISL_14070854, EPI_ISL_14070855	Instituto de Infectologia Emilio Ribas	Instituto Adolfo Lutz Strategic Laboratory	Claudio Tavares Sacchi, Karoline Rodrigues Campos, Ariadne Ferreira Amarante, Marlon Benedito Nascimento Santos, Alex Domingos Reis, Adriano Abbud, Adriana Bugno
EPI_ISL_14089382	Pathogen Genomics Lab, National Institute for Biomedical Research (INRB)	Pathogen Genomics Lab, National Institute for Biomedical Research (INRB)	Placide Mbala-Kingebeni, Eddy Kinganda-Lusamaki, Adrienne Amuri-Aziza, Elisabeth Pukuta, Catherine Pratt, Nicolas Fernandez, Emmanuel Lokilo Lofiko, Gradi Luakanda Ndelemo, Francisca Muyembe Mawete, Jean Claude Makangara Cigolo, Elisabeth Muyamuna, Raphaël Lumembe Numbi, Gabriel Kabamba Lungenyi, Prince Akil Bandali, Pauline Musuamba Kayembe, Rilia Ola Mpumbe, Emile Malembi, Emmanuel Hasivirwe Vakaniaki, Andrew Rambaut, Nick Loman, Kristian Andersen, Michael Wiley, Ahidjo Ayouba, Steve Ahuka-Mundeke, Martine Peeters, Eric Delaporte, Jean-Jacques Muyembe Tamfum
EPI_ISL_14153982	Vajira Hospital	National Institute of Health, Department of Medical Sciences, Ministry of Public Health, Thailand	Pilailuk Okada; Siripaporn Phuygun; Nuttida Thongpramul; Thanutsapa Thanadachakul; Kazuhisa Okada; Archawin Rojanawiwat; Chakkarat Pitayawonganon; Supakit Sirilak
EPI_ISL_14166709	Medical University of Vienna Center for Virology	Medical University of Vienna Center for Virology	Jeremy V Camp, Monika Redlberger-Fritz, Stephan W. Aberle
EPI_ISL_14167248, EPI_ISL_14167573, EPI_ISL_14167574, EPI_ISL_14167575	Medical University of Vienna Center for Virology	Medical University of Vienna Center for Virology	Jeremy V. Camp, Monika Redlberger-Fritz, Stephan W. Aberle
EPI_ISL_14170200, EPI_ISL_14170201, EPI_ISL_14170203, EPI_ISL_14170204	Erasmus Medical Center Department of Virology	Erasmus Medical Center Department of Virology	Bas Oude Munnink, Marjan Boter, Babette Weller, Babs Verstrepen, Richard Molenkamp, Janette Rahamat-Langendoen, Reina Sikkema, Marion Koopmans
EPI_ISL_14181948, EPI_ISL_14181949, EPI_ISL_14181950, EPI_ISL_14181951, EPI_ISL_14181952, EPI_ISL_14181953, EPI_ISL_14181954, EPI_ISL_14181955, EPI_ISL_14181956, EPI_ISL_14181957, EPI_ISL_14181958, EPI_ISL_14181959, EPI_ISL_14181960			
see above	Institute of Health Carlos III, Bioinformatics Unit	Institute of Health Carlos III, Bioinformatics Unit	Cuesta,I.
EPI_ISL_14189012, EPI_ISL_14189013, EPI_ISL_14189014, EPI_ISL_14189015, EPI_ISL_14189016, EPI_ISL_14189017, EPI_ISL_14189018, EPI_ISL_14189019	Los Angeles County Public Health Laboratories	Los Angeles County Public Health Laboratories	P. Hemarajata et al.
EPI_ISL_14201640, EPI_ISL_14201641, EPI_ISL_14201642, EPI_ISL_14201643, EPI_ISL_14201644, EPI_ISL_14201645	Pathogen Genomics Lab, National Institute for Biomedical Research (INRB)	Pathogen Genomics Lab, National Institute for Biomedical Research (INRB)	Placide Mbala-Kingebeni, Eddy Kinganda-Lusamaki, Adrienne Amuri-Aziza, Elisabeth Pukuta, Catherine Pratt, Nicolas Fernandez, Emmanuel Lokilo Lofiko, Gradi Luakanda Ndelemo, Francisca Muyembe Mawete, Jean Claude Makangara Cigolo, Elisabeth Muyamuna, Raphaël Lumembe Numbi, Gabriel Kabamba Lungenyi, Prince Akil Bandali, Pauline Musuamba Kayembe, Rilia Ola Mpumbe, Emile Malembi, Emmanuel Hasivirwe Vakaniaki, Andrew Rambaut, Nick Loman, Kristian Andersen, Michael Wiley, Ahidjo Ayouba, Steve Ahuka-Mundeke, Martine Peeters, Eric Delaporte, Jean-Jacques Muyembe Tamfum
EPI_ISL_14207724, EPI_ISL_14207725, EPI_ISL_14207726, EPI_ISL_14207727, EPI_ISL_14207728, EPI_ISL_14207729, EPI_ISL_14207730, EPI_ISL_14207731, EPI_ISL_14207732, EPI_ISL_14207733, EPI_ISL_14207734, EPI_ISL_14207735, EPI_ISL_14207736, EPI_ISL_14207737, EPI_ISL_14207738, EPI_ISL_14207739, EPI_ISL_14207740, EPI_ISL_14207741			
see above	Laboratorio de Referencia Nacional de Virus Respiratorio. Centro Nacional de Salud Publica. Instituto Nacional de Salud.	Laboratorio de Referencia Nacional de Virus Respiratorio. Centro Nacional de Salud Publica. Instituto Nacional de Salud.	Carlos Padilla Rojas, Veronica Hurtado Vela, Iris Silva Molina, Luren Sevilla Castañeda, Victor Jimenez Vasquez, Orson Mestanza Millones, Luis Barcena Flores, Wendy Lizarraga Olivares, Alicia Nuñez Llanos, Steve Acedo Lazo, Francisco Ascue Orosco, Kelly Izarra Rojas, Princesa Medrano Alhuay, Karla Vasquez Cajachahua, Estela Huaman Angeles, Jorge Giraldo Chavez, Lilian Huarca Balbin, Lisbet Roxana Inga Angulo, Maria Sandra Villar Saavedra, Henri Bailon Calderon, Lely Solari Zerpa, Gloria Arotinco Garayar. Equipo de vigilancia genomica del Instituto Nacional de Salud.
EPI_ISL_14211644, EPI_ISL_14211645	Public Health Authority of the Slovak Republic	Laboratory of Genomics and Bioinformatics, Comenius University Science Park	Tomáš Szemes, Edita Staroňová, Elena Tichá, Lucia Ševčíková, Terézia Vrabľová, Tatiana Sedláčková, Miroslav Böhmer, Jaroslav Budiš, Pavol Mišenko
EPI_ISL_14216746, EPI_ISL_14216748, EPI_ISL_14216750, EPI_ISL_14216752, EPI_ISL_14216753, EPI_ISL_14216754, EPI_ISL_14216755, EPI_ISL_14216756, EPI_ISL_14216757, EPI_ISL_14216758, EPI_ISL_14216759, EPI_ISL_14216761, EPI_ISL_14216762, EPI_ISL_14216764, EPI_ISL_14216767, EPI_ISL_14216769			
see above	Laboratory Medicine, UW Virology	Laboratory Medicine, UW Virology	Sereewit,J., Xie,H., Roychoudhury,P. and Greninger,A.
EPI_ISL_14224334	Genetica Molecular and Subdepartamento de Virologia ISP Chile	Instituto de Salud Publica de Chile	Paulo C. Covarrubias, Andrés E. Castillo, Constanza Campano, Mariela Guajardo, Bárbara Parra, Rodrigo Fasce Pineda, Jorge Fernández

EPI_ISL_14241409, EPI_ISL_14241410	Department of Clinical Sciences, Institute of Tropica Medicine	Department of Clinical Sciences, Institute of Tropica Medicine	De Baetselier,I., Van Dijck,C., Kenyon,C., Coppens,J., Smet,H., de Block,T., Coppens,S., Vanroye,F., Bugert,J., Girl,P., Liesenborghs,L., Selhorst,P., Arien,K., den Bossche,D.V., Florence,E., Rezende,A.M., Vercauteren,K. and Esbroeck,M.V.
EPI_ISL_14241411	Department of Clinical Sciences, Institute of Tropica Medicine	Department of Clinical Sciences, Institute of Tropica Medicine	Rezende,A.M., de Block,T., Coppens,S., Florence,E., Bracke,S., Brosius,I., Liesenborghs,L., Soentjens,P., Arien,K., Esbroeck,M.V., Selhorst,P. and Vercauteren,K.
EPI_ISL_14241412, EPI_ISL_14241413, EPI_ISL_14241414, EPI_ISL_14241415	Department of Clinical Sciences, Institute of Tropica Medicine	Department of Clinical Sciences, Institute of Tropica Medicine	De Baetselier,I., Van Dijck,C., Kenyon,C., Coppens,J., Smet,H., de Block,T., Coppens,S., Vanroye,F., Bugert,J., Girl,P., Liesenborghs,L., Selhorst,P., Arien,K., den Bossche,D.V., Florence,E., Rezende,A.M., Vercauteren,K. and Esbroeck,M.V.
EPI_ISL_14244555	Centers for Disease Control & Prevention (CDC), Division of High Consequence Pathogens and Pathology (DHCPP-PRB)	Centers for Disease Control & Prevention (CDC), Division of High Consequence Pathogens and Pathology (DHCPP-PRB)	Gigante,C., Ventura,J., Seabolt,M.H., Zhao,H., Wilkins,K., McCollum,A., Hutson,C., Davidson,W., Rao,A., Nash,J. and Li,Y.
EPI_ISL_14244556	Centers for Disease Control & Prevention (CDC), Division of High Consequence Pathogens and Pathology (DHCPP-PRB)	Centers for Disease Control & Prevention (CDC), Division of High Consequence Pathogens and Pathology (DHCPP-PRB)	Gigante,C., Ventura,J., Seabolt,M.H., Zhao,H., Wilkins,K., McCollum,A., Hutson,C., Davidson,W., Rao,A., Nash,J., Sheth,M. and Li,Y.
EPI_ISL_14244557	Centers for Disease Control & Prevention (CDC), Division of High Consequence Pathogens and Pathology (DHCPP-PRB)	Centers for Disease Control & Prevention (CDC), Division of High Consequence Pathogens and Pathology (DHCPP-PRB)	Gigante,C., Xia,D., Seabolt,M., Zhao,H., Wilkins,K., McCollum,A., Hutson,C., Davidson,W., Rao,A., Plipat,N. and Li,Y.
EPI_ISL_14244558	Centers for Disease Control & Prevention (CDC), Division of High Consequence Pathogens and Pathology (DHCPP-PRB)	Centers for Disease Control & Prevention (CDC), Division of High Consequence Pathogens and Pathology (DHCPP-PRB)	Gigante,C., Goldoft,M., Seabolt,M., Zhao,H., Wilkins,K., McCollum,A., Hutson,C., Davidson,W., Rao,A., Holshue,M. and Li,Y.
EPI_ISL_14244559	Centers for Disease Control & Prevention (CDC), Division of High Consequence Pathogens and Pathology (DHCPP-PRB)	Centers for Disease Control & Prevention (CDC), Division of High Consequence Pathogens and Pathology (DHCPP-PRB)	Gigante,C., Pavlick,J., Seabolt,M., Zhao,H., Wilkins,K., McCollum,A., Hutson,C., Davidson,W., Rao,A., Parrott,T. and Li,Y.
EPI_ISL_14251112	University of Rochester Medical Center	University of Rochester Medical Center	Andrew Cameron, Mondraya Howard, Joel Maki, Sara Connelly, Kelly Delary, Dwight Hardy
EPI_ISL_14254435, EPI_ISL_14254436, EPI_ISL_14254437, EPI_ISL_14254438	Erasmus Medical Center Department of Virology	Erasmus Medical Center Department of Virology	Bas Oude Munnink, Marjan Boter, Babette Weller, Babs Verstrepen, Richard Molenkamp, Janette Rahamat-Langendoen, Reina Sikkema, Marion Koopmans
EPI_ISL_14259830	Virology Unit, Azienda Ospedaliero-Universitaria Pisana	Virology Unit, Azienda Ospedaliero-Universitaria Pisana	Vatteroni,M. and Frateschi,S.
EPI_ISL_14295679	Bangkok Hospital Phuket	National Institute of Health, Department of Medical Sciences, Ministry of Public Health, Thailand	Pilailuk Okada; Siripaporn Phuygun; Nuttida Thongpramul; Thanutsapa Thanadachakul; Kazuhisa Okada; Archawin Rojanawiwat; Chakkarat Pitayawonganon; Supakit Sirilak
EPI_ISL_14315314, EPI_ISL_14315315, EPI_ISL_14315316, EPI_ISL_14315317, EPI_ISL_14315318, EPI_ISL_14315319, EPI_ISL_14315320, EPI_ISL_14315321, EPI_ISL_14315322, EPI_ISL_14315323, EPI_ISL_14315324			
see above	Laboratory Medicine, UW Virology	Laboratory Medicine, UW Virology	Sereewit,J., Xie,H., Roychoudhury,P. and Greninger,A.L.
EPI_ISL_14326638, EPI_ISL_14326639, EPI_ISL_14326640, EPI_ISL_14326641, EPI_ISL_14326642, EPI_ISL_14326643	Environmental, Agricultural, and Occupational Health, University of Nebraska Medical Center, 984388 Nebraska Medical Center	Environmental, Agricultural, and Occupational Health, University of Nebraska Medical Center, 984388 Nebraska Medical Center	Tegomoh,B., Cross,S.T., Chapman,R.C., Bernhard,K., McCutchen,E.L., Fauver,J.R., Pratt,C.B., Warden,D.E., Iwen,P.C., Donahue,M. and Wiley,M.R.
EPI_ISL_14326644	Environmental, Agricultural, and Occupational Health, University of Nebraska Medical Center, 984388 Nebraska Medical Center	Environmental, Agricultural, and Occupational Health, University of Nebraska Medical Center, 984388 Nebraska Medical Center	Tegomoh,B., Cross,S.T., Chapman,R.C., Bernhard,K., McCutchen,E.L., Fauver,J.R., Pratt,C.B., Warden,D.E., Iwen,P.C., Donahue,M. and Wiley,M.R
EPI_ISL_14355206, EPI_ISL_14355207, EPI_ISL_14355208, EPI_ISL_14355209, EPI_ISL_14355210, EPI_ISL_14355211	Laboratory Medicine, UW Virology	Laboratory Medicine, UW Virology	Sereewit,J., Xie,H., Roychoudhury,P. and Greninger,A.L.
EPI_ISL_14362272	Centers for Disease Control & Prevention (CDC), Division of High Consequence Pathogens and Pathology (DHCPP-PRB)	Centers for Disease Control & Prevention (CDC), Division of High Consequence Pathogens and Pathology (DHCPP-PRB)	Gigante,C.M., Kubin,G., Seabolt,M.H., Zhao,H., Wilkins,K., McCollum,A., Hutson,C., Davidson,W., Rao,A., White,S.L. and Li,Y.
EPI_ISL_14362274, EPI_ISL_14362276	Centers for Disease Control & Prevention (CDC), Division of High Consequence Pathogens and Pathology (DHCPP-PRB)	Centers for Disease Control & Prevention (CDC), Division of High Consequence Pathogens and Pathology (DHCPP-PRB)	Gigante,C.M., Hughes,S., Seabolt,M.H., Zhao,H., Wilkins,K., McCollum,A., Hutson,C., Davidson,W., Rao,A., Baumgartner,J. and Li,Y.
EPI_ISL_14362278	Centers for Disease Control & Prevention (CDC), Division of High Consequence Pathogens and Pathology (DHCPP-PRB)	Centers for Disease Control & Prevention (CDC), Division of High Consequence Pathogens and Pathology (DHCPP-PRB)	Gigante,C.M., Ghinai,I., Seabolt,M.H., Zhao,H., Wilkins,K., McCollum,A., Hutson,C., Davidson,W., Rao,A., Kerins,J. and Li,Y.
EPI_ISL_14362280	Centers for Disease Control & Prevention (CDC), Division of High Consequence Pathogens and Pathology (DHCPP-PRB)	Centers for Disease Control & Prevention (CDC), Division of High Consequence Pathogens and Pathology (DHCPP-PRB)	Gigante,C.M., Lee,P., Seabolt,M.H., Zhao,H., Wilkins,K., McCollum,A., Hutson,C., Davidson,W., Rao,A., Mendoza,R. and Li,Y.
EPI_ISL_14362282, EPI_ISL_14362285	Centers for Disease Control & Prevention (CDC), Division of High Consequence Pathogens and Pathology (DHCPP-PRB)	Centers for Disease Control & Prevention (CDC), Division of High Consequence Pathogens and Pathology (DHCPP-PRB)	Gigante,C.M., Steidley,B., Seabolt,M.H., Zhao,H., Wilkins,K., McCollum,A., Hutson,C., Davidson,W., Rao,A., Davizon,E.S. and Li,Y.
EPI_ISL_14362287	Centers for Disease Control & Prevention (CDC), Division of High Consequence Pathogens and Pathology (DHCPP-PRB)	Centers for Disease Control & Prevention (CDC), Division of High Consequence Pathogens and Pathology (DHCPP-PRB)	Gigante,C.M., Ventura,J., Seabolt,M.H., Zhao,H., Wilkins,K., McCollum,A., Hutson,C., Davidson,W., Rao,A., Nash,J. and Li,Y.
EPI_ISL_14362289	Centers for Disease Control & Prevention (CDC), Division of High Consequence Pathogens and Pathology (DHCPP-PRB)	Centers for Disease Control & Prevention (CDC), Division of High Consequence Pathogens and Pathology (DHCPP-PRB)	Gigante,C.M., Francis,D., Seabolt,M.H., Zhao,H., Wilkins,K., McCollum,A., Hutson,C., Davidson,W., Rao,A., Escobar,J. and Li,Y.
EPI_ISL_14394060	Ryota Kumagai Tokyo Metropolitan Institute of Public Health, Department of Microbiology	Ryota Kumagai Tokyo Metropolitan Institute of Public Health, Department of Microbiology	Kasuya,F., Negishi,A., Kumagai,R., Hasegawa,M., Fujiwara,T.,Miyake,H., Nagashima,M. and Sadamasu,K.
EPI_ISL_14414948	UMS Parque Industrial Curitiba	Instituto Adolfo Lutz Strategic Laboratory	Claudio Tavares Sacchi, Karoline Rodrigues Campos, Ariadne Ferreira Amarante, Marlon Benedito Nascimento Santos, Alex Domingos Reis, Adriano Abbud, Adriana Bugno
EPI_ISL_14415810	CTA Sao Miguel	Instituto Adolfo Lutz Strategic Laboratory	Claudio Tavares Sacchi, Karoline Rodrigues Campos, Ariadne Ferreira Amarante, Marlon Benedito Nascimento Santos, Alex Domingos Reis, Adriano Abbud, Adriana Bugno
EPI_ISL_14438678, EPI_ISL_14438679, EPI_ISL_14438682, EPI_ISL_14438683, EPI_ISL_14438684, EPI_ISL_14438685, EPI_ISL_14438686, EPI_ISL_14438687, EPI_ISL_14438688, EPI_ISL_14438689, EPI_ISL_14438690, EPI_ISL_14438691, EPI_ISL_14438692, EPI_ISL_14438693, EPI_ISL_14438696, EPI_ISL_14438697, EPI_ISL_14438698			
see above	Rhode Island State Health Laboratory	Rhode Island State Health Laboratory	Kristin Carpenter-Azevedo, Sean Sierra-Patev, Richard C. Huard
EPI_ISL_14439712, EPI_ISL_14439713, EPI_ISL_14439714, EPI_ISL_14439715, EPI_ISL_14439716, EPI_ISL_14439717, EPI_ISL_14439718, EPI_ISL_14439719, EPI_ISL_14439720, EPI_ISL_14439721, EPI_ISL_14439722, EPI_ISL_14439723, EPI_ISL_14439724, EPI_ISL_14439725, EPI_ISL_14439726, EPI_ISL_14439727, EPI_ISL_14439728, EPI_ISL_14439729, EPI_ISL_14439730, EPI_ISL_14439731, EPI_ISL_14439732, EPI_ISL_14439733, EPI_ISL_14439734, EPI_ISL_14439735, EPI_ISL_14439736, EPI_ISL_14439737, EPI_ISL_14439738, EPI_ISL_14439739, EPI_ISL_14439740, EPI_ISL_14439741, EPI_ISL_14439742, EPI_ISL_14439743, EPI_ISL_14439745, EPI_ISL_14439746, EPI_ISL_14439747, EPI_ISL_14439748, EPI_ISL_14439749, EPI_ISL_14439750, EPI_ISL_14439751, EPI_ISL_14439752, EPI_ISL_14439753, EPI_ISL_14439754, EPI_ISL_14439755, EPI_ISL_14439756, EPI_ISL_14439757, EPI_ISL_14439758, EPI_ISL_14439759, EPI_ISL_14439760, EPI_ISL_14439761, EPI_ISL_14439762, EPI_ISL_14439763, EPI_ISL_14439764, EPI_ISL_14439765, EPI_ISL_14439766, EPI_ISL_14439767, EPI_ISL_14439768, EPI_ISL_14439769, EPI_ISL_14439770, EPI_ISL_14439771, EPI_ISL_14439772, EPI_ISL_14439773, EPI_ISL_14439774, EPI_ISL_14439775, EPI_ISL_14439776, EPI_ISL_14439777, EPI_ISL_14439778, EPI_ISL_14439779, EPI_ISL_14439780, EPI_ISL_14439781, EPI_ISL_14439782, EPI_ISL_14439783, EPI_ISL_14439784, EPI_ISL_14439785			
see above	Research and Evaluation, UKHSA	Research and Evaluation, UKHSA	Groves,N., Osman,K.L., Lewandowski,K.S., Carter,D.P., Pullan,S.T., Myers,R., Vipond,R. and Chand,M.
EPI_ISL_14441870, EPI_ISL_14441871, EPI_ISL_14441872, EPI_ISL_14441873, EPI_ISL_14441874, EPI_ISL_14441875, EPI_ISL_14441876, EPI_ISL_14441877, EPI_ISL_14441878, EPI_ISL_14441879, EPI_ISL_14441880, EPI_ISL_14441881, EPI_ISL_14441882, EPI_ISL_14441883, EPI_ISL_14441884, EPI_ISL_14441885, EPI_ISL_14441886, EPI_ISL_14441887			
see above	Rhode Island State Health Laboratory	Rhode Island State Health Laboratory	Kristin Carpenter-Azevedo, Sean Sierra-Patev, Richard C. Huard
EPI_ISL_14445098, EPI_ISL_14445100, EPI_ISL_14445101, EPI_ISL_14445102, EPI_ISL_14445103, EPI_ISL_14445104, EPI_ISL_14445107, EPI_ISL_14445109, EPI_ISL_14445111, EPI_ISL_14445113, EPI_ISL_14445114, EPI_ISL_14445115, EPI_ISL_14445116, EPI_ISL_14445117, EPI_ISL_14445118, EPI_ISL_14445119, EPI_ISL_14445120, EPI_ISL_14445121, EPI_ISL_14445122, EPI_ISL_14445123, EPI_ISL_14445124, EPI_ISL_14445125, EPI_ISL_14445126, EPI_ISL_14445127, EPI_ISL_14445128, EPI_ISL_14445129, EPI_ISL_14445130, EPI_ISL_14445131, EPI_ISL_14445132, EPI_ISL_14445133, EPI_ISL_14445134, EPI_ISL_14445135, EPI_ISL_14445136, EPI_ISL_14445137, EPI_ISL_14445138, EPI_ISL_14445139, EPI_ISL_14445140, EPI_ISL_14445141, EPI_ISL_14445142, EPI_ISL_14445143, EPI_ISL_14445144, EPI_ISL_14445145, EPI_ISL_14445146, EPI_ISL_14445147, EPI_ISL_14445148, EPI_ISL_14445149, EPI_ISL_14445150, EPI_ISL_14445151, EPI_ISL_14445152, EPI_ISL_14445153			
see above	Laboratorio de Referencia Nacional de Virus Respiratorio. Centro Nacional de Salud Publica. Instituto Nacional de Salud.	Laboratorio de Referencia Nacional de Virus Respiratorio. Centro Nacional de Salud Publica. Instituto Nacional de Salud.	Carlos Padilla Rojas, Veronica Hurtado Vela, Iris Silva Molina, Luren Sevilla Castañeda, Victor Jimenez Vasquez, Orson Mestanza Millones, Luis Barcena Flores, Wendy Lizarraga Olivares, Alicia Nuñez Llanos, Steve Acedo Lazo, Francisco Ascue Orosco, Kelly Izarra Rojas, Princesa Medrano Alhuay, Karla Vasquez Cajachahua, Estela Huaman Angeles, Jorge Giraldo Chavez, Lilian Huarca Balbin, Lisbet Roxana Inga Angulo, Maria Sandra Villar Saavedra, Henri Bailon Calderon, Lely Solari Zerpa, Gloria Arotinco Garayar. Equipo de vigilancia genomica del Instituto Nacional de Salud.
EPI_ISL_14445154, EPI_ISL_14445155, EPI_ISL_14445156	Centre for Biological Threats, Highly Pathogenic Viruses, Robert Koch Institute	Centre for Biological Threats, Highly Pathogenic Viruses, Robert Koch Institute	Brinkmann,A., Kohl,C., Pape,K., Uddin,S., Schrick,L., Michel,J., Stocker,H., Schaade,L. and Nitsche,A.
EPI_ISL_14445157, EPI_ISL_14445158, EPI_ISL_14445159, EPI_ISL_14445160, EPI_ISL_14445161, EPI_ISL_14445162, EPI_ISL_14445163, EPI_ISL_14445164, EPI_ISL_14445165	Centre for Biological Threats, Highly Pathogenic Viruses, Robert Koch Institute	Centre for Biological Threats, Highly Pathogenic Viruses, Robert Koch Institute	Brinkmann,A., Kohl,C., Pape,K., Uddin,S., Schrick,L., Michel,J., Jessen,H., Schaade,L. and Nitsche,A.
EPI_ISL_14465517	Centro de Desenvolvimento Científico e Tecnológico (CDCT), Centro Estadual de Vigilância em Saúde (CEVS) da Secretaria Estadual da Saúde (SES-RS)	Centro de Desenvolvimento Científico e Tecnológico (CDCT), Centro Estadual de Vigilância em Saúde (CEVS) da Secretaria Estadual da Saúde (SES-RS)	Richard Steiner Salvato, Regina Bones Barcellos, Fernanda Marques Godinho
EPI_ISL_14467428, EPI_ISL_14467429	Laboratório Central de Saúde Pública do Amazonas - LACEN-AM	Laboratório de Ecologia de Doenças Transmissíveis na Amazônia, Instituto Leônidas e Maria Deane - Fiocruz Amazônia	Victor Souza, Fernanda Nascimento, Matilde Mejía, Dejanane Silva, Luciana Gonçalves, Tatyana Costa Amorim Ramos, Ana Ruth Lima Arcanjo, Valdinete Nascimento, Felipe Naveca on behalf of the Fiocruz COVID-19 Genomic Surveillance Network

EPI_ISL_14486937, EPI_ISL_14487241	Instituto Nacional de Salud	Instituto Nacional de Salud- Dirección de Investigación en Salud Pública	Katherine Laiton-Donato, Diego A. Álvarez-Díaz, Carlos Franco-Muñoz, Héctor A. Ruiz-Moreno, Paola Rojas-Estevez, Alicia Rosales, Daniel Martinez, Sergio Gómez, Astrid Carolina Flores, Franklin Prieto, Diana Walteros, Marcela Mercado-Reyes
EPI_ISL_14487651	Centers for Disease Control & Prevention (CDC), Division of High Consequence Pathogens and Pathology (DHCPP-PRB)	Centers for Disease Control & Prevention (CDC), Division of High Consequence Pathogens and Pathology (DHCPP-PRB)	Gigante,C.M., Hughes,S., Seabolt,M.H., Zhao,H., Wilkins,K., McCollum,A., Hutson,C., Davidson,W., Rao,A., Baumgartner,J. and Li,Y.
EPI_ISL_14487652	Centers for Disease Control & Prevention (CDC), Division of High Consequence Pathogens and Pathology (DHCPP-PRB)	Centers for Disease Control & Prevention (CDC), Division of High Consequence Pathogens and Pathology (DHCPP-PRB)	Gigante,C.M., Griffin-Thomas,L., Seabolt,M.H., Zhao,H., Wilkins,K., McCollum,A., Hutson,C., Davidson,W., Rao,A., Crain,J. and Li,Y.
EPI_ISL_14487653	Centers for Disease Control & Prevention (CDC), Division of High Consequence Pathogens and Pathology (DHCPP-PRB)	Centers for Disease Control & Prevention (CDC), Division of High Consequence Pathogens and Pathology (DHCPP-PRB)	Gigante,C.M., Ghinai,I., Seabolt,M.H., Zhao,H., Wilkins,K., McCollum,A., Hutson,C., Davidson,W., Rao,A., Kerins,J. and Li,Y.
EPI_ISL_14487654	Centers for Disease Control & Prevention (CDC), Division of High Consequence Pathogens and Pathology (DHCPP-PRB)	Centers for Disease Control & Prevention (CDC), Division of High Consequence Pathogens and Pathology (DHCPP-PRB)	Gigante,C.M., Steidley,B., Seabolt,M.H., Zhao,H., Wilkins,K., McCollum,A., Hutson,C., Davidson,W., Rao,A., Davizon,E. and Li,Y.
EPI_ISL_14487655	Centers for Disease Control & Prevention (CDC), Division of High Consequence Pathogens and Pathology (DHCPP-PRB)	Centers for Disease Control & Prevention (CDC), Division of High Consequence Pathogens and Pathology (DHCPP-PRB)	Gigante,C.M., Ghinai,I., Seabolt,M.H., Zhao,H., Wilkins,K., McCollum,A., Hutson,C., Davidson,W., Rao,A., Kerins,J. and Li,Y.
EPI_ISL_14487656	Centers for Disease Control & Prevention (CDC), Division of High Consequence Pathogens and Pathology (DHCPP-PRB)	Centers for Disease Control & Prevention (CDC), Division of High Consequence Pathogens and Pathology (DHCPP-PRB)	Gigante,C.M., Lee,P., Seabolt,M.H., Zhao,H., Wilkins,K., McCollum,A., Hutson,C., Davidson,W., Rao,A., Mendoza,R. and Li,Y.
EPI_ISL_14487657	Centers for Disease Control & Prevention (CDC), Division of High Consequence Pathogens and Pathology (DHCPP-PRB)	Centers for Disease Control & Prevention (CDC), Division of High Consequence Pathogens and Pathology (DHCPP-PRB)	Gigante,C.M., Ghinai,I., Seabolt,M.H., Zhao,H., Wilkins,K., McCollum,A., Hutson,C., Davidson,W., Rao,A., Kerins,J. and Li,Y.
EPI_ISL_14487658	Centers for Disease Control & Prevention (CDC), Division of High Consequence Pathogens and Pathology (DHCPP-PRB)	Centers for Disease Control & Prevention (CDC), Division of High Consequence Pathogens and Pathology (DHCPP-PRB)	Gigante,C.M., Lee,P., Seabolt,M.H., Zhao,H., Wilkins,K., McCollum,A., Hutson,C., Davidson,W., Rao,A., Mendoza,R. and Li,Y.
EPI_ISL_14487659	Centers for Disease Control & Prevention (CDC), Division of High Consequence Pathogens and Pathology (DHCPP-PRB)	Centers for Disease Control & Prevention (CDC), Division of High Consequence Pathogens and Pathology (DHCPP-PRB)	Gigante,C.M., Hauser,J.R., Seabolt,M.H., Zhao,H., Wilkins,K., McCollum,A., Hutson,C., Davidson,W., Rao,A., Mangla,A. and Li,Y.
EPI_ISL_14487660	Centers for Disease Control & Prevention (CDC), Division of High Consequence Pathogens and Pathology (DHCPP-PRB)	Centers for Disease Control & Prevention (CDC), Division of High Consequence Pathogens and Pathology (DHCPP-PRB)	Gigante,C.M., Ghinai,I., Seabolt,M.H., Zhao,H., Wilkins,K., McCollum,A., Hutson,C., Davidson,W., Rao,A., Kerins,J. and Li,Y.
EPI_ISL_14494949	Division of High-risk Pathogens, Korea Disease Control and Prevention Agency	Division of High-risk Pathogens, Korea Disease Control and Prevention Agency	Rhie,G.-E.
EPI_ISL_14515100, EPI_ISL_14515101, EPI_ISL_14515102, EPI_ISL_14515103, EPI_ISL_14515104, EPI_ISL_14515105, EPI_ISL_14515106, EPI_ISL_14515107, EPI_ISL_14515108, EPI_ISL_14515109, EPI_ISL_14515110, EPI_ISL_14515111, EPI_ISL_14515112, EPI_ISL_14515113			
see above	Centre for Biological Threats, Highly Pathogenic Viruses, Robert Koch Institute	Centre for Biological Threats, Highly Pathogenic Viruses, Robert Koch Institute	Brinkmann,A., Kohl,C., Pape,K., Uddin,S., Schrick,L., Michel,J., Jessen,H., Schaade,L. and Nitsche,A.
EPI_ISL_14515114, EPI_ISL_14515115, EPI_ISL_14515116, EPI_ISL_14515117, EPI_ISL_14515118, EPI_ISL_14515119, EPI_ISL_14515120, EPI_ISL_14515121, EPI_ISL_14515122, EPI_ISL_14515123, EPI_ISL_14515124, EPI_ISL_14515125, EPI_ISL_14515126, EPI_ISL_14515127, EPI_ISL_14515128, EPI_ISL_14515129, EPI_ISL_14515130, EPI_ISL_14515131, EPI_ISL_14515132, EPI_ISL_14515133, EPI_ISL_14515134, EPI_ISL_14515135, EPI_ISL_14515136, EPI_ISL_14515137, EPI_ISL_14515138, EPI_ISL_14515139, EPI_ISL_14515140, EPI_ISL_14515141, EPI_ISL_14515142, EPI_ISL_14515143, EPI_ISL_14515144, EPI_ISL_14515145, EPI_ISL_14515146, EPI_ISL_14515147, EPI_ISL_14515148, EPI_ISL_14515149, EPI_ISL_14515150, EPI_ISL_14515151, EPI_ISL_14515152			
see above	Centre for Biological Threats, Highly Pathogenic Viruses, Robert Koch Institute	Centre for Biological Threats, Highly Pathogenic Viruses, Robert Koch Institute	Brinkmann,A., Kohl,C., Pape,K., Uddin,S., Schrick,L., Michel,J., Schaade,L. and Nitsche,A.
EPI_ISL_14515153, EPI_ISL_14515154, EPI_ISL_14515155, EPI_ISL_14515156	Centre for Biological Threats, Highly Pathogenic Viruses, Robert Koch Institute	Centre for Biological Threats, Highly Pathogenic Viruses, Robert Koch Institute	Brinkmann,A., Kohl,C., Pape,K., Uddin,S., Schrick,L., Michel,J., Jessen,H., Schaade,L. and Nitsche,A.
EPI_ISL_14515157, EPI_ISL_14515158, EPI_ISL_14515159, EPI_ISL_14515160, EPI_ISL_14515161, EPI_ISL_14515162, EPI_ISL_14515163, EPI_ISL_14515164, EPI_ISL_14515165, EPI_ISL_14515166, EPI_ISL_14515167, EPI_ISL_14515168, EPI_ISL_14515169, EPI_ISL_14515170, EPI_ISL_14515171, EPI_ISL_14515172			
see above	Centre for Biological Threats, Highly Pathogenic Viruses, Robert Koch Institute	Centre for Biological Threats, Highly Pathogenic Viruses, Robert Koch Institute	Brinkmann,A., Kohl,C., Pape,K., Uddin,S., Schrick,L., Michel,J., Schaade,L. and Nitsche,A.
EPI_ISL_14515173, EPI_ISL_14515174, EPI_ISL_14515175, EPI_ISL_14515176, EPI_ISL_14515178, EPI_ISL_14515180, EPI_ISL_14515181, EPI_ISL_14515182, EPI_ISL_14515183, EPI_ISL_14515184, EPI_ISL_14515185, EPI_ISL_14515186, EPI_ISL_14515187, EPI_ISL_14515188, EPI_ISL_14515189, EPI_ISL_14515190, EPI_ISL_14515191, EPI_ISL_14515192, EPI_ISL_14515193, EPI_ISL_14515194, EPI_ISL_14515195, EPI_ISL_14515196, EPI_ISL_14515197, EPI_ISL_14515198, EPI_ISL_14515199, EPI_ISL_14515200, EPI_ISL_14515201, EPI_ISL_14515203, EPI_ISL_14515204, EPI_ISL_14515206, EPI_ISL_14515208, EPI_ISL_14515209, EPI_ISL_14515210, EPI_ISL_14515211, EPI_ISL_14515212, EPI_ISL_14515215, EPI_ISL_14515216, EPI_ISL_14515217, EPI_ISL_14515218, EPI_ISL_14515219, EPI_ISL_14515220, EPI_ISL_14515221, EPI_ISL_14515222, EPI_ISL_14515224, EPI_ISL_14515225, EPI_ISL_14515226, EPI_ISL_14515227, EPI_ISL_14515228			
see above	Department of Infectious Diseases, National Institute of Health Doutor Ricardo Jorge, Portugal (INSA)	Department of Infectious Diseases, National Institute of Health Doutor Ricardo Jorge, Portugal (INSA)	Isidro,J., Borges,V., Pinto,M., Sobral,D., Santos,J., Nunes,A., Mixao,V., Ferreira,R., Santos,D., Duarte,S., Vieira,L., Borrego,M.J., Nuncio,S., Lopes de Carvalho,I., Pelerito,A., Cordeiro,R. and Gomes,J.P.
EPI_ISL_14526939, EPI_ISL_14526940, EPI_ISL_14526941, EPI_ISL_14526942, EPI_ISL_14526943, EPI_ISL_14526944, EPI_ISL_14526945, EPI_ISL_14526947, EPI_ISL_14526948, EPI_ISL_14526949, EPI_ISL_14526950, EPI_ISL_14526951, EPI_ISL_14526952, EPI_ISL_14526953, EPI_ISL_14526954, EPI_ISL_14526955, EPI_ISL_14526956			
see above	Connecticut Department of Public Health	Grubaugh Lab - Yale School of Public Health	Nicholas F. G. Chen, Chrispin Chaguza, Kien Pham, Nathan D. Grubaugh, Christina Nishimura, Claire Pearson, Kutluhan Incekara, Jian Ping Huang, Emily Gagnon, Ethan Reever, Jafar Razeq, Anthony Muyombwe, Chantal B. F. Vogels
EPI_ISL_14541645, EPI_ISL_14541647, EPI_ISL_14541649, EPI_ISL_14541652, EPI_ISL_14541654	Public Health Authority of the Slovak Republic	Laboratory of Genomics and Bioinformatics, Comenius University Science Park	Tomáš Szemes, Edita Staroňová, Elena Tichá, Lucia Ševčíková, Terézia Vrabľová, Tatiana Sedláčková, Miroslav Böhmer, Jaroslav Budiš, Pavol Mišenko
EPI_ISL_14553812	Hospital CIMA San Jose	Inciensa, Instituto de Investigación y Enseñanza en Nutrición y Salud	Francisco Duarte, Ana Isela Ruiz-Gonzalez, Hillary Serrano, Diana Cantillo, Claudio Soto-Garita, Gustavo Vega, Estela Cordero, Adriana Godinez & Melany Calderon
EPI_ISL_14561914, EPI_ISL_14561915, EPI_ISL_14561916, EPI_ISL_14561917, EPI_ISL_14561918, EPI_ISL_14561919, EPI_ISL_14561920, EPI_ISL_14561921, EPI_ISL_14561922, EPI_ISL_14561923, EPI_ISL_14561924, EPI_ISL_14561925, EPI_ISL_14561926, EPI_ISL_14561927, EPI_ISL_14561928, EPI_ISL_14561929, EPI_ISL_14561930, EPI_ISL_14561931, EPI_ISL_14561932			
see above	Los Angeles County Public Health Laboratories	Los Angeles County Public Health Laboratories	P. Hemarajata et al.
EPI_ISL_14562478, EPI_ISL_14562479, EPI_ISL_14562480, EPI_ISL_14562481, EPI_ISL_14562482, EPI_ISL_14562483, EPI_ISL_14562484, EPI_ISL_14562485, EPI_ISL_14562486, EPI_ISL_14562487, EPI_ISL_14562488, EPI_ISL_14562489, EPI_ISL_14562490, EPI_ISL_14562491, EPI_ISL_14562492, EPI_ISL_14562493, EPI_ISL_14562494, EPI_ISL_14562495, EPI_ISL_14562496, EPI_ISL_14562497, EPI_ISL_14562498, EPI_ISL_14562499, EPI_ISL_14562500, EPI_ISL_14562501, EPI_ISL_14562502			
see above	Laboratory Medicine, UW Virology	Laboratory Medicine, UW Virology	Sereewit,J., Xie,H., Roychoudhury,P. and Greninger,A.L.
EPI_ISL_14562503, EPI_ISL_14562504, EPI_ISL_14562505, EPI_ISL_14562506, EPI_ISL_14562507, EPI_ISL_14562508, EPI_ISL_14562509, EPI_ISL_14562510, EPI_ISL_14562511, EPI_ISL_14562512	Centre for Biological Threats, Highly Pathogenic Viruses, Robert Koch Institute	Centre for Biological Threats, Highly Pathogenic Viruses, Robert Koch Institute	Brinkmann,A., Kohl,C., Uddin,S., Pape,K., Schrick,L., Michel,J., Schaade,L. and Nitsche,A.
EPI_ISL_14571429	Hosp. Municipal Dr. Jose de Carvalho Florence	Instituto Adolfo Lutz Strategic Laboratory	Claudio Tavares Sacchi, Karoline Rodrigues Campos, Ariadne Ferreira Amarante, Marlon Benedito Nascimento Santos, Alex Domingos Reis, Adriano Abbud, Adriana Bugno
EPI_ISL_14571433	Casa de Saude Stella Maris	Instituto Adolfo Lutz Strategic Laboratory	Claudio Tavares Sacchi, Karoline Rodrigues Campos, Ariadne Ferreira Amarante, Marlon Benedito Nascimento Santos, Alex Domingos Reis, Adriano Abbud, Adriana Bugno
EPI_ISL_14571435	Secrtetaria Municipal de Saude de Sertaozinho	Instituto Adolfo Lutz Strategic Laboratory	Claudio Tavares Sacchi, Karoline Rodrigues Campos, Ariadne Ferreira Amarante, Marlon Benedito Nascimento Santos, Alex Domingos Reis, Adriano Abbud, Adriana Bugno
EPI_ISL_14571439	Secrtetaria Municipal de Saude de Sata Barbara D Oeste	Instituto Adolfo Lutz Strategic Laboratory	Claudio Tavares Sacchi, Karoline Rodrigues Campos, Ariadne Ferreira Amarante, Marlon Benedito Nascimento Santos, Alex Domingos Reis, Adriano Abbud, Adriana Bugno
EPI_ISL_14571441	Hosp. Municipal Dr. Waldemar Tebaldi	Instituto Adolfo Lutz Strategic Laboratory	Claudio Tavares Sacchi, Karoline Rodrigues Campos, Ariadne Ferreira Amarante, Marlon Benedito Nascimento Santos, Alex Domingos Reis, Adriano Abbud, Adriana Bugno
EPI_ISL_14571442	Instituto de Infectologia Emilio Ribas II Baixada Santista	Instituto Adolfo Lutz Strategic Laboratory	Claudio Tavares Sacchi, Karoline Rodrigues Campos, Ariadne Ferreira Amarante, Marlon Benedito Nascimento Santos, Alex Domingos Reis, Adriano Abbud, Adriana Bugno
EPI_ISL_14571444	UBDS DR. Italo Baruffi Castelo Branco	Instituto Adolfo Lutz Strategic Laboratory	Claudio Tavares Sacchi, Karoline Rodrigues Campos, Ariadne Ferreira Amarante, Marlon Benedito Nascimento Santos, Alex Domingos Reis, Adriano Abbud, Adriana Bugno
EPI_ISL_14583298, EPI_ISL_14583299, EPI_ISL_14583300, EPI_ISL_14583301, EPI_ISL_14583302, EPI_ISL_14583303, EPI_ISL_14583305, EPI_ISL_14583306, EPI_ISL_14583307, EPI_ISL_14583308, EPI_ISL_14583309			
see above	Rhode Island State Health Laboratory	Rhode Island State Health Laboratory	Kristin Carpenter-Azevedo, Sean Sierra-Patev, Richard C. Huard
EPI_ISL_14584274, EPI_ISL_14584275, EPI_ISL_14584276, EPI_ISL_14584277, EPI_ISL_14584278, EPI_ISL_14584279, EPI_ISL_14584281, EPI_ISL_14584282, EPI_ISL_14584283, EPI_ISL_14584284, EPI_ISL_14584286, EPI_ISL_14584287, EPI_ISL_14584289, EPI_ISL_14584290, EPI_ISL_14584291, EPI_ISL_14584292, EPI_ISL_14584293, EPI_ISL_14584294, EPI_ISL_14584295, EPI_ISL_14584296, EPI_ISL_14584297, EPI_ISL_14584298, EPI_ISL_14584299, EPI_ISL_14584300, EPI_ISL_14584301, EPI_ISL_14584302, EPI_ISL_14584303, EPI_ISL_14584304, EPI_ISL_14584305, EPI_ISL_14584306, EPI_ISL_14584307, EPI_ISL_14584308, EPI_ISL_14584309, EPI_ISL_14584310, EPI_ISL_14584311			
see above	Laboratorio de Referencia Nacional de Virus Respiratorio. Centro Nacional de Salud Publica. Instituto Nacional de Salud.	Laboratorio de Referencia Nacional de Virus Respiratorio. Centro Nacional de Salud Publica. Instituto Nacional de Salud.	Carlos Padilla Rojas, Veronica Hurtado Vela, Iris Silva Molina, Luren Sevilla Castañeda, Victor Jimenez Vasquez, Orson Mestanza Millones, Luis Barcena Flores, Wendy Lizarraga Olivares, Alicia Nuñez Llanos, Steve Acedo Lazo, Francisco Ascue Orosco, Kelly Izarra Rojas, Princesa Medrano Alhuay, Karla Vasquez Cajachahua, Estela Huaman Angeles, Jorge Giraldo Chavez, Lilian Huarca Balbin, Lisbet Roxana Inga Angulo, Maria Sandra Villar Saavedra, Henri Bailon Calderon, Lely Solari Zerpa, Gloria Arotinco Garayar. Equipo de vigilancia genomica del Instituto Nacional de Salud.
EPI_ISL_14586688	Public Health Authority of the Slovak Republic	Laboratory of Genomics and Bioinformatics, Comenius University Science Park	Tomáš Szemes, Edita Staroňová, Elena Tichá, Lucia Ševčíková, Terézia Vrabľová, Tatiana Sedláčková, Miroslav Böhmer, Jaroslav Budiš, Pavol Mišenko
EPI_ISL_14587544, EPI_ISL_14587545, EPI_ISL_14587546, EPI_ISL_14587548, EPI_ISL_14587549, EPI_ISL_14587550, EPI_ISL_14587551	Public Health Agency of Canada, National Microbiology Laboratory	Public Health Agency of Canada, National Microbiology Laboratory	Duggan,A., Hole,D., Yadav,C., Knox,N., Tyler,A., Haidl,E., Chapel,M., Domselaar,G.V., Graham,M., Audet,J., Fernando,L., Hagan,M., Safronetz,D., Leung,A., Peters,G., Go,A., Laminman,V., Kaplen,B., Antonation,K., Griffiths,E., Jolly,G., Charest,H., Levade,I. and Fafard,J.

EPI_ISL_14587552, EPI_ISL_14587553, EPI_ISL_14587554, EPI_ISL_14587555, EPI_ISL_14587556, EPI_ISL_14587557, EPI_ISL_14587558	Centre for Biological Threats, Highly Pathogenic Viruses, Robert Koch Institute	Centre for Biological Threats, Highly Pathogenic Viruses, Robert Koch Institute	Brinkmann,A., Kohl,C., Pape,K., Uddin,S., Schrick,L., Michel,J., Friesen,J., Schaade,L. and Nitsche,A.
EPI_ISL_14594041, EPI_ISL_14594042, EPI_ISL_14594043, EPI_ISL_14594047, EPI_ISL_14594049, EPI_ISL_14594050, EPI_ISL_14594051, EPI_ISL_14594052, EPI_ISL_14594053, EPI_ISL_14594054, EPI_ISL_14594055, EPI_ISL_14594056			
see above	Public Health Agency of Canada, National Microbiology Laboratory	Public Health Agency of Canada, National Microbiology Laboratory	Duggan,A., Hole,D., Yadav,C., Knox,N., Tyler,A., Haidl,E., Chapel,M., Domselaar,G.V., Graham,M., Audet,J., Fernando,L., Antonation,K., Safronetz,D., Hagan,M., Peters,G., Go,A., Laminman,V., Kaplen,B., Leung,A., Griffiths,E., Jolly,G., Eshaghi,A., Gubbay,J.B., Hasso,M., Marchand-Austin,A., Olsha,R. and Patel,S.N.
EPI_ISL_14615579	RSUPN dr. Cipto Mangunkusumo	National Institute of Health Research and Development	Hana Apsari Pawestri, Arie Ardiansyah Nugraha, Fajar Nur Sulistiyohadi, Subangkit, Krisna NA Pangesti, Tze Minn Mak, I Gede Made Wirabrata
EPI_ISL_14621525, EPI_ISL_14621526	Virology, APHP Pitie Salpetriere SU	Virology, APHP Pitie Salpetriere SU	Seang,S., Burrel,S., Todesco,E., Leducq,V., Monsel,G., Le Pluart,D., Cordevant,C., Pourcher,V. and Palich,R.
EPI_ISL_14622055	Instituto de Infectologia Emilio Ribas	Instituto Adolfo Lutz Strategic Laboratory	Claudio Tavares Sacchi, Karoline Rodrigues Campos, Ariadne Ferreira Amarante, Marlon Benedito Nascimento Santos, Alex Domingos Reis, Adriano Abbud, Adriana Bugno
EPI_ISL_14622520	UBS Jovaia	Instituto Adolfo Lutz Strategic Laboratory	Claudio Tavares Sacchi, Karoline Rodrigues Campos, Ariadne Ferreira Amarante, Marlon Benedito Nascimento Santos, Alex Domingos Reis, Adriano Abbud, Adriana Bugno
EPI_ISL_14622705	UBS Jardim Santista	Instituto Adolfo Lutz Strategic Laboratory	Claudio Tavares Sacchi, Karoline Rodrigues Campos, Ariadne Ferreira Amarante, Marlon Benedito Nascimento Santos, Alex Domingos Reis, Adriano Abbud, Adriana Bugno
EPI_ISL_14622706	Centro de Referencia Modulo I SAE II Bauru	Instituto Adolfo Lutz Strategic Laboratory	Claudio Tavares Sacchi, Karoline Rodrigues Campos, Ariadne Ferreira Amarante, Marlon Benedito Nascimento Santos, Alex Domingos Reis, Adriano Abbud, Adriana Bugno
EPI_ISL_14622707	USF Boicucanga I Sao Sebastiao	Instituto Adolfo Lutz Strategic Laboratory	Claudio Tavares Sacchi, Karoline Rodrigues Campos, Ariadne Ferreira Amarante, Marlon Benedito Nascimento Santos, Alex Domingos Reis, Adriano Abbud, Adriana Bugno
EPI_ISL_14622913	Secretaria Municipal de Saude de Caxias do Sul	Instituto Adolfo Lutz Strategic Laboratory	Claudio Tavares Sacchi, Karoline Rodrigues Campos, Ariadne Ferreira Amarante, Marlon Benedito Nascimento Santos, Alex Domingos Reis, Adriano Abbud, Adriana Bugno
EPI_ISL_14622953	Sistema de Vigilancia em Saude Viamao	Instituto Adolfo Lutz Strategic Laboratory	Claudio Tavares Sacchi, Karoline Rodrigues Campos, Ariadne Ferreira Amarante, Marlon Benedito Nascimento Santos, Alex Domingos Reis, Adriano Abbud, Adriana Bugno
EPI_ISL_14622960	Vigilancia Epidemiologica Municipal	Instituto Adolfo Lutz Strategic Laboratory	Claudio Tavares Sacchi, Karoline Rodrigues Campos, Ariadne Ferreira Amarante, Marlon Benedito Nascimento Santos, Alex Domingos Reis, Adriano Abbud, Adriana Bugno
EPI_ISL_14623175	Centro de Referencia em Especialidades Central Rib Preto	Instituto Adolfo Lutz Strategic Laboratory	Claudio Tavares Sacchi, Karoline Rodrigues Campos, Ariadne Ferreira Amarante, Marlon Benedito Nascimento Santos, Alex Domingos Reis, Adriano Abbud, Adriana Bugno
EPI_ISL_14623523	Laboratorio Municipal de Piracicaba	Instituto Adolfo Lutz Strategic Laboratory	Claudio Tavares Sacchi, Karoline Rodrigues Campos, Ariadne Ferreira Amarante, Marlon Benedito Nascimento Santos, Alex Domingos Reis, Adriano Abbud, Adriana Bugno
EPI_ISL_14623704	Unidade Basica de Saude Esplanada	Instituto Adolfo Lutz Strategic Laboratory	Claudio Tavares Sacchi, Karoline Rodrigues Campos, Ariadne Ferreira Amarante, Marlon Benedito Nascimento Santos, Alex Domingos Reis, Adriano Abbud, Adriana Bugno
EPI_ISL_14624411	Hospital Albert Sabin Atibaia	Instituto Adolfo Lutz Strategic Laboratory	Claudio Tavares Sacchi, Karoline Rodrigues Campos, Ariadne Ferreira Amarante, Marlon Benedito Nascimento Santos, Alex Domingos Reis, Adriano Abbud, Adriana Bugno
EPI_ISL_14624610	USAFA Forte	Instituto Adolfo Lutz Strategic Laboratory	Claudio Tavares Sacchi, Karoline Rodrigues Campos, Ariadne Ferreira Amarante, Marlon Benedito Nascimento Santos, Alex Domingos Reis, Adriano Abbud, Adriana Bugno
EPI_ISL_14624698	Centro de Referencia em AIDS SECRAIDS	Instituto Adolfo Lutz Strategic Laboratory	Claudio Tavares Sacchi, Karoline Rodrigues Campos, Ariadne Ferreira Amarante, Marlon Benedito Nascimento Santos, Alex Domingos Reis, Adriano Abbud, Adriana Bugno
EPI_ISL_14624832	Servico de Vigilancia Epidemiologica e de Zoonoses do Guaruja	Instituto Adolfo Lutz Strategic Laboratory	Claudio Tavares Sacchi, Karoline Rodrigues Campos, Ariadne Ferreira Amarante, Marlon Benedito Nascimento Santos, Alex Domingos Reis, Adriano Abbud, Adriana Bugno
EPI_ISL_14624915	SMS Aruja	Instituto Adolfo Lutz Strategic Laboratory	Claudio Tavares Sacchi, Karoline Rodrigues Campos, Ariadne Ferreira Amarante, Marlon Benedito Nascimento Santos, Alex Domingos Reis, Adriano Abbud, Adriana Bugno
EPI_ISL_14625156	Secretaria Municipal de Saude de Suzano	Instituto Adolfo Lutz Strategic Laboratory	Claudio Tavares Sacchi, Karoline Rodrigues Campos, Ariadne Ferreira Amarante, Marlon Benedito Nascimento Santos, Alex Domingos Reis, Adriano Abbud, Adriana Bugno
EPI_ISL_14625157	PSF Vila Nossa Senhora de Fatima Fartura	Instituto Adolfo Lutz Strategic Laboratory	Claudio Tavares Sacchi, Karoline Rodrigues Campos, Ariadne Ferreira Amarante, Marlon Benedito Nascimento Santos, Alex Domingos Reis, Adriano Abbud, Adriana Bugno
EPI_ISL_14625190	Ambulatorio de Atendimentode DST de Guariba	Instituto Adolfo Lutz Strategic Laboratory	Claudio Tavares Sacchi, Karoline Rodrigues Campos, Ariadne Ferreira Amarante, Marlon Benedito Nascimento Santos, Alex Domingos Reis, Adriano Abbud, Adriana Bugno
EPI_ISL_14625230	UBS Centro Clair Aparecida Pavan	Instituto Adolfo Lutz Strategic Laboratory	Claudio Tavares Sacchi, Karoline Rodrigues Campos, Ariadne Ferreira Amarante, Marlon Benedito Nascimento Santos, Alex Domingos Reis, Adriano Abbud, Adriana Bugno
EPI_ISL_14625256	UMS Campina do Siqueira	Instituto Adolfo Lutz Strategic Laboratory	Claudio Tavares Sacchi, Karoline Rodrigues Campos, Ariadne Ferreira Amarante, Marlon Benedito Nascimento Santos, Alex Domingos Reis, Adriano Abbud, Adriana Bugno
EPI_ISL_14625282	Hospital Edmundo Vasconcelos	Instituto Adolfo Lutz Strategic Laboratory	Claudio Tavares Sacchi, Karoline Rodrigues Campos, Ariadne Ferreira Amarante, Marlon Benedito Nascimento Santos, Alex Domingos Reis, Adriano Abbud, Adriana Bugno
EPI_ISL_14664595, EPI_ISL_14665380, EPI_ISL_14665384, EPI_ISL_14665389, EPI_ISL_14665390, EPI_ISL_14665391, EPI_ISL_14665393	CT Department of Public Health	CT Department of Public Health	Claire Pearson, Tu N. Nguyen, Kutluhan Incekara
EPI_ISL_14666780	Public Health Authority of the Slovak Republic	Laboratory of Genomics and Bioinformatics, Comenius University Science Park	Tomáš Szemes, Edita Staroňová, Elena Tichá, Lucia Ševčíková, Terézia Vrabľová, Tatiana Sedláčková, Miroslav Böhmer, Jaroslav Budiš, Pavol Mišenko
EPI_ISL_14676265	Centro de Desenvolvimento Científico e Tecnológico (CDCT)/CEVS/SES-RS	Centro de Desenvolvimento Científico e Tecnológico (CDCT)/CEVS/SES-RS	Richard Steiner Salvato, Regina Bones Barcellos, Fernanda Marques Godinho
EPI_ISL_14699907, EPI_ISL_14699908, EPI_ISL_14699909, EPI_ISL_14699910	Centers for Disease Control & Prevention (CDC), Division of High Consequence Pathogens and Pathology (DHCPP-PRB)	Centers for Disease Control & Prevention (CDC), Division of High Consequence Pathogens and Pathology (DHCPP-PRB)	Gigante,C.M., Lee,P., Zhao,H., Batra,D., Hetrick,E.E., Howard,D.T., Kovar,L., Seabolt,M.H., Weigand,M.R., Burroughs,M.S., Lee,J., Wilkins,K., McCollum,A., Hutson,C., Davidson,W., Rao,A., Mendoza,R. and Li,Y.
EPI_ISL_14699911, EPI_ISL_14699912, EPI_ISL_14699913, EPI_ISL_14699914, EPI_ISL_14699915	Centers for Disease Control & Prevention (CDC), Division of High Consequence Pathogens and Pathology (DHCPP-PRB)	Centers for Disease Control & Prevention (CDC), Division of High Consequence Pathogens and Pathology (DHCPP-PRB)	Gigante,C.M., Ghinai,I., Zhao,H., Batra,D., Hetrick,E.E., Howard,D.T., Kovar,L., Seabolt,M.H., Weigand,M.R., Burroughs,M.S., Lee,J., Wilkins,K., McCollum,A., Hutson,C., Davidson,W., Rao,A., Kerins,J. and Li,Y.
EPI_ISL_14699916	Centers for Disease Control & Prevention (CDC), Division of High Consequence Pathogens and Pathology (DHCPP-PRB)	Centers for Disease Control & Prevention (CDC), Division of High Consequence Pathogens and Pathology (DHCPP-PRB)	Gigante,C.M., Kubin,G., Zhao,H., Batra,D., Hetrick,E.E., Howard,D.T., Kovar,L., Seabolt,M.H., Weigand,M.R., Burroughs,M.S., Lee,J., Wilkins,K., McCollum,A., Hutson,C., Davidson,W., Rao,A., White,S.L. and Li,Y.
EPI_ISL_14699917	Centers for Disease Control & Prevention (CDC), Division of High Consequence Pathogens and Pathology (DHCPP-PRB)	Centers for Disease Control & Prevention (CDC), Division of High Consequence Pathogens and Pathology (DHCPP-PRB)	Gigante,C.M., Winter,K., Zhao,H., Batra,D., Hetrick,E.E., Howard,D.T., Kovar,L., Seabolt,M.H., Weigand,M.R., Burroughs,M.S., Lee,J., Wilkins,K., McCollum,A., Hutson,C., Davidson,W., Rao,A., Arora,V. and Li,Y.
EPI_ISL_14699918	Centers for Disease Control & Prevention (CDC), Division of High Consequence Pathogens and Pathology (DHCPP-PRB)	Centers for Disease Control & Prevention (CDC), Division of High Consequence Pathogens and Pathology (DHCPP-PRB)	Gigante,C.M., Hughes,S., Zhao,H., Batra,D., Hetrick,E.E., Howard,D.T., Kovar,L., Seabolt,M.H., Weigand,M.R., Burroughs,M.S., Lee,J., Wilkins,K., McCollum,A., Hutson,C., Davidson,W., Rao,A., Baumgartner,J. and Li,Y.
EPI_ISL_14699919	Centers for Disease Control & Prevention (CDC), Division of High Consequence Pathogens and Pathology (DHCPP-PRB)	Centers for Disease Control & Prevention (CDC), Division of High Consequence Pathogens and Pathology (DHCPP-PRB)	Gigante,C.M., Ghinai,I., Zhao,H., Batra,D., Hetrick,E.E., Howard,D.T., Kovar,L., Seabolt,M.H., Weigand,M.R., Burroughs,M.S., Lee,J., Wilkins,K., McCollum,A., Hutson,C., Davidson,W., Rao,A., Kerins,J. and Li,Y.
EPI_ISL_14699920, EPI_ISL_14699921	Centers for Disease Control & Prevention (CDC), Division of High Consequence Pathogens and Pathology (DHCPP-PRB)	Centers for Disease Control & Prevention (CDC), Division of High Consequence Pathogens and Pathology (DHCPP-PRB)	Gigante,C.M., Iwen,P.C., Zhao,H., Batra,D., Hetrick,E.E., Howard,D.T., Kovar,L., Seabolt,M.H., Weigand,M.R., Burroughs,M.S., Lee,J., Wilkins,K., McCollum,A., Hutson,C., Davidson,W., Rao,A., Donahue,M. and Li,Y.
EPI_ISL_14699922, EPI_ISL_14699923	Centers for Disease Control & Prevention (CDC), Division of High Consequence Pathogens and Pathology (DHCPP-PRB)	Centers for Disease Control & Prevention (CDC), Division of High Consequence Pathogens and Pathology (DHCPP-PRB)	Gigante,C.M., Hughes,S., Zhao,H., Batra,D., Hetrick,E.E., Howard,D.T., Kovar,L., Seabolt,M.H., Weigand,M.R., Burroughs,M.S., Lee,J., Wilkins,K., McCollum,A., Hutson,C., Davidson,W., Rao,A., Baumgartner,J. and Li,Y.
EPI_ISL_14699924	Centers for Disease Control & Prevention (CDC), Division of High Consequence Pathogens and Pathology (DHCPP-PRB)	Centers for Disease Control & Prevention (CDC), Division of High Consequence Pathogens and Pathology (DHCPP-PRB)	Gigante,C.M., Lee,B., Zhao,H., Batra,D., Hetrick,E.E., Howard,D.T., Kovar,L., Seabolt,M.H., Weigand,M.R., Burroughs,M.S., Lee,J., Wilkins,K., McCollum,A., Hutson,C., Davidson,W., Rao,A., Salehi,E. and Li,Y.
EPI_ISL_14699925	Centers for Disease Control & Prevention (CDC), Division of High Consequence Pathogens and Pathology (DHCPP-PRB)	Centers for Disease Control & Prevention (CDC), Division of High Consequence Pathogens and Pathology (DHCPP-PRB)	Gigante,C.M., Hughes,S., Zhao,H., Batra,D., Hetrick,E.E., Howard,D.T., Kovar,L., Seabolt,M.H., Weigand,M.R., Burroughs,M.S., Lee,J., Wilkins,K., McCollum,A., Hutson,C., Davidson,W., Rao,A., Baumgartner,J. and Li,Y.
EPI_ISL_14699926	Centers for Disease Control & Prevention (CDC), Division of High Consequence Pathogens and Pathology (DHCPP-PRB)	Centers for Disease Control & Prevention (CDC), Division of High Consequence Pathogens and Pathology (DHCPP-PRB)	Gigante,C.M., Griffin-Thomas,L., Zhao,H., Batra,D., Hetrick,E.E., Howard,D.T., Kovar,L., Seabolt,M.H., Weigand,M.R., Burroughs,M.S., Lee,J., Wilkins,K., McCollum,A., Hutson,C., Davidson,W., Rao,A., Crain,J. and Li,Y.
EPI_ISL_14699927, EPI_ISL_14699928, EPI_ISL_14699929, EPI_ISL_14699930, EPI_ISL_14699931, EPI_ISL_14699932, EPI_ISL_14699933, EPI_ISL_14699934, EPI_ISL_14699935, EPI_ISL_14699936, EPI_ISL_14699937, EPI_ISL_14699938, EPI_ISL_14699939, EPI_ISL_14699940, EPI_ISL_14699941, EPI_ISL_14699942, EPI_ISL_14699943, EPI_ISL_14699944, EPI_ISL_14699945, EPI_ISL_14699946, EPI_ISL_14699947, EPI_ISL_14699948, EPI_ISL_14699949, EPI_ISL_14699950, EPI_ISL_14699951, EPI_ISL_14699952, EPI_ISL_14699953, EPI_ISL_14699954, EPI_ISL_14699955, EPI_ISL_14699956, EPI_ISL_14699957, EPI_ISL_14699958			
see above	Laboratory Medicine, UW Virology	Laboratory Medicine, UW Virology	Sereewit,J., Xie,H., Roychoudhury,P. and Greninger,A.L.
EPI_ISL_14707250	Charité Universitätsmedizin Berlin, Institut für Virologie, Charitéplatz 1, 10117 Berlin, Germany	Charité Universitätsmedizin Berlin, Institut für Virologie, Charitéplatz 1, 10117 Berlin, Germany	Julia Schneider, Victor M Corman, Terry C Jones, Christian Drosten
EPI_ISL_14721255, EPI_ISL_14721256, EPI_ISL_14721259, EPI_ISL_14721262, EPI_ISL_14721263, EPI_ISL_14721264, EPI_ISL_14721265	National Public Health Laboratory, National Centre for Infectious Diseases	National Public Health Laboratory, National Centre for Infectious Diseases	Yichen Ding, Benny Yeo, Daniel Lim, Zhenyang Zhou, Royce Ang, Samuel Loo, Lin Cui, Raymond Tzer Pin Lin
EPI_ISL_14736400, EPI_ISL_14736401, EPI_ISL_14736402, EPI_ISL_14736403, EPI_ISL_14736404, EPI_ISL_14736405, EPI_ISL_14736406, EPI_ISL_14736408, EPI_ISL_14736409	California Department of Public Health	California Department of Public Health	Viral and Rickettsial Disease Laboratory
EPI_ISL_14752090, EPI_ISL_14752091,	Environmental, Agricultural, and Occupational Health,	Environmental, Agricultural, and Occupational Health,	Tegomoh,B., Cross,S.T., Chapman,R.C., Bernhard,K., McCutchen,E.L., Fauver,J.R., Pratt,C.B., Warden,D.E., Iwen,P.C., Donahue,M. and Wiley,M.R.

EPI_ISL_14752093, EPI_ISL_14752094, EPI_ISL_14752096	University of Nebraska Medical Center	University of Nebraska Medical Center	
EPI_ISL_14752098, EPI_ISL_14752100, EPI_ISL_14752102, EPI_ISL_14752104, EPI_ISL_14752106, EPI_ISL_14752108, EPI_ISL_14752109, EPI_ISL_14752111, EPI_ISL_14752115, EPI_ISL_14752117, EPI_ISL_14752119, EPI_ISL_14752120, EPI_ISL_14752122, EPI_ISL_14752124, EPI_ISL_14752126, EPI_ISL_14752127, EPI_ISL_14752128, EPI_ISL_14752129, EPI_ISL_14752130, EPI_ISL_14752131, EPI_ISL_14752132, EPI_ISL_14752133, EPI_ISL_14752135, EPI_ISL_14752137, EPI_ISL_14752139, EPI_ISL_14752141, EPI_ISL_14752143, EPI_ISL_14752145, EPI_ISL_14752146, EPI_ISL_14752148, EPI_ISL_14752150, EPI_ISL_14752152, EPI_ISL_14752154, EPI_ISL_14752156, EPI_ISL_14752157, EPI_ISL_14752159, EPI_ISL_14752161, EPI_ISL_14752163, EPI_ISL_14752165, EPI_ISL_14752167, EPI_ISL_14752169, EPI_ISL_14752171, EPI_ISL_14752173, EPI_ISL_14752175, EPI_ISL_14752177, EPI_ISL_14752178, EPI_ISL_14752180, EPI_ISL_14752182, EPI_ISL_14752184, EPI_ISL_14752186, EPI_ISL_14752188, EPI_ISL_14752189, EPI_ISL_14752191, EPI_ISL_14752193, EPI_ISL_14752195, EPI_ISL_14752199, EPI_ISL_14752202, EPI_ISL_14752204, EPI_ISL_14752206, EPI_ISL_14752208, EPI_ISL_14752211, EPI_ISL_14752212, EPI_ISL_14752213, EPI_ISL_14752214, EPI_ISL_14752215, EPI_ISL_14752216, EPI_ISL_14752217, EPI_ISL_14752218, EPI_ISL_14752219, EPI_ISL_14752221, EPI_ISL_14752222, EPI_ISL_14752224, EPI_ISL_14752225, EPI_ISL_14752229, EPI_ISL_14752231, EPI_ISL_14752233, EPI_ISL_14752236, EPI_ISL_14752242, EPI_ISL_14752246, EPI_ISL_14752248, EPI_ISL_14752249, EPI_ISL_14752251, EPI_ISL_14752253			
see above	Department of Infectious Diseases, National Institute of Health Doutor Ricardo Jorge (INSA)	Department of Infectious Diseases, National Institute of Health Doutor Ricardo Jorge (INSA)	Isidro,J., Borges,V., Pinto,M., Sobral,D., Santos,J., Nunes,A., Mixao,V., Ferreira,R., Santos,D., Duarte,S., Vieira,L., Borrego,M.J., Nuncio,S., Lopes de Carvalho,I., Pelerito,A., Cordeiro,R. and Gomes,J.P.
EPI_ISL_14752257, EPI_ISL_14752259, EPI_ISL_14752260, EPI_ISL_14752261, EPI_ISL_14752262, EPI_ISL_14752263	Laboratory Medicine, UW Virology	Laboratory Medicine, UW Virology	Sereewit,J., Xie,H., Roychoudhury,P. and Greninger,A.L.
EPI_ISL_14752264, EPI_ISL_14752265, EPI_ISL_14752267, EPI_ISL_14752269, EPI_ISL_14752270, EPI_ISL_14752272, EPI_ISL_14752274, EPI_ISL_14752276, EPI_ISL_14752278, EPI_ISL_14752280, EPI_ISL_14752282			
see above	Centre for Biological Threats, Highly Pathogenic Viruses, Robert Koch Institute	Centre for Biological Threats, Highly Pathogenic Viruses, Robert Koch Institute	Brinkmann,A., Kohl,C., Uddin,S., Pape,K., Schrick,L., Michel,J., Schaade,L. and Nitsche,A.
EPI_ISL_14752284	Research and Evaluation, UKHSA	Research and Evaluation, UKHSA	Grove,N., Osman,K.L., Lewandowski,K.S., Carter,D.P., Pullan,S.T., Myers,R., Vipond,R. and Chand,M.
EPI_ISL_14752286, EPI_ISL_14752288, EPI_ISL_14752290, EPI_ISL_14752291	Research and Evaluation, UKHSA	Research and Evaluation, UKHSA	Groves,N., Osman,K.L., Lewandowski,K.S., Carter,D.P., Pullan,S.T., Myers,R., Vipond,R. and Chand,M.
EPI_ISL_14752293	Medical Microbiology & Infection Prevention, Amsterdam Medical Centres location AMC	Medical Microbiology & Infection Prevention, Amsterdam Medical Centres location AMC	Welkers,M., Jonges,M., de Regt,M., Ooijevaar,R. and Wagemakers,A.
EPI_ISL_14772317	Policlinica Jacare Wilson Federzoni Cabreuva	Instituto Adolfo Lutz Strategic Laboratory	Claudio Tavares Sacchi, Karoline Rodrigues Campos, Ariadne Ferreira Amarante, Marlon Benedito Nascimento Santos, Alex Domingos Reis, Adriano Abbud, Adriana Bugno
EPI_ISL_14772318	Secretaria Municipal de Saude de Sertaozinho	Instituto Adolfo Lutz Strategic Laboratory	Claudio Tavares Sacchi, Karoline Rodrigues Campos, Ariadne Ferreira Amarante, Marlon Benedito Nascimento Santos, Alex Domingos Reis, Adriano Abbud, Adriana Bugno
EPI_ISL_14772912	USF Jardim Oratorio	Instituto Adolfo Lutz Strategic Laboratory	Claudio Tavares Sacchi, Karoline Rodrigues Campos, Ariadne Ferreira Amarante, Marlon Benedito Nascimento Santos, Alex Domingos Reis, Adriano Abbud, Adriana Bugno
EPI_ISL_14772913	Vigilancia Epidemiologica Jardinopolis - SP	Instituto Adolfo Lutz Strategic Laboratory	Claudio Tavares Sacchi, Karoline Rodrigues Campos, Ariadne Ferreira Amarante, Marlon Benedito Nascimento Santos, Alex Domingos Reis, Adriano Abbud, Adriana Bugno
EPI_ISL_14772914	Pronto Atendimento Infantil e entral de Quimioterapia SJrpreto	Instituto Adolfo Lutz Strategic Laboratory	Claudio Tavares Sacchi, Karoline Rodrigues Campos, Ariadne Ferreira Amarante, Marlon Benedito Nascimento Santos, Alex Domingos Reis, Adriano Abbud, Adriana Bugno
EPI_ISL_14773001	CEDIC CTA	Instituto Adolfo Lutz Strategic Laboratory	Claudio Tavares Sacchi, Karoline Rodrigues Campos, Ariadne Ferreira Amarante, Marlon Benedito Nascimento Santos, Alex Domingos Reis, Adriano Abbud, Adriana Bugno
EPI_ISL_14783237	Sicilian Regional Laboratory - AOUP "P. Giaccone" - University of Palermo	Sicilian Regional Laboratory - AOUP "P. Giaccone" - University of Palermo	Fabio Tramuto, Carmelo Massimo Maida, Giulia Randazzo, Valeria Guzzetta, Walter Mazzucco, Giorgio Graziano, Vincenzo Restivo, Claudio Costantino, Francesco Vitale
EPI_ISL_14786290	IRCCS Sacro Cuore Don Calabria Hospital, Department of Infectious, Tropical Diseases & Microbiology	Department of Infectious, Tropical Diseases & Microbiology,IRCCS Sacro Cuore Don Calabria Hospital	Michela Deiana, Antonio Mori, Concetta Castilletti, Chiara Piubelli, Denise Lavezzari, Elena Pomari
EPI_ISL_14786346	IRCCS Sacro Cuore Don Calabria Hospital, Department of Infectious, Tropical Diseases & Microbiology	IRCCS Sacro Cuore Don Calabria Hospital, Department of Infectious, Tropical Diseases & Microbiology	Michela Deiana, Antonio Mori, Concetta Castilletti, Chiara Piubelli, Denise Lavezzari, Elena Pomari
EPI_ISL_14793992, EPI_ISL_14795058, EPI_ISL_14795084, EPI_ISL_14795085, EPI_ISL_14795259	Erasmus Medical Center Department of Virology	Erasmus Medical Center Department of Virology	Bas Oude Munnink, Leonard Schuele, Marjan Boter, Babette Weller, Babs Verstrepen, Richard Molenkamp, Janette Rahamat-Langendoen, Reina Sikkema, Marion Koopmans
EPI_ISL_14804616, EPI_ISL_14804617, EPI_ISL_14804618, EPI_ISL_14804619, EPI_ISL_14804620, EPI_ISL_14804621, EPI_ISL_14804622, EPI_ISL_14804623, EPI_ISL_14804624, EPI_ISL_14804625, EPI_ISL_14804626, EPI_ISL_14804627, EPI_ISL_14804628, EPI_ISL_14804629, EPI_ISL_14804630, EPI_ISL_14804631, EPI_ISL_14804632, EPI_ISL_14804633, EPI_ISL_14804634, EPI_ISL_14804635, EPI_ISL_14804636			
see above	Centre for Biological Threats, Highly Pathogenic Viruses, Robert Koch Institute	Centre for Biological Threats, Highly Pathogenic Viruses, Robert Koch Institute	Brinkmann,A., Kohl,C., Pape,K., Uddin,S., Schrick,L., Michel,J., Schaade,L., Nitsche,A.
EPI_ISL_14804638, EPI_ISL_14804639, EPI_ISL_14804640, EPI_ISL_14804641, EPI_ISL_14804642, EPI_ISL_14804643, EPI_ISL_14804644, EPI_ISL_14804645, EPI_ISL_14804646, EPI_ISL_14804647	Nebraska Public Health Laboratory	University of Nebraska Medical Center, Oklahoma Pathogen Genomics Consortium	Chapman,R.C., Bernhard,K., McCutchen,E.L., Fauver,J.R., O'Dell,J.X., Mannell,M., Wiley,M.R., Cross,S.T.
EPI_ISL_14809096	AMA Capao Redondo	Instituto Adolfo Lutz Strategic Laboratory	Claudio Tavares Sacchi, Karoline Rodrigues Campos, Ariadne Ferreira Amarante, Marlon Benedito Nascimento Santos, Alex Domingos Reis, Adriano Abbud, Adriana Bugno
EPI_ISL_14809097	Pronto Socorro Municipal de Taubate	Instituto Adolfo Lutz Strategic Laboratory	Claudio Tavares Sacchi, Karoline Rodrigues Campos, Ariadne Ferreira Amarante, Marlon Benedito Nascimento Santos, Alex Domingos Reis, Adriano Abbud, Adriana Bugno
EPI_ISL_14809098	Laboratorio Municipal de Piracicaba	Instituto Adolfo Lutz Strategic Laboratory	Claudio Tavares Sacchi, Karoline Rodrigues Campos, Ariadne Ferreira Amarante, Marlon Benedito Nascimento Santos, Alex Domingos Reis, Adriano Abbud, Adriana Bugno
EPI_ISL_14809099	Centro de Saude Gabriel de Lara	Instituto Adolfo Lutz Strategic Laboratory	Claudio Tavares Sacchi, Karoline Rodrigues Campos, Ariadne Ferreira Amarante, Marlon Benedito Nascimento Santos, Alex Domingos Reis, Adriano Abbud, Adriana Bugno
EPI_ISL_14809100	Secretaria Municipal da Saude de Joanopolis	Instituto Adolfo Lutz Strategic Laboratory	Claudio Tavares Sacchi, Karoline Rodrigues Campos, Ariadne Ferreira Amarante, Marlon Benedito Nascimento Santos, Alex Domingos Reis, Adriano Abbud, Adriana Bugno
EPI_ISL_14810370, EPI_ISL_14810404, EPI_ISL_14810405, EPI_ISL_14810406, EPI_ISL_14810407	Erasmus Medical Center Department of Virology	Erasmus Medical Center Department of Virology	Leonard Schuele, Bas Oude Munnink, Marjan Boter, Babette Weller, Babs Verstrepen, Richard Molenkamp, Janette Rahamat-Langendoen, Reina Sikkema, Marion Koopmans
EPI_ISL_14810460	Rapid Respose Lab, Pasteur Institute of Iran	Rapid Respose Lab, Pasteur Institute of Iran	Mahsa Tavakoli, Zahra Fereydouni, Zahra Ahmadi, Amir Hesam Nemati, Setareh Kashanian, Zahra Hosseini, Laya Farhan Asadi, Jahangir Rezaie, Parastoo Yektay Sanati, Farideh Niknam Oskouei, Tahmineh Jalali, Mohammad Hassan Pouriayevali, Keyhan Azadmanesh, Arash Arashkia, Ahmad Adeli, Ali Maleki, Mahdi Rohani, Mostafa Salehi-Vaziri
EPI_ISL_14818584, EPI_ISL_14818585, EPI_ISL_14818586, EPI_ISL_14818587, EPI_ISL_14818588, EPI_ISL_14818589, EPI_ISL_14818590, EPI_ISL_14818591, EPI_ISL_14818592, EPI_ISL_14818593, EPI_ISL_14818594, EPI_ISL_14818595, EPI_ISL_14818596, EPI_ISL_14818597, EPI_ISL_14818598, EPI_ISL_14818599, EPI_ISL_14818600, EPI_ISL_14818601, EPI_ISL_14818602, EPI_ISL_14818603, EPI_ISL_14818605, EPI_ISL_14818606, EPI_ISL_14818607, EPI_ISL_14818608, EPI_ISL_14818609, EPI_ISL_14818610, EPI_ISL_14818611, EPI_ISL_14818614, EPI_ISL_14818615, EPI_ISL_14818616, EPI_ISL_14818617			
see above	Los Angeles County Public Health Laboratories	Los Angeles County Public Health Laboratories	P. Hemarajata et al.
EPI_ISL_14818783, EPI_ISL_14818784, EPI_ISL_14818785, EPI_ISL_14818786, EPI_ISL_14818787, EPI_ISL_14818788, EPI_ISL_14818789, EPI_ISL_14818790, EPI_ISL_14818791, EPI_ISL_14818792, EPI_ISL_14818793, EPI_ISL_14818794, EPI_ISL_14818795, EPI_ISL_14818796, EPI_ISL_14818797, EPI_ISL_14818798, EPI_ISL_14818799, EPI_ISL_14818800, EPI_ISL_14818801, EPI_ISL_14818802, EPI_ISL_14818803, EPI_ISL_14818804, EPI_ISL_14818805, EPI_ISL_14818806, EPI_ISL_14818807, EPI_ISL_14818808, EPI_ISL_14818809, EPI_ISL_14818810, EPI_ISL_14818811, EPI_ISL_14818812, EPI_ISL_14818813, EPI_ISL_14818814, EPI_ISL_14818815, EPI_ISL_14818817, EPI_ISL_14818818, EPI_ISL_14818819, EPI_ISL_14818820, EPI_ISL_14818821, EPI_ISL_14818822			
see above	Laboratorio de Referencia Nacional de Virus Inmunoprevenibles. Centro Nacional de Salud Publica. Instituto Nacional de Salud.	Laboratorio de Referencia Nacional de Virus Inmunoprevenibles. Centro Nacional de Salud Publica. Instituto Nacional de Salud.	Carlos Padilla Rojas, Veronica Hurtado Vela, Iris Silva Molina, Luren Sevilla Castañeda, Victor Jimenez Vasquez, Luis Barcena Flores, Alicia Nuñez Llanos, Kelly Izarra Rojas, Karla Vasquez Cajachahua, Estela Huaman Angeles, Jorge Giraldo Chavez, Lilian Huarca Balbin, Maria Sandra Villar Saavedra, Henri Bailon Calderon, Lely Solari Zerpa, Gloria Arotinco Garayar. Equipo de vigilancia genomica del Instituto Nacional de Salud.
EPI_ISL_14835894, EPI_ISL_14835895, EPI_ISL_14835897	Quest Diagnostics Nichols Institute	Los Angeles County Public Health Laboratories	P. Hemarajata et al.
EPI_ISL_14838586, EPI_ISL_14838587, EPI_ISL_14838588, EPI_ISL_14838589	Pathogen Genomics Lab, National Institute for Biomedical Research (INRB)	Pathogen Genomics Lab, National Institute for Biomedical Research (INRB)	Placide Mbala-Kingebeni, Eddy Kinganda-Lusamaki, Adrienne Amuri-Aziza, Elisabeth Pukuta, Catherine Pratt, Nicolas Fernandez, Emmanuel Lokilo Lofiko, Gradi Luakanda Ndelemo, Francisca Muyembe Mawete, Jean Claude Makangara Cigolo, Elisabeth Muyamuna, Raphaël Lumembe Numbi, Gabriel Kabamba Lungenyi, Prince Akil Bandali, Pauline Musuamba Kayembe, Rilia Ola Mpumbe, Emile Malembi, Emmanuel Hasivirwe Vakaniaki, Andrew Rambaut, Nick Loman, Kristian Andersen, Michael Wiley, Ahidjo Ayouba, Steve Ahuka-Mundeke, Martine Peeters, Eric Delaporte, Jean-Jacques Muyembe Tamfum
EPI_ISL_14842151	Division of High Consequence Pathogens and Pathology (DHCPP)-PRB, CDC	Division of High Consequence Pathogens and Pathology (DHCPP)-PRB, CDC	Gigante,C.M., Hauser,J.R., Zhao,H., Batra,D., Hetrick,E.E., Howard,D.T., Kovar,L., Seabolt,M.H., Weigand,M.R., Burroughs,M., Lee,J., Wilkins,K., McCollum,A., Hutson,C., Davidson,W., Rao,A., Mangla,A. and Li,Y.
EPI_ISL_14842152	Division of High Consequence Pathogens and Pathology (DHCPP)-PRB, CDC	Division of High Consequence Pathogens and Pathology (DHCPP)-PRB, CDC	Gigante,C.M., Pavlick,J., Zhao,H., Batra,D., Hetrick,E.E., Howard,D.T., Kovar,L., Seabolt,M.H., Weigand,M.R., Burroughs,M., Lee,J., Wilkins,K., McCollum,A., Hutson,C., Davidson,W., Rao,A., Parrott,T. and Li,Y.
EPI_ISL_14842153, EPI_ISL_14842154	Division of High Consequence Pathogens and Pathology (DHCPP)-PRB, CDC	Division of High Consequence Pathogens and Pathology (DHCPP)-PRB, CDC	Gigante,C.M., Hauser,J.R., Zhao,H., Batra,D., Hetrick,E.E., Howard,D.T., Kovar,L., Seabolt,M.H., Weigand,M.R., Burroughs,M., Lee,J., Wilkins,K., McCollum,A., Hutson,C., Davidson,W., Rao,A., Mangla,A. and Li,Y.
EPI_ISL_14842155, EPI_ISL_14842156, EPI_ISL_14842157	Division of High Consequence Pathogens and Pathology (DHCPP)-PRB, CDC	Division of High Consequence Pathogens and Pathology (DHCPP)-PRB, CDC	Gigante,C.M., Pavlick,J., Zhao,H., Batra,D., Hetrick,E.E., Howard,D.T., Kovar,L., Seabolt,M.H., Weigand,M.R., Burroughs,M., Lee,J., Wilkins,K., McCollum,A., Hutson,C., Davidson,W., Rao,A., Parrott,T. and Li,Y.
EPI_ISL_14842158	Division of High Consequence Pathogens and Pathology (DHCPP)-PRB, CDC	Division of High Consequence Pathogens and Pathology (DHCPP)-PRB, CDC	Gigante,C.M., Hughes,S., Zhao,H., Batra,D., Hetrick,E.E., Howard,D.T., Kovar,L., Seabolt,M.H., Weigand,M.R., Burroughs,M., Lee,J., Wilkins,K., McCollum,A., Hutson,C., Davidson,W., Rao,A., Baumgartner,J. and Li,Y.
EPI_ISL_14842159	Division of High Consequence Pathogens and Pathology (DHCPP)-PRB, CDC	Division of High Consequence Pathogens and Pathology (DHCPP)-PRB, CDC	Gigante,C.M., Johnson,S., Zhao,H., Batra,D., Hetrick,E.E., Howard,D.T., Kovar,L., Seabolt,M.H., Weigand,M.R., Burroughs,M., Lee,J., Wilkins,K., McCollum,A., Hutson,C., Davidson,W., Rao,A., Riner,D. and Li,Y.
EPI_ISL_14842160	Division of High Consequence Pathogens and Pathology (DHCPP)-PRB, CDC	Division of High Consequence Pathogens and Pathology (DHCPP)-PRB, CDC	Gigante,C.M., Manuzak,A., Zhao,H., Batra,D., Hetrick,E.E., Howard,D.T., Kovar,L., Seabolt,M.H., Weigand,M.R., Burroughs,M., Lee,J., Wilkins,K., McCollum,A., Hutson,C., Davidson,W., Rao,A., Gose,R. and Li,Y.
EPI_ISL_14842161, EPI_ISL_14842162	Division of High Consequence Pathogens and Pathology (DHCPP)-PRB, CDC	Division of High Consequence Pathogens and Pathology (DHCPP)-PRB, CDC	Gigante,C.M., Kubin,G., Zhao,H., Batra,D., Hetrick,E.E., Howard,D.T., Kovar,L., Seabolt,M.H., Weigand,M.R., Burroughs,M., Lee,J., Wilkins,K., McCollum,A., Hutson,C., Davidson,W., Rao,A., White,S.L. and Li,Y.
EPI_ISL_14842163	Division of High Consequence Pathogens and Pathology (DHCPP)-PRB, CDC	Division of High Consequence Pathogens and Pathology (DHCPP)-PRB, CDC	Gigante,C.M., Ghinai,I., Zhao,H., Batra,D., Hetrick,E.E., Howard,D.T., Kovar,L., Seabolt,M.H., Weigand,M.R., Burroughs,M., Lee,J., Wilkins,K., McCollum,A., Hutson,C., Davidson,W., Rao,A., Kerins,J. and Li,Y.
EPI_ISL_14842164, EPI_ISL_14842165	Division of High Consequence Pathogens and Pathology (DHCPP)-PRB, CDC	Division of High Consequence Pathogens and Pathology (DHCPP)-PRB, CDC	Gigante,C.M., Griffin-Thomas,L., Zhao,H., Batra,D., Hetrick,E.E., Howard,D.T., Kovar,L., Seabolt,M.H., Weigand,M.R., Burroughs,M., Lee,J., Wilkins,K., McCollum,A., Hutson,C., Davidson,W., Rao,A., Crain,J. and Li,Y.
EPI_ISL_14842166	Division of High Consequence Pathogens and Pathology (DHCPP)-PRB, CDC	Division of High Consequence Pathogens and Pathology (DHCPP)-PRB, CDC	Gigante,C.M., Xia,D., Zhao,H., Batra,D., Hetrick,E.E., Howard,D.T., Kovar,L., Seabolt,M.H., Weigand,M.R., Burroughs,M., Lee,J., Wilkins,K., McCollum,A., Hutson,C., Davidson,W., Rao,A., Plipat,N. and Li,Y.

EPI_ISL_14842167, EPI_ISL_14842168	Division of High Consequence Pathogens and Pathology (DHCPP)-PRB, CDC	Division of High Consequence Pathogens and Pathology (DHCPP)-PRB, CDC	Gigante,C.M., Griffin-Thomas,L., Zhao,H., Batra,D., Hetrick,E.E., Howard,D.T., Kovar,L., Seabolt,M.H., Weigand,M.R., Burroughs,M., Lee,J., Wilkins,K., McCollum,A., Hutson,C., Davidson,W., Rao,A., Crain,J. and Li,Y.
EPI_ISL_14863040, EPI_ISL_14863041, EPI_ISL_14863042, EPI_ISL_14863043, EPI_ISL_14863044, EPI_ISL_14863045, EPI_ISL_14863046, EPI_ISL_14863047	Molecular Epidemiology, Idaho Bureau of Laboratories	Molecular Epidemiology, Idaho Bureau of Laboratories	Ceniseros,A.
EPI_ISL_14863048	MEPHI, IHU - Mediterranee Infection	MEPHI, IHU - Mediterranee Infection	Colson,P.
EPI_ISL_14863049	Molecular Epidemiology, Idaho Bureau of Laboratories	Molecular Epidemiology, Idaho Bureau of Laboratories	Ceniseros,A.
EPI_ISL_14863050, EPI_ISL_14863051, EPI_ISL_14863052, EPI_ISL_14863053, EPI_ISL_14863054, EPI_ISL_14863055, EPI_ISL_14863057, EPI_ISL_14863058, EPI_ISL_14863059, EPI_ISL_14863060, EPI_ISL_14863061, EPI_ISL_14863062, EPI_ISL_14863064, EPI_ISL_14863065			
see above	MEPHI, IHU - Mediterranee Infection	MEPHI, IHU - Mediterranee Infection	Colson,P.
EPI_ISL_14865785	UBS J COPA	Instituto Adolfo Lutz Strategic Laboratory	Claudio Tavares Sacchi, Karoline Rodrigues Campos, Ariadne Ferreira Amarante, Marlon Benedito Nascimento Santos, Alex Domingos Reis, Adriano Abbud, Adriana Bugno
EPI_ISL_14866481	PR S da Familia Unidade de Saude Adalberto Rocha	Instituto Adolfo Lutz Strategic Laboratory	Claudio Tavares Sacchi, Karoline Rodrigues Campos, Ariadne Ferreira Amarante, Marlon Benedito Nascimento Santos, Alex Domingos Reis, Adriano Abbud, Adriana Bugno
EPI_ISL_14866751	Pronto Socorro da Vila Dirce	Instituto Adolfo Lutz Strategic Laboratory	Claudio Tavares Sacchi, Karoline Rodrigues Campos, Ariadne Ferreira Amarante, Marlon Benedito Nascimento Santos, Alex Domingos Reis, Adriano Abbud, Adriana Bugno
EPI_ISL_14866752	Secretaria Municipal de Saude Sao Carlos	Instituto Adolfo Lutz Strategic Laboratory	Claudio Tavares Sacchi, Karoline Rodrigues Campos, Ariadne Ferreira Amarante, Marlon Benedito Nascimento Santos, Alex Domingos Reis, Adriano Abbud, Adriana Bugno
EPI_ISL_14887952, EPI_ISL_14887953, EPI_ISL_14887954, EPI_ISL_14887957, EPI_ISL_14887958, EPI_ISL_14887959, EPI_ISL_14887960, EPI_ISL_14887961, EPI_ISL_14887962, EPI_ISL_14887963, EPI_ISL_14887964, EPI_ISL_14887965, EPI_ISL_14887966, EPI_ISL_14887967, EPI_ISL_14887968, EPI_ISL_14887970, EPI_ISL_14887971, EPI_ISL_14887972, EPI_ISL_14887974, EPI_ISL_14887975, EPI_ISL_14887976, EPI_ISL_14887977, EPI_ISL_14887978, EPI_ISL_14887979, EPI_ISL_14887980, EPI_ISL_14887981, EPI_ISL_14887982, EPI_ISL_14887983, EPI_ISL_14887984, EPI_ISL_14887985, EPI_ISL_14887986, EPI_ISL_14887987, EPI_ISL_14887988, EPI_ISL_14887989, EPI_ISL_14887990			
see above	Viral Genotyping Reference Laboratory, Royal Infirmary of Edinburgh	Viral Genotyping Reference Laboratory, Royal Infirmary of Edinburgh	McHugh,M.P., Maloney,D., Parker,A., Mathers,K., Dewar,R., Kenicer,J., Cotton,S., Wild,J. and Templeton,K.E.
EPI_ISL_14910863, EPI_ISL_14910864, EPI_ISL_14910865, EPI_ISL_14910866, EPI_ISL_14910867, EPI_ISL_14910868, EPI_ISL_14910869, EPI_ISL_14910870, EPI_ISL_14910871, EPI_ISL_14910872, EPI_ISL_14910873, EPI_ISL_14910874, EPI_ISL_14910875, EPI_ISL_14910876, EPI_ISL_14910877, EPI_ISL_14910878, EPI_ISL_14910879, EPI_ISL_14910880, EPI_ISL_14910881, EPI_ISL_14910882, EPI_ISL_14910883, EPI_ISL_14910884, EPI_ISL_14910885			
see above	Laboratory Medicine, UW Virology	Laboratory Medicine, UW Virology	Sereewit,J., Xie,H., Roychoudhury,P. and Greninger,A.L.
EPI_ISL_14910886	Research and Evaluation, UKHSA	Research and Evaluation, UKHSA	Burton,J., Easterbrook,L., Drinkwater,E., Groves,N., Osman,K.L., Lewandowski,K.S., Carter,D., Pullan,S.T., Myers,R., Vipond,R. and Chand,M.
EPI_ISL_14917557, EPI_ISL_14917558, EPI_ISL_14917559, EPI_ISL_14917560, EPI_ISL_14917561, EPI_ISL_14917562, EPI_ISL_14917563, EPI_ISL_14917564, EPI_ISL_14917565, EPI_ISL_14917566, EPI_ISL_14917567, EPI_ISL_14917568, EPI_ISL_14917569, EPI_ISL_14917570, EPI_ISL_14917571, EPI_ISL_14917572, EPI_ISL_14917573, EPI_ISL_14917574			
see above	Los Angeles County Public Health Laboratories	Los Angeles County Public Health Laboratories	P. Hemarajata et al.
EPI_ISL_14917575	Quest Diagnostics Nichols Institute	Los Angeles County Public Health Laboratories	P. Hemarajata et al.
EPI_ISL_14917576	Los Angeles County Public Health Laboratories	Los Angeles County Public Health Laboratories	P. Hemarajata et al.
EPI_ISL_14917577, EPI_ISL_14917578	Quest Diagnostics Nichols Institute	Los Angeles County Public Health Laboratories	P. Hemarajata et al.
EPI_ISL_14917580, EPI_ISL_14917581	Los Angeles County Public Health Laboratories	Los Angeles County Public Health Laboratories	P. Hemarajata et al.
EPI_ISL_14917582	Quest Diagnostics Nichols Institute	Los Angeles County Public Health Laboratories	P. Hemarajata et al.
EPI_ISL_14917583, EPI_ISL_14917585, EPI_ISL_14917586, EPI_ISL_14917587, EPI_ISL_14917588	Los Angeles County Public Health Laboratories	Los Angeles County Public Health Laboratories	P. Hemarajata et al.
EPI_ISL_14923900, EPI_ISL_14923901, EPI_ISL_14923902, EPI_ISL_14923903, EPI_ISL_14923904, EPI_ISL_14923905	Research and Evaluation, UKHSA	Research and Evaluation, UKHSA	Groves,N., Osman,K.L., Lewandowski,K.S., Carter,D.P., Pullan,S.T., Myers,R., Vipond,R. and Chand,M.
EPI_ISL_14934116	Medical Center of Vienna Center for Virology	Medical University of Vienna Center for Virology	Jeremy V. Camp, Monika Redlberger-Fritz, Stephan W. Aberle
EPI_ISL_14934140	Center for Virology Medical Unviersity of Vienna	Medical University of Vienna Center for Virology	Jeremy V. Camp, Monika Redlberger-Fritz, Stephan W. Aberle
EPI_ISL_14934382	Medical University of Vienna Center for Virology	Medical University of Vienna Center for Virology	Jeremy V. Camp, Monika Redlberger-Fritz, Stephan W. Aberle
EPI_ISL_14934478	Medical University of Vienna Center for Virology	Medical University of Vienna Center for Virology	Jeremy V. Camp, Monika Redlberg-Fritz, Stephan W. Aberle
EPI_ISL_14934480, EPI_ISL_14934481, EPI_ISL_14934482, EPI_ISL_14934483, EPI_ISL_14934484, EPI_ISL_14934485, EPI_ISL_14934486, EPI_ISL_14934487, EPI_ISL_14934488, EPI_ISL_14934489, EPI_ISL_14934490, EPI_ISL_14934491, EPI_ISL_14934492, EPI_ISL_14934493, EPI_ISL_14934494, EPI_ISL_14934495			
see above	Research and Evaluation, UKHSA	Research and Evaluation, UKHSA	Groves,N., Osman,K.L., Lewandowski,K.S., Carter,D.P., Pullan,S.T., Myers,R., Vipond,R. and Chand,M.
EPI_ISL_14934496, EPI_ISL_14934497, EPI_ISL_14934498, EPI_ISL_14934499, EPI_ISL_14934500, EPI_ISL_14934501, EPI_ISL_14934502, EPI_ISL_14934503, EPI_ISL_14934505, EPI_ISL_14934506, EPI_ISL_14934507, EPI_ISL_14934510, EPI_ISL_14934511, EPI_ISL_14934512, EPI_ISL_14934513, EPI_ISL_14934514, EPI_ISL_14934515, EPI_ISL_14934517, EPI_ISL_14934518, EPI_ISL_14934519, EPI_ISL_14934520, EPI_ISL_14934521, EPI_ISL_14934522, EPI_ISL_14934523, EPI_ISL_14934524, EPI_ISL_14934525, EPI_ISL_14934526, EPI_ISL_14934527, EPI_ISL_14934528, EPI_ISL_14934529, EPI_ISL_14934530, EPI_ISL_14934531, EPI_ISL_14934534, EPI_ISL_14934535, EPI_ISL_14934536, EPI_ISL_14934537, EPI_ISL_14934538, EPI_ISL_14934539, EPI_ISL_14934540, EPI_ISL_14934541, EPI_ISL_14934543, EPI_ISL_14934544, EPI_ISL_14934545, EPI_ISL_14934546, EPI_ISL_14934547, EPI_ISL_14934548, EPI_ISL_14934549, EPI_ISL_14934550, EPI_ISL_14934551, EPI_ISL_14934554, EPI_ISL_14934555, EPI_ISL_14934556, EPI_ISL_14934558, EPI_ISL_14934559, EPI_ISL_14934560, EPI_ISL_14934561, EPI_ISL_14934562, EPI_ISL_14934563, EPI_ISL_14934564, EPI_ISL_14934565, EPI_ISL_14934566, EPI_ISL_14934568, EPI_ISL_14934569, EPI_ISL_14934570, EPI_ISL_14934572, EPI_ISL_14934573, EPI_ISL_14934574, EPI_ISL_14934575, EPI_ISL_14934576, EPI_ISL_14934577, EPI_ISL_14934578, EPI_ISL_14934579, EPI_ISL_14934580, EPI_ISL_14934581, EPI_ISL_14934582, EPI_ISL_14934583, EPI_ISL_14934584, EPI_ISL_14934585, EPI_ISL_14934586, EPI_ISL_14934587, EPI_ISL_14934588, EPI_ISL_14934589, EPI_ISL_14934608, EPI_ISL_14934609, EPI_ISL_14934611, EPI_ISL_14934612, EPI_ISL_14934613, EPI_ISL_14934614, EPI_ISL_14934615, EPI_ISL_14934616, EPI_ISL_14934619			
see above	Department of Infectious Diseases, National Institute of Health Doutor Ricardo Jorge, Portugal (INSA)	Department of Infectious Diseases, National Institute of Health Doutor Ricardo Jorge, Portugal (INSA)	Isidro,J., Borges,V., Pinto,M., Sobral,D., Santos,J., Nunes,A., Mixao,V., Ferreira,R., Santos,D., Duarte,S., Vieira,L., Borrego,M.J., Nuncio,S., Lopes de Carvalho,I., Pelerito,A., Cordeiro,R. and Gomes,J.P.
EPI_ISL_14934620, EPI_ISL_14934621, EPI_ISL_14934622, EPI_ISL_14934623, EPI_ISL_14934624, EPI_ISL_14934626, EPI_ISL_14934627, EPI_ISL_14934628, EPI_ISL_14934629, EPI_ISL_14934630, EPI_ISL_14934631, EPI_ISL_14934632, EPI_ISL_14934633, EPI_ISL_14934634, EPI_ISL_14934635, EPI_ISL_14934636, EPI_ISL_14934637, EPI_ISL_14934638, EPI_ISL_14934639, EPI_ISL_14934640, EPI_ISL_14934641, EPI_ISL_14934642, EPI_ISL_14934643, EPI_ISL_14934644, EPI_ISL_14934645, EPI_ISL_14934646, EPI_ISL_14934647, EPI_ISL_14934648, EPI_ISL_14934649, EPI_ISL_14934650, EPI_ISL_14934651, EPI_ISL_14934652, EPI_ISL_14934653, EPI_ISL_14934654, EPI_ISL_14934655, EPI_ISL_14934656, EPI_ISL_14934657, EPI_ISL_14934658, EPI_ISL_14934659, EPI_ISL_14934661, EPI_ISL_14934662, EPI_ISL_14934663, EPI_ISL_14934664, EPI_ISL_14934665, EPI_ISL_14934666, EPI_ISL_14934667, EPI_ISL_14934668, EPI_ISL_14934669, EPI_ISL_14934670, EPI_ISL_14934671, EPI_ISL_14934672, EPI_ISL_14934673, EPI_ISL_14934674, EPI_ISL_14934675, EPI_ISL_14934677, EPI_ISL_14934678, EPI_ISL_14934679, EPI_ISL_14934680, EPI_ISL_14934681, EPI_ISL_14934682, EPI_ISL_14934683, EPI_ISL_14934684, EPI_ISL_14934685, EPI_ISL_14934686, EPI_ISL_14934687, EPI_ISL_14934688, EPI_ISL_14934689, EPI_ISL_14934690, EPI_ISL_14934691, EPI_ISL_14934692, EPI_ISL_14934693, EPI_ISL_14934694, EPI_ISL_14934695, EPI_ISL_14934696, EPI_ISL_14934697, EPI_ISL_14934698, EPI_ISL_14934699, EPI_ISL_14934700, EPI_ISL_14934701, EPI_ISL_14934702, EPI_ISL_14934703, EPI_ISL_14934704, EPI_ISL_14934705, EPI_ISL_14934706, EPI_ISL_14934707			
see above	Research and Evaluation, UKHSA	Research and Evaluation, UKHSA	Groves,N., Osman,K.L., Lewandowski,K.S., Carter,D.P., Pullan,S.T., Myers,R., Vipond,R. and Chand,M.
EPI_ISL_14944276, EPI_ISL_14944277, EPI_ISL_14944278, EPI_ISL_14944279, EPI_ISL_14944280, EPI_ISL_14944282, EPI_ISL_14944283, EPI_ISL_14944284, EPI_ISL_14944285, EPI_ISL_14944288, EPI_ISL_14944289, EPI_ISL_14944291, EPI_ISL_14944292, EPI_ISL_14944293, EPI_ISL_14944294			
see above	Rhode Island State Health Laboratory	Rhode Island State Health Laboratory	Kristin Carpenter-Azevedo, Sean Sierra-Patev, Richard C. Huard
EPI_ISL_14945299	Department of Microbiology, The University of Hong Kong	Department of Microbiology, The University of Hong Kong	Kelvin K.W. To, Kwok-Yung Yuen
EPI_ISL_14952916	Indian Council of Medical Research-National Institute of Virology	Indian Council of Medical Research-National Institute of Virology	Pragya Yadav, Rima Sahay, Anita Aich Shete, Sreelekshmy Mohandas, Priya Abraham
EPI_ISL_14961089, EPI_ISL_14961090	Public Health Authority of the Slovak Republic	Laboratory of Genomics and Bioinformatics, Comenius University Science Park	Tomáš Szemes, Edita Staroňová, Elena Tichá, Lucia Ševčíková, Terézia Vrabľová, Tatiana Sedláčková, Miroslav Böhmer, Jaroslav Budiš, Pavol Mišenko
EPI_ISL_14962734	Laboratory of Virology, University Hospitals of Geneva	Laboratory of Virology, University Hospitals of Geneva	Laubscher,F., Chudzinsk,V., Cordey,S., Schibler,M., Kaiser,L. and Renzoni,A.
EPI_ISL_14977306, EPI_ISL_14977307, EPI_ISL_14977308, EPI_ISL_14977309, EPI_ISL_14977310	Environmental, Agricultural, and Occupational Health, University of Nebraska Medical Center	Environmental, Agricultural, and Occupational Health, University of Nebraska Medical Center	Tegomoh,B., Cross,S.T., Chapman,R.C., Bernhard,K., McCutchen,E.L., Fauver,J.R., Pratt,C.B., Warden,D.E., Iwen,P.C., Donahue,M. and Wiley,M.R.
EPI_ISL_14980972, EPI_ISL_14981151	Kingston Health Sciences Centre	Kingston Health Sciences Centre	Calvin Sjaarda, Henry Wong, Nick Buchner, Drew Roberts, Phung Ta, Jacob Whalen, Sheri Levesque, Prameet Sheth
EPI_ISL_14994740	UBS Vila California Zeilival Bruscagin	Instituto Adolfo Lutz Strategic Laboratory	Claudio Tavares Sacchi, Karoline Rodrigues Campos, Ariadne Ferreira Amarante, Marlon Benedito Nascimento Santos, Alex Domingos Reis, Adriano Abbud, Adriana Bugno
EPI_ISL_14995206	Pronto Socorro Municiplal de Cravinhos	Instituto Adolfo Lutz Strategic Laboratory	Claudio Tavares Sacchi, Karoline Rodrigues Campos, Ariadne Ferreira Amarante, Marlon Benedito Nascimento Santos, Alex Domingos Reis, Adriano Abbud, Adriana Bugno
EPI_ISL_14995578	Hosp. Municipal de Ilhabela Gov. Mario Covas Jr.	Instituto Adolfo Lutz Strategic Laboratory	Claudio Tavares Sacchi, Karoline Rodrigues Campos, Ariadne Ferreira Amarante, Marlon Benedito Nascimento Santos, Alex Domingos Reis, Adriano Abbud, Adriana Bugno
EPI_ISL_14995579	Secretaria Municipal de Saude de Feira de Santana	Instituto Adolfo Lutz Strategic Laboratory	Claudio Tavares Sacchi, Karoline Rodrigues Campos, Ariadne Ferreira Amarante, Marlon Benedito Nascimento Santos, Alex Domingos Reis, Adriano Abbud, Adriana Bugno
EPI_ISL_14995580	UBS Alexander Fleming Simioni	Instituto Adolfo Lutz Strategic Laboratory	Claudio Tavares Sacchi, Karoline Rodrigues Campos, Ariadne Ferreira Amarante, Marlon Benedito Nascimento Santos, Alex Domingos Reis, Adriano Abbud, Adriana Bugno
EPI_ISL_14995581	Secretaria Municipal de Saude Sorocaba	Instituto Adolfo Lutz Strategic Laboratory	Claudio Tavares Sacchi, Karoline Rodrigues Campos, Ariadne Ferreira Amarante, Marlon Benedito Nascimento Santos, Alex Domingos Reis, Adriano Abbud, Adriana Bugno
EPI_ISL_14995582	Hosp. Municipla. Dr. Jose de Carvalho Florence	Instituto Adolfo Lutz Strategic Laboratory	Claudio Tavares Sacchi, Karoline Rodrigues Campos, Ariadne Ferreira Amarante, Marlon Benedito Nascimento Santos, Alex Domingos Reis, Adriano Abbud, Adriana Bugno
EPI_ISL_14995583	UBS Agua Rasa	Instituto Adolfo Lutz Strategic Laboratory	Claudio Tavares Sacchi, Karoline Rodrigues Campos, Ariadne Ferreira Amarante, Marlon Benedito Nascimento Santos, Alex Domingos Reis, Adriano Abbud, Adriana Bugno
EPI_ISL_14995585	Pronto Socorro Municipal do Promorar	Instituto Adolfo Lutz Strategic Laboratory	Claudio Tavares Sacchi, Karoline Rodrigues Campos, Ariadne Ferreira Amarante, Marlon Benedito Nascimento Santos, Alex Domingos Reis, Adriano Abbud, Adriana Bugno
EPI_ISL_14995586	UPA Centro	Instituto Adolfo Lutz Strategic Laboratory	Claudio Tavares Sacchi, Karoline Rodrigues Campos, Ariadne Ferreira Amarante, Marlon Benedito Nascimento Santos, Alex Domingos Reis, Adriano Abbud, Adriana Bugno
EPI_ISL_14995587, EPI_ISL_14995588	Centro de Saude 24 horas	Instituto Adolfo Lutz Strategic Laboratory	Claudio Tavares Sacchi, Karoline Rodrigues Campos, Ariadne Ferreira Amarante, Marlon Benedito Nascimento Santos, Alex Domingos Reis, Adriano Abbud, Adriana Bugno
EPI_ISL_14995589	Cresser Centro de Referencia da Saúde Sexual e Reprodutiva	Instituto Adolfo Lutz Strategic Laboratory	Claudio Tavares Sacchi, Karoline Rodrigues Campos, Ariadne Ferreira Amarante, Marlon Benedito Nascimento Santos, Alex Domingos Reis, Adriano Abbud, Adriana Bugno
EPI_ISL_14995590, EPI_ISL_14995591	Instituto de Infectologia Emilio Ribas	Instituto Adolfo Lutz Strategic Laboratory	Claudio Tavares Sacchi, Karoline Rodrigues Campos, Ariadne Ferreira Amarante, Marlon Benedito Nascimento Santos, Alex Domingos Reis, Adriano Abbud, Adriana Bugno
EPI_ISL_14995592	Unidade de Pronto Atendimento Cipo	Instituto Adolfo Lutz Strategic Laboratory	Claudio Tavares Sacchi, Karoline Rodrigues Campos, Ariadne Ferreira Amarante, Marlon Benedito Nascimento Santos, Alex Domingos Reis, Adriano Abbud, Adriana Bugno
EPI_ISL_14995593	SAE DST / Aids Ipiranga Jose Francisco Araujo	Instituto Adolfo Lutz Strategic Laboratory	Claudio Tavares Sacchi, Karoline Rodrigues Campos, Ariadne Ferreira Amarante, Marlon Benedito Nascimento Santos, Alex Domingos Reis, Adriano Abbud, Adriana Bugno
EPI_ISL_14995611	UBS Horto Florestal	Instituto Adolfo Lutz Strategic Laboratory	Claudio Tavares Sacchi, Karoline Rodrigues Campos, Ariadne Ferreira Amarante, Marlon Benedito Nascimento Santos, Alex Domingos Reis, Adriano Abbud, Adriana Bugno
EPI_ISL_14995612	Secretaria Minicipal de Saude de IRECE	Instituto Adolfo Lutz Strategic Laboratory	Claudio Tavares Sacchi, Karoline Rodrigues Campos, Ariadne Ferreira Amarante, Marlon Benedito Nascimento Santos, Alex Domingos Reis, Adriano Abbud, Adriana Bugno

EPI_ISL_14995619	Hosp. Tereza de Lisieux	Instituto Adolfo Lutz Strategic Laboratory	Claudio Tavares Sacchi, Karoline Rodrigues Campos, Ariadne Ferreira Amarante, Marlon Benedito Nascimento Santos, Alex Domingos Reis, Adriano Abbud, Adriana Bugno
EPI_ISL_14995622	UBS Parque Meia Lua	Instituto Adolfo Lutz Strategic Laboratory	Claudio Tavares Sacchi, Karoline Rodrigues Campos, Ariadne Ferreira Amarante, Marlon Benedito Nascimento Santos, Alex Domingos Reis, Adriano Abbud, Adriana Bugno
EPI_ISL_14995631	UPA Novo Horizonte	Instituto Adolfo Lutz Strategic Laboratory	Claudio Tavares Sacchi, Karoline Rodrigues Campos, Ariadne Ferreira Amarante, Marlon Benedito Nascimento Santos, Alex Domingos Reis, Adriano Abbud, Adriana Bugno
EPI_ISL_14995649	Instituto de Infectologia Emilio Ribas	Instituto Adolfo Lutz Strategic Laboratory	Claudio Tavares Sacchi, Karoline Rodrigues Campos, Ariadne Ferreira Amarante, Marlon Benedito Nascimento Santos, Alex Domingos Reis, Adriano Abbud, Adriana Bugno
EPI_ISL_14995652	Hosp. Dr. Osiris Florindo Coelho Ferraz de Vasconcelos	Instituto Adolfo Lutz Strategic Laboratory	Claudio Tavares Sacchi, Karoline Rodrigues Campos, Ariadne Ferreira Amarante, Marlon Benedito Nascimento Santos, Alex Domingos Reis, Adriano Abbud, Adriana Bugno
EPI_ISL_14995653	Unidade Basica de Saude Vila Cristina	Instituto Adolfo Lutz Strategic Laboratory	Claudio Tavares Sacchi, Karoline Rodrigues Campos, Ariadne Ferreira Amarante, Marlon Benedito Nascimento Santos, Alex Domingos Reis, Adriano Abbud, Adriana Bugno
EPI_ISL_14995723	Unidade Mista de Atendimento Infantil Carapicuiba	Instituto Adolfo Lutz Strategic Laboratory	Claudio Tavares Sacchi, Karoline Rodrigues Campos, Ariadne Ferreira Amarante, Marlon Benedito Nascimento Santos, Alex Domingos Reis, Adriano Abbud, Adriana Bugno
EPI_ISL_14995724	Hosp. Carlos Chagas	Instituto Adolfo Lutz Strategic Laboratory	Claudio Tavares Sacchi, Karoline Rodrigues Campos, Ariadne Ferreira Amarante, Marlon Benedito Nascimento Santos, Alex Domingos Reis, Adriano Abbud, Adriana Bugno
EPI_ISL_14997063, EPI_ISL_14997064, EPI_ISL_14997065, EPI_ISL_14997066, EPI_ISL_14997067, EPI_ISL_14997068, EPI_ISL_14997069, EPI_ISL_14997070, EPI_ISL_14997071, EPI_ISL_14997072, EPI_ISL_14997073, EPI_ISL_14997074			
see above	Laboratory Medicine, UW Virology	Laboratory Medicine, UW Virology	Sereewit,J., Xie,H., Roychoudhury,P. and Greninger,A.L.
EPI_ISL_15003284, EPI_ISL_15003285, EPI_ISL_15003286, EPI_ISL_15003287, EPI_ISL_15003288, EPI_ISL_15003289, EPI_ISL_15003291, EPI_ISL_15003293, EPI_ISL_15003294, EPI_ISL_15003295, EPI_ISL_15003296, EPI_ISL_15003297			
see above	Rhode Island State Health Laboratory	Rhode Island State Health Laboratory	Kristin Carpenter-Azevedo, Sean Sierra-Patev, Richard C. Huard
EPI_ISL_15005641	Chongqing Municipal Center for Disease Control and Prevention	Chongqing Municipal Center for Disease Control and Prevention	Sheng Ye, Yun Tang, Shuang Chen, Mingyue Wang, Zhangping Tan, Zhen Yu
EPI_ISL_15008574, EPI_ISL_15008575, EPI_ISL_15008576, EPI_ISL_15008577	Indian Council of Medical Research-National Institute of Virology	Indian Council of Medical Research-National Institute of Virology	Pragya Yadav, Rima Sahay, Anita Aich Shete, Sreelekshmy Mohandas, Priya Abraham
EPI_ISL_15016104, EPI_ISL_15016105	Centers for Disease Control & Prevention (CDC), Division of High Consequence Pathogens and Pathology (DHCPP-PRB)	Centers for Disease Control & Prevention (CDC), Division of High Consequence Pathogens and Pathology (DHCPP-PRB)	Gigante,C.M., Kubin,G., Zhao,H., Batra,D., Hetrick,E.E., Howard,D.T., Kovar,L., Seabolt,M.H., Weigand,M.R., Burroughs,M., Lee,J., Wilkins,K., McCollum,A., Hutson,C., Davidson,W., Rao,A., White,S.L. and Li,Y.
EPI_ISL_15016106	Centers for Disease Control & Prevention (CDC), Division of High Consequence Pathogens and Pathology (DHCPP-PRB)	Centers for Disease Control & Prevention (CDC), Division of High Consequence Pathogens and Pathology (DHCPP-PRB)	Gigante,C.M., Goldoft,M., Zhao,H., Batra,D., Hetrick,E.E., Howard,D.T., Kovar,L., Seabolt,M.H., Weigand,M.R., Burroughs,M., Lee,J., Wilkins,K., McCollum,A., Hutson,C., Davidson,W., Rao,A., Holshue,M. and Li,Y.
EPI_ISL_15016107	Centers for Disease Control & Prevention (CDC), Division of High Consequence Pathogens and Pathology (DHCPP-PRB)	Centers for Disease Control & Prevention (CDC), Division of High Consequence Pathogens and Pathology (DHCPP-PRB)	Gigante,C.M., Thomas,L., Zhao,H., Batra,D., Hetrick,E.E., Howard,D.T., Kovar,L., Seabolt,M.H., Weigand,M.R., Burroughs,M., Lee,J., Wilkins,K., McCollum,A., Hutson,C., Davidson,W., Rao,A., Dunn,J. and Li,Y.
EPI_ISL_15016108, EPI_ISL_15016109	Centers for Disease Control & Prevention (CDC), Division of High Consequence Pathogens and Pathology (DHCPP-PRB)	Centers for Disease Control & Prevention (CDC), Division of High Consequence Pathogens and Pathology (DHCPP-PRB)	Gigante,C.M., Buttery,E., Zhao,H., Batra,D., Hetrick,E.E., Howard,D.T., Kovar,L., Seabolt,M.H., Weigand,M.R., Burroughs,M., Lee,J., Wilkins,K., McCollum,A., Hutson,C., Davidson,W., Rao,A., Raman,D. and Li,Y.
EPI_ISL_15016110	Centers for Disease Control & Prevention (CDC), Division of High Consequence Pathogens and Pathology (DHCPP-PRB)	Centers for Disease Control & Prevention (CDC), Division of High Consequence Pathogens and Pathology (DHCPP-PRB)	Gigante,C.M., Kubin,G., Zhao,H., Batra,D., Hetrick,E.E., Howard,D.T., Kovar,L., Seabolt,M.H., Weigand,M.R., Burroughs,M., Lee,J., Wilkins,K., McCollum,A., Hutson,C., Davidson,W., Rao,A., White,S.L. and Li,Y.
EPI_ISL_15016111	Centers for Disease Control & Prevention (CDC), Division of High Consequence Pathogens and Pathology (DHCPP-PRB)	Centers for Disease Control & Prevention (CDC), Division of High Consequence Pathogens and Pathology (DHCPP-PRB)	Gigante,C.M., Thomas,L., Zhao,H., Batra,D., Hetrick,E.E., Howard,D.T., Kovar,L., Seabolt,M.H., Weigand,M.R., Burroughs,M., Lee,J., Wilkins,K., McCollum,A., Hutson,C., Davidson,W., Rao,A., Dunn,J. and Li,Y.
EPI_ISL_15016112	Centers for Disease Control & Prevention (CDC), Division of High Consequence Pathogens and Pathology (DHCPP-PRB)	Centers for Disease Control & Prevention (CDC), Division of High Consequence Pathogens and Pathology (DHCPP-PRB)	Gigante,C.M., Pettit,D., Zhao,H., Batra,D., Hetrick,E.E., Howard,D.T., Kovar,L., Seabolt,M.H., Weigand,M.R., Burroughs,M., Lee,J., Wilkins,K., McCollum,A., Hutson,C., Davidson,W., Rao,A., Deutsch-Feldman,M. and Li,Y.
EPI_ISL_15016113	Centers for Disease Control & Prevention (CDC), Division of High Consequence Pathogens and Pathology (DHCPP-PRB)	Centers for Disease Control & Prevention (CDC), Division of High Consequence Pathogens and Pathology (DHCPP-PRB)	Gigante,C.M., Ghinai,I., Zhao,H., Batra,D., Hetrick,E.E., Howard,D.T., Kovar,L., Seabolt,M.H., Weigand,M.R., Burroughs,M., Lee,J., Wilkins,K., McCollum,A., Hutson,C., Davidson,W., Rao,A., Kerins,J. and Li,Y.
EPI_ISL_15016114, EPI_ISL_15016115	Centers for Disease Control & Prevention (CDC), Division of High Consequence Pathogens and Pathology (DHCPP-PRB)	Centers for Disease Control & Prevention (CDC), Division of High Consequence Pathogens and Pathology (DHCPP-PRB)	Gigante,C.M., Thomas,L., Zhao,H., Batra,D., Hetrick,E.E., Howard,D.T., Kovar,L., Seabolt,M.H., Weigand,M.R., Burroughs,M., Lee,J., Wilkins,K., McCollum,A., Hutson,C., Davidson,W., Rao,A., Dunn,J. and Li,Y.
EPI_ISL_15016116	Centers for Disease Control & Prevention (CDC), Division of High Consequence Pathogens and Pathology (DHCPP-PRB)	Centers for Disease Control & Prevention (CDC), Division of High Consequence Pathogens and Pathology (DHCPP-PRB)	Gigante,C.M., Xia,D., Zhao,H., Batra,D., Hetrick,E.E., Howard,D.T., Kovar,L., Seabolt,M.H., Weigand,M.R., Burroughs,M., Lee,J., Wilkins,K., McCollum,A., Hutson,C., Davidson,W., Rao,A., Plipat,N. and Li,Y.
EPI_ISL_15016117	Centers for Disease Control & Prevention (CDC), Division of High Consequence Pathogens and Pathology (DHCPP-PRB)	Centers for Disease Control & Prevention (CDC), Division of High Consequence Pathogens and Pathology (DHCPP-PRB)	Gigante,C.M., Epie,N., Zhao,H., Batra,D., Hetrick,E.E., Howard,D.T., Kovar,L., Seabolt,M.H., Weigand,M.R., Burroughs,M., Lee,J., Wilkins,K., McCollum,A., Hutson,C., Davidson,W., Rao,A., Perez,T. and Li,Y.
EPI_ISL_15016118, EPI_ISL_15016119, EPI_ISL_15016120	Centers for Disease Control & Prevention (CDC), Division of High Consequence Pathogens and Pathology (DHCPP-PRB)	Centers for Disease Control & Prevention (CDC), Division of High Consequence Pathogens and Pathology (DHCPP-PRB)	Gigante,C.M., Acheampong,E., Zhao,H., Batra,D., Hetrick,E.E., Howard,D.T., Kovar,L., Seabolt,M.H., Weigand,M.R., Burroughs,M., Lee,J., Wilkins,K., McCollum,A., Hutson,C., Davidson,W., Rao,A., McDermott,D. and Li,Y.
EPI_ISL_15016121	Centers for Disease Control & Prevention (CDC), Division of High Consequence Pathogens and Pathology (DHCPP-PRB)	Centers for Disease Control & Prevention (CDC), Division of High Consequence Pathogens and Pathology (DHCPP-PRB)	Gigante,C.M., Pettit,D., Zhao,H., Batra,D., Hetrick,E.E., Howard,D.T., Kovar,L., Seabolt,M.H., Weigand,M.R., Burroughs,M., Lee,J., Wilkins,K., McCollum,A., Hutson,C., Davidson,W., Rao,A., Deutsch-Feldman,M. and Li,Y.
EPI_ISL_15016122, EPI_ISL_15016123	Centers for Disease Control & Prevention (CDC), Division of High Consequence Pathogens and Pathology (DHCPP-PRB)	Centers for Disease Control & Prevention (CDC), Division of High Consequence Pathogens and Pathology (DHCPP-PRB)	Gigante,C.M., Wang,X., Zhao,H., Batra,D., Hetrick,E.E., Howard,D.T., Kovar,L., Seabolt,M.H., Weigand,M.R., Burroughs,M., Lee,J., Wilkins,K., McCollum,A., Hutson,C., Davidson,W., Rao,A., Ostadkar,R. and Li,Y.
EPI_ISL_15016124	Centers for Disease Control & Prevention (CDC), Division of High Consequence Pathogens and Pathology (DHCPP-PRB)	Centers for Disease Control & Prevention (CDC), Division of High Consequence Pathogens and Pathology (DHCPP-PRB)	Gigante,C.M., Haydel,D., Zhao,H., Batra,D., Hetrick,E.E., Howard,D.T., Kovar,L., Seabolt,M.H., Weigand,M.R., Burroughs,M., Lee,J., Wilkins,K., McCollum,A., Hutson,C., Davidson,W., Rao,A., Salinas,A. and Li,Y.
EPI_ISL_15016125, EPI_ISL_15016126	Centers for Disease Control & Prevention (CDC), Division of High Consequence Pathogens and Pathology (DHCPP-PRB)	Centers for Disease Control & Prevention (CDC), Division of High Consequence Pathogens and Pathology (DHCPP-PRB)	Gigante,C.M., Pavlick,J., Zhao,H., Batra,D., Hetrick,E.E., Howard,D.T., Kovar,L., Seabolt,M.H., Weigand,M.R., Burroughs,M., Lee,J., Wilkins,K., McCollum,A., Hutson,C., Davidson,W., Rao,A., Parrott,T. and Li,Y.
EPI_ISL_15016127, EPI_ISL_15016128	Centers for Disease Control & Prevention (CDC), Division of High Consequence Pathogens and Pathology (DHCPP-PRB)	Centers for Disease Control & Prevention (CDC), Division of High Consequence Pathogens and Pathology (DHCPP-PRB)	Gigante,C.M., Pearson,C., Zhao,H., Batra,D., Hetrick,E.E., Howard,D.T., Kovar,L., Seabolt,M.H., Weigand,M.R., Burroughs,M., Lee,J., Wilkins,K., McCollum,A., Hutson,C., Davidson,W., Rao,A., Maloney,M. and Li,Y.
EPI_ISL_15016129	Centers for Disease Control & Prevention (CDC), Division of High Consequence Pathogens and Pathology (DHCPP-PRB)	Centers for Disease Control & Prevention (CDC), Division of High Consequence Pathogens and Pathology (DHCPP-PRB)	Gigante,C.M., Hauser,J.R., Zhao,H., Batra,D., Hetrick,E.E., Howard,D.T., Kovar,L., Seabolt,M.H., Weigand,M.R., Burroughs,M., Lee,J., Wilkins,K., McCollum,A., Hutson,C., Davidson,W., Rao,A., Mangla,A. and Li,Y.
EPI_ISL_15016130	Centers for Disease Control & Prevention (CDC), Division of High Consequence Pathogens and Pathology (DHCPP-PRB)	Centers for Disease Control & Prevention (CDC), Division of High Consequence Pathogens and Pathology (DHCPP-PRB)	Gigante,C.M., Pearson,C., Zhao,H., Batra,D., Hetrick,E.E., Howard,D.T., Kovar,L., Seabolt,M.H., Weigand,M.R., Burroughs,M., Lee,J., Wilkins,K., McCollum,A., Hutson,C., Davidson,W., Rao,A., Maloney,M. and Li,Y.
EPI_ISL_15016131, EPI_ISL_15016132, EPI_ISL_15016133, EPI_ISL_15016134, EPI_ISL_15016135	Centers for Disease Control & Prevention (CDC), Division of High Consequence Pathogens and Pathology (DHCPP-PRB)	Centers for Disease Control & Prevention (CDC), Division of High Consequence Pathogens and Pathology (DHCPP-PRB)	Gigante,C.M., Ventura,J., Zhao,H., Batra,D., Hetrick,E.E., Howard,D.T., Kovar,L., Seabolt,M.H., Weigand,M.R., Burroughs,M., Lee,J., Wilkins,K., McCollum,A., Hutson,C., Davidson,W., Rao,A., Nash,J. and Li,Y.
EPI_ISL_15022589, EPI_ISL_15022590	Indian Council of Medical Research-National Institute of Virology	Indian Council of Medical Research-National Institute of Virology	Pragya Yadav, Rima Sahay, Anita Aich Shete, Sreelekshmy Mohandas, Priya Abraham
EPI_ISL_15023203	Centers for Disease Control & Prevention (CDC), Division of High Consequence Pathogens and Pathology (DHCPP-PRB)	Centers for Disease Control & Prevention (CDC), Division of High Consequence Pathogens and Pathology (DHCPP-PRB)	Gigante,C.M., Pavlick,J., Zhao,H., Batra,D., Hetrick,E.E., Howard,D.T., Kovar,L., Seabolt,M.H., Weigand,M.R., Burroughs,M., Lee,J., Wilkins,K., McCollum,A., Hutson,C., Davidson,W., Rao,A., Parrott,T. and Li,Y.
EPI_ISL_15055820	Sicilian Regional Laboratory - AOUP "P. Giaccone" - University of Palermo	Sicilian Regional Laboratory - AOUP "P. Giaccone" - University of Palermo	Fabio Tramuto, Carmelo Massimo Maida, Giulia Randazzo, Valeria Guzzetta, Walter Mazzucco, Giorgio Graziano, Vincenzo Restivo, Claudio Costantino, Francesco Vitale
EPI_ISL_15076130, EPI_ISL_15076131	Environmental, Agricultural, and Occupational Health, University of Nebraska Medical Center	Environmental, Agricultural, and Occupational Health, University of Nebraska Medical Center	Chapman,R.C., Bernhard,K., McCutchen,E.L., Fauver,J.R., O'Dell,J.X., Mannell,M., Wiley,M.R. and Cross,S.T.
EPI_ISL_15076132, EPI_ISL_15076133, EPI_ISL_15076134, EPI_ISL_15076135, EPI_ISL_15076136, EPI_ISL_15076137, EPI_ISL_15076138, EPI_ISL_15076139, EPI_ISL_15076140, EPI_ISL_15076141, EPI_ISL_15076142, EPI_ISL_15076143, EPI_ISL_15076144, EPI_ISL_15076145, EPI_ISL_15076146, EPI_ISL_15076147, EPI_ISL_15076148, EPI_ISL_15076149, EPI_ISL_15076150, EPI_ISL_15076151, EPI_ISL_15076152, EPI_ISL_15076153, EPI_ISL_15076154, EPI_ISL_15076155, EPI_ISL_15076156, EPI_ISL_15076157, EPI_ISL_15076158, EPI_ISL_15076159, EPI_ISL_15076160, EPI_ISL_15076161, EPI_ISL_15076162, EPI_ISL_15076163, EPI_ISL_15076164, EPI_ISL_15076165			
see above	Centre for Biological Threats, Highly Pathogenic Viruses, Robert Koch Institute	Centre for Biological Threats, Highly Pathogenic Viruses, Robert Koch Institute	Brinkmann,A., Kohl,C., Pape,K., Uddin,S., Schrick,L., Michel,J., Schaade,L. and Nitsche,A
EPI_ISL_15076180, EPI_ISL_15076181, EPI_ISL_15076182, EPI_ISL_15076183, EPI_ISL_15076184, EPI_ISL_15076185, EPI_ISL_15076186, EPI_ISL_15076187, EPI_ISL_15076188, EPI_ISL_15076189, EPI_ISL_15076191, EPI_ISL_15076192, EPI_ISL_15076193, EPI_ISL_15076194, EPI_ISL_15076195, EPI_ISL_15076196, EPI_ISL_15076197			
see above	Department of Genetics, University of North Carolina at Chapel Hill	Department of Genetics, University of North Carolina at Chapel Hill	Deanhardt,B., Miller,M. and Wang,J.R.

EPI_ISL_15083938, EPI_ISL_15083939, EPI_ISL_15083940, EPI_ISL_15083941, EPI_ISL_15083942, EPI_ISL_15083943, EPI_ISL_15083944, EPI_ISL_15083945, EPI_ISL_15083946, EPI_ISL_15083947	Centre for Biological Threats, Highly Pathogenic Viruses, Robert Koch Institute	Centre for Biological Threats, Highly Pathogenic Viruses, Robert Koch Institute	Brinkmann,A., Kohl,C., Pape,K., Schrick,L., Michel,J., Schaade,L. and Nitsche,A.
EPI_ISL_15098376, EPI_ISL_15098377, EPI_ISL_15098378, EPI_ISL_15098379, EPI_ISL_15098381, EPI_ISL_15098382, EPI_ISL_15098383, EPI_ISL_15098384, EPI_ISL_15098385, EPI_ISL_15098386, EPI_ISL_15098387, EPI_ISL_15098388, EPI_ISL_15098389, EPI_ISL_15098390, EPI_ISL_15098391, EPI_ISL_15098392, EPI_ISL_15098393, EPI_ISL_15098394, EPI_ISL_15098395			
see above	CT Department of Public Health	CT Department of Public Health	Claire Pearson, Tu N. Nguyen, Kutluhan Incekara
EPI_ISL_15104903	Institute for Virology, Philipps-University Marburg	Institute for Virology, Philipps-University Marburg	Eickmann, M., Lier, C., Kowalski, K., Kraft, F., Becker, S.
EPI_ISL_15116266, EPI_ISL_15116267, EPI_ISL_15116268, EPI_ISL_15116269, EPI_ISL_15116270, EPI_ISL_15116271, EPI_ISL_15116272, EPI_ISL_15116273, EPI_ISL_15116274, EPI_ISL_15116275, EPI_ISL_15116276, EPI_ISL_15116277, EPI_ISL_15116278, EPI_ISL_15116279, EPI_ISL_15116280, EPI_ISL_15116281, EPI_ISL_15116282, EPI_ISL_15116283, EPI_ISL_15116284, EPI_ISL_15116285, EPI_ISL_15116286, EPI_ISL_15116287, EPI_ISL_15116288, EPI_ISL_15116289, EPI_ISL_15116290, EPI_ISL_15116291, EPI_ISL_15116292, EPI_ISL_15116293, EPI_ISL_15116294, EPI_ISL_15116295, EPI_ISL_15116296, EPI_ISL_15116297, EPI_ISL_15116298, EPI_ISL_15116299			
see above	Centre for Biological Threats, Highly Pathogenic Viruses, Robert Koch Institute	Centre for Biological Threats, Highly Pathogenic Viruses, Robert Koch Institute	Brinkmann,A., Kohl,C., Pape,K., Schrick,L., Michel,J., Schaade,L. and Nitsche,A.
EPI_ISL_15116300, EPI_ISL_15116301, EPI_ISL_15116302, EPI_ISL_15116303, EPI_ISL_15116304, EPI_ISL_15116305, EPI_ISL_15116306, EPI_ISL_15116307	Centers for Disease Control & Prevention (CDC), Division of High Consequence Pathogens and Pathology (DHCPP-PRB)	Centers for Disease Control & Prevention (CDC), Division of High Consequence Pathogens and Pathology (DHCPP-PRB)	Gigante,C.M., Plumb,M., Ruprecht,A., Zhao,H., Wicker,V., Wilkins,K., Matheny,A., Khan,T., Davidson,W., Sheth,M., Burgin,A., Burroughs,M., Padilla,J., Lee,J.S., Batra,D., Hetrick,E.E., Howard,D.T., Garfin,J., Tate,L., Hubsmith,S.J., Mendoza,R.M., Stanek,D., Gillani,S., Lee,M., Mangla,A., Blythe,D., SierraPatev,S., Carpenter-Azevedo,K., Huard,R.C., Gallagher,G., Hall,J., Ash,S., Kovar,L., Seabolt,M.H., Weigand,M.R., Damon,I., Satheshkumar,P.S., McCollum,A.M., Hutson,C.L., Wang,X. and Li,Y.
EPI_ISL_15120448, EPI_ISL_15120449, EPI_ISL_15120450, EPI_ISL_15120451, EPI_ISL_15120452, EPI_ISL_15120453, EPI_ISL_15120454, EPI_ISL_15120455, EPI_ISL_15120456, EPI_ISL_15120457, EPI_ISL_15120458, EPI_ISL_15120459, EPI_ISL_15120461, EPI_ISL_15120462, EPI_ISL_15120463, EPI_ISL_15120464, EPI_ISL_15120465, EPI_ISL_15120466, EPI_ISL_15120467, EPI_ISL_15120468, EPI_ISL_15120469, EPI_ISL_15120470, EPI_ISL_15120473, EPI_ISL_15120474, EPI_ISL_15120475, EPI_ISL_15120477, EPI_ISL_15120478, EPI_ISL_15120479, EPI_ISL_15120480, EPI_ISL_15120481, EPI_ISL_15120482, EPI_ISL_15120483, EPI_ISL_15120484, EPI_ISL_15120485, EPI_ISL_15120486, EPI_ISL_15120487, EPI_ISL_15120489, EPI_ISL_15120490, EPI_ISL_15120492, EPI_ISL_15120494, EPI_ISL_15120495, EPI_ISL_15120496, EPI_ISL_15120497, EPI_ISL_15120498, EPI_ISL_15120499, EPI_ISL_15120500, EPI_ISL_15120501, EPI_ISL_15120502, EPI_ISL_15120503, EPI_ISL_15120504			
see above	Los Angeles County Public Health Laboratories	Los Angeles County Public Health Laboratories	P. Hemarajata et al.
EPI_ISL_15158314	Arbovirology and Entomology, Bernhard Nocht Institute for Tropical Medicine	Arbovirology and Entomology, Bernhard Nocht Institute for Tropical Medicine	Emmerich,P., Bialonski,A., Tomazatos,A. and Cadar,D.
EPI_ISL_15158315, EPI_ISL_15158316, EPI_ISL_15158317, EPI_ISL_15158318, EPI_ISL_15158319, EPI_ISL_15158320, EPI_ISL_15158321, EPI_ISL_15158322, EPI_ISL_15158323, EPI_ISL_15158324, EPI_ISL_15158325, EPI_ISL_15158326, EPI_ISL_15158327, EPI_ISL_15158328, EPI_ISL_15158329, EPI_ISL_15158330, EPI_ISL_15158331, EPI_ISL_15158332, EPI_ISL_15158333, EPI_ISL_15158335, EPI_ISL_15158336, EPI_ISL_15158337, EPI_ISL_15158338, EPI_ISL_15158339, EPI_ISL_15158340, EPI_ISL_15158341, EPI_ISL_15158342, EPI_ISL_15158343, EPI_ISL_15158344, EPI_ISL_15158345, EPI_ISL_15158346, EPI_ISL_15158347, EPI_ISL_15158348, EPI_ISL_15158350, EPI_ISL_15158351, EPI_ISL_15158352, EPI_ISL_15158353, EPI_ISL_15158354, EPI_ISL_15158355, EPI_ISL_15158356, EPI_ISL_15158357, EPI_ISL_15158358, EPI_ISL_15158359, EPI_ISL_15158360, EPI_ISL_15158361, EPI_ISL_15158362, EPI_ISL_15158363, EPI_ISL_15158364, EPI_ISL_15158365, EPI_ISL_15158366, EPI_ISL_15158367, EPI_ISL_15158368, EPI_ISL_15158369, EPI_ISL_15158370, EPI_ISL_15158371, EPI_ISL_15158374, EPI_ISL_15158375, EPI_ISL_15158376, EPI_ISL_15158378, EPI_ISL_15158379, EPI_ISL_15158380, EPI_ISL_15158381, EPI_ISL_15158382, EPI_ISL_15158383, EPI_ISL_15158384, EPI_ISL_15158385, EPI_ISL_15158387, EPI_ISL_15158388, EPI_ISL_15158389, EPI_ISL_15158390, EPI_ISL_15158391, EPI_ISL_15158392, EPI_ISL_15158393, EPI_ISL_15158394, EPI_ISL_15158395, EPI_ISL_15158396, EPI_ISL_15158397, EPI_ISL_15158398			
see above	Molecular Biology, Microbiology, and Biochemistry, Southern Illinois University	Molecular Biology, Microbiology, and Biochemistry, Southern Illinois University	Gagnon,K.T.
EPI_ISL_15158399, EPI_ISL_15158400, EPI_ISL_15158401, EPI_ISL_15158402, EPI_ISL_15158403, EPI_ISL_15158404, EPI_ISL_15158405, EPI_ISL_15158406, EPI_ISL_15158407, EPI_ISL_15158408, EPI_ISL_15158409, EPI_ISL_15158410, EPI_ISL_15158411, EPI_ISL_15158412, EPI_ISL_15158413, EPI_ISL_15158414, EPI_ISL_15158415, EPI_ISL_15158416, EPI_ISL_15158417, EPI_ISL_15158418, EPI_ISL_15158419, EPI_ISL_15158420, EPI_ISL_15158421, EPI_ISL_15158422, EPI_ISL_15158423, EPI_ISL_15158424, EPI_ISL_15158425, EPI_ISL_15158426, EPI_ISL_15158427, EPI_ISL_15158428, EPI_ISL_15158429, EPI_ISL_15158430, EPI_ISL_15158431, EPI_ISL_15158432, EPI_ISL_15158433, EPI_ISL_15158434, EPI_ISL_15158435, EPI_ISL_15158436, EPI_ISL_15158437, EPI_ISL_15158438, EPI_ISL_15158439, EPI_ISL_15158440, EPI_ISL_15158441, EPI_ISL_15158442, EPI_ISL_15158443, EPI_ISL_15158444, EPI_ISL_15158445, EPI_ISL_15158446, EPI_ISL_15158447, EPI_ISL_15158448, EPI_ISL_15158449, EPI_ISL_15158450, EPI_ISL_15158451, EPI_ISL_15158452, EPI_ISL_15158453, EPI_ISL_15158454, EPI_ISL_15158455, EPI_ISL_15158456, EPI_ISL_15158457, EPI_ISL_15158458, EPI_ISL_15158459, EPI_ISL_15158460, EPI_ISL_15158461, EPI_ISL_15158462, EPI_ISL_15158463, EPI_ISL_15158464, EPI_ISL_15158465, EPI_ISL_15158466, EPI_ISL_15158467, EPI_ISL_15158468			
see above	Research and Evaluation, UKHSA	Research and Evaluation, UKHSA	Groves,N., Osman,K.L., Lewandowski,K.S., Carter,D.P., Pullan,S.T., Myers,R., Vipond,R. and Chand,M.
EPI_ISL_15165602, EPI_ISL_15165603, EPI_ISL_15165604, EPI_ISL_15165605, EPI_ISL_15165606, EPI_ISL_15165607, EPI_ISL_15165608, EPI_ISL_15165610, EPI_ISL_15165611, EPI_ISL_15165612, EPI_ISL_15165614, EPI_ISL_15165615, EPI_ISL_15165616, EPI_ISL_15165617, EPI_ISL_15165618			
see above	Centro de Desenvolvimento Científico e Tecnológico (CDCT), Centro Estadual de Vigilância em Saúde (CEVS) da Secretaria Estadual da Saúde (SES-RS)	Centro de Desenvolvimento Científico e Tecnológico (CDCT), Centro Estadual de Vigilância em Saúde (CEVS) da Secretaria Estadual da Saúde (SES-RS)	Richard Steiner Salvato, Fernanda Marques Godinho, Regina Bones Barcellos, Patricia Sesterheim, Amanda Pellenz Ruivo, Viviane Horn de Melo, Júlio Augusto Schroder
EPI_ISL_15199623, EPI_ISL_15199624, EPI_ISL_15199627, EPI_ISL_15199628, EPI_ISL_15199629, EPI_ISL_15199630, EPI_ISL_15199632, EPI_ISL_15199635, EPI_ISL_15199636, EPI_ISL_15199638, EPI_ISL_15199639, EPI_ISL_15199640, EPI_ISL_15199643, EPI_ISL_15199644, EPI_ISL_15199645, EPI_ISL_15199646, EPI_ISL_15199647, EPI_ISL_15199648, EPI_ISL_15199649, EPI_ISL_15199652, EPI_ISL_15199653, EPI_ISL_15199655, EPI_ISL_15199656, EPI_ISL_15199657, EPI_ISL_15199658, EPI_ISL_15199659, EPI_ISL_15199660, EPI_ISL_15199661, EPI_ISL_15199662, EPI_ISL_15199664, EPI_ISL_15199665, EPI_ISL_15199666, EPI_ISL_15199667, EPI_ISL_15199668, EPI_ISL_15199669, EPI_ISL_15199670, EPI_ISL_15199671, EPI_ISL_15199672, EPI_ISL_15199673, EPI_ISL_15199675, EPI_ISL_15199676, EPI_ISL_15199677, EPI_ISL_15199679, EPI_ISL_15199680, EPI_ISL_15199681, EPI_ISL_15199682, EPI_ISL_15199685, EPI_ISL_15199686, EPI_ISL_15199687, EPI_ISL_15199688, EPI_ISL_15199690, EPI_ISL_15199691, EPI_ISL_15199692, EPI_ISL_15199693, EPI_ISL_15199695, EPI_ISL_15199696, EPI_ISL_15199697, EPI_ISL_15199698, EPI_ISL_15199699, EPI_ISL_15199700, EPI_ISL_15199701, EPI_ISL_15199702, EPI_ISL_15199703, EPI_ISL_15199705, EPI_ISL_15199706, EPI_ISL_15199707, EPI_ISL_15199710, EPI_ISL_15199711, EPI_ISL_15199712, EPI_ISL_15199713, EPI_ISL_15199717, EPI_ISL_15199720, EPI_ISL_15199721, EPI_ISL_15199723, EPI_ISL_15199724, EPI_ISL_15199725, EPI_ISL_15199726, EPI_ISL_15199727, EPI_ISL_15199728, EPI_ISL_15199729, EPI_ISL_15199730, EPI_ISL_15199731, EPI_ISL_15199732, EPI_ISL_15199733, EPI_ISL_15199735, EPI_ISL_15199736, EPI_ISL_15199737, EPI_ISL_15199738, EPI_ISL_15199739, EPI_ISL_15199740, EPI_ISL_15199741, EPI_ISL_15199742, EPI_ISL_15199743, EPI_ISL_15199744, EPI_ISL_15199745, EPI_ISL_15199747, EPI_ISL_15199748, EPI_ISL_15199749, EPI_ISL_15199750, EPI_ISL_15199751, EPI_ISL_15199752, EPI_ISL_15199753, EPI_ISL_15199754, EPI_ISL_15199755, EPI_ISL_15199757, EPI_ISL_15199758, EPI_ISL_15199759, EPI_ISL_15199761, EPI_ISL_15199762, EPI_ISL_15199763, EPI_ISL_15199764, EPI_ISL_15199765, EPI_ISL_15199766, EPI_ISL_15199767, EPI_ISL_15199768, EPI_ISL_15199769, EPI_ISL_15199773, EPI_ISL_15199775, EPI_ISL_15199776, EPI_ISL_15199777, EPI_ISL_15199778, EPI_ISL_15199780, EPI_ISL_15199781, EPI_ISL_15199785, EPI_ISL_15199787, EPI_ISL_15199788, EPI_ISL_15199789, EPI_ISL_15199790, EPI_ISL_15199791, EPI_ISL_15199792, EPI_ISL_15199793, EPI_ISL_15199794, EPI_ISL_15199795, EPI_ISL_15199796, EPI_ISL_15199797, EPI_ISL_15199798, EPI_ISL_15199799, EPI_ISL_15199830, EPI_ISL_15199856, EPI_ISL_15199859, EPI_ISL_15199860, EPI_ISL_15199861, EPI_ISL_15199862, EPI_ISL_15199863, EPI_ISL_15199864, EPI_ISL_15199866, EPI_ISL_15199868, EPI_ISL_15199869, EPI_ISL_15199870, EPI_ISL_15199871, EPI_ISL_15200120			
see above	Department of Infectious Diseases, National Institute of Health Doutor Ricardo Jorge, Portugal (INSA)	Department of Infectious Diseases, National Institute of Health Doutor Ricardo Jorge, Portugal (INSA)	Isidro,J., Borges,V., Pinto,M., Sobral,D., Santos,J., Nunes,A., Mixao,V., Ferreira,R., Santos,D., Duarte,S., Vieira,L., Borrego,M.J., Nuncio,S., Lopes de Carvalho,I., Pelerito,A., Cordeiro,R. and Gomes,J.P.
EPI_ISL_15226669, EPI_ISL_15226670, EPI_ISL_15226671, EPI_ISL_15226672, EPI_ISL_15226673, EPI_ISL_15226674, EPI_ISL_15226675, EPI_ISL_15226676, EPI_ISL_15226677, EPI_ISL_15226678, EPI_ISL_15226679, EPI_ISL_15226681, EPI_ISL_15226682, EPI_ISL_15226683, EPI_ISL_15226684, EPI_ISL_15226685, EPI_ISL_15226686, EPI_ISL_15226687, EPI_ISL_15226688, EPI_ISL_15226689, EPI_ISL_15226690, EPI_ISL_15226691, EPI_ISL_15226692, EPI_ISL_15226693, EPI_ISL_15226694, EPI_ISL_15226695			
see above	Los Angeles County Public Health Laboratories	Los Angeles County Public Health Laboratories	P. Hemarajata et al.
EPI_ISL_15247221, EPI_ISL_15247222, EPI_ISL_15247223, EPI_ISL_15247224, EPI_ISL_15247225, EPI_ISL_15247226, EPI_ISL_15247227, EPI_ISL_15247228	Medical University of Vienna Center for Virology	Medical University of Vienna Center for Virology	Jeremy V. Camp, Monika Redlberger-Fritz, Stephan W. Aberle
EPI_ISL_15257658, EPI_ISL_15257659, EPI_ISL_15257660, EPI_ISL_15257661, EPI_ISL_15257662, EPI_ISL_15257663, EPI_ISL_15257664, EPI_ISL_15257665, EPI_ISL_15257666, EPI_ISL_15257667, EPI_ISL_15257668			
see above	Centre for Biological Threats, Highly Pathogenic Viruses, Robert Koch Institute	Centre for Biological Threats, Highly Pathogenic Viruses, Robert Koch Institute	Brinkmann,A., Kohl,C., Pape,K., Schrick,L., Michel,J., Schaade,L. and Nitsche,A.
EPI_ISL_15257669	Public Health Agency of Canada, National Microbiology Laboratory	Public Health Agency of Canada, National Microbiology Laboratory	Duggan,A., Hole,D., Yadav,C., Knox,N., Haidl,E., Chapel,M., Tyler,A.D., Domselaar,G.V., Graham,M., Audet,J., Fernando,L., Hagan,M., Safronetz,D., Leung,A., Peters,G., Go,A., Kaplen,B., Antonation,K., Laminman,V., Jolly,G., Croxen,M., Deo,A., Dieu,P., Dong,X., Gill,K., Granger,D., Ferrato,C., Ikkurti,V., Kanji,J., Koleva,P., Li,V., Lloyd,C., Lynch,T., Ma,R., Pabbaraju,K., Rotich,S., Sergeant,H., Skitsko,T., Tipples,G., Thayer,J., Shideler,S. and Wong,A.
EPI_ISL_15257670, EPI_ISL_15257671, EPI_ISL_15257672, EPI_ISL_15257673, EPI_ISL_15257674, EPI_ISL_15257675, EPI_ISL_15257676, EPI_ISL_15257678, EPI_ISL_15257679, EPI_ISL_15257680, EPI_ISL_15257681, EPI_ISL_15257682, EPI_ISL_15257683, EPI_ISL_15257684, EPI_ISL_15257685, EPI_ISL_15257686, EPI_ISL_15257687			
see above	Viral and Rickettsial Disease Laboratory, California Department of Public Health	Viral and Rickettsial Disease Laboratory, California Department of Public Health	Probert,W., Espinosa,A., Kath,C., Haw,M., O'Neil,R., Bell,J. and Hacker,J.
EPI_ISL_15260057	High Containment Facility, Virology Division, Defence Research & Develeopment Establishment (DRDE)	Virology Division, Defence Research & Develeopment Establishment (DRDE)	Dr Sushil Kumar Sharma, Dr Paban Kumar Dash, Ram Govind Yadav, Ambuj Shrivastava, Rohit Menon, Dr Jyoti S Kumar, Dr Shashi Sharma, Dr Suman Dhankher, Divya Kumari, Dr Manmohan Parida
EPI_ISL_15263355	Sicilian Regional Laboratory - AOUP "P. Giaccone" - University of Palermo	Sicilian Regional Laboratory - AOUP "P. Giaccone" - University of Palermo	Fabio Tramuto, Carmelo Massimo Maida, Giulia Randazzo, Valeria Guzzetta, Walter Mazzucco, Giorgio Graziano, Vincenzo Restivo, Claudio Costantino, Francesco Vitale
EPI_ISL_15264003, EPI_ISL_15266514, EPI_ISL_15266518, EPI_ISL_15266615	Erasmus Medical Center Department of Virology	Erasmus Medical Center Department of Virology	Leonard Schuele, Bas Oude Munnink, Marjan Boter, Babette Weller, Babs Verstrepen, Richard Molenkamp, Janette Rahamat-Langendoen, Reina Sikkema, Marion Koopmans
EPI_ISL_15266843	Erasmus Medical Center Department of Virology	Erasmus Medical Center Department of Virology	Leonard Schuele, Bas Oude Munnink, Marjan Boter, David Nieuwenhuijse, Babette Weller, Babs Verstrepen, Richard Molenkamp, Janette Rahamat-Langendoen, Reina Sikkema, Marion Koopmans
EPI_ISL_15266988, EPI_ISL_15267015, EPI_ISL_15267029, EPI_ISL_15267031, EPI_ISL_15267800, EPI_ISL_15268281, EPI_ISL_15269049	Erasmus Medical Center Department of Virology	Erasmus Medical Center Department of Virology	Leonard Schuele, Bas Oude Munnink, Marjan Boter, Babette Weller, Babs Verstrepen, Richard Molenkamp, Janette Rahamat-Langendoen, Reina Sikkema, Marion Koopmans
EPI_ISL_15269237, EPI_ISL_15269238, EPI_ISL_15269375, EPI_ISL_15269384, EPI_ISL_15269386, EPI_ISL_15269387, EPI_ISL_15269388, EPI_ISL_15269544, EPI_ISL_15269595, EPI_ISL_15269598	Erasmus Medical Center Department of Virology	Erasmus Medical Center Department of Virology	Leonard Schuele, Bas Oude Munnink, Marjan Boter, David Nieuwenhuijse, Babette Weller, Babs Verstrepen, Richard Molenkamp, Janette Rahamat-Langendoen, Reina Sikkema, Marion Koopmans
EPI_ISL_15269698, EPI_ISL_15269699, EPI_ISL_15269702, EPI_ISL_15269704, EPI_ISL_15269708	Erasmus Medical Center Department of Virology	Erasmus Medical Center Department of Virology	Leonard Schuele, Bas Oude Munnink, Marjan Boter, Babette Weller, Babs Verstrepen, Richard Molenkamp, Janette Rahamat-Langendoen, Reina Sikkema, Marion Koopmans
EPI_ISL_15283583, EPI_ISL_15283585, EPI_ISL_15283587, EPI_ISL_15283589, EPI_ISL_15283590, EPI_ISL_15283592, EPI_ISL_15283594, EPI_ISL_15283596, EPI_ISL_15283598, EPI_ISL_15283600, EPI_ISL_15283601, EPI_ISL_15283603, EPI_ISL_15283605, EPI_ISL_15283607, EPI_ISL_15283608, EPI_ISL_15283610, EPI_ISL_15283611, EPI_ISL_15283612, EPI_ISL_15283614, EPI_ISL_15283616, EPI_ISL_15283618, EPI_ISL_15283620, EPI_ISL_15283622			
see above	Centre for Biological Threats, Highly Pathogenic Viruses, Robert Koch Institute	Centre for Biological Threats, Highly Pathogenic Viruses, Robert Koch Institute	Brinkmann,A., Kohl,C., Pape,K., Schrick,L., Michel,J., Schaade,L. and Nitsche,A.
EPI_ISL_15292947	Center of Scientific Excellence for Influenza Viruses, National Research Centre (NRC)	Center of Scientific Excellence for Influenza Viruses, National Research Centre (NRC)	Roshdy,W.H., El-Shesheny,R., Moatasim,Y.M., Kamel,M., Shawky,S., Gomaa,M., Naguib,A., El Guindy,N., Fahim,M., Khalifa,M., Galal,R., Hassany,M., Mohsen,A., Ali,M.A. and Kandeel,A.
EPI_ISL_15292951, EPI_ISL_15292953, EPI_ISL_15292954, EPI_ISL_15292960, EPI_ISL_15292962, EPI_ISL_15292969	Minnesota Department of Health	Minnesota Department of Health	Plumb,M.R.
EPI_ISL_15292973, EPI_ISL_15292974, EPI_ISL_15292975	USAFSAM, US Air Force	USAFSAM, US Air Force	Fries,A.
EPI_ISL_15292976, EPI_ISL_15292977, EPI_ISL_15292978, EPI_ISL_15292979, EPI_ISL_15292980, EPI_ISL_15292981, EPI_ISL_15292982, EPI_ISL_15292983, EPI_ISL_15292984, EPI_ISL_15292985, EPI_ISL_15292986, EPI_ISL_15292987, EPI_ISL_15292988, EPI_ISL_15292989, EPI_ISL_15292990, EPI_ISL_15292991, EPI_ISL_15292992, EPI_ISL_15292993, EPI_ISL_15292994, EPI_ISL_15292995,			

EPI_ISL_15292996, EPI_ISL_15292997, EPI_ISL_15292998, EPI_ISL_15292999, EPI_ISL_15293000, EPI_ISL_15293001, EPI_ISL_15293002, EPI_ISL_15293003, EPI_ISL_15293004, EPI_ISL_15293005, EPI_ISL_15293006, EPI_ISL_15293007, EPI_ISL_15293008, EPI_ISL_15293009			
see above	Institute of Health Carlos III, Bioinformatics Unit	Institute of Health Carlos III, Bioinformatics Unit	Cuesta,I.
EPI_ISL_15293815	National Institute for Viral Disease Control and Prevention (IVDC), Chinese Center for Disease Control and Prevention , Beijing, China	National Institute for Viral Disease Control and Prevention (IVDC), Chinese Center for Disease Control and Prevention , Beijing, China	Wenjie Tan, Changcheng Wu, Ruhan A, Wenling Wang, Roujian Lu, Li Zhao, Baoying Huang, Fei Ye, Wenbo Xu
EPI_ISL_15317143	Centers for Disease Control & Prevention (CDC), Division of High Consequence Pathogens and Pathology (DHCPP-PRB)	Centers for Disease Control & Prevention (CDC), Division of High Consequence Pathogens and Pathology (DHCPP-PRB)	Gigante,C.M., Vang,K., Zhao,H., Batra,D., Hetrick,E.E., Howard,D.T., Kovar,L., Seabolt,M.H., Weigand,M.R., Burroughs,M., Lee,J., Wilkins,K., McCollum,A., Hutson,C., Davidson,W., Rao,A., Seely,K. and Li,Y.
EPI_ISL_15317144	Centers for Disease Control & Prevention (CDC), Division of High Consequence Pathogens and Pathology (DHCPP-PRB)	Centers for Disease Control & Prevention (CDC), Division of High Consequence Pathogens and Pathology (DHCPP-PRB)	Gigante,C.M., Segaloff,H., Zhao,H., Batra,D., Hetrick,E.E., Howard,D.T., Kovar,L., Seabolt,M.H., Weigand,M.R., Burroughs,M., Lee,J., Wilkins,K., McCollum,A., Hutson,C., Davidson,W., Rao,A., Florek,K. and Li,Y.
EPI_ISL_15317145	Centers for Disease Control & Prevention (CDC), Division of High Consequence Pathogens and Pathology (DHCPP-PRB)	Centers for Disease Control & Prevention (CDC), Division of High Consequence Pathogens and Pathology (DHCPP-PRB)	Gigante,C.M., Pavlick,J., Zhao,H., Batra,D., Hetrick,E.E., Howard,D.T., Kovar,L., Seabolt,M.H., Weigand,M.R., Burroughs,M., Lee,J., Wilkins,K., McCollum,A., Hutson,C., Davidson,W., Rao,A., Parrott,T. and Li,Y.
EPI_ISL_15317146	Centers for Disease Control & Prevention (CDC), Division of High Consequence Pathogens and Pathology (DHCPP-PRB)	Centers for Disease Control & Prevention (CDC), Division of High Consequence Pathogens and Pathology (DHCPP-PRB)	Gigante,C.M., Plumb,M., Ruprecht,A., Zhao,H., Wicker,V., Wilkins,K., Matheny,A., Khan,T., Davidson,W., Sheth,M., Burgin,A., Burroughs,M., Padilla,J., Lee,J.S., Batra,D., Hetrick,E.E., Howard,D.T., Garfin,J., Tate,L., Hubsmith,S.J., Mendoza,R.M., Stanek,D., Gillani,S., Lee,M., Mangla,A., Blythe,D., SierraPatev,S., Carpenter-Azevedo,K., Huard,R.C., Gallagher,G., Hall,J., Ash,S., Kovar,L., Seabolt,M.H., Weigand,M.R., Damon,I., Satheshkumar,P.S., McCollum,A.M., Hutson,C.L., Wang,X. and Li,Y.
EPI_ISL_15317147	Centers for Disease Control & Prevention (CDC), Division of High Consequence Pathogens and Pathology (DHCPP-PRB)	Centers for Disease Control & Prevention (CDC), Division of High Consequence Pathogens and Pathology (DHCPP-PRB)	Gigante,C.M., Epie,N., Zhao,H., Batra,D., Hetrick,E.E., Howard,D.T., Kovar,L., Seabolt,M.H., Weigand,M.R., Burroughs,M., Lee,J., Wilkins,K., McCollum,A., Hutson,C., Davidson,W., Rao,A., Perez,T. and Li,Y.
EPI_ISL_15317148	Centers for Disease Control & Prevention (CDC), Division of High Consequence Pathogens and Pathology (DHCPP-PRB)	Centers for Disease Control & Prevention (CDC), Division of High Consequence Pathogens and Pathology (DHCPP-PRB)	Gigante,C.M., Acheampong,E., Zhao,H., Batra,D., Hetrick,E.E., Howard,D.T., Kovar,L., Seabolt,M.H., Weigand,M.R., Burroughs,M., Lee,J., Wilkins,K., McCollum,A., Hutson,C., Davidson,W., Rao,A., McDermott,D. and Li,Y.
EPI_ISL_15317149	Centers for Disease Control & Prevention (CDC), Division of High Consequence Pathogens and Pathology (DHCPP-PRB)	Centers for Disease Control & Prevention (CDC), Division of High Consequence Pathogens and Pathology (DHCPP-PRB)	Gigante,C.M., Xia,D., Zhao,H., Batra,D., Hetrick,E.E., Howard,D.T., Kovar,L., Seabolt,M.H., Weigand,M.R., Burroughs,M., Lee,J., Wilkins,K., McCollum,A., Hutson,C., Davidson,W., Rao,A., Plipat,N. and Li,Y.
EPI_ISL_15317150	Centers for Disease Control & Prevention (CDC), Division of High Consequence Pathogens and Pathology (DHCPP-PRB)	Centers for Disease Control & Prevention (CDC), Division of High Consequence Pathogens and Pathology (DHCPP-PRB)	Gigante,C.M., Ruiz,V., Zhao,H., Batra,D., Hetrick,E.E., Howard,D.T., Kovar,L., Seabolt,M.H., Weigand,M.R., Burroughs,M., Lee,J., Wilkins,K., McCollum,A., Hutson,C., Davidson,W., Rao,A., Wang,J. and Li,Y.
EPI_ISL_15317151	Centers for Disease Control & Prevention (CDC), Division of High Consequence Pathogens and Pathology (DHCPP-PRB)	Centers for Disease Control & Prevention (CDC), Division of High Consequence Pathogens and Pathology (DHCPP-PRB)	Gigante,C.M., Francis,D., Zhao,H., Batra,D., Hetrick,E.E., Howard,D.T., Kovar,L., Seabolt,M.H., Weigand,M.R., Burroughs,M., Lee,J., Wilkins,K., McCollum,A., Hutson,C., Davidson,W., Rao,A., Escobar,J. and Li,Y.
EPI_ISL_15317152	Centers for Disease Control & Prevention (CDC), Division of High Consequence Pathogens and Pathology (DHCPP-PRB)	Centers for Disease Control & Prevention (CDC), Division of High Consequence Pathogens and Pathology (DHCPP-PRB)	Gigante,C.M., Johnson,S., Zhao,H., Batra,D., Hetrick,E.E., Howard,D.T., Kovar,L., Seabolt,M.H., Weigand,M.R., Burroughs,M., Lee,J., Wilkins,K., McCollum,A., Hutson,C., Davidson,W., Rao,A., Riner,D. and Li,Y.
EPI_ISL_15317153	Centers for Disease Control & Prevention (CDC), Division of High Consequence Pathogens and Pathology (DHCPP-PRB)	Centers for Disease Control & Prevention (CDC), Division of High Consequence Pathogens and Pathology (DHCPP-PRB)	Gigante,C.M., Culbertson,M., Zhao,H., Batra,D., Hetrick,E.E., Howard,D.T., Kovar,L., Seabolt,M.H., Weigand,M.R., Burroughs,M., Lee,J., Wilkins,K., McCollum,A., Hutson,C., Davidson,W., Rao,A., Pope,B. and Li,Y.
EPI_ISL_15317154	Centers for Disease Control & Prevention (CDC), Division of High Consequence Pathogens and Pathology (DHCPP-PRB)	Centers for Disease Control & Prevention (CDC), Division of High Consequence Pathogens and Pathology (DHCPP-PRB)	Gigante,C.M., Ventura,J., Zhao,H., Batra,D., Hetrick,E.E., Howard,D.T., Kovar,L., Seabolt,M.H., Weigand,M.R., Burroughs,M., Lee,J., Wilkins,K., McCollum,A., Hutson,C., Davidson,W., Rao,A., Nash,J. and Li,Y.
EPI_ISL_15317155	Centers for Disease Control & Prevention (CDC), Division of High Consequence Pathogens and Pathology (DHCPP-PRB)	Centers for Disease Control & Prevention (CDC), Division of High Consequence Pathogens and Pathology (DHCPP-PRB)	Gigante,C.M., Francis,D., Zhao,H., Batra,D., Hetrick,E.E., Howard,D.T., Kovar,L., Seabolt,M.H., Weigand,M.R., Burroughs,M., Lee,J., Wilkins,K., McCollum,A., Hutson,C., Davidson,W., Rao,A., Escobar,J. and Li,Y.
EPI_ISL_15317156	Centers for Disease Control & Prevention (CDC), Division of High Consequence Pathogens and Pathology (DHCPP-PRB)	Centers for Disease Control & Prevention (CDC), Division of High Consequence Pathogens and Pathology (DHCPP-PRB)	Gigante,C.M., Ruiz,V., Zhao,H., Batra,D., Hetrick,E.E., Howard,D.T., Kovar,L., Seabolt,M.H., Weigand,M.R., Burroughs,M., Lee,J., Wilkins,K., McCollum,A., Hutson,C., Davidson,W., Rao,A., Wang,J. and Li,Y.
EPI_ISL_15317157	Centers for Disease Control & Prevention (CDC), Division of High Consequence Pathogens and Pathology (DHCPP-PRB)	Centers for Disease Control & Prevention (CDC), Division of High Consequence Pathogens and Pathology (DHCPP-PRB)	Gigante,C.M., Pavlick,J., Zhao,H., Batra,D., Hetrick,E.E., Howard,D.T., Kovar,L., Seabolt,M.H., Weigand,M.R., Burroughs,M., Lee,J., Wilkins,K., McCollum,A., Hutson,C., Davidson,W., Rao,A., Parrott,T. and Li,Y.
EPI_ISL_15317158, EPI_ISL_15317159, EPI_ISL_15317160	Centers for Disease Control & Prevention (CDC), Division of High Consequence Pathogens and Pathology (DHCPP-PRB)	Centers for Disease Control & Prevention (CDC), Division of High Consequence Pathogens and Pathology (DHCPP-PRB)	Gigante,C.M., Ventura,J., Zhao,H., Batra,D., Hetrick,E.E., Howard,D.T., Kovar,L., Seabolt,M.H., Weigand,M.R., Burroughs,M., Lee,J., Wilkins,K., McCollum,A., Hutson,C., Davidson,W., Rao,A., Nash,J. and Li,Y.
EPI_ISL_15321163, EPI_ISL_15321165, EPI_ISL_15321166, EPI_ISL_15321167, EPI_ISL_15321168, EPI_ISL_15321169, EPI_ISL_15321170, EPI_ISL_15321171, EPI_ISL_15321172, EPI_ISL_15321174, EPI_ISL_15321175			
see above	Rhode Island State Health Laboratory	Rhode Island State Health Laboratory	Kristin Carpenter-Azevedo, Sean Sierra-Patev, Richard C. Huard
EPI_ISL_15325408, EPI_ISL_15325409, EPI_ISL_15325410, EPI_ISL_15325411, EPI_ISL_15325413, EPI_ISL_15325414, EPI_ISL_15325415, EPI_ISL_15325416, EPI_ISL_15325417, EPI_ISL_15325418, EPI_ISL_15325419, EPI_ISL_15325421, EPI_ISL_15325422, EPI_ISL_15325423, EPI_ISL_15325424, EPI_ISL_15325425, EPI_ISL_15325426, EPI_ISL_15325428, EPI_ISL_15325429, EPI_ISL_15325430, EPI_ISL_15325431, EPI_ISL_15325432			
see above	Los Angeles County Public Health Laboratories	Los Angeles County Public Health Laboratories	P. Hemarajata et al.
EPI_ISL_15325433	Quest Diagnostics Nichols Institute	Los Angeles County Public Health Laboratories	P. Hemarajata et al.
EPI_ISL_15332321	Centers for Disease Control & Prevention (CDC), Division of High Consequence Pathogens and Pathology (DHCPP-PRB)	Centers for Disease Control & Prevention (CDC), Division of High Consequence Pathogens and Pathology (DHCPP-PRB)	Gigante,C.M., Carlson,C., Zhao,H., Batra,D., Hetrick,E.E., Howard,D.T., Kovar,L., Seabolt,M.H., Knipe,K., Burroughs,S., Lee,J., Wilkins,K., McCollum,A., Hutson,C., Davidson,W., Rao,A., Southern,T. and Li,Y.
EPI_ISL_15332322	Centers for Disease Control & Prevention (CDC), Division of High Consequence Pathogens and Pathology (DHCPP-PRB)	Centers for Disease Control & Prevention (CDC), Division of High Consequence Pathogens and Pathology (DHCPP-PRB)	Gigante,C.M., Murray,J., Zhao,H., Batra,D., Hetrick,E.E., Howard,D.T., Kovar,L., Seabolt,M.H., Knipe,K., Burroughs,S., Lee,J., Wilkins,K., McCollum,A., Hutson,C., Davidson,W., Rao,A., Atkinson,A. and Li,Y.
EPI_ISL_15332323	Centers for Disease Control & Prevention (CDC), Division of High Consequence Pathogens and Pathology (DHCPP-PRB)	Centers for Disease Control & Prevention (CDC), Division of High Consequence Pathogens and Pathology (DHCPP-PRB)	Gigante,C.M., Kubin,G., Zhao,H., Batra,D., Hetrick,E.E., Howard,D.T., Kovar,L., Seabolt,M.H., Knipe,K., Burroughs,S., Lee,J., Wilkins,K., McCollum,A., Hutson,C., Davidson,W., Rao,A., White,S.L. and Li,Y.
EPI_ISL_15332324	Centers for Disease Control & Prevention (CDC), Division of High Consequence Pathogens and Pathology (DHCPP-PRB)	Centers for Disease Control & Prevention (CDC), Division of High Consequence Pathogens and Pathology (DHCPP-PRB)	Gigante,C.M., Xia,D., Zhao,H., Batra,D., Hetrick,E.E., Howard,D.T., Kovar,L., Seabolt,M.H., Knipe,K., Burroughs,S., Lee,J., Wilkins,K., McCollum,A., Hutson,C., Davidson,W., Rao,A., Plipat,N. and Li,Y.
EPI_ISL_15332325	Centers for Disease Control & Prevention (CDC), Division of High Consequence Pathogens and Pathology (DHCPP-PRB)	Centers for Disease Control & Prevention (CDC), Division of High Consequence Pathogens and Pathology (DHCPP-PRB)	Gigante,C.M., Pettit,D., Zhao,H., Batra,D., Hetrick,E.E., Howard,D.T., Kovar,L., Seabolt,M.H., Knipe,K., Burroughs,S., Lee,J., Wilkins,K., McCollum,A., Hutson,C., Davidson,W., Rao,A., Deutsch-Feldman,M. and Li,Y.
EPI_ISL_15332326	Centers for Disease Control & Prevention (CDC), Division of High Consequence Pathogens and Pathology (DHCPP-PRB)	Centers for Disease Control & Prevention (CDC), Division of High Consequence Pathogens and Pathology (DHCPP-PRB)	Gigante,C.M., Buttery,E., Zhao,H., Batra,D., Hetrick,E.E., Howard,D.T., Kovar,L., Seabolt,M.H., Knipe,K., Burroughs,S., Lee,J., Wilkins,K., McCollum,A., Hutson,C., Davidson,W., Rao,A., Raman,D. and Li,Y.
EPI_ISL_15332327	Centers for Disease Control & Prevention (CDC), Division of High Consequence Pathogens and Pathology (DHCPP-PRB)	Centers for Disease Control & Prevention (CDC), Division of High Consequence Pathogens and Pathology (DHCPP-PRB)	Gigante,C.M., Acheampong,E., Zhao,H., Batra,D., Hetrick,E.E., Howard,D.T., Kovar,L., Seabolt,M.H., Knipe,K., Burroughs,S., Lee,J., Wilkins,K., McCollum,A., Hutson,C., Davidson,W., Rao,A., McDermott,D. and Li,Y.
EPI_ISL_15332328	Centers for Disease Control & Prevention (CDC), Division of High Consequence Pathogens and Pathology (DHCPP-PRB)	Centers for Disease Control & Prevention (CDC), Division of High Consequence Pathogens and Pathology (DHCPP-PRB)	Gigante,C.M., Pettit,D., Zhao,H., Batra,D., Hetrick,E.E., Howard,D.T., Kovar,L., Seabolt,M.H., Knipe,K., Burroughs,S., Lee,J., Wilkins,K., McCollum,A., Hutson,C., Davidson,W., Rao,A., Deutsch-Feldman,M. and Li,Y.
EPI_ISL_15332329, EPI_ISL_15332330	Centers for Disease Control & Prevention (CDC), Division of High Consequence Pathogens and Pathology (DHCPP-PRB)	Centers for Disease Control & Prevention (CDC), Division of High Consequence Pathogens and Pathology (DHCPP-PRB)	Gigante,C.M., Myers,R., Zhao,H., Batra,D., Hetrick,E.E., Howard,D.T., Kovar,L., Seabolt,M.H., Knipe,K., Burroughs,S., Lee,J., Wilkins,K., McCollum,A., Hutson,C., Davidson,W., Rao,A., Blythe,D. and Li,Y.
EPI_ISL_15332331	Centers for Disease Control & Prevention (CDC), Division of High Consequence Pathogens and Pathology (DHCPP-PRB)	Centers for Disease Control & Prevention (CDC), Division of High Consequence Pathogens and Pathology (DHCPP-PRB)	Gigante,C.M., Pavlick,J., Zhao,H., Batra,D., Hetrick,E.E., Howard,D.T., Kovar,L., Seabolt,M.H., Knipe,K., Burroughs,S., Lee,J., Wilkins,K., McCollum,A., Hutson,C., Davidson,W., Rao,A., Parrott,T. and Li,Y.
EPI_ISL_15332332, EPI_ISL_15332333	Centers for Disease Control & Prevention (CDC), Division of High Consequence Pathogens and Pathology (DHCPP-PRB)	Centers for Disease Control & Prevention (CDC), Division of High Consequence Pathogens and Pathology (DHCPP-PRB)	Gigante,C.M., Lee,P., Zhao,H., Batra,D., Hetrick,E.E., Howard,D.T., Kovar,L., Seabolt,M.H., Knipe,K., Burroughs,S., Lee,J., Wilkins,K., McCollum,A., Hutson,C., Davidson,W., Rao,A., Stanek,D. and Li,Y.
EPI_ISL_15332334	Centers for Disease Control & Prevention (CDC), Division of High Consequence Pathogens and Pathology (DHCPP-PRB)	Centers for Disease Control & Prevention (CDC), Division of High Consequence Pathogens and Pathology (DHCPP-PRB)	Gigante,C.M., Hauser,J.R., Zhao,H., Batra,D., Hetrick,E.E., Howard,D.T., Kovar,L., Seabolt,M.H., Knipe,K., Burroughs,S., Lee,J., Wilkins,K., McCollum,A., Hutson,C., Davidson,W., Rao,A., Mangla,A. and Li,Y.

EPI_ISL_15332335	Centers for Disease Control & Prevention (CDC), Division of High Consequence Pathogens and Pathology (DHCPP-PRB)	Centers for Disease Control & Prevention (CDC), Division of High Consequence Pathogens and Pathology (DHCPP-PRB)	Gigante,C.M., Ventura,J., Zhao,H., Batra,D., Hetrick,E.E., Howard,D.T., Kovar,L., Seabolt,M.H., Knipe,K., Burroughs,S., Lee,J., Wilkins,K., McCollum,A., Hutson,C., Davidson,W., Rao,A., Nash,J. and Li,Y.
EPI_ISL_15332336, EPI_ISL_15332337	Environmental, Agricultural, and Occupational Health, University of Nebraska Medical Center	Environmental, Agricultural, and Occupational Health, University of Nebraska Medical Center	Tegomoh,B., Cross,S.T., Chapman,R.C., Bernhard,K., McCutchen,E.L., Fauver,J.R., Pratt,C.B., Warden,D.E., Iwen,P.C., Donahue,M. and Wiley,M.R.
EPI_ISL_15332338, EPI_ISL_15332339, EPI_ISL_15332340	Environmental, Agricultural, and Occupational Health, University of Nebraska Medical Center	Environmental, Agricultural, and Occupational Health, University of Nebraska Medical Center	Chapman,R.C., Bernhard,K., McCutchen,E.L., Fauver,J.R., O'Dell,J.X., Mannell,M., Wiley,M.R. and Cross,S.T.
EPI_ISL_15352249, EPI_ISL_15352251, EPI_ISL_15352252, EPI_ISL_15352254, EPI_ISL_15352256, EPI_ISL_15352258, EPI_ISL_15352259, EPI_ISL_15352262, EPI_ISL_15352263, EPI_ISL_15352265, EPI_ISL_15352267, EPI_ISL_15352268, EPI_ISL_15352271, EPI_ISL_15352276, EPI_ISL_15352278, EPI_ISL_15352279, EPI_ISL_15352280, EPI_ISL_15352282, EPI_ISL_15352283, EPI_ISL_15352284, EPI_ISL_15352285, EPI_ISL_15352287, EPI_ISL_15352289, EPI_ISL_15352291, EPI_ISL_15352293, EPI_ISL_15352297, EPI_ISL_15352299, EPI_ISL_15352300, EPI_ISL_15352302, EPI_ISL_15352305, EPI_ISL_15352307, EPI_ISL_15352308, EPI_ISL_15352310, EPI_ISL_15352311, EPI_ISL_15352312, EPI_ISL_15352314			
see above	Centre for Biological Threats, Highly Pathogenic Viruses, Robert Koch Institute	Centre for Biological Threats, Highly Pathogenic Viruses, Robert Koch Institute	Brinkmann,A., Kohl,C., Pape,K., Schrick,L., Michel,J., Schaade,L. and Nitsche,A
EPI_ISL_15367957, EPI_ISL_15367958, EPI_ISL_15367959, EPI_ISL_15367960, EPI_ISL_15367961, EPI_ISL_15367962, EPI_ISL_15367963, EPI_ISL_15367964, EPI_ISL_15367965, EPI_ISL_15367966, EPI_ISL_15367967, EPI_ISL_15367968, EPI_ISL_15367969, EPI_ISL_15367970, EPI_ISL_15367971, EPI_ISL_15367972, EPI_ISL_15367973, EPI_ISL_15367974, EPI_ISL_15367975, EPI_ISL_15367976, EPI_ISL_15367977, EPI_ISL_15367978, EPI_ISL_15367979, EPI_ISL_15367980, EPI_ISL_15367981, EPI_ISL_15367982, EPI_ISL_15367983, EPI_ISL_15367984, EPI_ISL_15367985, EPI_ISL_15367986, EPI_ISL_15367987, EPI_ISL_15367988, EPI_ISL_15367989, EPI_ISL_15367990, EPI_ISL_15367991, EPI_ISL_15367992, EPI_ISL_15367993, EPI_ISL_15367994, EPI_ISL_15367995, EPI_ISL_15367996, EPI_ISL_15367997, EPI_ISL_15367998, EPI_ISL_15367999, EPI_ISL_15368000, EPI_ISL_15368001, EPI_ISL_15368002, EPI_ISL_15368003, EPI_ISL_15368004, EPI_ISL_15368005, EPI_ISL_15368006, EPI_ISL_15368007, EPI_ISL_15368008, EPI_ISL_15368009, EPI_ISL_15368010, EPI_ISL_15368011			
see above	Laboratory Medicine, UW Virology	Laboratory Medicine, UW Virology	Sereewit,J., Xie,H., Roychoudhury,P. and Greninger,A.L.
EPI_ISL_15370026, EPI_ISL_15370027, EPI_ISL_15370028, EPI_ISL_15370030, EPI_ISL_15370031, EPI_ISL_15370043, EPI_ISL_15370044, EPI_ISL_15370045, EPI_ISL_15370047, EPI_ISL_15370048, EPI_ISL_15370050, EPI_ISL_15370053, EPI_ISL_15370056, EPI_ISL_15370057, EPI_ISL_15370058, EPI_ISL_15370059, EPI_ISL_15370060, EPI_ISL_15370061			
see above	Laboratory for Diagnostics of Zoonoses and WHO Centre, Institute of Microbiology and Immunology, Faculty of Medicine, University of Ljubljana	Laboratory for Diagnostics of Zoonoses and WHO Centre, Institute of Microbiology and Immunology, Faculty of Medicine, University of Ljubljana	Zakotnik,S., Vlaj,D., Suljic,A., Zorec,T.M., Korva,M., Poljak,M. and Avsic Zupanc,T.
EPI_ISL_15370065, EPI_ISL_15370066, EPI_ISL_15370068, EPI_ISL_15370069, EPI_ISL_15370070, EPI_ISL_15370071, EPI_ISL_15370072, EPI_ISL_15370073, EPI_ISL_15370074, EPI_ISL_15370075, EPI_ISL_15370076, EPI_ISL_15370077, EPI_ISL_15370078, EPI_ISL_15370079, EPI_ISL_15370080, EPI_ISL_15370081, EPI_ISL_15370082			
see above	Nigeria Centre for Disease Control, National Reference Laboratory	Chemical, Biological and Radiological Sciences, Defence Science and Technology Laboratory	Ndodo,N., Ashcroft,J., Lewandowski,K., Yinka-Ogunleye,A., Chukwu,C., Ahmad,A., King,D., Akinpelu,A., Maluquer de Motes,C., Ribeca,P., Summer,R.P., Rambaut,A., Chester,M., Maishman,T., Babatunde,O., Mba,N., Babatunde,O., Aruna,O., Pullan,S.T., Gannon,B., Brown,C., Ihekweazu,C., Adetifa,I. and Ulaeto,D.O.
EPI_ISL_15373792	Department of immunology and microbiology - Pasteur Institute in Ho Chi Minh city	Department of immunology and microbiology - Pasteur Institute in Ho Chi Minh city	Manh H. Dao, Nhung H. P. Vu, Hang T. T. Pham, Thang M. Cao, Thinh V. Nguyen, Quang D. Pham, Quang C. Luong, Trung V. Nguyen
EPI_ISL_15380492	Eastwood Medical City	Molecular Biology Laboratory, Research Institute for Tropical Medicine	Samantha Louise P. Bado, Niquitta B. Galap, Bea C. Mateo, Chelsea Mae M. Reyes, Amalea Dulcene Nicolasora, Miguel Francisco B. Abulencia, Francisco Gerardo M. Polotan on behalf of the Research Institute for Tropical Medicine
EPI_ISL_15384398, EPI_ISL_15384399, EPI_ISL_15384400, EPI_ISL_15384401, EPI_ISL_15384402, EPI_ISL_15384403, EPI_ISL_15384404, EPI_ISL_15384405, EPI_ISL_15384406, EPI_ISL_15384407, EPI_ISL_15384408			
see above	Laboratory Medicine, UW Virology	Laboratory Medicine, UW Virology	Sereewit,J., Xie,H., Roychoudhury,P. and Greninger,A.L.
EPI_ISL_15384410	Sequencing/Bioinformatics, Delaware Public Health Lab	Sequencing/Bioinformatics, Delaware Public Health Lab	Bajwa,M.I. and Miller,H.
EPI_ISL_15384418, EPI_ISL_15384419, EPI_ISL_15384420, EPI_ISL_15384421, EPI_ISL_15384422, EPI_ISL_15384423, EPI_ISL_15384424, EPI_ISL_15384425, EPI_ISL_15384426, EPI_ISL_15384427, EPI_ISL_15384428, EPI_ISL_15384429, EPI_ISL_15384430, EPI_ISL_15384431, EPI_ISL_15384432, EPI_ISL_15384433, EPI_ISL_15384435, EPI_ISL_15384436, EPI_ISL_15384437, EPI_ISL_15384438, EPI_ISL_15384439			
see above	Centre for Biological Threats, Highly Pathogenic Viruses, Robert Koch Institute	Centre for Biological Threats, Highly Pathogenic Viruses, Robert Koch Institute	Brinkmann,A., Kohl,C., Pape,K., Schrick,L., Michel,J., Schaade,L. and Nitsche,A.
EPI_ISL_15390495, EPI_ISL_15390496, EPI_ISL_15390497, EPI_ISL_15390498, EPI_ISL_15390499, EPI_ISL_15390500, EPI_ISL_15390501	Centre for Biological Threats, Highly Pathogenic Viruses, Robert Koch Institute	Centre for Biological Threats, Highly Pathogenic Viruses, Robert Koch Institute	Brinkmann,A., Kohl,C., Pape,K., Uddin,S., Schrick,L., Michel,J., Schaade,L. and Nitsche,A.
EPI_ISL_15390502	Division of High-Consequence Pathogens and Pathology (DHCPP-PRB), CDC	Division of High-Consequence Pathogens and Pathology (DHCPP-PRB), CDC	Gigante,C.M., Pavlick,J., Zhao,H., Batra,D., Hetrick,E.E., Howard,D.T., Kovar,L., Seabolt,M.H., Weigand,M.R., Burroughs,M., Lee,J., Wilkins,K., McCollum,A., Hutson,C., Davidson,W., Rao,A., Parrott,T. and Li,Y.
EPI_ISL_15412318, EPI_ISL_15412320, EPI_ISL_15412322, EPI_ISL_15412324, EPI_ISL_15412326, EPI_ISL_15412328, EPI_ISL_15412330, EPI_ISL_15412331, EPI_ISL_15412333, EPI_ISL_15412335, EPI_ISL_15412336, EPI_ISL_15412338			
see above	CT Department of Public Health	CT Department of Public Health	Claire Pearson, Tu N. Nguyen, Kutluhan Incekara, Neranjan V. Perera
EPI_ISL_15415308	Hospital Clínica Bíblica	Inciensa, Instituto de Investigación y Enseñanza en Nutrición y Salud	Francisco Duarte, Ana Isela Ruiz-Gonzalez, Hillary Serrano, Diana Cantillo, Claudio Soto-Garita, Gustavo Vega, Estela Cordero, Adriana Godinez & Melany Calderon
EPI_ISL_15417986, EPI_ISL_15417987, EPI_ISL_15417989, EPI_ISL_15417990, EPI_ISL_15417991, EPI_ISL_15417992, EPI_ISL_15417993, EPI_ISL_15417994, EPI_ISL_15417995, EPI_ISL_15417996, EPI_ISL_15417997, EPI_ISL_15417998, EPI_ISL_15417999, EPI_ISL_15418000, EPI_ISL_15418001, EPI_ISL_15418002, EPI_ISL_15418003, EPI_ISL_15418004, EPI_ISL_15418005, EPI_ISL_15418006, EPI_ISL_15418007, EPI_ISL_15418008, EPI_ISL_15418009, EPI_ISL_15418010, EPI_ISL_15418011, EPI_ISL_15418012			
see above	Los Angeles County Public Health Laboratories	Los Angeles County Public Health Laboratories	P. Hemarajata et al.
EPI_ISL_15419131	Instituto de Infectologia Emilio Ribas	Instituto Adolfo Lutz Strategic Laboratory	Claudio Tavares Sacchi, Karoline Rodrigues Campos, Ariadne Ferreira Amarante, Marlon Benedito Nascimento Santos, Adriano Abbud, Adriana Bugno
EPI_ISL_15419132	Centro de Saude de Sao Roque Dr Jose Carvalho Brito	Instituto Adolfo Lutz Strategic Laboratory	Claudio Tavares Sacchi, Karoline Rodrigues Campos, Ariadne Ferreira Amarante, Marlon Benedito Nascimento Santos, Adriano Abbud, Adriana Bugno
EPI_ISL_15419133	UBS II COHAB Presidente Prudente	Instituto Adolfo Lutz Strategic Laboratory	Claudio Tavares Sacchi, Karoline Rodrigues Campos, Ariadne Ferreira Amarante, Marlon Benedito Nascimento Santos, Adriano Abbud, Adriana Bugno
EPI_ISL_15419134	CTA Centro de Testagem e Aconselhamento de Caieiras	Instituto Adolfo Lutz Strategic Laboratory	Claudio Tavares Sacchi, Karoline Rodrigues Campos, Ariadne Ferreira Amarante, Marlon Benedito Nascimento Santos, Adriano Abbud, Adriana Bugno
EPI_ISL_15419135	SAE DST AIDS Cidade Dutra	Instituto Adolfo Lutz Strategic Laboratory	Claudio Tavares Sacchi, Karoline Rodrigues Campos, Ariadne Ferreira Amarante, Marlon Benedito Nascimento Santos, Adriano Abbud, Adriana Bugno
EPI_ISL_15419136	UBS J Nordeste	Instituto Adolfo Lutz Strategic Laboratory	Claudio Tavares Sacchi, Karoline Rodrigues Campos, Ariadne Ferreira Amarante, Marlon Benedito Nascimento Santos, Adriano Abbud, Adriana Bugno
EPI_ISL_15419137	CTA Centro de Testagem e Aconselhamento Favo de Mel	Instituto Adolfo Lutz Strategic Laboratory	Claudio Tavares Sacchi, Karoline Rodrigues Campos, Ariadne Ferreira Amarante, Marlon Benedito Nascimento Santos, Adriano Abbud, Adriana Bugno
EPI_ISL_15419138	Vigilancia Epidemiologica e Controle de Vetores de Pirassununga	Instituto Adolfo Lutz Strategic Laboratory	Claudio Tavares Sacchi, Karoline Rodrigues Campos, Ariadne Ferreira Amarante, Marlon Benedito Nascimento Santos, Adriano Abbud, Adriana Bugno
EPI_ISL_15419139	Policlinica Maria Dirce	Instituto Adolfo Lutz Strategic Laboratory	Claudio Tavares Sacchi, Karoline Rodrigues Campos, Ariadne Ferreira Amarante, Marlon Benedito Nascimento Santos, Adriano Abbud, Adriana Bugno
EPI_ISL_15419140	Secretaria Municipal de Saude de Batatais SP	Instituto Adolfo Lutz Strategic Laboratory	Claudio Tavares Sacchi, Karoline Rodrigues Campos, Ariadne Ferreira Amarante, Marlon Benedito Nascimento Santos, Adriano Abbud, Adriana Bugno
EPI_ISL_15419141	UBS J Nordeste	Instituto Adolfo Lutz Strategic Laboratory	Claudio Tavares Sacchi, Karoline Rodrigues Campos, Ariadne Ferreira Amarante, Marlon Benedito Nascimento Santos, Adriano Abbud, Adriana Bugno
EPI_ISL_15419142	Instituto de Infectologia Emilio Ribas	Instituto Adolfo Lutz Strategic Laboratory	Claudio Tavares Sacchi, Karoline Rodrigues Campos, Ariadne Ferreira Amarante, Marlon Benedito Nascimento Santos, Adriano Abbud, Adriana Bugno
EPI_ISL_15419143	Santa Casa de Barretos	Instituto Adolfo Lutz Strategic Laboratory	Claudio Tavares Sacchi, Karoline Rodrigues Campos, Ariadne Ferreira Amarante, Marlon Benedito Nascimento Santos, Adriano Abbud, Adriana Bugno
EPI_ISL_15419144	Hospital Vera Cruz	Instituto Adolfo Lutz Strategic Laboratory	Claudio Tavares Sacchi, Karoline Rodrigues Campos, Ariadne Ferreira Amarante, Marlon Benedito Nascimento Santos, Adriano Abbud, Adriana Bugno
EPI_ISL_15419145	NotreDame Intermedica Saude	Instituto Adolfo Lutz Strategic Laboratory	Claudio Tavares Sacchi, Karoline Rodrigues Campos, Ariadne Ferreira Amarante, Marlon Benedito Nascimento Santos, Adriano Abbud, Adriana Bugno
EPI_ISL_15419146	Hospital e Maternidade Santa Maria Cruz Azul	Instituto Adolfo Lutz Strategic Laboratory	Claudio Tavares Sacchi, Karoline Rodrigues Campos, Ariadne Ferreira Amarante, Marlon Benedito Nascimento Santos, Adriano Abbud, Adriana Bugno
EPI_ISL_15419147	Pronto Socorro Central de Diadema	Instituto Adolfo Lutz Strategic Laboratory	Claudio Tavares Sacchi, Karoline Rodrigues Campos, Ariadne Ferreira Amarante, Marlon Benedito Nascimento Santos, Adriano Abbud, Adriana Bugno
EPI_ISL_15419148	NotreDame Intermedica Saude Santo Andre	Instituto Adolfo Lutz Strategic Laboratory	Claudio Tavares Sacchi, Karoline Rodrigues Campos, Ariadne Ferreira Amarante, Marlon Benedito Nascimento Santos, Adriano Abbud, Adriana Bugno
EPI_ISL_15419149	UBS Dom Angelico	Instituto Adolfo Lutz Strategic Laboratory	Claudio Tavares Sacchi, Karoline Rodrigues Campos, Ariadne Ferreira Amarante, Marlon Benedito Nascimento Santos, Adriano Abbud, Adriana Bugno
EPI_ISL_15419150	Secao de Centro de Diagnostico SECEDI	Instituto Adolfo Lutz Strategic Laboratory	Claudio Tavares Sacchi, Karoline Rodrigues Campos, Ariadne Ferreira Amarante, Marlon Benedito Nascimento Santos, Adriano Abbud, Adriana Bugno
EPI_ISL_15419151	UPA 24H Brotas	Instituto Adolfo Lutz Strategic Laboratory	Claudio Tavares Sacchi, Karoline Rodrigues Campos, Ariadne Ferreira Amarante, Marlon Benedito Nascimento Santos, Adriano Abbud, Adriana Bugno
EPI_ISL_15419152	Hospital Nossa Senhora de Lourdes	Instituto Adolfo Lutz Strategic Laboratory	Claudio Tavares Sacchi, Karoline Rodrigues Campos, Ariadne Ferreira Amarante, Marlon Benedito Nascimento Santos, Adriano Abbud, Adriana Bugno
EPI_ISL_15419153	AMA Paraisopolis	Instituto Adolfo Lutz Strategic Laboratory	Claudio Tavares Sacchi, Karoline Rodrigues Campos, Ariadne Ferreira Amarante, Marlon Benedito Nascimento Santos, Adriano Abbud, Adriana Bugno
EPI_ISL_15419154	Pronto Atendimento Infantil e Central de Quimioterapia de Sao Jose do Rio Preto	Instituto Adolfo Lutz Strategic Laboratory	Claudio Tavares Sacchi, Karoline Rodrigues Campos, Ariadne Ferreira Amarante, Marlon Benedito Nascimento Santos, Adriano Abbud, Adriana Bugno
EPI_ISL_15419155	Santa Casa de Atibaia Pro Saude	Instituto Adolfo Lutz Strategic Laboratory	Claudio Tavares Sacchi, Karoline Rodrigues Campos, Ariadne Ferreira Amarante, Marlon Benedito Nascimento Santos, Adriano Abbud, Adriana Bugno
EPI_ISL_15419156	UPA Vila Mariana	Instituto Adolfo Lutz Strategic Laboratory	Claudio Tavares Sacchi, Karoline Rodrigues Campos, Ariadne Ferreira Amarante, Marlon Benedito Nascimento Santos, Adriano Abbud, Adriana Bugno
EPI_ISL_15419157	Secretaria de Saude de Mogi das Cruzes	Instituto Adolfo Lutz Strategic Laboratory	Claudio Tavares Sacchi, Karoline Rodrigues Campos, Ariadne Ferreira Amarante, Marlon Benedito Nascimento Santos, Adriano Abbud, Adriana Bugno
EPI_ISL_15419158	Unidade Mista de saude Mariano Gayoso Castelo Branco	Instituto Adolfo Lutz Strategic Laboratory	Claudio Tavares Sacchi, Karoline Rodrigues Campos, Ariadne Ferreira Amarante, Marlon Benedito Nascimento Santos, Adriano Abbud, Adriana Bugno
EPI_ISL_15419159	Hospital Vivalle	Instituto Adolfo Lutz Strategic Laboratory	Claudio Tavares Sacchi, Karoline Rodrigues Campos, Ariadne Ferreira Amarante, Marlon Benedito Nascimento Santos, Adriano Abbud, Adriana Bugno
EPI_ISL_15419160	UPA Nova Hortolandia Manoel Geogino Lopes	Instituto Adolfo Lutz Strategic Laboratory	Claudio Tavares Sacchi, Karoline Rodrigues Campos, Ariadne Ferreira Amarante, Marlon Benedito Nascimento Santos, Adriano Abbud, Adriana Bugno
EPI_ISL_15419161	Centro de Saude I Albertino Affonso Jaboticabal	Instituto Adolfo Lutz Strategic Laboratory	Claudio Tavares Sacchi, Karoline Rodrigues Campos, Ariadne Ferreira Amarante, Marlon Benedito Nascimento Santos, Adriano Abbud, Adriana Bugno
EPI_ISL_15419162	SAE DST AIDS M Boi Mirim Servico de Atencao Especializada	Instituto Adolfo Lutz Strategic Laboratory	Claudio Tavares Sacchi, Karoline Rodrigues Campos, Ariadne Ferreira Amarante, Marlon Benedito Nascimento Santos, Adriano Abbud, Adriana Bugno

EPI_ISL_15419163	Instituto de Infectologia Emilio Ribas	Instituto Adolfo Lutz Strategic Laboratory	Claudio Tavares Sacchi, Karoline Rodrigues Campos, Ariadne Ferreira Amarante, Marlon Benedito Nascimento Santos, Adriano Abbud, Adriana Bugno
EPI_ISL_15433343, EPI_ISL_15433344, EPI_ISL_15433345, EPI_ISL_15433346, EPI_ISL_15433347, EPI_ISL_15433348	Laboratory Medicine, UW Virology	Laboratory Medicine, UW Virology	Sereewit,J., Xie,H., Roychoudhury,P. and Greninger,A.L.
EPI_ISL_15455851	Centers for Disease Control & Prevention (CDC), Division of High Consequence Pathogens and Pathology (DHCPP-PRB)	Centers for Disease Control & Prevention (CDC), Division of High Consequence Pathogens and Pathology (DHCPP-PRB)	Gigante,C.M., Francis,D., Zhao,H., Batra,D., Hetrick,E.E., Howard,D.T., Kovar,L., Morrison,S.S., Seabolt,M.H., Weigand,M.R., Knipe,K., Sheth,M., Burgin,A., Burroughs,M., Lee,J., Wilkins,K., McCollum,A., Hutson,C., Davidson,W., Rao,A., Escobar,J. and Li,Y.
EPI_ISL_15455852, EPI_ISL_15455853	Centers for Disease Control & Prevention (CDC), Division of High Consequence Pathogens and Pathology (DHCPP-PRB)	Centers for Disease Control & Prevention (CDC), Division of High Consequence Pathogens and Pathology (DHCPP-PRB)	Gigante,C.M., Ventura,J., Zhao,H., Batra,D., Hetrick,E.E., Howard,D.T., Kovar,L., Morrison,S.S., Seabolt,M.H., Weigand,M.R., Knipe,K., Sheth,M., Burgin,A., Burroughs,M., Lee,J., Wilkins,K., McCollum,A., Hutson,C., Davidson,W., Rao,A., Nash,J. and Li,Y.
EPI_ISL_15455854	Centers for Disease Control & Prevention (CDC), Division of High Consequence Pathogens and Pathology (DHCPP-PRB)	Centers for Disease Control & Prevention (CDC), Division of High Consequence Pathogens and Pathology (DHCPP-PRB)	Gigante,C.M., Maloney,M., Zhao,H., Batra,D., Hetrick,E.E., Howard,D.T., Kovar,L., Morrison,S.S., Seabolt,M.H., Weigand,M.R., Knipe,K., Sheth,M., Burgin,A., Burroughs,M., Lee,J., Wilkins,K., McCollum,A., Hutson,C., Davidson,W., Rao,A., Pearson,C. and Li,Y.
EPI_ISL_15455855, EPI_ISL_15455856, EPI_ISL_15455857	Centers for Disease Control & Prevention (CDC), Division of High Consequence Pathogens and Pathology (DHCPP-PRB)	Centers for Disease Control & Prevention (CDC), Division of High Consequence Pathogens and Pathology (DHCPP-PRB)	Gigante,C.M., Lee,P., Zhao,H., Batra,D., Hetrick,E.E., Howard,D.T., Kovar,L., Morrison,S.S., Seabolt,M.H., Weigand,M.R., Knipe,K., Sheth,M., Burgin,A., Burroughs,M., Lee,J., Wilkins,K., McCollum,A., Hutson,C., Davidson,W., Rao,A., Stanek,D. and Li,Y.
EPI_ISL_15455858	Centers for Disease Control & Prevention (CDC), Division of High Consequence Pathogens and Pathology (DHCPP-PRB)	Centers for Disease Control & Prevention (CDC), Division of High Consequence Pathogens and Pathology (DHCPP-PRB)	Gigante,C.M., Johnson,S., Zhao,H., Batra,D., Hetrick,E.E., Howard,D.T., Kovar,L., Morrison,S.S., Seabolt,M.H., Weigand,M.R., Knipe,K., Sheth,M., Burgin,A., Burroughs,M., Lee,J., Wilkins,K., McCollum,A., Hutson,C., Davidson,W., Rao,A., Riner,D. and Li,Y.
EPI_ISL_15455859	Centers for Disease Control & Prevention (CDC), Division of High Consequence Pathogens and Pathology (DHCPP-PRB)	Centers for Disease Control & Prevention (CDC), Division of High Consequence Pathogens and Pathology (DHCPP-PRB)	Gigante,C.M., Ostadkar,R., Zhao,H., Batra,D., Hetrick,E.E., Howard,D.T., Kovar,L., Morrison,S.S., Seabolt,M.H., Weigand,M.R., Knipe,K., Sheth,M., Burgin,A., Burroughs,M., Lee,J., Wilkins,K., McCollum,A., Hutson,C., Davidson,W., Rao,A., Wang,X. and Li,Y.
EPI_ISL_15455860	Centers for Disease Control & Prevention (CDC), Division of High Consequence Pathogens and Pathology (DHCPP-PRB)	Centers for Disease Control & Prevention (CDC), Division of High Consequence Pathogens and Pathology (DHCPP-PRB)	Gigante,C.M., Pettit,D., Zhao,H., Batra,D., Hetrick,E.E., Howard,D.T., Kovar,L., Morrison,S.S., Seabolt,M.H., Weigand,M.R., Knipe,K., Sheth,M., Burgin,A., Burroughs,M., Lee,J., Wilkins,K., McCollum,A., Hutson,C., Davidson,W., Rao,A., Deutsch-Feldman,M. and Li,Y.
EPI_ISL_15455861	Centers for Disease Control & Prevention (CDC), Division of High Consequence Pathogens and Pathology (DHCPP-PRB)	Centers for Disease Control & Prevention (CDC), Division of High Consequence Pathogens and Pathology (DHCPP-PRB)	Gigante,C.M., Pettit,D., Zhao,H., Batra,D., Hetrick,E.E., Howard,D.T., Kovar,L., Morrison,S.S., Seabolt,M.H., Weigand,M.R., Knipe,K., Sheth,M., Burgin,A., Burroughs,M., Lee,J., Wilkins,K., McCollum,A., Hutson,C., Davidson,W., Rao,A., Deutsch-Feldman,M. and Li,Y
EPI_ISL_15455862, EPI_ISL_15455863	Centers for Disease Control & Prevention (CDC), Division of High Consequence Pathogens and Pathology (DHCPP-PRB)	Centers for Disease Control & Prevention (CDC), Division of High Consequence Pathogens and Pathology (DHCPP-PRB)	Gigante,C.M., Acheampong,E., Zhao,H., Batra,D., Hetrick,E.E., Howard,D.T., Kovar,L., Morrison,S.S., Seabolt,M.H., Weigand,M.R., Knipe,K., Sheth,M., Burgin,A., Burroughs,M., Lee,J., Wilkins,K., McCollum,A., Hutson,C., Davidson,W., Rao,A., McDermott,D. and Li,Y.
EPI_ISL_15455864, EPI_ISL_15455865, EPI_ISL_15455866	Centers for Disease Control & Prevention (CDC), Division of High Consequence Pathogens and Pathology (DHCPP-PRB)	Centers for Disease Control & Prevention (CDC), Division of High Consequence Pathogens and Pathology (DHCPP-PRB)	Gigante,C.M., Ruiz,V., Zhao,H., Batra,D., Hetrick,E.E., Howard,D.T., Kovar,L., Morrison,S.S., Seabolt,M.H., Weigand,M.R., Knipe,K., Sheth,M., Burgin,A., Burroughs,M., Lee,J., Wilkins,K., McCollum,A., Hutson,C., Davidson,W., Rao,A., Wang,J. and Li,Y.
EPI_ISL_15455867	Centers for Disease Control & Prevention (CDC), Division of High Consequence Pathogens and Pathology (DHCPP-PRB)	Centers for Disease Control & Prevention (CDC), Division of High Consequence Pathogens and Pathology (DHCPP-PRB)	Gigante,C.M., Lee,B., Zhao,H., Batra,D., Hetrick,E.E., Howard,D.T., Kovar,L., Morrison,S.S., Seabolt,M.H., Weigand,M.R., Knipe,K., Sheth,M., Burgin,A., Burroughs,M., Lee,J., Wilkins,K., McCollum,A., Hutson,C., Davidson,W., Rao,A., Salehi,E. and Li,Y.
EPI_ISL_15455868	Centers for Disease Control & Prevention (CDC), Division of High Consequence Pathogens and Pathology (DHCPP-PRB)	Centers for Disease Control & Prevention (CDC), Division of High Consequence Pathogens and Pathology (DHCPP-PRB)	Gigante,C.M., Berns,A., Zhao,H., Batra,D., Hetrick,E.E., Howard,D.T., Kovar,L., Morrison,S.S., Seabolt,M.H., Weigand,M.R., Knipe,K., Sheth,M., Burgin,A., Burroughs,M., Lee,J., Wilkins,K., McCollum,A., Hutson,C., Davidson,W., Rao,A., Carpenter-Azevedo,K. and Li,Y.
EPI_ISL_15455869, EPI_ISL_15455870	Centers for Disease Control & Prevention (CDC), Division of High Consequence Pathogens and Pathology (DHCPP-PRB)	Centers for Disease Control & Prevention (CDC), Division of High Consequence Pathogens and Pathology (DHCPP-PRB)	Gigante,C.M., Kubin,G., Zhao,H., Batra,D., Hetrick,E.E., Howard,D.T., Kovar,L., Morrison,S.S., Seabolt,M.H., Weigand,M.R., Knipe,K., Sheth,M., Burgin,A., Burroughs,M., Lee,J., Wilkins,K., McCollum,A., Hutson,C., Davidson,W., Rao,A., White,S.L. and Li,Y.
EPI_ISL_15455871, EPI_ISL_15455872, EPI_ISL_15455873, EPI_ISL_15455874	Centers for Disease Control & Prevention (CDC), Division of High Consequence Pathogens and Pathology (DHCPP-PRB)	Centers for Disease Control & Prevention (CDC), Division of High Consequence Pathogens and Pathology (DHCPP-PRB)	Brinkmann,A., Kohl,C., Pape,K., Schrick,L., Michel,J., Schaade,L. and Nitsche,A.
EPI_ISL_15455875, EPI_ISL_15455876, EPI_ISL_15455877, EPI_ISL_15455878, EPI_ISL_15455879, EPI_ISL_15455880, EPI_ISL_15455881, EPI_ISL_15455882, EPI_ISL_15455883, EPI_ISL_15455884, EPI_ISL_15455885, EPI_ISL_15455886, EPI_ISL_15455887, EPI_ISL_15455888, EPI_ISL_15455889, EPI_ISL_15455890, EPI_ISL_15455891, EPI_ISL_15455892, EPI_ISL_15455893, EPI_ISL_15455894, EPI_ISL_15455895, EPI_ISL_15455896, EPI_ISL_15455897, EPI_ISL_15455898, EPI_ISL_15455899, EPI_ISL_15455900, EPI_ISL_15455901, EPI_ISL_15455902, EPI_ISL_15455903, EPI_ISL_15455904, EPI_ISL_15455905, EPI_ISL_15455906, EPI_ISL_15455907			
see above	Centre for Biological Threats, Highly Pathogenic Viruses, Robert Koch Institute	Centre for Biological Threats, Highly Pathogenic Viruses, Robert Koch Institute	Brinkmann,A., Kohl,C., Pape,K., Schrick,L., Michel,J., Schaade,L. and Nitsche,A.
EPI_ISL_15455914, EPI_ISL_15455916, EPI_ISL_15455917	Direccion de Investigacion en Salud Publica, Instituto Nacional de Salud	Direccion de Investigacion en Salud Publica, Instituto Nacional de Salud	Laiton-Donato,K., Alvarez-Diaz,D.A., Franco-Munoz,C., Ruiz-Moreno,H.A., Rojas-Estevez,P., Rosales,A., Martinez,D., Flores,A., Prieto,F., Walteros,D., Gomez,S. and Mercado-Reyes,M.
EPI_ISL_15458903, EPI_ISL_15458904, EPI_ISL_15458905, EPI_ISL_15458906	Nebraska Public Health Laboratory (NPHL)	Environmental, Agricultural, and Occupational Health, University of Nebraska Medical Center	Chapman,R.C., Bernhard,K., McCutchen,E.L., Fauver,J.R., O'Dell,J.X., Mannell,M., Wiley,M.R. and Cross,S.T.
EPI_ISL_15458907, EPI_ISL_15500242, EPI_ISL_15500243	Laboratory Medicine, UW Virology	Laboratory Medicine, UW Virology	Sereewit,J., Xie,H., Roychoudhury,P. and Greninger,A.L.
EPI_ISL_15500244, EPI_ISL_15500245, EPI_ISL_15500246, EPI_ISL_15500247, EPI_ISL_15500248, EPI_ISL_15500249, EPI_ISL_15500250, EPI_ISL_15500251, EPI_ISL_15500252, EPI_ISL_15500253, EPI_ISL_15500254, EPI_ISL_15500255, EPI_ISL_15500256, EPI_ISL_15500257, EPI_ISL_15500258, EPI_ISL_15500259, EPI_ISL_15500260, EPI_ISL_15500261, EPI_ISL_15500262, EPI_ISL_15500263, EPI_ISL_15500264			
see above	Centre for Biological Threats, Highly Pathogenic Viruses, Robert Koch Institute	Centre for Biological Threats, Highly Pathogenic Viruses, Robert Koch Institute	Brinkmann,A., Kohl,C., Pape,K., Schrick,L., Michel,J., Schaade,L. and Nitsche,A.
EPI_ISL_15502320, EPI_ISL_15502321, EPI_ISL_15502322, EPI_ISL_15502324, EPI_ISL_15502325, EPI_ISL_15502326, EPI_ISL_15502327, EPI_ISL_15502328, EPI_ISL_15502329, EPI_ISL_15502330, EPI_ISL_15502331, EPI_ISL_15502332, EPI_ISL_15502333, EPI_ISL_15502334, EPI_ISL_15502335, EPI_ISL_15502336, EPI_ISL_15502337, EPI_ISL_15502338, EPI_ISL_15502339, EPI_ISL_15502340, EPI_ISL_15502341, EPI_ISL_15502342, EPI_ISL_15502343			
see above	Los Angeles County Public Health Laboratories	Los Angeles County Public Health Laboratories	P. Hemarajata et al.
EPI_ISL_15528148	Department of Clinical Sciences, Institute of Tropica Medicine	Department of Clinical Sciences, Institute of Tropica Medicine	Berens-Riha,N., De Block,T., Rutgers,J., Van Gestel,L., Hens,M., Kenyon,C., Soentjens,P., Van Griensven,J., Brosius,I., Arien,K., Van Esbroeck,M., Rezende,A.M., Vercauteren,K. and Liesenborghs,L.
EPI_ISL_15528149, EPI_ISL_15528150, EPI_ISL_15528151	Department of Clinical Sciences, Institute of Tropica Medicine	Department of Clinical Sciences, Institute of Tropica Medicine	Berens-Riha,N., De Block,T., Rutgers,J., Van Gestel,L., Hens,M., Kenyon,C., Soentjens,P., Van Griensven,J., Brosius,I., Arien,K., Van Esbroeck,M., Rezende,A.M. and Vercauteren,K.
EPI_ISL_15587960, EPI_ISL_15587961, EPI_ISL_15587962, EPI_ISL_15587963, EPI_ISL_15587964, EPI_ISL_15587965, EPI_ISL_15587966, EPI_ISL_15587967, EPI_ISL_15587968, EPI_ISL_15587969	Utah Public Health Laboratory	Utah Public Health Laboratory	Iverson,T., Oakeson,K.F., Young,E.L., Wagner,J. and Hergert,J.
EPI_ISL_15593715	División Diagnóstico Molecular Hospital México	División Diagnóstico Molecular Hospital México	Juan Carlos Villalobos Ugalde, Vanessa Villalobos Alfaro, Carlos Ramirez Chavarria
EPI_ISL_15593717	LESP State of Mexico	Instituto de Diagnostico y Referencia Epidemiologicos (INDRE)	Abril Rodríguez-Maldonado; Claudia Wong-Arámbula; Felipe Arguijo-Perez; Helios Cárdenas-Hernández; Carmen Castro-Méndez; Lidia García-Torres; Ruth Madera-Sandoval; América Mandujano-Martínez; Nancy Martínez-Velázquez; Mireya Mederos-Michel; Angélica Pedraza-Meléndez; Joaquín Quiroz-Mercado; Daniel Regalado-Santiago; Silvia Rivero-Arredondo; Erika Sierra-Atanacio; Fernando González-Domínguez; Lucia Hernández-Rivas, Irma López-Martínez; Ernesto Ramírez-González; Maribel González-Villa
EPI_ISL_15593718	LESP Mexico City	Instituto de Diagnostico y Referencia Epidemiologicos (INDRE)	Abril Rodríguez-Maldonado; Claudia Wong-Arámbula; Felipe Arguijo-Perez; Helios Cárdenas-Hernández; Carmen Castro-Méndez; Lidia García-Torres; Ruth Madera-Sandoval; América Mandujano-Martínez; Nancy Martínez-Velázquez; Mireya Mederos-Michel; Angélica Pedraza-Meléndez; Joaquín Quiroz-Mercado; Daniel Regalado-Santiago; Silvia Rivero-Arredondo; Erika Sierra-Atanacio; Fernando González-Domínguez; Lucia Hernández-Rivas, Irma López-Martínez; Ernesto Ramírez-González; Maribel González-Villa
EPI_ISL_15593719	LESP Puebla	Instituto de Diagnostico y Referencia Epidemiologicos (INDRE)	Abril Rodríguez-Maldonado; Claudia Wong-Arámbula; Felipe Arguijo-Perez; Helios Cárdenas-Hernández; Carmen Castro-Méndez; Lidia García-Torres; Ruth Madera-Sandoval; América Mandujano-Martínez; Nancy Martínez-Velázquez; Mireya Mederos-Michel; Angélica Pedraza-Meléndez; Joaquín Quiroz-Mercado; Daniel Regalado-Santiago; Silvia Rivero-Arredondo; Erika Sierra-Atanacio; Fernando González-Domínguez; Lucia Hernández-Rivas, Irma López-Martínez; Ernesto Ramírez-González; Maribel González-Villa
EPI_ISL_15593720	LESP Tamaulipas	Instituto de Diagnostico y Referencia Epidemiologicos (INDRE)	Abril Rodríguez-Maldonado; Claudia Wong-Arámbula; Felipe Arguijo-Perez; Helios Cárdenas-Hernández; Carmen Castro-Méndez; Lidia García-Torres; Ruth Madera-Sandoval; América Mandujano-Martínez; Nancy Martínez-Velázquez; Mireya Mederos-Michel; Angélica Pedraza-Meléndez; Joaquín Quiroz-Mercado; Daniel Regalado-Santiago; Silvia Rivero-Arredondo; Erika Sierra-Atanacio; Fernando González-Domínguez; Lucia Hernández-Rivas, Irma López-Martínez; Ernesto Ramírez-González; Maribel González-Villa
EPI_ISL_15593721	LESP Baja California	Instituto de Diagnostico y Referencia Epidemiologicos (INDRE)	Abril Rodríguez-Maldonado; Claudia Wong-Arámbula; Felipe Arguijo-Perez; Helios Cárdenas-Hernández; Carmen Castro-Méndez; Lidia García-Torres; Ruth Madera-Sandoval; América Mandujano-Martínez; Nancy Martínez-Velázquez; Mireya Mederos-Michel; Angélica Pedraza-Meléndez; Joaquín Quiroz-Mercado; Daniel Regalado-Santiago; Silvia Rivero-Arredondo; Erika Sierra-Atanacio; Fernando González-Domínguez; Lucia Hernández-Rivas, Irma López-Martínez; Ernesto Ramírez-González; Maribel González-Villa
EPI_ISL_15593722	LESP Nuevo Leon	Instituto de Diagnostico y Referencia Epidemiologicos (INDRE)	Abril Rodríguez-Maldonado; Claudia Wong-Arámbula; Felipe Arguijo-Perez; Helios Cárdenas-Hernández; Carmen Castro-Méndez; Lidia García-Torres; Ruth Madera-Sandoval; América Mandujano-Martínez; Nancy Martínez-Velázquez; Mireya Mederos-Michel; Angélica Pedraza-Meléndez; Joaquín Quiroz-Mercado; Daniel Regalado-Santiago; Silvia Rivero-Arredondo; Erika Sierra-Atanacio; Fernando González-Domínguez; Lucia Hernández-Rivas, Irma López-Martínez; Ernesto Ramírez-González; Maribel González-Villa
EPI_ISL_15597039	Los Angeles County Public Health Laboratories	Los Angeles County Public Health Laboratories	P. Hemarajata et al.
EPI_ISL_15597040, EPI_ISL_15597041, EPI_ISL_15597042, EPI_ISL_15597043,	UCLA Clinical Micro Lab	Los Angeles County Public Health Laboratories	P. Hemarajata et al.

EPI_ISL_15597044, EPI_ISL_15597045, EPI_ISL_15597046			
EPI_ISL_15597047, EPI_ISL_15597048, EPI_ISL_15597049, EPI_ISL_15597050, EPI_ISL_15597051, EPI_ISL_15597052, EPI_ISL_15597054, EPI_ISL_15597055, EPI_ISL_15597056	Quest Diagnostics Nichols Institute	Los Angeles County Public Health Laboratories	P. Hemarajata et al.
EPI_ISL_15597058, EPI_ISL_15597059, EPI_ISL_15597060, EPI_ISL_15597061, EPI_ISL_15597062, EPI_ISL_15597063	Los Angeles County Public Health Laboratories	Los Angeles County Public Health Laboratories	P. Hemarajata et al.
EPI_ISL_15607969, EPI_ISL_15607970, EPI_ISL_15607971, EPI_ISL_15607972, EPI_ISL_15607973, EPI_ISL_15607974, EPI_ISL_15607975, EPI_ISL_15607976, EPI_ISL_15607977, EPI_ISL_15607978, EPI_ISL_15607979, EPI_ISL_15607980, EPI_ISL_15607981, EPI_ISL_15607982, EPI_ISL_15607983, EPI_ISL_15607984, EPI_ISL_15607985, EPI_ISL_15607986, EPI_ISL_15607987, EPI_ISL_15607988, EPI_ISL_15607989, EPI_ISL_15607990, EPI_ISL_15607991, EPI_ISL_15608021, EPI_ISL_15608022, EPI_ISL_15608023, EPI_ISL_15608024, EPI_ISL_15608025, EPI_ISL_15608026, EPI_ISL_15608027, EPI_ISL_15608028, EPI_ISL_15608029, EPI_ISL_15608030, EPI_ISL_15608031, EPI_ISL_15608032, EPI_ISL_15608033, EPI_ISL_15608034, EPI_ISL_15608035, EPI_ISL_15608036, EPI_ISL_15608037, EPI_ISL_15608038, EPI_ISL_15608039, EPI_ISL_15608040, EPI_ISL_15608041, EPI_ISL_15608042, EPI_ISL_15608043, EPI_ISL_15608044, EPI_ISL_15608045, EPI_ISL_15608046, EPI_ISL_15608047, EPI_ISL_15608048, EPI_ISL_15608049, EPI_ISL_15608050, EPI_ISL_15608051, EPI_ISL_15608052, EPI_ISL_15608053, EPI_ISL_15608054, EPI_ISL_15608055, EPI_ISL_15608056, EPI_ISL_15608057, EPI_ISL_15608058, EPI_ISL_15608059			
see above	Laboratory Medicine, UW Virology	Laboratory Medicine, UW Virology	Sereewit,J., Xie,H., Roychoudhury,P. and Greninger,A.L.
EPI_ISL_16283050, EPI_ISL_16283051, EPI_ISL_16283052, EPI_ISL_16283053, EPI_ISL_16283054, EPI_ISL_16283055, EPI_ISL_16283056, EPI_ISL_16283057, EPI_ISL_16283058, EPI_ISL_16283059, EPI_ISL_16283060, EPI_ISL_16283061, EPI_ISL_16283062, EPI_ISL_16283063, EPI_ISL_16283064, EPI_ISL_16283065, EPI_ISL_16283066, EPI_ISL_16283067, EPI_ISL_16283068, EPI_ISL_16283069, EPI_ISL_16283070, EPI_ISL_16283071, EPI_ISL_16283072, EPI_ISL_16283073, EPI_ISL_16283074, EPI_ISL_16283075, EPI_ISL_16283076, EPI_ISL_16283077, EPI_ISL_16283078			
see above	National Reference Laboratory, Nigeria Centre for Disease Control	National Reference Laboratory, Nigeria Centre for Disease Control	Ndodo Nnaemeka, Olusola Anuoluwapo Akanbi, Chimaobi Chukwu, Oyeronke Ayansola, Adama Ahmad, Adesola Yinka-Ogunleye, Akinpelu Afolabi, Olajumoke Babatunde, Chikwe Ihekweazu, Ifedayo Adetifa, Matthew Mauldin, Crystal Gigante, Dhwani Batra, Michael Weigand, Dakota Howard, Mili Sheth, Matthew Seabolt, Yu Li
EPI_ISL_16283079	Nigeria Centre for Disease Control, National Reference Laboratory	Nigeria Centre for Disease Control, National Reference Laboratory	Ndodo Nnaemeka, Olusola Anuoluwapo Akanbi, Chimaobi Chukwu, Oyeronke Ayansola, Adama Ahmad, Adesola Yinka-Ogunleye, Akinpelu Afolabi, Olajumoke Babatunde, Chikwe Ihekweazu, Ifedayo Adetifa, Matthew Mauldin, Crystal Gigante, Dhwani Batra, Michael Weigand, Dakota Howard, Mili Sheth, Matthew Seabolt, Yu Li
