# Supplementary material for: Extraction-free LAMP assays for generic detection of Old World Orthopoxviruses and specific detection of Mpox virus
Source: Sci Rep. 2023 Nov 30;13:21093. doi: 10.1038/s41598-023-48391-z (PMC10689478; doi:10.1038/s41598-023-48391-z)
Supplement: Supplementary file 16 — Supplementary Table 4. [file 41598_2023_48391_MOESM16_ESM.pdf]

We gratefully acknowledge the following Authors from the Originating laboratories responsible for obtaining the specimens, as well as the Submitting laboratories where the genome data were generated and shared via GISAID, on which this research is based.

All Submitters of data may be contacted directly via www.gisaid.org

Authors are sorted alphabetically.

Accession ID	Originating Laboratory	Submitting Laboratory	Authors
EPI_ISL_15608908, EPI_ISL_15608909, EPI_ISL_15608910, EPI_ISL_15608911	Southern Nevada Public Health Laboratory	Southern Nevada Public Health Laboratory	Michael Picker
EPI_ISL_15617330, EPI_ISL_15617332, EPI_ISL_15617333	Sequencing/Bioinformatics, Delaware Public Health Lab	Sequencing/Bioinformatics, Delaware Public Health Lab	Bajwa,M.I. and Miller,H.
EPI_ISL_15617335	Centers for Disease Control & Prevention (CDC), Division of High Consequence Pathogens and Pathology (DHCPP-PRB)	Centers for Disease Control & Prevention (CDC), Division of High Consequence Pathogens and Pathology (DHCPP-PRB)	Gigante,C.M., Vang,K., Zhao,H., Batra,D., Hetrick,E.E., Howard,D.T., Kovar,L., Seabolt,M.H., Morrison,S.S., Desch,M., Knipe,K., Weigand,M.R., Sheth,M., Burgin,A., Burroughs,M., Lee,J., Wilkins,K., McCollum,A., Hutson,C., Davidson,W., Rao,A., Seely,K. and Li,Y.
EPI_ISL_15617336	Centers for Disease Control & Prevention (CDC), Division of High Consequence Pathogens and Pathology (DHCPP-PRB)	Centers for Disease Control & Prevention (CDC), Division of High Consequence Pathogens and Pathology (DHCPP-PRB)	Gigante,C.M., Francis,D., Zhao,H., Batra,D., Hetrick,E.E., Howard,D.T., Kovar,L., Seabolt,M.H., Morrison,S.S., Desch,M., Knipe,K., Weigand,M.R., Sheth,M., Burgin,A., Burroughs,M., Lee,J., Wilkins,K., McCollum,A., Hutson,C., Davidson,W., Rao,A., Escobar,J. and Li,Y.
EPI_ISL_15617337	Centers for Disease Control & Prevention (CDC), Division of High Consequence Pathogens and Pathology (DHCPP-PRB)	Centers for Disease Control & Prevention (CDC), Division of High Consequence Pathogens and Pathology (DHCPP-PRB)	Gigante,C.M., Ventura,J., Zhao,H., Batra,D., Hetrick,E.E., Howard,D.T., Kovar,L., Seabolt,M.H., Morrison,S.S., Desch,M., Knipe,K., Weigand,M.R., Sheth,M., Burgin,A., Burroughs,M., Lee,J., Wilkins,K., McCollum,A., Hutson,C., Davidson,W., Rao,A., Nash,J. and Li,Y.
EPI_ISL_15617338	Centers for Disease Control & Prevention (CDC), Division of High Consequence Pathogens and Pathology (DHCPP-PRB)	Centers for Disease Control & Prevention (CDC), Division of High Consequence Pathogens and Pathology (DHCPP-PRB)	Gigante,C.M., Lee,P., Zhao,H., Batra,D., Hetrick,E.E., Howard,D.T., Kovar,L., Seabolt,M.H., Morrison,S.S., Desch,M., Knipe,K., Weigand,M.R., Sheth,M., Burgin,A., Burroughs,M., Lee,J., Wilkins,K., McCollum,A., Hutson,C., Davidson,W., Rao,A., Stanek,D. and Li,Y.
EPI_ISL_15617339	Centers for Disease Control & Prevention (CDC), Division of High Consequence Pathogens and Pathology (DHCPP-PRB)	Centers for Disease Control & Prevention (CDC), Division of High Consequence Pathogens and Pathology (DHCPP-PRB)	Gigante,C.M., Pavlick,J., Zhao,H., Batra,D., Hetrick,E.E., Howard,D.T., Kovar,L., Seabolt,M.H., Morrison,S.S., Desch,M., Knipe,K., Weigand,M.R., Sheth,M., Burgin,A., Burroughs,M., Lee,J., Wilkins,K., McCollum,A., Hutson,C., Davidson,W., Rao,A., Parrott,T. and Li,Y.
EPI_ISL_15617340, EPI_ISL_15617341	Centers for Disease Control & Prevention (CDC), Division of High Consequence Pathogens and Pathology (DHCPP-PRB)	Centers for Disease Control & Prevention (CDC), Division of High Consequence Pathogens and Pathology (DHCPP-PRB)	Gigante,C.M., Fisher,S., Zhao,H., Batra,D., Hetrick,E.E., Howard,D.T., Kovar,L., Seabolt,M.H., Morrison,S.S., Desch,M., Knipe,K., Weigand,M.R., Sheth,M., Burgin,A., Burroughs,M., Lee,J., Wilkins,K., McCollum,A., Hutson,C., Davidson,W., Rao,A., Siebert,M. and Li,Y.
EPI_ISL_15617342, EPI_ISL_15617343, EPI_ISL_15617344, EPI_ISL_15617345	Centers for Disease Control & Prevention (CDC), Division of High Consequence Pathogens and Pathology (DHCPP-PRB)	Centers for Disease Control & Prevention (CDC), Division of High Consequence Pathogens and Pathology (DHCPP-PRB)	Gigante,C.M., Ruiz,V., Zhao,H., Batra,D., Hetrick,E.E., Howard,D.T., Kovar,L., Seabolt,M.H., Morrison,S.S., Desch,M., Knipe,K., Weigand,M.R., Sheth,M., Burgin,A., Burroughs,M., Lee,J., Wilkins,K., McCollum,A., Hutson,C., Davidson,W., Rao,A., Wang,J. and Li,Y.
EPI_ISL_15617346, EPI_ISL_15617347, EPI_ISL_15617348	Centers for Disease Control & Prevention (CDC), Division of High Consequence Pathogens and Pathology (DHCPP-PRB)	Centers for Disease Control & Prevention (CDC), Division of High Consequence Pathogens and Pathology (DHCPP-PRB)	Gigante,C.M., Cogswell,K., Zhao,H., Batra,D., Hetrick,E.E., Howard,D.T., Kovar,L., Seabolt,M.H., Morrison,S.S., Desch,M., Knipe,K., Weigand,M.R., Sheth,M., Burgin,A., Burroughs,M., Lee,J., Wilkins,K., McCollum,A., Hutson,C., Davidson,W., Rao,A., Grenz,L. and Li,Y.
EPI_ISL_15617349, EPI_ISL_15617350	Centers for Disease Control & Prevention (CDC), Division of High Consequence Pathogens and Pathology (DHCPP-PRB)	Centers for Disease Control & Prevention (CDC), Division of High Consequence Pathogens and Pathology (DHCPP-PRB)	Gigante,C.M., Epie,N., Zhao,H., Batra,D., Hetrick,E.E., Howard,D.T., Kovar,L., Seabolt,M.H., Morrison,S.S., Desch,M., Knipe,K., Weigand,M.R., Sheth,M., Burgin,A., Burroughs,M., Lee,J., Wilkins,K., McCollum,A., Hutson,C., Davidson,W., Rao,A., Perez,T. and Li,Y.
EPI_ISL_15617351, EPI_ISL_15617352	Centers for Disease Control & Prevention (CDC), Division of High Consequence Pathogens and Pathology (DHCPP-PRB)	Centers for Disease Control & Prevention (CDC), Division of High Consequence Pathogens and Pathology (DHCPP-PRB)	Gigante,C.M., Thomas,L., Zhao,H., Batra,D., Hetrick,E.E., Howard,D.T., Kovar,L., Seabolt,M.H., Morrison,S.S., Desch,M., Knipe,K., Weigand,M.R., Sheth,M., Burgin,A., Burroughs,M., Lee,J., Wilkins,K., McCollum,A., Hutson,C., Davidson,W., Rao,A., Dunn,J. and Li,Y.
EPI_ISL_15617353	Centers for Disease Control & Prevention (CDC), Division of High Consequence Pathogens and Pathology (DHCPP-PRB)	Centers for Disease Control & Prevention (CDC), Division of High Consequence Pathogens and Pathology (DHCPP-PRB)	Gigante,C.M., Kubin,G., Zhao,H., Batra,D., Hetrick,E.E., Howard,D.T., Kovar,L., Seabolt,M.H., Morrison,S.S., Desch,M., Knipe,K., Weigand,M.R., Sheth,M., Burgin,A., Burroughs,M., Lee,J., Wilkins,K., McCollum,A., Hutson,C., Davidson,W., Rao,A., White,S.L. and Li,Y.
EPI_ISL_15617354, EPI_ISL_15617355, EPI_ISL_15617356, EPI_ISL_15617357, EPI_ISL_15617358	Centers for Disease Control & Prevention (CDC), Division of High Consequence Pathogens and Pathology (DHCPP-PRB)	Centers for Disease Control & Prevention (CDC), Division of High Consequence Pathogens and Pathology (DHCPP-PRB)	Gigante,C.M., Goldoft,M., Zhao,H., Batra,D., Hetrick,E.E., Howard,D.T., Kovar,L., Seabolt,M.H., Morrison,S.S., Desch,M., Knipe,K., Weigand,M.R., Sheth,M., Burgin,A., Burroughs,M., Lee,J., Wilkins,K., McCollum,A., Hutson,C., Davidson,W., Rao,A., Holshue,M. and Li,Y.
EPI_ISL_15641541, EPI_ISL_15641542, EPI_ISL_15641543, EPI_ISL_15641544, EPI_ISL_15641545, EPI_ISL_15641546, EPI_ISL_15641547, EPI_ISL_15641548, EPI_ISL_15641549, EPI_ISL_15641550, EPI_ISL_15641551, EPI_ISL_15641552, EPI_ISL_15641553, EPI_ISL_15641554, EPI_ISL_15641555, EPI_ISL_15641556, EPI_ISL_15641557, EPI_ISL_15641559, EPI_ISL_15641560, EPI_ISL_15641561, EPI_ISL_15641562, EPI_ISL_15641563, EPI_ISL_15641564, EPI_ISL_15641565, EPI_ISL_15641566, EPI_ISL_15641567, EPI_ISL_15641568, EPI_ISL_15641569, EPI_ISL_15641570, EPI_ISL_15641571, EPI_ISL_15641572, EPI_ISL_15641573, EPI_ISL_15641574, EPI_ISL_15641576, EPI_ISL_15641577, EPI_ISL_15641578, EPI_ISL_15641579, EPI_ISL_15641580, EPI_ISL_15641581, EPI_ISL_15641582, EPI_ISL_15641583, EPI_ISL_15641584, EPI_ISL_15641585, EPI_ISL_15641586, EPI_ISL_15641587, EPI_ISL_15641588, EPI_ISL_15641589, EPI_ISL_15641590, EPI_ISL_15641591, EPI_ISL_15641592, EPI_ISL_15641593, EPI_ISL_15641594, EPI_ISL_15641595, EPI_ISL_15641596, EPI_ISL_15641597, EPI_ISL_15641598, EPI_ISL_15641599, EPI_ISL_15641600, EPI_ISL_15641601, EPI_ISL_15641602, EPI_ISL_15641603, EPI_ISL_15641604, EPI_ISL_15641605, EPI_ISL_15641606, EPI_ISL_15641607, EPI_ISL_15641608, EPI_ISL_15641609			
see above	Public Health Laboratory, Public Health Service Amsterdam, The Netherlands	Department of Medical Microbiology & Infection prevention, Amsterdam University Medical Centers location AMC	Matthijs Welkers, Jelle Koopsen, Robin van Houdt, Marcel Jonges, Sebastien Matamoros, Sjoerd Rebers, Fokla Zorgdrager, Sylvia Bruisten, Akke Cornelissen, Janke Schinkel, Ewout Fanoy, Roisin Bavalia, Menno de Jong and Mariken van der Lubben on behalf of the Amsterdam Regional Genomic epidemiology and Outbreak Surveillance (ARGOS) consortium
EPI_ISL_15651836, EPI_ISL_15651837, EPI_ISL_15651839, EPI_ISL_15651841, EPI_ISL_15651842, EPI_ISL_15651844, EPI_ISL_15651846, EPI_ISL_15651847, EPI_ISL_15651849, EPI_ISL_15651850, EPI_ISL_15651852, EPI_ISL_15651854, EPI_ISL_15651856, EPI_ISL_15651857, EPI_ISL_15651859, EPI_ISL_15651861			
see above	Centre for Biological Threats, Highly Pathogenic Viruses, Robert Koch Institute	Centre for Biological Threats, Highly Pathogenic Viruses, Robert Koch Institute	Brinkmann,A., Kohl,C., Pape,K., Schrick,L., Michel,J., Schaade,L. and Nitsche,A.
EPI_ISL_15655944	Erasmus Medical Center Department of Virology	Erasmus Medical Center Department of Virology	Leonard Schuele, Bas Oude Munnink, Marjan Boter, Babette Weller, Babs Verstrepen, Richard Molenkamp, Janette Rahamat-Langendoen, Reina Sikkema, Marion Koopmans
EPI_ISL_15684645	Laboratory of Virology, University Hospitals of Geneva	Laboratory of Virology, University Hospitals of Geneva	Laubscher,F., Marques-Melancia,S., Cordey,S., Schibler,M.,Kaiser,L. and Renzoni,A.
EPI_ISL_15701569	Institute for Medical Virology, University Hospital, Goethe University Frankfurt, Germany	Institute of Virology, Charité-Universitätsmedizin Berlin, Humboldt-Universität zu Berlin, Germany	Denisa Bojkova, Julia Schneider, Victor M. Corman, Jindrich Cinatl jr.
EPI_ISL_15702015, EPI_ISL_15702711	Erasmus Medical Center Department of Virology	Erasmus Medical Center Department of Virology	Leonard Schuele, Bas Oude Munnink, Marjan Boter, Babette Weller, Babs Verstrepen, Richard Molenkamp, Janette Rahamat-Langendoen, Reina Sikkema, Marion Koopmans
EPI_ISL_15704688	Institute for Medical Virology, University Hospital, Goethe University Frankfurt, Germany	Institute of Virology, Charité-Universitätsmedizin Berlin, Humboldt-Universität zu Berlin, Germany	Denisa Bojkova, Julia Schneider, Victor M. Corman, Jindrich Cinatl jr.
EPI_ISL_15704845	Erasmus Medical Center Department of Virology	Erasmus Medical Center Department of Virology	Leonard Schuele, Bas Oude Munnink, Marjan Boter, Babette Weller, Babs Verstrepen, Richard Molenkamp, Janette Rahamat-Langendoen, Reina Sikkema, Marion Koopmans
EPI_ISL_15704861, EPI_ISL_15705166, EPI_ISL_15705315, EPI_ISL_15705498, EPI_ISL_15705640, EPI_ISL_15705771, EPI_ISL_15705945, EPI_ISL_15706105, EPI_ISL_15706238, EPI_ISL_15706380	Institute for Medical Virology, University Hospital, Goethe University Frankfurt, Germany	Institute of Virology, Charité-Universitätsmedizin Berlin, Humboldt-Universität zu Berlin, Germany	Denisa Bojkova, Julia Schneider, Victor M. Corman, Jindrich Cinatl jr.
EPI_ISL_15709387	División de Microbiología, Hospital Nacional de Niños Dr. Carlos Sáenz Herrera	Inciensa, Instituto de Investigación y Enseñanza en Nutrición y Salud	Francisco Duarte, Ana Isela Ruiz-Gonzalez, Hillary Serrano, Diana Cantillo, Claudio Soto-Garita, Gustavo Vega, Estela Cordero, Adriana Godinez, Melany Calderon & Cristian Pérez-Corrales
EPI_ISL_15712853, EPI_ISL_15712860, EPI_ISL_15714234, EPI_ISL_15714244, EPI_ISL_15714245, EPI_ISL_15714286, EPI_ISL_15714287, EPI_ISL_15714288, EPI_ISL_15714299, EPI_ISL_15714539, EPI_ISL_15714920, EPI_ISL_15714989, EPI_ISL_15714990, EPI_ISL_15715289, EPI_ISL_15715292, EPI_ISL_15715305, EPI_ISL_15718514, EPI_ISL_15718920			
see above	Erasmus Medical Center Department of Virology	Erasmus Medical Center Department of Virology	Leonard Schuele, Bas Oude Munnink, Marjan Boter, Babette Weller, Babs Verstrepen, Richard Molenkamp, Janette Rahamat-Langendoen, Reina Sikkema, Marion Koopmans
EPI_ISL_15722680, EPI_ISL_15722681, EPI_ISL_15722682, EPI_ISL_15722683, EPI_ISL_15722684, EPI_ISL_15722685, EPI_ISL_15722686, EPI_ISL_15722687, EPI_ISL_15722688, EPI_ISL_15722689, EPI_ISL_15722690			
see above	Rush University Medical Center	RIPHL at Rush University Medical Center	Stefan Green, Kevin Kunstman, Hannah Barbian, Felix Araujo Perez, Edith Perez, Sofiya Bobrovska, Alyse Kittner, Cecilia Chau, Giancarlo Balangue, Lok Yiu Ashley Wu, Mary Hayden, Joyce Houlihan, Diane Springer, Nicholas Moore
EPI_ISL_15747900	WHO National Influenza Centre Russian Federation	WHO National Influenza Centre Russian Federation	Andrey Komissarov, Artem Fadeev, Nikita Yolshin, Evgeny Venev, Kseniya Komissarova, Daria Danilenko, Dmitry Lioznov
EPI_ISL_15763810, EPI_ISL_15763811, EPI_ISL_15763812, EPI_ISL_15763813, EPI_ISL_15763814, EPI_ISL_15763815, EPI_ISL_15763816, EPI_ISL_15763817, EPI_ISL_15763818, EPI_ISL_15763819, EPI_ISL_15763820, EPI_ISL_15763821, EPI_ISL_15763822, EPI_ISL_15763823, EPI_ISL_15763824, EPI_ISL_15763825, EPI_ISL_15763826, EPI_ISL_15763827, EPI_ISL_15763828, EPI_ISL_15763829, EPI_ISL_15763830, EPI_ISL_15763831, EPI_ISL_15763832			
see above	National Virus Reference Laboratory	National Virus Reference Laboratory	Gabriel Gonzalez, Michael Carr, Brian Keogan, Jose Maria Urtasun Elizari, Jonathan Dean, Daniel Hare, Cillian F De Gascun
EPI_ISL_15802695, EPI_ISL_15802696, EPI_ISL_15802697, EPI_ISL_15802698, EPI_ISL_15802700, EPI_ISL_15802703, EPI_ISL_15802704, EPI_ISL_15802705, EPI_ISL_15802706, EPI_ISL_15802707, EPI_ISL_15802708			
see above	Direccion de Investigacion en Salud Publica, Instituto Nacional de Salud	Direccion de Investigacion en Salud Publica, Instituto Nacional de Salud	Laiton-Donato,K.D., Franco,C.E., Alvarez-Diaz,D.A., Ruiz-Moreno,H.A., Prada,D.A., Martinez,D. and Mercado-Reyes,M.M.
EPI_ISL_15802709	Hanimal Health, Istituto Zooprofilattico Sperimentale del Mezzogiorno	Hanimal Health, Istituto Zooprofilattico Sperimentale del Mezzogiorno	Viscardi,M., Cozzolino,L., De Martinis,C., Cardillo,L. and Fusco,G.
EPI_ISL_15802710, EPI_ISL_15802711, EPI_ISL_15802712, EPI_ISL_15802713, EPI_ISL_15802714, EPI_ISL_15802715, EPI_ISL_15802718, EPI_ISL_15802721	Direccion de Investigacion en Salud Publica, Instituto Nacional de Salud	Direccion de Investigacion en Salud Publica, Instituto Nacional de Salud	Laiton-Donato,K.D., Franco,C.E., Alvarez-Diaz,D.A., Ruiz-Moreno,H.A., Prada,D.A., Martinez,D. and Mercado-Reyes,M.M.
EPI_ISL_15802722, EPI_ISL_15802723, EPI_ISL_15802724, EPI_ISL_15802725, EPI_ISL_15802726, EPI_ISL_15802727, EPI_ISL_15802728, EPI_ISL_15802729, EPI_ISL_15802730, EPI_ISL_15802731, EPI_ISL_15802732, EPI_ISL_15802733, EPI_ISL_15802734, EPI_ISL_15802735, EPI_ISL_15802736, EPI_ISL_15802737, EPI_ISL_15802738, EPI_ISL_15802739, EPI_ISL_15802740, EPI_ISL_15802741,			

EPI_ISL_15802742, EPI_ISL_15802743			
see above	Centre for Biological Threats, Highly Pathogenic Viruses, Robert Koch Institute	Centre for Biological Threats, Highly Pathogenic Viruses, Robert Koch Institute	Brinkmann,A., Kohl,C., Pape,K., Schrick,L., Michel,J., Schaade,L. and Nitsche,A.
EPI_ISL_15819624, EPI_ISL_15819625, EPI_ISL_15819626, EPI_ISL_15819627, EPI_ISL_15819628, EPI_ISL_15819629, EPI_ISL_15819630, EPI_ISL_15819631, EPI_ISL_15819632, EPI_ISL_15819633, EPI_ISL_15819634, EPI_ISL_15819635, EPI_ISL_15819636, EPI_ISL_15819637, EPI_ISL_15819638			
see above	California Department of Public Health	California Department of Public Health	Viral and Rickettsial Disease Laboratory
EPI_ISL_15831211	División Diagnóstico Molecular Hospital México	División Diagnóstico Molecular Hospital México	Juan Carlos Villalobos Ugalde, Vanessa Villalobos Alfaro, Carlos Ramirez Chavarría
EPI_ISL_15831212	División Diagnóstico Molecular Hospital México	División Diagnóstico Molecular Hospital México	Juan Carlos Villalobos Ugalde, Vanessa Villalobos Alfaro, Sofía Villalobos Abarca
EPI_ISL_15855044, EPI_ISL_15855046, EPI_ISL_15855048, EPI_ISL_15855050, EPI_ISL_15855052, EPI_ISL_15855054, EPI_ISL_15855056, EPI_ISL_15855058, EPI_ISL_15855061, EPI_ISL_15855063, EPI_ISL_15855065, EPI_ISL_15855067, EPI_ISL_15855069			
see above	Centre for Biological Threats, Highly Pathogenic Viruses, Robert Koch Institute	Centre for Biological Threats, Highly Pathogenic Viruses, Robert Koch Institute	Brinkmann,A., Kohl,C., Pape,K., Schrick,L., Michel,J., Schaade,L. and Nitsche,A
EPI_ISL_15889123, EPI_ISL_15889124, EPI_ISL_15889125, EPI_ISL_15889127, EPI_ISL_15889129, EPI_ISL_15889130, EPI_ISL_15889131, EPI_ISL_15889132, EPI_ISL_15889134, EPI_ISL_15889135, EPI_ISL_15889136, EPI_ISL_15889138, EPI_ISL_15889147, EPI_ISL_15889148, EPI_ISL_15889149, EPI_ISL_15889151, EPI_ISL_15889152, EPI_ISL_15889155, EPI_ISL_15889156, EPI_ISL_15889158, EPI_ISL_15889159, EPI_ISL_15889160, EPI_ISL_15889161, EPI_ISL_15889163, EPI_ISL_15889164, EPI_ISL_15889167, EPI_ISL_15889169			
see above	Public Health and Environmental Laboratories, New Jersey Department of Health	Public Health and Environmental Laboratories, New Jersey Department of Health	Palmateer,N.C.
EPI_ISL_15889180	Centers for Disease Control & Prevention (CDC), Division of High Consequence Pathogens and Pathology (DHCPP-PRB)	Centers for Disease Control & Prevention (CDC), Division of High Consequence Pathogens and Pathology (DHCPP-PRB)	Gigante,C., Ingram,A., Zhao,H., Batra,D., Hetrick,E., Howard,D., Kovar,L., Seabolt,M., Morrison,S., Desch,M., Knipe,K., Weigand,M., Sheth,M., Burgin,A., Burroughs,M., Lee,J., Wilkins,K., McCollum,A., Hutson,C., Davidson,W., Rao,A., Geeter,E. and Li,Y.
EPI_ISL_15889181, EPI_ISL_15889182	Centers for Disease Control & Prevention (CDC), Division of High Consequence Pathogens and Pathology (DHCPP-PRB)	Centers for Disease Control & Prevention (CDC), Division of High Consequence Pathogens and Pathology (DHCPP-PRB)	Gigante,C., Ventura,J., Zhao,H., Batra,D., Hetrick,E., Howard,D., Kovar,L., Seabolt,M., Morrison,S., Desch,M., Knipe,K., Weigand,M., Sheth,M., Burgin,A., Burroughs,M., Lee,J., Wilkins,K., McCollum,A., Hutson,C., Davidson,W., Rao,A., Nash,J. and Li,Y.
EPI_ISL_15889183	Centers for Disease Control & Prevention (CDC), Division of High Consequence Pathogens and Pathology (DHCPP-PRB)	Centers for Disease Control & Prevention (CDC), Division of High Consequence Pathogens and Pathology (DHCPP-PRB)	Gigante,C., Moreno-Gorrin,C., Zhao,H., Batra,D., Hetrick,E., Howard,D., Kovar,L., Seabolt,M., Morrison,S., Desch,M., Knipe,K., Weigand,M., Sheth,M., Burgin,A., Burroughs,M., Lee,J., Wilkins,K., McCollum,A., Hutson,C., Davidson,W., Rao,A., Miller,H. and Li,Y.
EPI_ISL_15889184, EPI_ISL_15889185	Centers for Disease Control & Prevention (CDC), Division of High Consequence Pathogens and Pathology (DHCPP-PRB)	Centers for Disease Control & Prevention (CDC), Division of High Consequence Pathogens and Pathology (DHCPP-PRB)	Gigante,C., Lee,P., Zhao,H., Batra,D., Hetrick,E., Howard,D., Kovar,L., Seabolt,M., Morrison,S., Desch,M., Knipe,K., Weigand,M., Sheth,M., Burgin,A., Burroughs,M., Lee,J., Wilkins,K., McCollum,A., Hutson,C., Davidson,W., Rao,A., Stanek,D. and Li,Y.
EPI_ISL_15889186	Centers for Disease Control & Prevention (CDC), Division of High Consequence Pathogens and Pathology (DHCPP-PRB)	Centers for Disease Control & Prevention (CDC), Division of High Consequence Pathogens and Pathology (DHCPP-PRB)	Gigante,C., Salinas,A., Zhao,H., Batra,D., Hetrick,E., Howard,D., Kovar,L., Seabolt,M., Morrison,S., Desch,M., Knipe,K., Weigand,M., Sheth,M., Burgin,A., Burroughs,M., Lee,J., Wilkins,K., McCollum,A., Hutson,C., Davidson,W., Rao,A., Haydel,D. and Li,Y.
EPI_ISL_15889187, EPI_ISL_15889188	Centers for Disease Control & Prevention (CDC), Division of High Consequence Pathogens and Pathology (DHCPP-PRB)	Centers for Disease Control & Prevention (CDC), Division of High Consequence Pathogens and Pathology (DHCPP-PRB)	Gigante,C., Myers,R., Zhao,H., Batra,D., Hetrick,E., Howard,D., Kovar,L., Seabolt,M., Morrison,S., Desch,M., Knipe,K., Weigand,M., Sheth,M., Burgin,A., Burroughs,M., Lee,J., Wilkins,K., McCollum,A., Hutson,C., Davidson,W., Rao,A., Blythe,D. and Li,Y.
EPI_ISL_15889189	Centers for Disease Control & Prevention (CDC), Division of High Consequence Pathogens and Pathology (DHCPP-PRB)	Centers for Disease Control & Prevention (CDC), Division of High Consequence Pathogens and Pathology (DHCPP-PRB)	Gigante,C., Cleavinger,K., Zhao,H., Batra,D., Hetrick,E., Howard,D., Kovar,L., Seabolt,M., Morrison,S., Desch,M., Knipe,K., Weigand,M., Sheth,M., Burgin,A., Burroughs,M., Lee,J., Wilkins,K., McCollum,A., Hutson,C., Davidson,W., Rao,A., Sinn,M. and Li,Y.
EPI_ISL_15889190, EPI_ISL_15889191, EPI_ISL_15889192	Centers for Disease Control & Prevention (CDC), Division of High Consequence Pathogens and Pathology (DHCPP-PRB)	Centers for Disease Control & Prevention (CDC), Division of High Consequence Pathogens and Pathology (DHCPP-PRB)	Gigante,C., Kubin,G., Zhao,H., Batra,D., Hetrick,E., Howard,D., Kovar,L., Seabolt,M., Morrison,S., Desch,M., Knipe,K., Weigand,M., Sheth,M., Burgin,A., Burroughs,M., Lee,J., Wilkins,K., McCollum,A., Hutson,C., Davidson,W., Rao,A., White,S. and Li,Y.
EPI_ISL_15889193, EPI_ISL_15889194	Centers for Disease Control & Prevention (CDC), Division of High Consequence Pathogens and Pathology (DHCPP-PRB)	Centers for Disease Control & Prevention (CDC), Division of High Consequence Pathogens and Pathology (DHCPP-PRB)	Gigante,C., Griffin-Thomas,L., Zhao,H., Batra,D., Hetrick,E., Howard,D., Kovar,L., Seabolt,M., Morrison,S., Desch,M., Knipe,K., Weigand,M., Sheth,M., Burgin,A., Burroughs,M., Lee,J., Wilkins,K., McCollum,A., Hutson,C., Davidson,W., Rao,A., Crain,J. and Li,Y.
EPI_ISL_15889195	Centers for Disease Control & Prevention (CDC), Division of High Consequence Pathogens and Pathology (DHCPP-PRB)	Centers for Disease Control & Prevention (CDC), Division of High Consequence Pathogens and Pathology (DHCPP-PRB)	Gigante,C., Achillies,C., Zhao,H., Batra,D., Hetrick,E., Howard,D., Kovar,L., Seabolt,M., Morrison,S., Desch,M., Knipe,K., Weigand,M., Sheth,M., Burgin,A., Burroughs,M., Lee,J., Wilkins,K., McCollum,A., Hutson,C., Davidson,W., Rao,A., Blevins,L. and Li,Y.
EPI_ISL_15896301, EPI_ISL_15896302, EPI_ISL_15896303, EPI_ISL_15896305	Los Angeles County Public Health Laboratories	Los Angeles County Public Health Laboratories	P. Hemarajata et al.
EPI_ISL_15896306, EPI_ISL_15896307, EPI_ISL_15896308, EPI_ISL_15896309, EPI_ISL_15896310, EPI_ISL_15896311, EPI_ISL_15896312, EPI_ISL_15896313, EPI_ISL_15896314, EPI_ISL_15896315, EPI_ISL_15896316, EPI_ISL_15896317, EPI_ISL_15896318, EPI_ISL_15896319, EPI_ISL_15896320, EPI_ISL_15896321, EPI_ISL_15896322, EPI_ISL_15896323, EPI_ISL_15896324, EPI_ISL_15896325, EPI_ISL_15896326, EPI_ISL_15896327			
see above	Kaiser Permanente Chino Hills Regional Reference Laboratories	Los Angeles County Public Health Laboratories	P. Hemarajata et al.
EPI_ISL_15896329, EPI_ISL_15896330, EPI_ISL_15896331, EPI_ISL_15896332, EPI_ISL_15896334, EPI_ISL_15896335, EPI_ISL_15896336, EPI_ISL_15896337, EPI_ISL_15896338, EPI_ISL_15896339, EPI_ISL_15896340, EPI_ISL_15896341, EPI_ISL_15896342, EPI_ISL_15896343, EPI_ISL_15896344, EPI_ISL_15896345, EPI_ISL_15896346, EPI_ISL_15896347, EPI_ISL_15896348, EPI_ISL_15896349, EPI_ISL_15896350, EPI_ISL_15896351, EPI_ISL_15896352, EPI_ISL_15896353			
see above	Los Angeles County Public Health Laboratories	Los Angeles County Public Health Laboratories	P. Hemarajata et al.
EPI_ISL_15896817, EPI_ISL_15896818, EPI_ISL_15897122, EPI_ISL_15897123, EPI_ISL_15897124, EPI_ISL_15897493, EPI_ISL_15897494, EPI_ISL_15897569, EPI_ISL_15897570, EPI_ISL_15897573, EPI_ISL_15897587, EPI_ISL_15897588, EPI_ISL_15897589, EPI_ISL_15897590, EPI_ISL_15897591			
see above	Landspitali Department of Clinical Microbiology	Landspitali Department of Clinical Microbiology	Freyja Valsdottir, Zarko Urosevic, Brynja Armannsdottir, Karl Gustaf Kristinsson
EPI_ISL_15912046, EPI_ISL_15912047, EPI_ISL_15912048, EPI_ISL_15912049, EPI_ISL_15912050, EPI_ISL_15912051, EPI_ISL_15912052, EPI_ISL_15912053, EPI_ISL_15912054, EPI_ISL_15912055, EPI_ISL_15912056, EPI_ISL_15912057, EPI_ISL_15912058, EPI_ISL_15912059, EPI_ISL_15912060, EPI_ISL_15912061, EPI_ISL_15912062, EPI_ISL_15912063, EPI_ISL_15912064, EPI_ISL_15912066, EPI_ISL_15912067, EPI_ISL_15912068, EPI_ISL_15912069, EPI_ISL_15912070, EPI_ISL_15912071, EPI_ISL_15912072, EPI_ISL_15912073, EPI_ISL_15912074, EPI_ISL_15912075, EPI_ISL_15912076, EPI_ISL_15912077, EPI_ISL_15912078, EPI_ISL_15912079, EPI_ISL_15912080, EPI_ISL_15912081, EPI_ISL_15912082, EPI_ISL_15912083, EPI_ISL_15912084, EPI_ISL_15912085, EPI_ISL_15912086, EPI_ISL_15912087, EPI_ISL_15912088, EPI_ISL_15912089, EPI_ISL_15912090, EPI_ISL_15912091, EPI_ISL_15912092, EPI_ISL_15912093, EPI_ISL_15912094, EPI_ISL_15912095, EPI_ISL_15912096, EPI_ISL_15912097, EPI_ISL_15912098, EPI_ISL_15912099, EPI_ISL_15912100, EPI_ISL_15912101, EPI_ISL_15912102, EPI_ISL_15912103, EPI_ISL_15912104, EPI_ISL_15912105, EPI_ISL_15912106, EPI_ISL_15912107, EPI_ISL_15912108, EPI_ISL_15912109, EPI_ISL_15912110, EPI_ISL_15912111, EPI_ISL_15912112, EPI_ISL_15912113, EPI_ISL_15912114, EPI_ISL_15912115, EPI_ISL_15912116, EPI_ISL_15912117, EPI_ISL_15912118, EPI_ISL_15912119, EPI_ISL_15912120, EPI_ISL_15912121, EPI_ISL_15912123, EPI_ISL_15912124, EPI_ISL_15912125, EPI_ISL_15912126, EPI_ISL_15912127, EPI_ISL_15912129, EPI_ISL_15912130, EPI_ISL_15912131, EPI_ISL_15912132, EPI_ISL_15912133, EPI_ISL_15912134, EPI_ISL_15912135, EPI_ISL_15912137, EPI_ISL_15912138, EPI_ISL_15912140, EPI_ISL_15912142, EPI_ISL_15912143, EPI_ISL_15912145, EPI_ISL_15912146, EPI_ISL_15912147, EPI_ISL_15912148, EPI_ISL_15912150, EPI_ISL_15912151, EPI_ISL_15912153, EPI_ISL_15912154, EPI_ISL_15912156, EPI_ISL_15912157, EPI_ISL_15912159, EPI_ISL_15912160, EPI_ISL_15912164, EPI_ISL_15912165, EPI_ISL_15912166, EPI_ISL_15912167, EPI_ISL_15912168, EPI_ISL_15912169, EPI_ISL_15912170, EPI_ISL_15912171, EPI_ISL_15912174, EPI_ISL_15912175, EPI_ISL_15912176, EPI_ISL_15912177, EPI_ISL_15912181, EPI_ISL_15912182, EPI_ISL_15912183, EPI_ISL_15912184, EPI_ISL_15912185, EPI_ISL_15912187, EPI_ISL_15912188, EPI_ISL_15912189, EPI_ISL_15912190, EPI_ISL_15912191, EPI_ISL_15912196, EPI_ISL_15912199, EPI_ISL_15912200, EPI_ISL_15912203, EPI_ISL_15912205, EPI_ISL_15912208, EPI_ISL_15912209, EPI_ISL_15912211			
see above	DPH, Massachusetts State Public Health Laboratory	DPH, Massachusetts State Public Health Laboratory	Doucette,M., Gagne,L. and Smole,S.C.
EPI_ISL_15912317, EPI_ISL_15912318, EPI_ISL_15912319, EPI_ISL_15912320, EPI_ISL_15912321, EPI_ISL_15912322, EPI_ISL_15912323, EPI_ISL_15912324, EPI_ISL_15912325, EPI_ISL_15912326, EPI_ISL_15912327, EPI_ISL_15912328, EPI_ISL_15912329, EPI_ISL_15912330, EPI_ISL_15912331, EPI_ISL_15912332, EPI_ISL_15912333, EPI_ISL_15912334, EPI_ISL_15912335, EPI_ISL_15912336, EPI_ISL_15912337, EPI_ISL_15912338, EPI_ISL_15912339, EPI_ISL_15912340, EPI_ISL_15912341, EPI_ISL_15912342, EPI_ISL_15912343, EPI_ISL_15912344			
see above	Indian Council of Medical Research-National Institute of Virology, Microbial Containment Complex	Indian Council of Medical Research-National Institute of Virology, Microbial Containment Complex	Pragya D. Yadav
EPI_ISL_15912916	The Microbiology laboratory, AUSL Romagna, Piesestina, Cesena, FC	Dipartimento di Medicina Specialistica Diagnostica e Sperimentale, University of Bologna Italy	Alessandra Scagliarini, Vittorio Sambri, MAria Elena Turba, Fabio Gentilini
EPI_ISL_15942296	Sexually Transmitted Diseases (STDs) outpatient service of Dermatology Unit, Fondazione IRCCS Ca' Granda Ospedale Maggiore Policlinico of Milan	Bioinformatic lab, Scientific Institute IRCCS E. Medea	Diego Forni, Rachele Cagliani, Manuela Sironi, Chiara Moltrasio, Luigia Venegoni, Eleonora Quattri, Angelo Marzano
EPI_ISL_15942637, EPI_ISL_15942638, EPI_ISL_15942885, EPI_ISL_15942886	Sexually Transmitted Diseases (STDs) outpatient service of Dermatology Unit, Fondazione IRCCS Ca' Granda Ospedale Maggiore Policlinico of Milan	Bioinformatic Lab, Scientific Institute IRCCS E. Medea	Diego Forni, Rachele Cagliani, Manuela Sironi, Chiara Moltrasio, Luigia Venegoni, Eleonora Quattri, Angelo Marzano
EPI_ISL_15950021, EPI_ISL_15950022, EPI_ISL_15950023, EPI_ISL_15950024, EPI_ISL_15950025, EPI_ISL_15950026, EPI_ISL_15950027, EPI_ISL_15950029, EPI_ISL_15950030, EPI_ISL_15950031, EPI_ISL_15950032			
see above	Rhode Island State Health Laboratory	Rhode Island State Health Laboratory	Kristin Carpenter-Azevedo, Sean Sierra-Patev, Richard C. Huard
EPI_ISL_15955332, EPI_ISL_15955333, EPI_ISL_15955334, EPI_ISL_15955335, EPI_ISL_15955336, EPI_ISL_15955337	Kaiser Permanente Chino Hills Regional Reference Laboratories	Los Angeles County Public Health Laboratories	P. Hemarajata et al.
EPI_ISL_15955338, EPI_ISL_15955339	Los Angeles County Public Health Laboratories	Los Angeles County Public Health Laboratories	P. Hemarajata et al.
EPI_ISL_15955340	Laboratory Corporation of America	Los Angeles County Public Health Laboratories	P. Hemarajata et al.
EPI_ISL_15955341, EPI_ISL_15955342, EPI_ISL_15955343, EPI_ISL_15955344, EPI_ISL_15955345, EPI_ISL_15955346, EPI_ISL_15955347, EPI_ISL_15955348, EPI_ISL_15955349, EPI_ISL_15955350, EPI_ISL_15955351, EPI_ISL_15955352, EPI_ISL_15955353, EPI_ISL_15955354, EPI_ISL_15955355, EPI_ISL_15955356, EPI_ISL_15955357			
see above	Kaiser Permanente Chino Hills Regional Reference Laboratories	Los Angeles County Public Health Laboratories	P. Hemarajata et al.
EPI_ISL_15965179	Institute of Microbiology, Universidad San Francisco de Quito	Institute of Microbiology, Universidad San Francisco de Quito	Belén Prado-Vivar, Mateo Carvajal, Sully Márquez, Erika B. Muñoz, Rommel Guevara, Maritza Paez, Estefanía Rivadeneira, Evelyn Sánchez Espinoza, Josefina Coloma, Verónica Barragán, Patricio Rojas-Silva, Gabriel Trueba, Michelle Grunauer, Paúl Cárdenas
EPI_ISL_15969892	Oxford University Clinical Research Unit	Oxford University Clinical Research Unit	Nguyen Thanh Dung, Le Manh Hung, Huynh Thi Thuy Hoa, Tang Chi Thuong, Le Hong Nga, Nguyen Huu Hung, Nghiem My Ngoc, Nguyen Thi Thu Hong, Vo Truong Quy, Vu Thi Kim Thoa, Nguyen Thi Thanh, Phan Vinh Tho, Nguyen Le Nhu Tung, Le Mau Toan, Vo Minh Quang, Dinh Nguyen Huy Man, Nguyễn Tấn Phát, Trần Thị Lan Phương, Trần Thị Thanh Tâm, Phạm Thị Ngọc Thoa, Nguyen Hong Tam, Truong Thi Thanh Lan, Nguyen Thi Han Ny, Tran Tan Thanh, Le Thuy Thuy Khanh, Lam Minh Yen, Guy Thwaites, Louise Thwaites, Nguyen Van Vinh Chau, Nguyen To Anh, Le Van Tan
EPI_ISL_15972402, EPI_ISL_15972403, EPI_ISL_15972404, EPI_ISL_15972406, EPI_ISL_15972407, EPI_ISL_15972408,	Laboratorio Central, Ministerio de Salud Córdoba	Laboratorio Central, Ministerio de Salud Córdoba	Castro, G.; Sicilia, P.; Poklepovich, T.; Campos, J.; Barbas, G.

EPI_ISL_15972409			
EPI_ISL_15992095	LESP State of Mexico	Instituto de Diagnostico y Referencia Epidemiologicos (INDRE)	Abril Rodríguez-Maldonado; Claudia Wong-Arámbula; Felipe Arguijo-Perez; Helios Cárdenas-Hernández; Carmen Castro-Méndez; Lidia García-Torres; Ruth Madera-Sandoval; América Mandujano-Martínez; Nancy Martínez-Velázquez; Mireya Mederos-Michel; Angélica Pedraza-Meléndez; Joaquín Quiroz-Mercado; Daniel Regalado-Santiago; Silvia Rivero-Arredondo; Erika Sierra-Atanacio; Fernando González-Domínguez; Lucia Hernández-Rivas, Irma López-Martínez; Ernesto Ramírez-González; Maribel González-Villa
EPI_ISL_15992096	LESP Jalisco	Instituto de Diagnostico y Referencia Epidemiologicos (INDRE)	Abril Rodríguez-Maldonado; Claudia Wong-Arámbula; Felipe Arguijo-Perez; Helios Cárdenas-Hernández; Carmen Castro-Méndez; Lidia García-Torres; Ruth Madera-Sandoval; América Mandujano-Martínez; Nancy Martínez-Velázquez; Mireya Mederos-Michel; Angélica Pedraza-Meléndez; Joaquín Quiroz-Mercado; Daniel Regalado-Santiago; Silvia Rivero-Arredondo; Erika Sierra-Atanacio; Fernando González-Domínguez; Lucia Hernández-Rivas, Irma López-Martínez; Ernesto Ramírez-González; Maribel González-Villa
EPI_ISL_15992097	LESP Morelos	Instituto de Diagnostico y Referencia Epidemiologicos (INDRE)	Abril Rodríguez-Maldonado; Claudia Wong-Arámbula; Felipe Arguijo-Perez; Helios Cárdenas-Hernández; Carmen Castro-Méndez; Lidia García-Torres; Ruth Madera-Sandoval; América Mandujano-Martínez; Nancy Martínez-Velázquez; Mireya Mederos-Michel; Angélica Pedraza-Meléndez; Joaquín Quiroz-Mercado; Daniel Regalado-Santiago; Silvia Rivero-Arredondo; Erika Sierra-Atanacio; Fernando González-Domínguez; Lucia Hernández-Rivas, Irma López-Martínez; Ernesto Ramírez-González; Maribel González-Villa
EPI_ISL_15992098	LESP Nuevo Leon	Instituto de Diagnostico y Referencia Epidemiologicos (INDRE)	Abril Rodríguez-Maldonado; Claudia Wong-Arámbula; Felipe Arguijo-Perez; Helios Cárdenas-Hernández; Carmen Castro-Méndez; Lidia García-Torres; Ruth Madera-Sandoval; América Mandujano-Martínez; Nancy Martínez-Velázquez; Mireya Mederos-Michel; Angélica Pedraza-Meléndez; Joaquín Quiroz-Mercado; Daniel Regalado-Santiago; Silvia Rivero-Arredondo; Erika Sierra-Atanacio; Fernando González-Domínguez; Lucia Hernández-Rivas, Irma López-Martínez; Ernesto Ramírez-González; Maribel González-Villa
EPI_ISL_15992099	LESP Hidalgo	Instituto de Diagnostico y Referencia Epidemiologicos (INDRE)	Abril Rodríguez-Maldonado; Claudia Wong-Arámbula; Felipe Arguijo-Perez; Helios Cárdenas-Hernández; Carmen Castro-Méndez; Lidia García-Torres; Ruth Madera-Sandoval; América Mandujano-Martínez; Nancy Martínez-Velázquez; Mireya Mederos-Michel; Angélica Pedraza-Meléndez; Joaquín Quiroz-Mercado; Daniel Regalado-Santiago; Silvia Rivero-Arredondo; Erika Sierra-Atanacio; Fernando González-Domínguez; Lucia Hernández-Rivas, Irma López-Martínez; Ernesto Ramírez-González; Maribel González-Villa
EPI_ISL_15992100	LESP Campeche	Instituto de Diagnostico y Referencia Epidemiologicos (INDRE)	Abril Rodríguez-Maldonado; Claudia Wong-Arámbula; Felipe Arguijo-Perez; Helios Cárdenas-Hernández; Carmen Castro-Méndez; Lidia García-Torres; Ruth Madera-Sandoval; América Mandujano-Martínez; Nancy Martínez-Velázquez; Mireya Mederos-Michel; Angélica Pedraza-Meléndez; Joaquín Quiroz-Mercado; Daniel Regalado-Santiago; Silvia Rivero-Arredondo; Erika Sierra-Atanacio; Fernando González-Domínguez; Lucia Hernández-Rivas, Irma López-Martínez; Ernesto Ramírez-González; Maribel González-Villa
EPI_ISL_15992101	LESP Tlaxcala	Instituto de Diagnostico y Referencia Epidemiologicos (INDRE)	Abril Rodríguez-Maldonado; Claudia Wong-Arámbula; Felipe Arguijo-Perez; Helios Cárdenas-Hernández; Carmen Castro-Méndez; Lidia García-Torres; Ruth Madera-Sandoval; América Mandujano-Martínez; Nancy Martínez-Velázquez; Mireya Mederos-Michel; Angélica Pedraza-Meléndez; Joaquín Quiroz-Mercado; Daniel Regalado-Santiago; Silvia Rivero-Arredondo; Erika Sierra-Atanacio; Fernando González-Domínguez; Lucia Hernández-Rivas, Irma López-Martínez; Ernesto Ramírez-González; Maribel González-Villa
EPI_ISL_15992102	LESP Aguascalientes	Instituto de Diagnostico y Referencia Epidemiologicos (INDRE)	Abril Rodríguez-Maldonado; Claudia Wong-Arámbula; Felipe Arguijo-Perez; Helios Cárdenas-Hernández; Carmen Castro-Méndez; Lidia García-Torres; Ruth Madera-Sandoval; América Mandujano-Martínez; Nancy Martínez-Velázquez; Mireya Mederos-Michel; Angélica Pedraza-Meléndez; Joaquín Quiroz-Mercado; Daniel Regalado-Santiago; Silvia Rivero-Arredondo; Erika Sierra-Atanacio; Fernando González-Domínguez; Lucia Hernández-Rivas, Irma López-Martínez; Ernesto Ramírez-González; Maribel González-Villa
EPI_ISL_15992103, EPI_ISL_15992104	LESP San Luis Potosi	Instituto de Diagnostico y Referencia Epidemiologicos (INDRE)	Abril Rodríguez-Maldonado; Claudia Wong-Arámbula; Felipe Arguijo-Perez; Helios Cárdenas-Hernández; Carmen Castro-Méndez; Lidia García-Torres; Ruth Madera-Sandoval; América Mandujano-Martínez; Nancy Martínez-Velázquez; Mireya Mederos-Michel; Angélica Pedraza-Meléndez; Joaquín Quiroz-Mercado; Daniel Regalado-Santiago; Silvia Rivero-Arredondo; Erika Sierra-Atanacio; Fernando González-Domínguez; Lucia Hernández-Rivas, Irma López-Martínez; Ernesto Ramírez-González; Maribel González-Villa
EPI_ISL_16006562, EPI_ISL_16006563	Sequencing/Bioinformatics, Delaware Public Health Lab	Sequencing/Bioinformatics, Delaware Public Health Lab	Bajwa,M.I. and Miller,H.
EPI_ISL_16012468, EPI_ISL_16012469, EPI_ISL_16012470, EPI_ISL_16012471, EPI_ISL_16012472, EPI_ISL_16012473, EPI_ISL_16012474, EPI_ISL_16012475, EPI_ISL_16012476, EPI_ISL_16012477, EPI_ISL_16012478, EPI_ISL_16012479, EPI_ISL_16012480, EPI_ISL_16012481, EPI_ISL_16012482, EPI_ISL_16012483, EPI_ISL_16012484, EPI_ISL_16012485, EPI_ISL_16012486, EPI_ISL_16012487, EPI_ISL_16012488, EPI_ISL_16012489, EPI_ISL_16012490, EPI_ISL_16012491, EPI_ISL_16012492, EPI_ISL_16012493, EPI_ISL_16012494, EPI_ISL_16012495, EPI_ISL_16012496, EPI_ISL_16012497, EPI_ISL_16012498, EPI_ISL_16012499, EPI_ISL_16012500, EPI_ISL_16012501, EPI_ISL_16012502, EPI_ISL_16012503, EPI_ISL_16012504, EPI_ISL_16012505, EPI_ISL_16012506, EPI_ISL_16012507, EPI_ISL_16012508, EPI_ISL_16012509, EPI_ISL_16012510, EPI_ISL_16012511, EPI_ISL_16012512, EPI_ISL_16012513, EPI_ISL_16012514, EPI_ISL_16012515, EPI_ISL_16012516, EPI_ISL_16012517, EPI_ISL_16012518, EPI_ISL_16012519, EPI_ISL_16012520, EPI_ISL_16012521, EPI_ISL_16012522, EPI_ISL_16012523, EPI_ISL_16012524, EPI_ISL_16012525, EPI_ISL_16012526, EPI_ISL_16012527, EPI_ISL_16012528, EPI_ISL_16012529, EPI_ISL_16012530, EPI_ISL_16012531, EPI_ISL_16012532, EPI_ISL_16012533, EPI_ISL_16012534, EPI_ISL_16012535, EPI_ISL_16012536			
see above	National Virus Reference Laboratory	National Virus Reference Laboratory	Gabriel Gonzalez, Michael Carr, Brian Keogan, Jose Maria Urtasun Elizari, Jonathan Dean, Daniel Hare, Cillian F De Gascun
EPI_ISL_16031970, EPI_ISL_16031971, EPI_ISL_16031974, EPI_ISL_16031976, EPI_ISL_16031979	Laboratorio de Investigacion Molecular (UNIMOL), Universidad de Cartagena	Laboratorio de Investigacion Molecular (UNIMOL), Universidad de Cartagena	Loyola,S., Fernandez-Ruiz,M., Torres-Pacheco,J., Franco-Munoz,C., Laiton-Donato,K., Ruiz-Moreno,H., Mercado-Reyes,M. and Gomez-Camargo,D.
EPI_ISL_16074741	Virology Lab, Institute of Tropical Medicine of Sao Paulo, School of Medicine, Universidade de Sao Paulo	Virology Lab	Antonio Charlys da Costa, Maria Cassia Mendes-Correa
EPI_ISL_16074742	Virology Lab, Institute of Tropical Medicine of Sao Paulo, School of Medicine, Universidade de Sao Paulo	Virology Lab, Institute of Tropical Medicine of Sao Paulo, School of Medicine, Universidade de Sao Paulo	Antonio Charlys da Costa, Maria Cassia Mendes-Correa
EPI_ISL_16080585, EPI_ISL_16080586, EPI_ISL_16080587, EPI_ISL_16080588, EPI_ISL_16080589, EPI_ISL_16080590	Institute of Virology, Faculty of Medicine and University Hospital Cologne, University of Cologne, Cologne, Germany	Institute of Virology, Faculty of Medicine and University Hospital Cologne, University of Cologne, Cologne, Germany	Eva Heger, Michael Böhm, Ulrike Wieland, Alexander Kreuter
EPI_ISL_16104842, EPI_ISL_16104843, EPI_ISL_16104844, EPI_ISL_16104845, EPI_ISL_16104846, EPI_ISL_16104847, EPI_ISL_16104848, EPI_ISL_16104849, EPI_ISL_16104850, EPI_ISL_16104851, EPI_ISL_16104852, EPI_ISL_16104853, EPI_ISL_16104854, EPI_ISL_16104855, EPI_ISL_16104856, EPI_ISL_16104857, EPI_ISL_16104858, EPI_ISL_16104859, EPI_ISL_16104860, EPI_ISL_16104861, EPI_ISL_16104862, EPI_ISL_16104863, EPI_ISL_16104864, EPI_ISL_16104865, EPI_ISL_16104866, EPI_ISL_16104867, EPI_ISL_16104868, EPI_ISL_16104869, EPI_ISL_16104870, EPI_ISL_16104871, EPI_ISL_16104872, EPI_ISL_16104873, EPI_ISL_16104874, EPI_ISL_16104875, EPI_ISL_16104876, EPI_ISL_16104877, EPI_ISL_16104878, EPI_ISL_16104879, EPI_ISL_16104880, EPI_ISL_16104881, EPI_ISL_16104882, EPI_ISL_16104883, EPI_ISL_16104884, EPI_ISL_16104885, EPI_ISL_16104886, EPI_ISL_16104887, EPI_ISL_16104888, EPI_ISL_16104889, EPI_ISL_16104890, EPI_ISL_16104891, EPI_ISL_16104892, EPI_ISL_16104893, EPI_ISL_16104894, EPI_ISL_16104895, EPI_ISL_16104896, EPI_ISL_16104897, EPI_ISL_16104898, EPI_ISL_16104899, EPI_ISL_16104900, EPI_ISL_16104901, EPI_ISL_16104902, EPI_ISL_16104903, EPI_ISL_16104904, EPI_ISL_16104905, EPI_ISL_16104906, EPI_ISL_16104907, EPI_ISL_16104908, EPI_ISL_16104909, EPI_ISL_16104910, EPI_ISL_16104911, EPI_ISL_16104912, EPI_ISL_16104913, EPI_ISL_16104914, EPI_ISL_16104915, EPI_ISL_16104916, EPI_ISL_16104917, EPI_ISL_16104918, EPI_ISL_16104919, EPI_ISL_16104920, EPI_ISL_16104921, EPI_ISL_16104922, EPI_ISL_16116727, EPI_ISL_16116728, EPI_ISL_16116729, EPI_ISL_16116730, EPI_ISL_16116733, EPI_ISL_16116734, EPI_ISL_16116735, EPI_ISL_16116736, EPI_ISL_16116737, EPI_ISL_16116738, EPI_ISL_16116739, EPI_ISL_16116740, EPI_ISL_16116741, EPI_ISL_16116742, EPI_ISL_16116743, EPI_ISL_16116744, EPI_ISL_16116745, EPI_ISL_16116746, EPI_ISL_16116747, EPI_ISL_16116748, EPI_ISL_16116749, EPI_ISL_16116750, EPI_ISL_16116751, EPI_ISL_16116752, EPI_ISL_16116754, EPI_ISL_16116755, EPI_ISL_16116756, EPI_ISL_16116757, EPI_ISL_16116758, EPI_ISL_16116759, EPI_ISL_16116760, EPI_ISL_16116761, EPI_ISL_16116762, EPI_ISL_16116763, EPI_ISL_16116764, EPI_ISL_16116765			
see above	Laboratorio de Referencia Nacional de Viruas Inmunoprevenibles. Centro Nacional de Salud Publica. Instiuto Nacional de Salud	Laboratorio de Referencia Nacional de Viruas Inmunoprevenibles. Centro Nacional de Salud Publica. Instiuto Nacional de Salud	Carlos Patricio Padilla Rojas, Carmen Verónica Hurtado Vela, Juana Iris Silva Molina, Luis Bárcena Flores, Víctor Jiménez Vásquez, Alicia Elizabeth Núñez Llanos, Wendy Lizarraga Olivares, Luren Nieves Sevilla Catañeda, Kelly Vanessa Izarra Rojas, Karla Vasquez Cajachahua, Steve Vladimir Acedo Lazo, Omar Alberto Cáceres Rey, Henri Bailón Calderón, Priscila Nayu Lope Pari, Nancy Rojas Serrano, Gloria Arotinco Garayar. Equipo de vigilancia genomica del Instituto Nacional de Salud.
EPI_ISL_16138916, EPI_ISL_16138917, EPI_ISL_16138918, EPI_ISL_16138919, EPI_ISL_16138920, EPI_ISL_16138921, EPI_ISL_16138922, EPI_ISL_16138923, EPI_ISL_16138924, EPI_ISL_16138925, EPI_ISL_16138926, EPI_ISL_16138927, EPI_ISL_16138928, EPI_ISL_16138929, EPI_ISL_16138930, EPI_ISL_16138933, EPI_ISL_16138937, EPI_ISL_16138939, EPI_ISL_16138940, EPI_ISL_16138941, EPI_ISL_16138944			
see above	California Department of Public Health	California Department of Public Health	Viral and Rickettsial Disease Laboratory
EPI_ISL_16183626, EPI_ISL_16183627	Centers for Disease Control & Prevention (CDC), Division of High Consequence Pathogens and Pathology (DHCPP-PRB)	Centers for Disease Control & Prevention (CDC), Division of High Consequence Pathogens and Pathology (DHCPP-PRB)	Gigante,C., Pavlick,J., Zhao,H., Batra,D., Hetrick,E., Howard,D., Kovar,L., Seabolt,M., Morrison,S., Desch,M., Knipe,K., Weigand,M., Sheth,M., Burgin,A., Burroughs,M., Lee,J., Wilkins,K., McCollum,A., Hutson,C., Davidson,W., Rao,A., Parrott,T. and Li,Y.
EPI_ISL_16183628, EPI_ISL_16183629, EPI_ISL_16183630	Centers for Disease Control & Prevention (CDC), Division of High Consequence Pathogens and Pathology (DHCPP-PRB)	Centers for Disease Control & Prevention (CDC), Division of High Consequence Pathogens and Pathology (DHCPP-PRB)	Gigante,C., Hottel,W., Zhao,H., Batra,D., Hetrick,E., Howard,D., Kovar,L., Seabolt,M., Morrison,S., Desch,M., Knipe,K., Weigand,M., Sheth,M., Burgin,A., Burroughs,M., Lee,J., Wilkins,K., McCollum,A., Hutson,C., Davidson,W., Rao,A., Nelson,M. and Li,Y.
EPI_ISL_16183631, EPI_ISL_16183632	Centers for Disease Control & Prevention (CDC), Division of High Consequence Pathogens and Pathology (DHCPP-PRB)	Centers for Disease Control & Prevention (CDC), Division of High Consequence Pathogens and Pathology (DHCPP-PRB)	Gigante,C., Ghinai,I., Zhao,H., Batra,D., Hetrick,E., Howard,D., Kovar,L., Seabolt,M., Morrison,S., Desch,M., Knipe,K., Weigand,M., Sheth,M., Burgin,A., Burroughs,M., Lee,J., Wilkins,K., McCollum,A., Hutson,C., Davidson,W., Rao,A., Kerins,J. and Li,Y.
EPI_ISL_16183633, EPI_ISL_16183634, EPI_ISL_16183635	Centers for Disease Control & Prevention (CDC), Division of High Consequence Pathogens and Pathology (DHCPP-PRB)	Centers for Disease Control & Prevention (CDC), Division of High Consequence Pathogens and Pathology (DHCPP-PRB)	Gigante,C., Pettit,D., Zhao,H., Batra,D., Hetrick,E., Howard,D., Kovar,L., Seabolt,M., Morrison,S., Desch,M., Knipe,K., Weigand,M., Sheth,M., Burgin,A., Burroughs,M., Lee,J., Wilkins,K., McCollum,A., Hutson,C., Davidson,W., Rao,A., Deutsch-Feldman,M. and Li,Y.
EPI_ISL_16183636	Centers for Disease Control & Prevention (CDC), Division of High Consequence Pathogens and Pathology (DHCPP-PRB)	Centers for Disease Control & Prevention (CDC), Division of High Consequence Pathogens and Pathology (DHCPP-PRB)	Gigante,C., Ruiz,V., Zhao,H., Batra,D., Hetrick,E., Howard,D., Kovar,L., Seabolt,M., Morrison,S., Desch,M., Knipe,K., Weigand,M., Sheth,M., Burgin,A., Burroughs,M., Lee,J., Wilkins,K., McCollum,A., Hutson,C., Davidson,W., Rao,A., Wang,J. and Li,Y.
EPI_ISL_16183637	Centers for Disease Control & Prevention (CDC), Division of High Consequence Pathogens and Pathology (DHCPP-PRB)	Centers for Disease Control & Prevention (CDC), Division of High Consequence Pathogens and Pathology (DHCPP-PRB)	Gigante,C., Cogswell,K., Zhao,H., Batra,D., Hetrick,E., Howard,D., Kovar,L., Seabolt,M., Morrison,S., Desch,M., Knipe,K., Weigand,M., Sheth,M., Burgin,A., Burroughs,M., Lee,J., Wilkins,K., McCollum,A., Hutson,C., Davidson,W., Rao,A., Grenz,L. and Li,Y.
EPI_ISL_16183638, EPI_ISL_16183639, EPI_ISL_16183640, EPI_ISL_16183641, EPI_ISL_16183642	Centers for Disease Control & Prevention (CDC), Division of High Consequence Pathogens and Pathology (DHCPP-PRB)	Centers for Disease Control & Prevention (CDC), Division of High Consequence Pathogens and Pathology (DHCPP-PRB)	Gigante,C., Xia,D., Zhao,H., Batra,D., Hetrick,E., Howard,D., Kovar,L., Seabolt,M., Morrison,S., Desch,M., Knipe,K., Weigand,M., Sheth,M., Burgin,A., Burroughs,M., Lee,J., Wilkins,K., McCollum,A., Hutson,C., Davidson,W., Rao,A., Plipat,N. and Li,Y.
EPI_ISL_16183643	Centers for Disease Control & Prevention (CDC), Division of High Consequence Pathogens and Pathology (DHCPP-PRB)	Centers for Disease Control & Prevention (CDC), Division of High Consequence Pathogens and Pathology (DHCPP-PRB)	Gigante,C., Epie,N., Zhao,H., Batra,D., Hetrick,E., Howard,D., Kovar,L., Seabolt,M., Morrison,S., Desch,M., Knipe,K., Weigand,M., Sheth,M., Burgin,A., Burroughs,M., Lee,J., Wilkins,K., McCollum,A., Hutson,C., Davidson,W., Rao,A., Perez,T. and Li,Y.
EPI_ISL_16183644, EPI_ISL_16183645	Centers for Disease Control & Prevention (CDC), Division of High Consequence Pathogens and Pathology (DHCPP-PRB)	Centers for Disease Control & Prevention (CDC), Division of High Consequence Pathogens and Pathology (DHCPP-PRB)	Gigante,C., Thomas,L., Zhao,H., Batra,D., Hetrick,E., Howard,D., Kovar,L., Seabolt,M., Morrison,S., Desch,M., Knipe,K., Weigand,M., Sheth,M., Burgin,A., Burroughs,M., Lee,J., Wilkins,K., McCollum,A., Hutson,C., Davidson,W., Rao,A., Dunn,J. and Li,Y.
EPI_ISL_16183646, EPI_ISL_16183647, EPI_ISL_16183648	Centers for Disease Control & Prevention (CDC), Division of High Consequence Pathogens and Pathology (DHCPP-PRB)	Centers for Disease Control & Prevention (CDC), Division of High Consequence Pathogens and Pathology (DHCPP-PRB)	Gigante,C., Kubin,G., Zhao,H., Batra,D., Hetrick,E., Howard,D., Kovar,L., Seabolt,M., Morrison,S., Desch,M., Knipe,K., Sheth,M.R., Burgin,A., Burroughs,M., Lee,J., Wilkins,K., McCollum,A., Hutson,C., Davidson,W., Rao,A., White,S. and Li,Y.
EPI_ISL_16183649, EPI_ISL_16183650	Centers for Disease Control & Prevention (CDC), Division of High Consequence Pathogens and Pathology (DHCPP-PRB)	Centers for Disease Control & Prevention (CDC), Division of High Consequence Pathogens and Pathology (DHCPP-PRB)	Gigante,C., Mooring,E., Zhao,H., Batra,D., Hetrick,E., Howard,D., Kovar,L., Seabolt,M., Morrison,S., Desch,M., Knipe,K., Weigand,M., Sheth,M., Burgin,A., Burroughs,M., Lee,J., Wilkins,K., McCollum,A., Hutson,C., Davidson,W., Rao,A., Laurence,J. and Li,Y.
EPI_ISL_16183651	Centers for Disease Control & Prevention (CDC), Division of High Consequence Pathogens and Pathology (DHCPP-PRB)	Centers for Disease Control & Prevention (CDC), Division of High Consequence Pathogens and Pathology (DHCPP-PRB)	Gigante,C., Francis,D., Zhao,H., Batra,D., Hetrick,E., Howard,D., Kovar,L., Seabolt,M., Morrison,S., Desch,M., Knipe,K., Weigand,M., Sheth,M., Burgin,A., Burroughs,M., Lee,J., Wilkins,K., McCollum,A., Hutson,C., Davidson,W., Rao,A., Escobar,J. and Li,Y.
EPI_ISL_16183652, EPI_ISL_16183653	Centers for Disease Control & Prevention (CDC), Division of High Consequence Pathogens and Pathology (DHCPP-PRB)	Centers for Disease Control & Prevention (CDC), Division of High Consequence Pathogens and Pathology (DHCPP-PRB)	Gigante,C., Ventura,J., Zhao,H., Batra,D., Hetrick,E., Howard,D., Kovar,L., Seabolt,M., Morrison,S., Desch,M., Knipe,K., Weigand,M., Sheth,M., Burgin,A., Burroughs,M., Lee,J., Wilkins,K., McCollum,A., Hutson,C., Davidson,W., Rao,A., Nash,J. and Li,Y.

EPI_ISL_16183654, EPI_ISL_16183655	Centers for Disease Control & Prevention (CDC), Division of High Consequence Pathogens and Pathology (DHCPP-PRB)	Centers for Disease Control & Prevention (CDC), Division of High Consequence Pathogens and Pathology (DHCPP-PRB)	Gigante,C., Lee,P., Zhao,H., Batra,D., Hetrick,E., Howard,D., Kovar,L., Seabolt,M., Morrison,S., Desch,M., Knipe,K., Weigand,M., Sheth,M., Burgin,A., Burroughs,M., Lee,J., Wilkins,K., McCollum,A., Hutson,C., Davidson,W., Rao,A., Stanek,D. and Li,Y.
EPI_ISL_16190089, EPI_ISL_16190090, EPI_ISL_16190092, EPI_ISL_16190094, EPI_ISL_16190099	Rush University Medical Center	RIPHL at Rush University Medical Center	Stefan Green, Kevin Kunstman, Hannah Barbian, Felix Araujo Perez, Edith Perez, Sofiya Bobrovska, Alyse Kittner, Cecilia Chau, Giancarlo Balangue, Lok Yiu Ashley Wu, Mary Hayden, Joyce Houlihan, Diane Springer, Nicholas Moore
EPI_ISL_16222568, EPI_ISL_16222569	Centers for Disease Control & Prevention (CDC), Division of High Consequence Pathogens and Pathology (DHCPP-PRB)	Centers for Disease Control & Prevention (CDC), Division of High Consequence Pathogens and Pathology (DHCPP-PRB)	Gigante,C., Ruiz,V., Zhao,H., Batra,D., Hetrick,E., Howard,D., Kovar,L., Seabolt,M., Morrison,S., Desch,M., Knipe,K., Weigand,M., Sheth,M., Burgin,A., Burroughs,M., Lee,J., Wilkins,K., McCollum,A., Hutson,C., Davidson,W., Rao,A., Wang,J. and Li,Y.
EPI_ISL_16222570	Centers for Disease Control & Prevention (CDC), Division of High Consequence Pathogens and Pathology (DHCPP-PRB)	Centers for Disease Control & Prevention (CDC), Division of High Consequence Pathogens and Pathology (DHCPP-PRB)	Gigante,C., Hauser,J., Zhao,H., Batra,D., Hetrick,E., Howard,D., Kovar,L., Seabolt,M., Morrison,S., Desch,M., Knipe,K., Weigand,M., Sheth,M., Burgin,A., Burroughs,M., Lee,J., Wilkins,K., McCollum,A., Hutson,C., Davidson,W., Rao,A., Mangla,A. and Li,Y.
EPI_ISL_16222571	Centers for Disease Control & Prevention (CDC), Division of High Consequence Pathogens and Pathology (DHCPP-PRB)	Centers for Disease Control & Prevention (CDC), Division of High Consequence Pathogens and Pathology (DHCPP-PRB)	Gigante,C., Lee,P., Zhao,H., Batra,D., Hetrick,E., Howard,D., Kovar,L., Seabolt,M., Morrison,S., Desch,M., Knipe,K., Weigand,M., Sheth,M., Burgin,A., Burroughs,M., Lee,J., Wilkins,K., McCollum,A., Hutson,C., Davidson,W., Rao,A., Stanek,D. and Li,Y.
EPI_ISL_16222572, EPI_ISL_16222573	Centers for Disease Control & Prevention (CDC), Division of High Consequence Pathogens and Pathology (DHCPP-PRB)	Centers for Disease Control & Prevention (CDC), Division of High Consequence Pathogens and Pathology (DHCPP-PRB)	Gigante,C., Ghinai,I., Zhao,H., Batra,D., Hetrick,E., Howard,D., Kovar,L., Seabolt,M., Morrison,S., Desch,M., Knipe,K., Weigand,M., Sheth,M., Burgin,A., Burroughs,M., Lee,J., Wilkins,K., McCollum,A., Hutson,C., Davidson,W., Rao,A., Kerins,J. and Li,Y.
EPI_ISL_16233781	Complejo Hospitalario Universitario de Pontevedra	Microbiology Department. Complexo Hospitalario Universitario de Vigo	Daviña C, Pizcueta J, Trigo M, Perez-Castro S
EPI_ISL_16233782, EPI_ISL_16233783, EPI_ISL_16233784, EPI_ISL_16233785, EPI_ISL_16233786, EPI_ISL_16233787, EPI_ISL_16233788	Microbiology Department. Complexo Hospitalario Universitario de Vigo	Microbiology Department. Complexo Hospitalario Universitario de Vigo	Daviña C, Pizcueta J, Perez-Castro S
EPI_ISL_16260351	Centre Médical de l'Institut Pasteur	Cellule d'Intervention Biologique d'Urgence, Institut Pasteur	Charlotte Balière, Véronique Hourdel, Aurelia Kwasiborski, Quentin Grassin, Maxence Feher, Damien Hoinard, Jessica Vanhomwegen, Fabien Taieb, Paul-Henri Consigny, Jean-Claude Manuguerra, India Leclercq, Christophe Batéjat, Valérie Caro
EPI_ISL_16260402	Cellule d'Intervention Biologique d'Urgence, Institut Pasteur	Cellule d'Intervention Biologique d'Urgence, Institut Pasteur	Charlotte Balière, Véronique Hourdel, Aurelia Kwasiborski, Quentin Grassin, Maxence Feher, Damien Hoinard, Jessica Vanhomwegen, Fabien Taieb, Paul-Henri Consigny, Jean-Claude Manuguerra, India Leclercq, Christophe Batéjat, Valérie Caro
EPI_ISL_16299702, EPI_ISL_16299718	Azienda Sanitaria dell'Alto Adige - Laboratorio Aziendale di Microbiologia e Virologia	Azienda Sanitaria dell'Alto Adige - Laboratorio Aziendale di Microbiologia e Virologia	Teresa Fortini, Elisabetta Incrocci, Elisabetta Giacobazzi, Elisa Masi, Irene Bianconi, Elisabetta Pagani
EPI_ISL_16350819	Laboratorio de Biologia Molecular y Biotecnologia / Facultad de ciencias de la salud, Universidad Tecnologica de Pereira	Laboratorio de Biologia Molecular y Biotecnologia / Facultad de ciencias de la salud, Universidad Tecnologica de Pereira	Orjuela,M., Tabares,F.A., Anacona,J.D., Lopez,P.A., Zuluaga-Velez,A. and Sepulveda-Arias,J.C.
EPI_ISL_16350820	Laboratorio de Biologia Molecular y Biotecnologia / Facultad de ciencias de la salud, Universidad Tecnologica de Pereira	Laboratorio de Biologia Molecular y Biotecnologia / Facultad de ciencias de la salud, Universidad Tecnologica de Pereira	Tabares,F.A., Anacona,J.D., Lopez,P.A., Orjuela,M., Zuluaga-Velez,A. and Sepulveda-Arias,J.C.
EPI_ISL_16360909, EPI_ISL_16360911, EPI_ISL_16360913, EPI_ISL_16360915, EPI_ISL_16360917, EPI_ISL_16360919, EPI_ISL_16360921, EPI_ISL_16360922, EPI_ISL_16360924, EPI_ISL_16360927, EPI_ISL_16360929, EPI_ISL_16360931, EPI_ISL_16360932, EPI_ISL_16360935, EPI_ISL_16360937, EPI_ISL_16360939, EPI_ISL_16360941, EPI_ISL_16360943, EPI_ISL_16360946, EPI_ISL_16360948, EPI_ISL_16360950, EPI_ISL_16360952, EPI_ISL_16360954, EPI_ISL_16360955, EPI_ISL_16360957, EPI_ISL_16360959, EPI_ISL_16360961, EPI_ISL_16360963, EPI_ISL_16360965, EPI_ISL_16360967, EPI_ISL_16360969			
see above	Centre for Biological Threats, Highly Pathogenic Viruses, Robert Koch Institute	Centre for Biological Threats, Highly Pathogenic Viruses, Robert Koch Institute	Brinkmann,A., Kohl,C., Pape,K., Schrick,L., Michel,J., Schaade,L. and Nitsche,A.
EPI_ISL_16467111	IRCCS Sacro Cuore Don Calabria Hospital, Department of Infectious, Tropical Diseases & Microbiology	IRCCS Sacro Cuore Don Calabria Hospital, Department of Infectious, Tropical Diseases & Microbiology	Michela Deiana, Denise Lavezzari, Silvia Accordini, Concetta Castilletti, Antonio Mori, Elena Pomari, Chiara Piubelli
EPI_ISL_16505425	Hopital Saint Louis	Hopital Saint Louis	Zeggagh,J., Ferraris,O., Salmona,M., Tarantola,A., Molina,J.M. and Delaugerre,C.
EPI_ISL_16510131, EPI_ISL_16510132, EPI_ISL_16510134, EPI_ISL_16510136, EPI_ISL_16510138, EPI_ISL_16510140, EPI_ISL_16510141, EPI_ISL_16510143, EPI_ISL_16510145, EPI_ISL_16510147, EPI_ISL_16510148, EPI_ISL_16510150, EPI_ISL_16510153, EPI_ISL_16510154, EPI_ISL_16510155, EPI_ISL_16510156, EPI_ISL_16510157, EPI_ISL_16510158, EPI_ISL_16510159, EPI_ISL_16510162, EPI_ISL_16510163, EPI_ISL_16510164, EPI_ISL_16510165, EPI_ISL_16510166, EPI_ISL_16510167, EPI_ISL_16510168, EPI_ISL_16510169, EPI_ISL_16510170, EPI_ISL_16510171, EPI_ISL_16510172, EPI_ISL_16510173, EPI_ISL_16510174, EPI_ISL_16510175, EPI_ISL_16510177, EPI_ISL_16510178, EPI_ISL_16510179, EPI_ISL_16510180, EPI_ISL_16510181, EPI_ISL_16510182, EPI_ISL_16510183, EPI_ISL_16510184, EPI_ISL_16510185			
see above	National Virus Reference Laboratory	National Virus Reference Laboratory	Gabriel Gonzalez, Michael Carr, Brian Keogan, Jose Maria Urtasun Elizari, Jonathan Dean, Daniel Hare, Cillian F De Gascun
EPI_ISL_16526309	Direccion de Investigacion en Salud Publica, Instituto Nacional de Salud	Direccion de Investigacion en Salud Publica, Instituto Nacional de Salud	Laiton-Donato,K.D., Franco,C.E., Alvarez-Diaz,D.A., Ruiz-Moreno,H.A., Prada,D.A., Rosales,A. and Mercado-Reyes,M.M.
EPI_ISL_16588110, EPI_ISL_16588825, EPI_ISL_16589442	Erasmus Medical Center Department of Virology	Erasmus Medical Center Department of Virology	Leonard Schuele, Bas Oude Munnink, Marjan Boter, Babette Weller, Babs Verstrepen, Richard Molenkamp, Janette Rahamat-Langendoen, Reina Sikkema, Marion Koopmans
EPI_ISL_16645184	Microbiology Service of University Hospital of A Coruna (SERGAS)	Microbiology, Instituto de Investigacion Biomedica de A Coruña (INIBIC)	Macaya,P., Rumbo-Feal,S., Poza,M., Canizares,A., Vallejo,J.A. and Bou,G.
EPI_ISL_16645185	Microbiology Service of University Hospital of A Coruna (SERGAS	Microbiology, Instituto de Investigacion Biomedica de A Coruña (INIBIC)	Macaya,P., Rumbo-Feal,S., Poza,M., Canizares,A., Vallejo,J.A. and Bou,G.
EPI_ISL_16645186, EPI_ISL_16645187, EPI_ISL_16645188, EPI_ISL_16645189, EPI_ISL_16645190, EPI_ISL_16645192, EPI_ISL_16645193, EPI_ISL_16645194, EPI_ISL_16645195, EPI_ISL_16645196, EPI_ISL_16645197, EPI_ISL_16645198, EPI_ISL_16645199, EPI_ISL_16645200, EPI_ISL_16645201, EPI_ISL_16645202, EPI_ISL_16645203, EPI_ISL_16645204, EPI_ISL_16645205			
see above	Departamento de Genetica, Instituto de Biologia, Universidade federal do Rio de Janeiro	Departamento de Genetica, Instituto de Biologia, Universidade federal do Rio de Janeiro	Nunes,D.S., Higa,L.M., Oliveira,R.L., Costa,L.C., Bomfim,L.M., Goncalves,C.C.A., Mariane,D., Hruby,D.E., Voloch,C.M., Castineiras,T.M.P.P., Tanuri,A. and Damaso,C.R.
EPI_ISL_16645206	Division of High-risk Pathogens, Korea Disease Control and Prevention Agency	Division of High-risk Pathogens, Korea Disease Control and Prevention Agency	Rhie,G.-e.
EPI_ISL_16645207, EPI_ISL_16645208, EPI_ISL_16645209, EPI_ISL_16645211, EPI_ISL_16645212, EPI_ISL_16645213, EPI_ISL_16645214, EPI_ISL_16645215, EPI_ISL_16645216, EPI_ISL_16645218, EPI_ISL_16645219, EPI_ISL_16645220, EPI_ISL_16645221, EPI_ISL_16645223, EPI_ISL_16645224, EPI_ISL_16645226, EPI_ISL_16645227, EPI_ISL_16645228			
see above	Antioquia, Laboratorio Departamental de Salud Publica de Antioquia	Antioquia, Laboratorio Departamental de Salud Publica de Antioquia	Betancur,I.I.B., Velarde-Hoyos,C.-A.C.V., Gomez,R.R.G. and Mercado-Reyes,M.M.R.
EPI_ISL_16645229	Environment and Infectious Risks Unit, Insitut Pasteur	Environment and Infectious Risks Unit, Insitut Pasteur	Baliere,C., Hourdel,V., Kwasiborski,A., Grassin,Q., Feher,M., Hoinard,D., Vanhomwegen,J., Taieb,F., Consigny,P.-H., Manuguerra,J.-C., Leclercq,I., Batejat,C. and Caro,V.
EPI_ISL_16650246, EPI_ISL_16650247, EPI_ISL_16650248, EPI_ISL_16650249, EPI_ISL_16650251, EPI_ISL_16650260	Laboratorio Central de Saude Publica do Estado de Minas Gerais (Lacen-MG)	Laboratorio Central de Saude Publica do Estado de Minas Gerais (Lacen-MG)	Felipe Campos de Melo Iani, Ludmila Oliveira Lamounier, Luiz Marcelo Ribeiro Tomé, Natália Rocha Guimarães,Talita Emile Ribeiro Adelino.
EPI_ISL_16650297, EPI_ISL_16650298, EPI_ISL_16650299, EPI_ISL_16650300, EPI_ISL_16650301, EPI_ISL_16650302, EPI_ISL_16650303, EPI_ISL_16650304, EPI_ISL_16650305, EPI_ISL_16650307, EPI_ISL_16650309, EPI_ISL_16650311			
see above	Centre for Biological Threats, Highly Pathogenic Viruses, Robert Koch Institute	Centre for Biological Threats, Highly Pathogenic Viruses, Robert Koch Institute	Brinkmann,A., Kohl,C., Pape,K., Schrick,L., Michel,J., Schaade,L. and Nitsche,A.
EPI_ISL_16679203, EPI_ISL_16679204, EPI_ISL_16679206, EPI_ISL_16679207, EPI_ISL_16679208	Los Angeles County Public Health Laboratories	Los Angeles County Public Health Laboratories	P. Hemarajata et al.
EPI_ISL_16679209, EPI_ISL_16679210, EPI_ISL_16679212, EPI_ISL_16679213, EPI_ISL_16679214, EPI_ISL_16679215, EPI_ISL_16679216, EPI_ISL_16679217, EPI_ISL_16679219, EPI_ISL_16679220, EPI_ISL_16679221, EPI_ISL_16679222, EPI_ISL_16679223, EPI_ISL_16679224, EPI_ISL_16679225, EPI_ISL_16679226, EPI_ISL_16679227, EPI_ISL_16679229, EPI_ISL_16679230			
see above	Kaiser Permanente Chino Hills Regional Reference Laboratories	Los Angeles County Public Health Laboratories	P. Hemarajata et al.
EPI_ISL_16679232	Los Angeles County Public Health Laboratories	Los Angeles County Public Health Laboratories	P. Hemarajata et al.
EPI_ISL_16679235	Kaiser Permanente Chino Hills Regional Reference Laboratories	Los Angeles County Public Health Laboratories	P. Hemarajata et al.
EPI_ISL_16679236, EPI_ISL_16679237, EPI_ISL_16679238, EPI_ISL_16679240	Los Angeles County Public Health Laboratories	Los Angeles County Public Health Laboratories	P. Hemarajata et al.
EPI_ISL_16679242, EPI_ISL_16679243, EPI_ISL_16679244, EPI_ISL_16679245, EPI_ISL_16679246, EPI_ISL_16679247, EPI_ISL_16679248, EPI_ISL_16679249, EPI_ISL_16679250, EPI_ISL_16679251, EPI_ISL_16679252, EPI_ISL_16679253, EPI_ISL_16679254, EPI_ISL_16679255, EPI_ISL_16679256, EPI_ISL_16679257, EPI_ISL_16679258, EPI_ISL_16679259, EPI_ISL_16679260, EPI_ISL_16679261, EPI_ISL_16679262, EPI_ISL_16679263, EPI_ISL_16679264, EPI_ISL_16679265, EPI_ISL_16679266, EPI_ISL_16679267, EPI_ISL_16679268, EPI_ISL_16679269, EPI_ISL_16679270, EPI_ISL_16679271, EPI_ISL_16679272, EPI_ISL_16679273, EPI_ISL_16679274, EPI_ISL_16679275, EPI_ISL_16679276, EPI_ISL_16679277, EPI_ISL_16679278, EPI_ISL_16679279, EPI_ISL_16679280, EPI_ISL_16679281, EPI_ISL_16679282, EPI_ISL_16679283, EPI_ISL_16679284, EPI_ISL_16679285, EPI_ISL_16679286, EPI_ISL_16679287, EPI_ISL_16679288, EPI_ISL_16679289, EPI_ISL_16679290, EPI_ISL_16679291, EPI_ISL_16679292, EPI_ISL_16679293, EPI_ISL_16679294, EPI_ISL_16679295, EPI_ISL_16679296, EPI_ISL_16679297, EPI_ISL_16679298, EPI_ISL_16679299, EPI_ISL_16679300, EPI_ISL_16679301, EPI_ISL_16679302, EPI_ISL_16679303, EPI_ISL_16679304			
see above	California Department of Public Health	California Department of Public Health	Probert,W., Espinosa,A., Kath,C., Haw,M., O'Neil,R., Bell,J. and Hacker,J.
EPI_ISL_16727186, EPI_ISL_16727549	Erasmus Medical Center, Department of Virology	Erasmus Medical Center Department of Virology	Leonard Schuele, Bas Oude Munnink, Marjan Boter, Babette Weller, Babs Verstrepen, Richard Molenkamp, Janette Rahamat-Langendoen, Reina Sikkema, Marion Koopmans
EPI_ISL_16751080, EPI_ISL_16751081, EPI_ISL_16751082, EPI_ISL_16751083, EPI_ISL_16751084, EPI_ISL_16751085, EPI_ISL_16751086, EPI_ISL_16751087, EPI_ISL_16751088, EPI_ISL_16751089, EPI_ISL_16751090, EPI_ISL_16751091, EPI_ISL_16751092			
see above	Centre for Biological Threats, Highly Pathogenic Viruses, Robert Koch Institute	Centre for Biological Threats, Highly Pathogenic Viruses, Robert Koch Institute	Brinkmann,A., Kohl,C., Pape,K., Schrick,L., Michel,J., Schaade,L. and Nitsche,A.
EPI_ISL_16751093, EPI_ISL_16751094, EPI_ISL_16751095, EPI_ISL_16751096	Centers for Disease Control & Prevention (CDC), Division of High Consequence Pathogens and Pathology (DHCPP-PRB)	Centers for Disease Control & Prevention (CDC), Division of High Consequence Pathogens and Pathology (DHCPP-PRB)	Gigante,C., Kubin,G., Zhao,H., Batra,D., Hetrick,E., Howard,D., Kovar,L., Seabolt,M., Morrison,S., Desch,M., Knipe,K., Sheth,M.R., Burgin,A., Burroughs,M., Lee,J., Wilkins,K., McCollum,A., Hutson,C., Davidson,W., Rao,A., White,S. and Li,Y.
EPI_ISL_16751097, EPI_ISL_16751098	Centers for Disease Control & Prevention (CDC), Division of High Consequence Pathogens and	Centers for Disease Control & Prevention (CDC), Division of High Consequence Pathogens and	Gigante,C., Kubin,G., Zhao,H., Batra,D., Hetrick,E., Howard,D., Kovar,L., Seabolt,M., Morrison,S., Desch,M., Knipe,K., Weigand,M., Sheth,M., Burgin,A., Burroughs,M., Lee,J., Wilkins,K., McCollum,A., Hutson,C., Davidson,W., Rao,A., White,S. and Li,Y.

	Pathology (DHCPP-PRB)	Pathology (DHCPP-PRB)	
EPI_ISL_16751099, EPI_ISL_16751100	Centers for Disease Control & Prevention (CDC), Division of High Consequence Pathogens and Pathology (DHCPP-PRB)	Centers for Disease Control & Prevention (CDC), Division of High Consequence Pathogens and Pathology (DHCPP-PRB)	Gigante,C., Murray,J., Zhao,H., Batra,D., Hetrick,E., Howard,D., Kovar,L., Seabolt,M., Morrison,S., Desch,M., Knipe,K., Weigand,M., Sheth,M., Burroughs,A.B., Lee,J., Wilkins,K., McCollum,A., Hutson,C., Davidson,W., Rao,A., Atkinson,A. and Li,Y.
EPI_ISL_16751101, EPI_ISL_16751102, EPI_ISL_16751103, EPI_ISL_16751104, EPI_ISL_16751105, EPI_ISL_16751106, EPI_ISL_16751107	Centers for Disease Control & Prevention (CDC), Division of High Consequence Pathogens and Pathology (DHCPP-PRB)	Centers for Disease Control & Prevention (CDC), Division of High Consequence Pathogens and Pathology (DHCPP-PRB)	Gigante,C., Hauser,J., Zhao,H., Batra,D., Hetrick,E., Howard,D., Kovar,L., Seabolt,M., Morrison,S., Desch,M., Knipe,K., Weigand,M., Sheth,M., Burgin,A., Burroughs,M., Lee,J., Wilkins,K., McCollum,A., Hutson,C., Davidson,W., Rao,A., Mangla,A. and Li,Y.
EPI_ISL_16751108, EPI_ISL_16751109, EPI_ISL_16751110, EPI_ISL_16751111	Centers for Disease Control & Prevention (CDC), Division of High Consequence Pathogens and Pathology (DHCPP-PRB)	Centers for Disease Control & Prevention (CDC), Division of High Consequence Pathogens and Pathology (DHCPP-PRB)	Gigante,C., Bradley,A., Zhao,H., Batra,D., Hetrick,E., Howard,D., Kovar,L., Seabolt,M., Morrison,S., Desch,M., Knipe,K., Sheth,M.R., Burgin,A., Burroughs,M., Lee,J., Wilkins,K., McCollum,A., Hutson,C., Davidson,W., Rao,A., Anderson,J. and Li,Y.
EPI_ISL_16751112	Centers for Disease Control & Prevention (CDC), Division of High Consequence Pathogens and Pathology (DHCPP-PRB)	Centers for Disease Control & Prevention (CDC), Division of High Consequence Pathogens and Pathology (DHCPP-PRB)	Gigante,C., Johnson,S., Zhao,H., Batra,D., Hetrick,E., Howard,D., Kovar,L., Seabolt,M., Morrison,S., Weigand,M., Knipe,K., Sheth,M., Burgin,A., Burroughs,M., Lee,J., Wilkins,K., McCollum,A., Hutson,C., Davidson,W., Rao,A., Riner,D. and Li,Y.
EPI_ISL_16751113	Centers for Disease Control & Prevention (CDC), Division of High Consequence Pathogens and Pathology (DHCPP-PRB)	Centers for Disease Control & Prevention (CDC), Division of High Consequence Pathogens and Pathology (DHCPP-PRB)	Gigante,C., Cleavinger,K., Zhao,H., Batra,D., Hetrick,E., Howard,D., Kovar,L., Seabolt,M., Morrison,S., Desch,M., Knipe,K., Burroughs,M.R., Lee,J., Wilkins,K., McCollum,A., Hutson,C., Davidson,W., Rao,A., Sinn,M. and Li,Y.
EPI_ISL_16751114, EPI_ISL_16751115	Centers for Disease Control & Prevention (CDC), Division of High Consequence Pathogens and Pathology (DHCPP-PRB)	Centers for Disease Control & Prevention (CDC), Division of High Consequence Pathogens and Pathology (DHCPP-PRB)	Gigante,C., Mozer,M., Zhao,H., Batra,D., Hetrick,E., Howard,D., Kovar,L., Seabolt,M., Morrison,S., Desch,M., Knipe,K., Weigand,M., Sheth,M., Burgin,A., Burroughs,M., Lee,J., Wilkins,K., McCollum,A., Hutson,C., Davidson,W., Rao,A., Hopkins,B. and Li,Y.
EPI_ISL_16751116	Centers for Disease Control & Prevention (CDC), Division of High Consequence Pathogens and Pathology (DHCPP-PRB)	Centers for Disease Control & Prevention (CDC), Division of High Consequence Pathogens and Pathology (DHCPP-PRB)	Gigante,C., Buttery,E., Zhao,H., Batra,D., Hetrick,E., Howard,D., Kovar,L., Seabolt,M., Morrison,S., Desch,M., Knipe,K., Weigand,M., Sheth,M., Burroughs,A.B., Lee,J., Wilkins,K., McCollum,A., Hutson,C., Davidson,W., Rao,A., Raman,D. and Li,Y.
EPI_ISL_16751117, EPI_ISL_16751118, EPI_ISL_16751119, EPI_ISL_16751120, EPI_ISL_16751121, EPI_ISL_16751122	Centers for Disease Control & Prevention (CDC), Division of High Consequence Pathogens and Pathology (DHCPP-PRB)	Centers for Disease Control & Prevention (CDC), Division of High Consequence Pathogens and Pathology (DHCPP-PRB)	Gigante,C., Ruiz,V., Zhao,H., Batra,D., Hetrick,E., Howard,D., Kovar,L., Seabolt,M., Morrison,S., Desch,M., Knipe,K., Weigand,M., Sheth,M., Burgin,A., Burroughs,M., Lee,J., Wilkins,K., McCollum,A., Hutson,C., Davidson,W., Rao,A., Wang,J. and Li,Y.
EPI_ISL_16751123	Centers for Disease Control & Prevention (CDC), Division of High Consequence Pathogens and Pathology (DHCPP-PRB)	Centers for Disease Control & Prevention (CDC), Division of High Consequence Pathogens and Pathology (DHCPP-PRB)	Gigante,C., Thomas,L., Zhao,H., Batra,D., Hetrick,E., Howard,D., Kovar,L., Seabolt,M., Morrison,S., Desch,M., Knipe,K., Weigand,M., Sheth,M., Burgin,A., Burroughs,M., Lee,J., Wilkins,K., McCollum,A., Hutson,C., Davidson,W., Rao,A., Dunn,J. and Li,Y.
EPI_ISL_16751124	Centers for Disease Control & Prevention (CDC), Division of High Consequence Pathogens and Pathology (DHCPP-PRB)	Centers for Disease Control & Prevention (CDC), Division of High Consequence Pathogens and Pathology (DHCPP-PRB)	Gigante,C., Kubin,G., Zhao,H., Batra,D., Hetrick,E., Howard,D., Kovar,L., Seabolt,M., Morrison,S., Desch,M., Knipe,K., Sheth,M.R., Burgin,A., Burroughs,M., Lee,J., Wilkins,K., McCollum,A., Hutson,C., Davidson,W., Rao,A., White,S. and Li,Y.
EPI_ISL_16751125, EPI_ISL_16751126	Centers for Disease Control & Prevention (CDC), Division of High Consequence Pathogens and Pathology (DHCPP-PRB)	Centers for Disease Control & Prevention (CDC), Division of High Consequence Pathogens and Pathology (DHCPP-PRB)	Gigante,C., Pettit,D., Zhao,H., Batra,D., Hetrick,E., Howard,D., Kovar,L., Seabolt,M., Morrison,S., Desch,M., Knipe,K., Weigand,M., Sheth,M., Burgin,A., Burroughs,M., Lee,J., Wilkins,K., McCollum,A., Hutson,C., Davidson,W., Rao,A., Deutsch-Feldman,M. and Li,Y.
EPI_ISL_16751127, EPI_ISL_16751128, EPI_ISL_16751129	Centers for Disease Control & Prevention (CDC), Division of High Consequence Pathogens and Pathology (DHCPP-PRB)	Centers for Disease Control & Prevention (CDC), Division of High Consequence Pathogens and Pathology (DHCPP-PRB)	Gigante,C., Thomas,L., Zhao,H., Batra,D., Hetrick,E., Howard,D., Kovar,L., Seabolt,M., Morrison,S., Desch,M., Knipe,K., Weigand,M., Burroughs,M.S., Lee,J., Wilkins,K., McCollum,A., Hutson,C., Davidson,W., Rao,A., Dunn,J. and Li,Y.
EPI_ISL_16751130	Centers for Disease Control & Prevention (CDC), Division of High Consequence Pathogens and Pathology (DHCPP-PRB)	Centers for Disease Control & Prevention (CDC), Division of High Consequence Pathogens and Pathology (DHCPP-PRB)	Gigante,C., Kubin,G., Zhao,H., Batra,D., Hetrick,E., Howard,D., Kovar,L., Seabolt,M., Morrison,S., Desch,M., Knipe,K., Burroughs,M.R., Lee,J., Wilkins,K., McCollum,A., Hutson,C., Davidson,W., Rao,A., White,S. and Li,Y.
EPI_ISL_16751131	Centers for Disease Control & Prevention (CDC), Division of High Consequence Pathogens and Pathology (DHCPP-PRB)	Centers for Disease Control & Prevention (CDC), Division of High Consequence Pathogens and Pathology (DHCPP-PRB)	Gigante,C., Goldoft,M., Zhao,H., Batra,D., Hetrick,E., Howard,D., Kovar,L., Seabolt,M., Morrison,S., Desch,M., Knipe,K., Weigand,M., Burroughs,M.S., Lee,J., Wilkins,K., McCollum,A., Hutson,C., Davidson,W., Rao,A., Holshue,M. and Li,Y.
EPI_ISL_16751132	Centers for Disease Control & Prevention (CDC), Division of High Consequence Pathogens and Pathology (DHCPP-PRB)	Centers for Disease Control & Prevention (CDC), Division of High Consequence Pathogens and Pathology (DHCPP-PRB)	Gigante,C., Francis,D., Zhao,H., Batra,D., Hetrick,E., Howard,D., Kovar,L., Seabolt,M., Morrison,S., Desch,M., Knipe,K., Weigand,M., Burroughs,M.S., Lee,J., Wilkins,K., McCollum,A., Hutson,C., Davidson,W., Rao,A., Escobar,J. and Li,Y.
EPI_ISL_16751133	Centers for Disease Control & Prevention (CDC), Division of High Consequence Pathogens and Pathology (DHCPP-PRB)	Centers for Disease Control & Prevention (CDC), Division of High Consequence Pathogens and Pathology (DHCPP-PRB)	Gigante,C., Ventura,J., Zhao,H., Batra,D., Hetrick,E., Howard,D., Kovar,L., Seabolt,M., Morrison,S., Desch,M., Knipe,K., Weigand,M., Sheth,M., Burgin,A., Burroughs,M., Lee,J., Wilkins,K., McCollum,A., Hutson,C., Davidson,W., Rao,A., Nash,J. and Li,Y.
EPI_ISL_16751134	Centers for Disease Control & Prevention (CDC), Division of High Consequence Pathogens and Pathology (DHCPP-PRB)	Centers for Disease Control & Prevention (CDC), Division of High Consequence Pathogens and Pathology (DHCPP-PRB)	Gigante,C., Hauser,J., Zhao,H., Batra,D., Hetrick,E., Howard,D., Kovar,L., Seabolt,M., Knipe,K., Burroughs,M.S., Lee,J., Wilkins,K., McCollum,A., Hutson,C., Davidson,W., Rao,A., Mangla,A. and Li,Y.
EPI_ISL_16751135	Centers for Disease Control & Prevention (CDC), Division of High Consequence Pathogens and Pathology (DHCPP-PRB)	Centers for Disease Control & Prevention (CDC), Division of High Consequence Pathogens and Pathology (DHCPP-PRB)	Gigante,C., Lee,P., Zhao,H., Batra,D., Hetrick,E., Howard,D., Kovar,L., Seabolt,M., Morrison,S., Desch,M., Knipe,K., Weigand,M., Burroughs,M.S., Lee,J., Wilkins,K., McCollum,A., Hutson,C., Davidson,W., Rao,A., Stanek,D. and Li,Y.
EPI_ISL_16751136, EPI_ISL_16751137, EPI_ISL_16751138, EPI_ISL_16751139	Centers for Disease Control & Prevention (CDC), Division of High Consequence Pathogens and Pathology (DHCPP-PRB)	Centers for Disease Control & Prevention (CDC), Division of High Consequence Pathogens and Pathology (DHCPP-PRB)	Gigante,C., Pavlick,J., Zhao,H., Batra,D., Hetrick,E., Howard,D., Kovar,L., Seabolt,M., Morrison,S., Desch,M., Knipe,K., Weigand,M., Sheth,M., Burgin,A., Burroughs,M., Lee,J., Wilkins,K., McCollum,A., Hutson,C., Davidson,W., Rao,A., Parrott,T. and Li,Y.
EPI_ISL_16751140	Centers for Disease Control & Prevention (CDC), Division of High Consequence Pathogens and Pathology (DHCPP-PRB)	Centers for Disease Control & Prevention (CDC), Division of High Consequence Pathogens and Pathology (DHCPP-PRB)	Gigante,C., Culbertson,M., Zhao,H., Batra,D., Hetrick,E., Howard,D., Kovar,L., Seabolt,M., Weigand,M., Burroughs,M.S., Lee,J., Wilkins,K., McCollum,A., Hutson,C., Davidson,W., Rao,A., Pope,B. and Li,Y.
EPI_ISL_16751141	Centers for Disease Control & Prevention (CDC), Division of High Consequence Pathogens and Pathology (DHCPP-PRB)	Centers for Disease Control & Prevention (CDC), Division of High Consequence Pathogens and Pathology (DHCPP-PRB)	Gigante,C., Haydel,D., Zhao,H., Batra,D., Hetrick,E., Howard,D., Kovar,L., Seabolt,M., Weigand,M., Burroughs,M.S., Lee,J., Wilkins,K., McCollum,A., Hutson,C., Davidson,W., Rao,A., Salinas,A. and Li,Y.
EPI_ISL_16751142, EPI_ISL_16751143, EPI_ISL_16751144	Centers for Disease Control & Prevention (CDC), Division of High Consequence Pathogens and Pathology (DHCPP-PRB)	Centers for Disease Control & Prevention (CDC), Division of High Consequence Pathogens and Pathology (DHCPP-PRB)	Gigante,C., Ostadkar,R., Zhao,H., Batra,D., Hetrick,E., Howard,D., Kovar,L., Seabolt,M., Weigand,M., Knipe,K., Burroughs,M.S., Lee,J., Wilkins,K., McCollum,A., Hutson,C., Davidson,W., Rao,A., Wang,X. and Li,Y.
EPI_ISL_16751145	Centers for Disease Control & Prevention (CDC), Division of High Consequence Pathogens and Pathology (DHCPP-PRB)	Centers for Disease Control & Prevention (CDC), Division of High Consequence Pathogens and Pathology (DHCPP-PRB)	Gigante,C., Kubin,G., Zhao,H., Batra,D., Hetrick,E., Howard,D., Kovar,L., Seabolt,M., Morrison,S., Desch,M., Knipe,K., Burroughs,M.R., Lee,J., Wilkins,K., McCollum,A., Hutson,C., Davidson,W., Rao,A., White,S. and Li,Y.
EPI_ISL_16758555, EPI_ISL_16758556, EPI_ISL_16758557, EPI_ISL_16758558, EPI_ISL_16758559, EPI_ISL_16758560, EPI_ISL_16758561, EPI_ISL_16758562, EPI_ISL_16758563, EPI_ISL_16758564, EPI_ISL_16758565			
see above	Centre for Biological Threats, Highly Pathogenic Viruses, Robert Koch Institute	Centre for Biological Threats, Highly Pathogenic Viruses, Robert Koch Institute	Brinkmann,A., Kohl,C., Pape,K., Schrick,L., Michel,J., Schaade,L. and Nitsche,A.
EPI_ISL_16847486, EPI_ISL_16847487	Institute for Medical Virology, University Hospital, Goethe University	Institute for Medical Virology, University Hospital, Goethe University	Denisa Bojkova, Julia Schneider, Victor M. Corman, Martin Michaelis, Jindrich Cinatl jr.
EPI_ISL_16871158, EPI_ISL_16871159, EPI_ISL_16871160, EPI_ISL_16871161, EPI_ISL_16871162, EPI_ISL_16871163	Laboratorio de Enterovirus, Instituto Oswaldo Cruz, Fiocruz	Instituto Oswaldo Cruz FIOCRUZ - Laboratory of Respiratory Viruses and Measles (LVRS)	Paola Resende, Elisa Cavalcante Pereira, Bruna Mendonça da Silva, Jéssica Graça Macedo de Carvalho, Larissa Macedo Pinto, Victor Guimaraes, Marilda Siqueira, Renan da Silva Faustino, Marilia Santini, Edson Elias da Silva on behalf of the Fiocruz Genomic Surveillance Network
EPI_ISL_16905442, EPI_ISL_16905443, EPI_ISL_16905444	Tokyo Metropolitan Institute of Public Health, Department of Microbiology	Tokyo Metropolitan Institute of Public Health, Department of Microbiology	Kasuya,F., Negishi,A., Kumagai,R., Hasegawa,M., Fujiwara,T., Miyake,H., Nagashima,M. and Sadamasu,K.
EPI_ISL_16926988, EPI_ISL_16926991, EPI_ISL_16926994, EPI_ISL_16926997, EPI_ISL_16927000, EPI_ISL_16927003, EPI_ISL_16927007, EPI_ISL_16927010, EPI_ISL_16927013, EPI_ISL_16927016, EPI_ISL_16927018, EPI_ISL_16927021			
see above	Naval Infectious Diseases Diagnostic Laboratory	Naval Medical Research Center Biological Defense Research Directorate	Logan J. Voegtly, Gregory K. Rice, Adrian Pakey, Andrea E. Luquette, Maren C. Fitzpatrick, Hannah M. Drumm, Victor Sugiharto, Hua-Wei Chen, Francisco Malagon, Regina Z. Cer, Kimberly A. Bishop-Lilly
EPI_ISL_16930148, EPI_ISL_16930151, EPI_ISL_16930154, EPI_ISL_16930157, EPI_ISL_16930160, EPI_ISL_16930165, EPI_ISL_16930168, EPI_ISL_16930171, EPI_ISL_16930174, EPI_ISL_16930177, EPI_ISL_16930180, EPI_ISL_16930183			
see above	California Department of Public Health	California Department of Public Health	Probert,W., Espinosa,A., Kath,C., Haw,M., O'Neil,R., Bell,J. and Hacker,J.
EPI_ISL_16946400	Division de Microbiologia, Hospital Nacional de Niños Carlos Saenz Herrera	Instituto Costarricense de Investigacion y Enseñanza en Nutricion y Salud, Inciensa	Diana Cantillo, Hillary Serrano, Ana Isela Ruiz, Gustavo Vega, Claudio Soto-Garita, Adriana Godinez, Estela Cordero, Melany Calderon, Francisco Duarte
EPI_ISL_16955153, EPI_ISL_16955154, EPI_ISL_16955155, EPI_ISL_16955156, EPI_ISL_16955157, EPI_ISL_16955158, EPI_ISL_16955159, EPI_ISL_16955160	CT Department of Public Health	CT Department of Public Health	Claire Pearson, Tu N. Nguyen, Kutluhan Incekara, Neranjan V. Perera
EPI_ISL_16955204, EPI_ISL_16955205, EPI_ISL_16955206, EPI_ISL_16955207, EPI_ISL_16955208, EPI_ISL_16955209, EPI_ISL_16955210, EPI_ISL_16955211, EPI_ISL_16955212, EPI_ISL_16955213, EPI_ISL_16955214, EPI_ISL_16955215, EPI_ISL_16955216, EPI_ISL_16955217, EPI_ISL_16955218, EPI_ISL_16955219, EPI_ISL_16955220, EPI_ISL_16955221, EPI_ISL_16955222			
see above	Public Health Laboratory, NYC Department of Health and Mental Hygiene	Public Health Laboratory, NYC Department of Health and Mental Hygiene	Wang,J.C., Amin,H.S., Clabby,T.T., Taki,F., Su,M., Rahat,A., De La Cruz,N., Olsen,A., Thi,C., Silver,S., Akther,S., Chowdhury,M., Omoregie,E. and Hughes,S.
EPI_ISL_16955223, EPI_ISL_16955224, EPI_ISL_16955225, EPI_ISL_16955226	Centre for Biological Threats, Highly Pathogenic Viruses, Robert Koch Institute	Centre for Biological Threats, Highly Pathogenic Viruses, Robert Koch Institute	Brinkmann,A., Kohl,C., Pape,K., Schrick,L., Michel,J., Schaade,L. and Nitsche,A.

EPI_ISL_16955227, EPI_ISL_16955228, EPI_ISL_16955229, EPI_ISL_16955230	Public Health Laboratory, NYC Department of Health and Mental Hygiene	Public Health Laboratory, NYC Department of Health and Mental Hygiene	Wang,J.C., Amin,H.S., Clabby,T.T., Taki,F., Su,M., Rahat,A., De La Cruz,N., Olsen,A., Thi,C., Silver,S., Akther,S., Chowdhury,M., Omoregie,E. and Hughes,S.
EPI_ISL_16955231, EPI_ISL_16955232, EPI_ISL_16955233, EPI_ISL_16955234, EPI_ISL_16955235, EPI_ISL_16955237, EPI_ISL_16955238	Centre for Biological Threats, Highly Pathogenic Viruses, Robert Koch Institute	Centre for Biological Threats, Highly Pathogenic Viruses, Robert Koch Institute	Brinkmann,A., Kohl,C., Pape,K., Schrick,L., Michel,J., Schaade,L. and Nitsche,A.
EPI_ISL_16955239, EPI_ISL_16955240, EPI_ISL_16955241, EPI_ISL_16955242, EPI_ISL_16955243, EPI_ISL_16955244, EPI_ISL_16955245, EPI_ISL_16955246, EPI_ISL_16955247, EPI_ISL_16955248, EPI_ISL_16955249, EPI_ISL_16955250, EPI_ISL_16955251, EPI_ISL_16955252, EPI_ISL_16955253, EPI_ISL_16955254, EPI_ISL_16955255, EPI_ISL_16955256, EPI_ISL_16955257, EPI_ISL_16955258, EPI_ISL_16955259, EPI_ISL_16955260, EPI_ISL_16955261, EPI_ISL_16955262, EPI_ISL_16955263, EPI_ISL_16955264, EPI_ISL_16955265, EPI_ISL_16955266, EPI_ISL_16955267, EPI_ISL_16955268, EPI_ISL_16955269, EPI_ISL_16955270, EPI_ISL_16955271, EPI_ISL_16955272, EPI_ISL_16955273, EPI_ISL_16955274, EPI_ISL_16955275, EPI_ISL_16955276, EPI_ISL_16955277, EPI_ISL_16955278, EPI_ISL_16955279, EPI_ISL_16955283, EPI_ISL_16955284, EPI_ISL_16955285, EPI_ISL_16955286, EPI_ISL_16955287, EPI_ISL_16955288, EPI_ISL_16955289, EPI_ISL_16955290			
see above	Public Health Laboratory, NYC Department of Health and Mental Hygiene	Public Health Laboratory, NYC Department of Health and Mental Hygiene	Wang,J.C., Amin,H.S., Clabby,T.T., Taki,F., Su,M., Rahat,A., De La Cruz,N., Olsen,A., Thi,C., Silver,S., Akther,S., Chowdhury,M., Omoregie,E. and Hughes,S.
EPI_ISL_16955293	Laboratory of Virology, University Hospitals of Geneva	Laboratory of Virology, University Hospitals of Geneva	Laubscher,F., Chudzinsk,V., Schibler,M., Kaiser,L. and Renzoni,A.
EPI_ISL_16955294, EPI_ISL_16955295, EPI_ISL_16955296, EPI_ISL_16955297, EPI_ISL_16955298, EPI_ISL_16955299, EPI_ISL_16955300, EPI_ISL_16955302, EPI_ISL_16955303, EPI_ISL_16955304, EPI_ISL_16955305, EPI_ISL_16955306, EPI_ISL_16955307, EPI_ISL_16955308, EPI_ISL_16955309, EPI_ISL_16955310, EPI_ISL_16955311, EPI_ISL_16955312, EPI_ISL_16955313, EPI_ISL_16955314, EPI_ISL_16955315, EPI_ISL_16955316			
see above	Public Health Laboratory, NYC Department of Health and Mental Hygiene	Public Health Laboratory, NYC Department of Health and Mental Hygiene	Wang,J.C., Amin,H.S., Clabby,T.T., Taki,F., Su,M., Rahat,A., De La Cruz,N., Olsen,A., Thi,C., Silver,S., Akther,S., Chowdhury,M., Omoregie,E. and Hughes,S.
EPI_ISL_16985950, EPI_ISL_16985951, EPI_ISL_16985952, EPI_ISL_16985954, EPI_ISL_16985955, EPI_ISL_16985956, EPI_ISL_16985957, EPI_ISL_16985958, EPI_ISL_16985959, EPI_ISL_16985960, EPI_ISL_16985961, EPI_ISL_16985962, EPI_ISL_16985963, EPI_ISL_16985964, EPI_ISL_16985965, EPI_ISL_16985966, EPI_ISL_16985967, EPI_ISL_16985968, EPI_ISL_16985969, EPI_ISL_16985970, EPI_ISL_16985971, EPI_ISL_16985972			
see above	National Virus Reference Laboratory	National Virus Reference Laboratory	Gabriel Gonzalez, Michael Carr, Emer O'Byrne, Weronika Banka, Brian Keogan, Jose Maria Urtasun Elizari, Jonathan Dean, Daniel Hare, Cillian F De Gascun
EPI_ISL_16987277	Centers for Disease Control & Prevention (CDC), Division of High Consequence Pathogens and Pathology (DHCPP-PRB)	Centers for Disease Control & Prevention (CDC), Division of High Consequence Pathogens and Pathology (DHCPP-PRB)	Gigante,C., Buttery,E., Zhao,H., Batra,D., Hetrick,E., Howard,D., Kovar,L., Seabolt,M., Knipe,K., Burroughs,M.S., Lee,J., Wilkins,K., McCollum,A., Hutson,C., Davidson,W., Rao,A., Raman,D. and Li,Y.
EPI_ISL_16987278, EPI_ISL_16987279, EPI_ISL_16987280, EPI_ISL_16987281, EPI_ISL_16987282, EPI_ISL_16987283	Centers for Disease Control & Prevention (CDC), Division of High Consequence Pathogens and Pathology (DHCPP-PRB)	Centers for Disease Control & Prevention (CDC), Division of High Consequence Pathogens and Pathology (DHCPP-PRB)	Gigante,C., Ruiz,V., Zhao,H., Batra,D., Hetrick,E., Howard,D., Kovar,L., Seabolt,M., Morrison,S., Desch,M., Knipe,K., Weigand,M., Sheth,M., Burgin,A., Burroughs,M., Lee,J., Wilkins,K., McCollum,A., Hutson,C., Davidson,W., Rao,A., Wang,J. and Li,Y.
EPI_ISL_16987284	Centers for Disease Control & Prevention (CDC), Division of High Consequence Pathogens and Pathology (DHCPP-PRB)	Centers for Disease Control & Prevention (CDC), Division of High Consequence Pathogens and Pathology (DHCPP-PRB)	Gigante,C., Lee,B., Zhao,H., Batra,D., Hetrick,E., Howard,D., Kovar,L., Seabolt,M., Weigand,M., Knipe,K., Burroughs,M.S., Lee,J., Wilkins,K., McCollum,A., Hutson,C., Davidson,W., Rao,A., Salehi,E. and Li,Y.
EPI_ISL_16987285, EPI_ISL_16987286	Centers for Disease Control & Prevention (CDC), Division of High Consequence Pathogens and Pathology (DHCPP-PRB)	Centers for Disease Control & Prevention (CDC), Division of High Consequence Pathogens and Pathology (DHCPP-PRB)	Gigante,C., Cogswell,K., Zhao,H., Batra,D., Hetrick,E., Howard,D., Kovar,L., Seabolt,M., Morrison,S., Desch,M., Knipe,K., Weigand,M., Sheth,M., Burgin,A., Burroughs,M., Lee,J., Wilkins,K., McCollum,A., Hutson,C., Davidson,W., Rao,A., Grenz,L. and Li,Y.
EPI_ISL_16987287	Centers for Disease Control & Prevention (CDC), Division of High Consequence Pathogens and Pathology (DHCPP-PRB)	Centers for Disease Control & Prevention (CDC), Division of High Consequence Pathogens and Pathology (DHCPP-PRB)	Gigante,C., Xia,D., Zhao,H., Batra,D., Hetrick,E., Howard,D., Kovar,L., Seabolt,M., Morrison,S., Desch,M., Knipe,K., Weigand,M., Sheth,M., Burgin,A., Burroughs,M., Lee,J., Wilkins,K., McCollum,A., Hutson,C., Davidson,W., Rao,A., Plipat,N. and Li,Y.
EPI_ISL_16987288	Centers for Disease Control & Prevention (CDC), Division of High Consequence Pathogens and Pathology (DHCPP-PRB)	Centers for Disease Control & Prevention (CDC), Division of High Consequence Pathogens and Pathology (DHCPP-PRB)	Gigante,C., Thomas,L., Zhao,H., Batra,D., Hetrick,E., Howard,D., Kovar,L., Seabolt,M., Morrison,S., Desch,M., Knipe,K., Weigand,M., Sheth,M., Burgin,A., Burroughs,M., Lee,J., Wilkins,K., McCollum,A., Hutson,C., Davidson,W., Rao,A., Dunn,J. and Li,Y.
EPI_ISL_16987289, EPI_ISL_16987290, EPI_ISL_16987291, EPI_ISL_16987292	Centers for Disease Control & Prevention (CDC), Division of High Consequence Pathogens and Pathology (DHCPP-PRB)	Centers for Disease Control & Prevention (CDC), Division of High Consequence Pathogens and Pathology (DHCPP-PRB)	Gigante,C., Kubin,G., Zhao,H., Batra,D., Hetrick,E., Howard,D., Kovar,L., Seabolt,M., Morrison,S., Desch,M., Knipe,K., Sheth,M.R., Burgin,A., Burroughs,M., Lee,J., Wilkins,K., McCollum,A., Hutson,C., Davidson,W., Rao,A., White,S. and Li,Y.
EPI_ISL_16987293, EPI_ISL_16987294	Centers for Disease Control & Prevention (CDC), Division of High Consequence Pathogens and Pathology (DHCPP-PRB)	Centers for Disease Control & Prevention (CDC), Division of High Consequence Pathogens and Pathology (DHCPP-PRB)	Gigante,C., Murray,J., Zhao,H., Batra,D., Hetrick,E., Howard,D., Kovar,L., Seabolt,M., Knipe,K., Burroughs,M.S., Lee,J., Wilkins,K., McCollum,A., Hutson,C., Davidson,W., Rao,A., Atkinson,A. and Li,Y.
EPI_ISL_16987295	Centers for Disease Control & Prevention (CDC), Division of High Consequence Pathogens and Pathology (DHCPP-PRB)	Centers for Disease Control & Prevention (CDC), Division of High Consequence Pathogens and Pathology (DHCPP-PRB)	Gigante,C., Goldoft,M., Zhao,H., Batra,D., Hetrick,E., Howard,D., Kovar,L., Seabolt,M., Morrison,S., Desch,M., Knipe,K., Weigand,M., Burroughs,M.S., Lee,J., Wilkins,K., McCollum,A., Hutson,C., Davidson,W., Rao,A., Holshue,M. and Li,Y.
EPI_ISL_16987296, EPI_ISL_16987297, EPI_ISL_16987298, EPI_ISL_16987299	Centers for Disease Control & Prevention (CDC), Division of High Consequence Pathogens and Pathology (DHCPP-PRB)	Centers for Disease Control & Prevention (CDC), Division of High Consequence Pathogens and Pathology (DHCPP-PRB)	Gigante,C., Ventura,J., Zhao,H., Batra,D., Hetrick,E., Howard,D., Kovar,L., Seabolt,M., Morrison,S., Desch,M., Knipe,K., Weigand,M., Sheth,M., Burgin,A., Burroughs,M., Lee,J., Wilkins,K., McCollum,A., Hutson,C., Davidson,W., Rao,A., Nash,J. and Li,Y.
EPI_ISL_16987300, EPI_ISL_16987301	Centers for Disease Control & Prevention (CDC), Division of High Consequence Pathogens and Pathology (DHCPP-PRB)	Centers for Disease Control & Prevention (CDC), Division of High Consequence Pathogens and Pathology (DHCPP-PRB)	Gigante,C., Steidley,B., Seabolt,M., Zhao,H., Wilkins,K., McCollum,A., Hutson,C., Davidson,W., Rao,A., Davizon,E. and Li,Y.
EPI_ISL_16987302, EPI_ISL_16987303, EPI_ISL_16987380, EPI_ISL_16987381	Centers for Disease Control & Prevention (CDC), Division of High Consequence Pathogens and Pathology (DHCPP-PRB)	Centers for Disease Control & Prevention (CDC), Division of High Consequence Pathogens and Pathology (DHCPP-PRB)	Gigante,C., Hauser,J., Zhao,H., Batra,D., Hetrick,E., Howard,D., Kovar,L., Seabolt,M., Morrison,S., Desch,M., Knipe,K., Weigand,M., Sheth,M., Burgin,A., Burroughs,M., Lee,J., Wilkins,K., McCollum,A., Hutson,C., Davidson,W., Rao,A., Mangla,A. and Li,Y.
EPI_ISL_16987382, EPI_ISL_16987383, EPI_ISL_16987384, EPI_ISL_16987385	Centers for Disease Control & Prevention (CDC), Division of High Consequence Pathogens and Pathology (DHCPP-PRB)	Centers for Disease Control & Prevention (CDC), Division of High Consequence Pathogens and Pathology (DHCPP-PRB)	Gigante,C., Lee,P., Zhao,H., Batra,D., Hetrick,E., Howard,D., Kovar,L., Seabolt,M., Morrison,S., Desch,M., Knipe,K., Weigand,M., Sheth,M., Burgin,A., Burroughs,M., Lee,J., Wilkins,K., McCollum,A., Hutson,C., Davidson,W., Rao,A., Stanek,D. and Li,Y.
EPI_ISL_16987386	Centers for Disease Control & Prevention (CDC), Division of High Consequence Pathogens and Pathology (DHCPP-PRB)	Centers for Disease Control & Prevention (CDC), Division of High Consequence Pathogens and Pathology (DHCPP-PRB)	Gigante,C., Pavlick,J., Zhao,H., Batra,D., Hetrick,E., Howard,D., Kovar,L., Seabolt,M., Morrison,S., Desch,M., Knipe,K., Weigand,M., Sheth,M., Burgin,A., Burroughs,M., Lee,J., Wilkins,K., McCollum,A., Hutson,C., Davidson,W., Rao,A., Parrott,T. and Li,Y.
EPI_ISL_16987387	Centers for Disease Control & Prevention (CDC), Division of High Consequence Pathogens and Pathology (DHCPP-PRB)	Centers for Disease Control & Prevention (CDC), Division of High Consequence Pathogens and Pathology (DHCPP-PRB)	Gigante,C., Ceniseros,A., Zhao,H., Batra,D., Hetrick,E., Howard,D., Kovar,L., Seabolt,M., Morrison,S., Desch,M., Knipe,K., Weigand,M., Burroughs,M.S., Lee,J., Wilkins,K., McCollum,A., Hutson,C., Davidson,W., Rao,A., Cahill,M. and Li,Y.
EPI_ISL_16987388, EPI_ISL_16987389	Centers for Disease Control & Prevention (CDC), Division of High Consequence Pathogens and Pathology (DHCPP-PRB)	Centers for Disease Control & Prevention (CDC), Division of High Consequence Pathogens and Pathology (DHCPP-PRB)	Gigante,C., Ghinai,I., Zhao,H., Batra,D., Hetrick,E., Howard,D., Kovar,L., Seabolt,M., Morrison,S., Desch,M., Knipe,K., Weigand,M., Sheth,M., Burgin,A., Burroughs,M., Lee,J., Wilkins,K., McCollum,A., Hutson,C., Davidson,W., Rao,A., Kerins,J. and Li,Y.
EPI_ISL_16987390	Centers for Disease Control & Prevention (CDC), Division of High Consequence Pathogens and Pathology (DHCPP-PRB)	Centers for Disease Control & Prevention (CDC), Division of High Consequence Pathogens and Pathology (DHCPP-PRB)	Gigante,C., Salinas,A., Zhao,H., Batra,D., Hetrick,E., Howard,D., Kovar,L., Seabolt,M., Morrison,S., Desch,M., Knipe,K., Burroughs,M.R., Lee,J., Wilkins,K., McCollum,A., Hutson,C., Davidson,W., Rao,A., Haydel,D. and Li,Y.
EPI_ISL_16987391, EPI_ISL_16987392, EPI_ISL_16987393	Centers for Disease Control & Prevention (CDC), Division of High Consequence Pathogens and Pathology (DHCPP-PRB)	Centers for Disease Control & Prevention (CDC), Division of High Consequence Pathogens and Pathology (DHCPP-PRB)	Gigante,C., Johnson,S., Zhao,H., Batra,D., Hetrick,E., Howard,D., Kovar,L., Seabolt,M., Weigand,M., Knipe,K., Burroughs,M.S., Lee,J., Wilkins,K., McCollum,A., Hutson,C., Davidson,W., Rao,A., Riner,D. and Li,Y.
EPI_ISL_16997389, EPI_ISL_16997390, EPI_ISL_16997391, EPI_ISL_16997392, EPI_ISL_16997393, EPI_ISL_16997394, EPI_ISL_16997395, EPI_ISL_16997396, EPI_ISL_16997397, EPI_ISL_16997398, EPI_ISL_16997399, EPI_ISL_16997400, EPI_ISL_16997401, EPI_ISL_16997402, EPI_ISL_16997403			
see above	California Department of Public Health	California Department of Public Health	Probert,W., Espinosa,A., Kath,C., Haw,M., O'Neil,R., Bell,J. and Hacker,J.
EPI_ISL_16997404, EPI_ISL_16997405, EPI_ISL_16997406	Quest Diagnostics Nichols Institute	Los Angeles County Public Health Laboratories	P. Hemarajata et al.
EPI_ISL_16997407, EPI_ISL_16997411	Los Angeles County Public Health Laboratories	Los Angeles County Public Health Laboratories	P. Hemarajata et al.
EPI_ISL_16997413, EPI_ISL_16997415, EPI_ISL_16997417, EPI_ISL_16997418, EPI_ISL_16997419, EPI_ISL_16997421, EPI_ISL_16997422, EPI_ISL_16997423, EPI_ISL_16997424, EPI_ISL_16997425, EPI_ISL_16997426, EPI_ISL_16997427, EPI_ISL_16997432, EPI_ISL_16997433, EPI_ISL_16997434, EPI_ISL_16997435, EPI_ISL_16997437, EPI_ISL_16997439, EPI_ISL_16997440, EPI_ISL_16997441, EPI_ISL_16997442, EPI_ISL_16997443, EPI_ISL_16997444, EPI_ISL_16997445, EPI_ISL_16997446			
see above	Kaiser Permanente Chino Hills Regional Reference Laboratories	Los Angeles County Public Health Laboratories	P. Hemarajata et al.
EPI_ISL_16997447, EPI_ISL_16997449, EPI_ISL_16997450, EPI_ISL_16997451, EPI_ISL_16997452, EPI_ISL_16997453	Laboratory Corporation of America	Los Angeles County Public Health Laboratories	P. Hemarajata et al.
EPI_ISL_16997455, EPI_ISL_16997456, EPI_ISL_16997457, EPI_ISL_16997458	Los Angeles County Public Health Laboratories	Los Angeles County Public Health Laboratories	P. Hemarajata et al.
EPI_ISL_16997460, EPI_ISL_16997461, EPI_ISL_16997462, EPI_ISL_16997463, EPI_ISL_16997464, EPI_ISL_16997465, EPI_ISL_16997466, EPI_ISL_16997467, EPI_ISL_16997468, EPI_ISL_16997469	Quest Diagnostics Nichols Institute	Los Angeles County Public Health Laboratories	P. Hemarajata et al.
EPI_ISL_16997470	UCLA Clinical Micro Lab	Los Angeles County Public Health Laboratories	P. Hemarajata et al.
EPI_ISL_16999059, EPI_ISL_16999060, EPI_ISL_16999062, EPI_ISL_16999064, EPI_ISL_16999065, EPI_ISL_16999066, EPI_ISL_16999067, EPI_ISL_16999068, EPI_ISL_16999069, EPI_ISL_16999070, EPI_ISL_16999071, EPI_ISL_16999073, EPI_ISL_16999074, EPI_ISL_16999075, EPI_ISL_16999076, EPI_ISL_16999077, EPI_ISL_16999078, EPI_ISL_16999079, EPI_ISL_16999081, EPI_ISL_16999082, EPI_ISL_16999084, EPI_ISL_16999085, EPI_ISL_16999086, EPI_ISL_16999087, EPI_ISL_16999090, EPI_ISL_16999091, EPI_ISL_16999093, EPI_ISL_16999095, EPI_ISL_16999098, EPI_ISL_16999099, EPI_ISL_16999100, EPI_ISL_16999101, EPI_ISL_16999102, EPI_ISL_16999104, EPI_ISL_16999105, EPI_ISL_16999108, EPI_ISL_16999110, EPI_ISL_16999111, EPI_ISL_16999113, EPI_ISL_16999114, EPI_ISL_16999115, EPI_ISL_16999116, EPI_ISL_16999117, EPI_ISL_16999120, EPI_ISL_16999121, EPI_ISL_16999123, EPI_ISL_16999124, EPI_ISL_16999125, EPI_ISL_16999126, EPI_ISL_16999127, EPI_ISL_16999128, EPI_ISL_16999129, EPI_ISL_16999131, EPI_ISL_16999132, EPI_ISL_16999133, EPI_ISL_16999134, EPI_ISL_16999137, EPI_ISL_16999138, EPI_ISL_16999139			

see above	Laboratorio de Referencia Nacional de Virus Inmunoprevenibles. Centro Nacional de Salud Publica. Instiuto Nacional de Salud	Equipo de Vigilancia Genomica. Area de Innovación y Desarrollo. Centro Nacional de Salud Publica. Instiuto Nacional de Salud	Carlos Patricio Padilla Rojas, Carmen Verónica Hurtado Vela, Juana Iris Silva Molina, Luis Bárcena Flores, Víctor Jiménez Vásquez, Alicia Elizabeth Núñez Llanos, Wendy Lizarraga Olivares, Luren Nieves Sevilla Catañeda, Kelly Vanessa Izarra Rojas, Karla Vasquez Cajachahua, Steve Vladimir Acedo Lazo, Omar Alberto Cáceres Rey, Henri Bailón Calderón, Priscila Nayu Lope Pari, Nancy Rojas Serrano, Gloria Arotinco Garayar. Equipo de Vigilancia Genomica del Instituto Nacional de Salud.
EPI_ISL_17008293, EPI_ISL_17008294, EPI_ISL_17008295, EPI_ISL_17008296	Tokyo Metropolitan Institute of Public Health, Department of Microbiology	Tokyo Metropolitan Institute of Public Health, Department of Microbiology	Kasuya,F., Negishi,A., Kumagai,R., Hasegawa,M., Fujiwara,T., Miyake,H., Nagashima,M. and Sadamasu,K.
EPI_ISL_17008374, EPI_ISL_17008375, EPI_ISL_17008376, EPI_ISL_17008377, EPI_ISL_17008378, EPI_ISL_17008379, EPI_ISL_17008380, EPI_ISL_17008381, EPI_ISL_17008382, EPI_ISL_17008383, EPI_ISL_17008384			
see above	Centre for Biological Threats, Highly Pathogenic Viruses, Robert Koch Institute	Centre for Biological Threats, Highly Pathogenic Viruses, Robert Koch Institute	Brinkmann,A., Kohl,C., Pape,K., Schrick,L., Michel,J., Schaade,L. and Nitsche,A.
EPI_ISL_17012023, EPI_ISL_17012024, EPI_ISL_17012025, EPI_ISL_17012026, EPI_ISL_17012027, EPI_ISL_17012028, EPI_ISL_17012029, EPI_ISL_17012031, EPI_ISL_17012032, EPI_ISL_17012033, EPI_ISL_17012036, EPI_ISL_17012037, EPI_ISL_17012038, EPI_ISL_17012039, EPI_ISL_17012040, EPI_ISL_17012041, EPI_ISL_17012042, EPI_ISL_17012043, EPI_ISL_17012044, EPI_ISL_17012047, EPI_ISL_17012049, EPI_ISL_17012050, EPI_ISL_17012051, EPI_ISL_17012052, EPI_ISL_17012053, EPI_ISL_17012054, EPI_ISL_17012055, EPI_ISL_17012056, EPI_ISL_17012057, EPI_ISL_17012059, EPI_ISL_17012060, EPI_ISL_17012061, EPI_ISL_17012062, EPI_ISL_17012063, EPI_ISL_17012064, EPI_ISL_17012067, EPI_ISL_17012068, EPI_ISL_17012069, EPI_ISL_17012071			
see above	Laboratorio de Virus Exantematicos, Gastroentéricos y Otros Transmitidos por Vectores	Centro de Referencia Nacional de Genomica, Secuenciacion y Bioinformatica GENSBIO, INSPI-CZ9	Andrés Carrazco, Silvia Salgado, Diana Gutiérrez, Damaris Alarcón, Andrés Herrera, Andrés Tinizaray, Martha Sánchez, Johanna Parrales, Diego Morales, Jorge Bejarano, Leandro Patiño.
EPI_ISL_17012073	Laboratorio de Virus Exantematicos, Gastroentéricos y Otros Transmitidos por Vectores	Centro de Referencia Nacional de Genomica, Secuenciacion y Bioinformatica GENSBIO, INSPI-CZ12	Andrés Carrazco, Silvia Salgado, Diana Gutiérrez, Damaris Alarcón, Andrés Herrera, Andrés Tinizaray, Martha Sánchez, Johanna Parrales, Diego Morales, Jorge Bejarano, Leandro Patiño.
EPI_ISL_17012074	Laboratorio de Virus Exantematicos, Gastroentéricos y Otros Transmitidos por Vectores	Centro de Referencia Nacional de Genomica, Secuenciacion y Bioinformatica GENSBIO, INSPI-CZ14	Andrés Carrazco, Silvia Salgado, Diana Gutiérrez, Damaris Alarcón, Andrés Herrera, Andrés Tinizaray, Martha Sánchez, Johanna Parrales, Diego Morales, Jorge Bejarano, Leandro Patiño.
EPI_ISL_17012075	Laboratorio de Virus Exantematicos, Gastroentéricos y Otros Transmitidos por Vectores	Centro de Referencia Nacional de Genomica, Secuenciacion y Bioinformatica GENSBIO, INSPI-CZ15	Andrés Carrazco, Silvia Salgado, Diana Gutiérrez, Damaris Alarcón, Andrés Herrera, Andrés Tinizaray, Martha Sánchez, Johanna Parrales, Diego Morales, Jorge Bejarano, Leandro Patiño.
EPI_ISL_17012076	Laboratorio de Virus Exantematicos, Gastroentéricos y Otros Transmitidos por Vectores	Centro de Referencia Nacional de Genomica, Secuenciacion y Bioinformatica GENSBIO, INSPI-CZ16	Andrés Carrazco, Silvia Salgado, Diana Gutiérrez, Damaris Alarcón, Andrés Herrera, Andrés Tinizaray, Martha Sánchez, Johanna Parrales, Diego Morales, Jorge Bejarano, Leandro Patiño.
EPI_ISL_17012077	Laboratorio de Virus Exantematicos, Gastroentéricos y Otros Transmitidos por Vectores	Centro de Referencia Nacional de Genomica, Secuenciacion y Bioinformatica GENSBIO, INSPI-CZ18	Andrés Carrazco, Silvia Salgado, Diana Gutiérrez, Damaris Alarcón, Andrés Herrera, Andrés Tinizaray, Martha Sánchez, Johanna Parrales, Diego Morales, Jorge Bejarano, Leandro Patiño.
EPI_ISL_17012079, EPI_ISL_17012080, EPI_ISL_17012082, EPI_ISL_17012084, EPI_ISL_17012088, EPI_ISL_17012089, EPI_ISL_17012090, EPI_ISL_17012091, EPI_ISL_17012092, EPI_ISL_17012094, EPI_ISL_17012096, EPI_ISL_17012097, EPI_ISL_17012100, EPI_ISL_17012101, EPI_ISL_17012102, EPI_ISL_17012104, EPI_ISL_17012107, EPI_ISL_17012108, EPI_ISL_17012109, EPI_ISL_17012110, EPI_ISL_17012111, EPI_ISL_17012115			
see above	Laboratorio de Virus Exantematicos, Gastroentéricos y Otros Transmitidos por Vectores	Centro de Referencia Nacional de Genomica, Secuenciacion y Bioinformatica GENSBIO, INSPI-CZ9	Andrés Carrazco-Motalvo, Silvia Salgado, Diana Gutiérrez, Damaris Alarcón, Andrés Herrera, Andrés Tinizaray, Ruth Gómez, Martha Sánchez, Johanna Parrales, Diego Morales, Jorge Bejarano, Leandro Patiño.
EPI_ISL_17018429, EPI_ISL_17018430, EPI_ISL_17018431, EPI_ISL_17018433, EPI_ISL_17018434, EPI_ISL_17018435, EPI_ISL_17018436, EPI_ISL_17018437, EPI_ISL_17018438, EPI_ISL_17018439	National Institute for Infectious Diseases "Matei Bals"	National Institute for Infectious Diseases "Matei Bals"	Robert Hohan, Marius Surleac, Leontina Banica, Andreea Tudor, Simona Paraschiv
EPI_ISL_17019459, EPI_ISL_17019461, EPI_ISL_17019462, EPI_ISL_17019464, EPI_ISL_17019467	Parkland Health and Hospital System	Dallas County Health & Human Services Public Health Laboratory	Kabir, Farruk; Plaisance, Erin; Stringer, Joey; Short, Luke.
EPI_ISL_17019470	MD Progressive Care	Dallas County Health & Human Services Public Health Laboratory	Kabir, Farruk; Plaisance, Erin; Stringer, Joey; Short, Luke.
EPI_ISL_17019472	White Rock Medical Center	Dallas County Health & Human Services Public Health Laboratory	Kabir, Farruk; Plaisance, Erin; Stringer, Joey; Short, Luke.
EPI_ISL_17019473	Children's Health Dallas Texas	Dallas County Health & Human Services Public Health Laboratory	Kabir, Farruk; Plaisance, Erin; Stringer, Joey; Short, Luke.
EPI_ISL_17019476	Dallas County Jail	Dallas County Health & Human Services Public Health Laboratory	Kabir, Farruk; Plaisance, Erin; Stringer, Joey; Short, Luke.
EPI_ISL_17048204, EPI_ISL_17048205, EPI_ISL_17048206, EPI_ISL_17048207	Laboratorio Central de Saude Publica do Estado da Bahia (LACEN/BA)	Laboratory of Respiratory Viruses and Measles, Oswaldo Cruz Institute, FIOCRUZ	Paola Resende, Fernando Motta, Elisa Cavalcante Pereira, Bruna Mendonça da Silva, Jéssica Graça Macedo de Carvalho, Larissa Macedo Pinto, Victor Guimaraes, Felicidade Pereira, Marilda Siqueira, Renan da Silva Faustino, Marilia Santini, Edson Elias da Silva on behalf of the Fiocruz COVID-19 Genomic Surveillance Network
EPI_ISL_17048208	Laboratorio de Enterovirus, Instituto Oswaldo Cruz, Fiocruz	Laboratory of Respiratory Viruses and Measles, Oswaldo Cruz Institute, FIOCRUZ	Paola Resende, Elisa Cavalcante Pereira, Bruna Mendonça da Silva, Jéssica Graça Macedo de Carvalho, Larissa Macedo Pinto, Victor Guimaraes, Marilda Siqueira, Renan da Silva Faustino, Marilia Santini, Edson Elias da Silva on behalf of the Fiocruz Genomic Surveillance Network
EPI_ISL_17085647, EPI_ISL_17085649, EPI_ISL_17085650, EPI_ISL_17085652, EPI_ISL_17085653, EPI_ISL_17085655, EPI_ISL_17085656, EPI_ISL_17085658, EPI_ISL_17085659, EPI_ISL_17085661, EPI_ISL_17085662, EPI_ISL_17085664, EPI_ISL_17085665, EPI_ISL_17085667, EPI_ISL_17085668, EPI_ISL_17085670, EPI_ISL_17085671, EPI_ISL_17085673, EPI_ISL_17085674, EPI_ISL_17085676, EPI_ISL_17085678, EPI_ISL_17085681, EPI_ISL_17085682, EPI_ISL_17085684, EPI_ISL_17085685, EPI_ISL_17085687, EPI_ISL_17085688, EPI_ISL_17085690, EPI_ISL_17085692, EPI_ISL_17085693, EPI_ISL_17085695, EPI_ISL_17085696, EPI_ISL_17085698, EPI_ISL_17085700, EPI_ISL_17085701, EPI_ISL_17085703, EPI_ISL_17085704, EPI_ISL_17085706, EPI_ISL_17085708, EPI_ISL_17085709, EPI_ISL_17085712			
see above	Public Health Laboratory, NYC Department of Health and Mental Hygiene	Public Health Laboratory, NYC Department of Health and Mental Hygiene	Wang,J.C., Amin,H.S., Clabby,T.T., Taki,F., Su,M., Rahat,A., De La Cruz,N., Olsen,A., Thi,C., Silver,S., Akther,S., Chowdhury,M., Omoregie,E. and Hughes,S.
EPI_ISL_17085714, EPI_ISL_17085715, EPI_ISL_17085717, EPI_ISL_17085719, EPI_ISL_17085720, EPI_ISL_17085722, EPI_ISL_17085723, EPI_ISL_17085725, EPI_ISL_17085726, EPI_ISL_17085728, EPI_ISL_17085730, EPI_ISL_17085733, EPI_ISL_17085734, EPI_ISL_17085736, EPI_ISL_17085737, EPI_ISL_17085739, EPI_ISL_17085740, EPI_ISL_17085742, EPI_ISL_17085743, EPI_ISL_17085745, EPI_ISL_17085746, EPI_ISL_17085748, EPI_ISL_17085750, EPI_ISL_17085752, EPI_ISL_17085753, EPI_ISL_17085755, EPI_ISL_17085756, EPI_ISL_17085758, EPI_ISL_17085760, EPI_ISL_17085761, EPI_ISL_17085762, EPI_ISL_17085764, EPI_ISL_17085765, EPI_ISL_17085767, EPI_ISL_17085769, EPI_ISL_17085770, EPI_ISL_17085771, EPI_ISL_17085773, EPI_ISL_17085774, EPI_ISL_17085776, EPI_ISL_17085777, EPI_ISL_17085779, EPI_ISL_17085781, EPI_ISL_17085782, EPI_ISL_17085784, EPI_ISL_17085785, EPI_ISL_17085787, EPI_ISL_17085788, EPI_ISL_17085790, EPI_ISL_17085792, EPI_ISL_17085793, EPI_ISL_17085794, EPI_ISL_17085795, EPI_ISL_17085796, EPI_ISL_17085797, EPI_ISL_17085798, EPI_ISL_17085920, EPI_ISL_17085921, EPI_ISL_17085922, EPI_ISL_17085923, EPI_ISL_17085924, EPI_ISL_17085925, EPI_ISL_17085926, EPI_ISL_17085927, EPI_ISL_17085928, EPI_ISL_17085929, EPI_ISL_17085930, EPI_ISL_17085931, EPI_ISL_17085932, EPI_ISL_17085933, EPI_ISL_17085934, EPI_ISL_17085935, EPI_ISL_17085936, EPI_ISL_17085937, EPI_ISL_17085938, EPI_ISL_17085939, EPI_ISL_17085940, EPI_ISL_17085941, EPI_ISL_17085942, EPI_ISL_17085943, EPI_ISL_17085944, EPI_ISL_17085945, EPI_ISL_17085946, EPI_ISL_17085947, EPI_ISL_17085948, EPI_ISL_17085949, EPI_ISL_17085950, EPI_ISL_17085951, EPI_ISL_17085952, EPI_ISL_17085953, EPI_ISL_17085954, EPI_ISL_17085955, EPI_ISL_17085956, EPI_ISL_17085957, EPI_ISL_17085958, EPI_ISL_17085959, EPI_ISL_17085960, EPI_ISL_17085961			
see above	Public Health Laboratory, NYC Department of Health and Mental Hygiene	Public Health Laboratory, NYC Department of Health and Mental Hygiene	Clabby,T.T., Amin,H.S., Wang,J.C., Taki,F., Su,M., Rahat,A., De La Cruz,N., Olsen,A., Thi,C., Silver,S., Akther,S., Chowdhury,M., Omoregie,E. and Hughes,S.
EPI_ISL_17085962, EPI_ISL_17085963, EPI_ISL_17085964, EPI_ISL_17085965, EPI_ISL_17085966, EPI_ISL_17085967, EPI_ISL_17085968, EPI_ISL_17085969, EPI_ISL_17085970, EPI_ISL_17085971, EPI_ISL_17085972, EPI_ISL_17085973, EPI_ISL_17085974, EPI_ISL_17085975, EPI_ISL_17085976, EPI_ISL_17085977, EPI_ISL_17085978, EPI_ISL_17085979, EPI_ISL_17085980, EPI_ISL_17085981, EPI_ISL_17085983, EPI_ISL_17085984, EPI_ISL_17085985, EPI_ISL_17085986, EPI_ISL_17085987, EPI_ISL_17085988, EPI_ISL_17085989, EPI_ISL_17085990, EPI_ISL_17085991, EPI_ISL_17085992, EPI_ISL_17085993, EPI_ISL_17085994, EPI_ISL_17085995, EPI_ISL_17085996, EPI_ISL_17085997, EPI_ISL_17085998, EPI_ISL_17085999, EPI_ISL_17086000, EPI_ISL_17086001, EPI_ISL_17086002, EPI_ISL_17086003, EPI_ISL_17086004, EPI_ISL_17086005, EPI_ISL_17086006, EPI_ISL_17086007, EPI_ISL_17086008, EPI_ISL_17086009, EPI_ISL_17086010, EPI_ISL_17086011, EPI_ISL_17086012, EPI_ISL_17086013, EPI_ISL_17086014, EPI_ISL_17086015, EPI_ISL_17086016, EPI_ISL_17086017, EPI_ISL_17086018, EPI_ISL_17086019, EPI_ISL_17086020, EPI_ISL_17086021, EPI_ISL_17086022, EPI_ISL_17086023, EPI_ISL_17086024, EPI_ISL_17086025, EPI_ISL_17086026, EPI_ISL_17086027, EPI_ISL_17086028, EPI_ISL_17086029, EPI_ISL_17086030, EPI_ISL_17086031, EPI_ISL_17086032, EPI_ISL_17086033, EPI_ISL_17086034, EPI_ISL_17086035, EPI_ISL_17086036, EPI_ISL_17086037, EPI_ISL_17086038, EPI_ISL_17086039, EPI_ISL_17086040, EPI_ISL_17086042, EPI_ISL_17086043, EPI_ISL_17086044, EPI_ISL_17086045, EPI_ISL_17086046, EPI_ISL_17086048, EPI_ISL_17086049, EPI_ISL_17086050, EPI_ISL_17086051, EPI_ISL_17086052, EPI_ISL_17086053, EPI_ISL_17086054, EPI_ISL_17086055, EPI_ISL_17086056, EPI_ISL_17086057, EPI_ISL_17086058, EPI_ISL_17086059, EPI_ISL_17086060, EPI_ISL_17086061			
see above	Public Health Laboratory, NYC Department of Health and Mental Hygiene	Public Health Laboratory, NYC Department of Health and Mental Hygiene	Amin,H.S., Clabby,T.T., Wang,J.C., Taki,F., Su,M., Rahat,A., De La Cruz,N., Olsen,A., Thi,C., Silver,S., Akther,S., Chowdhury,M., Omoregie,E. and Hughes,S.
EPI_ISL_17086062, EPI_ISL_17086063, EPI_ISL_17086064, EPI_ISL_17086065, EPI_ISL_17086066, EPI_ISL_17086067, EPI_ISL_17086068, EPI_ISL_17086069, EPI_ISL_17086070, EPI_ISL_17086071, EPI_ISL_17086072, EPI_ISL_17086073, EPI_ISL_17086074, EPI_ISL_17086075, EPI_ISL_17086076, EPI_ISL_17086077, EPI_ISL_17086078, EPI_ISL_17086079, EPI_ISL_17086080, EPI_ISL_17086081, EPI_ISL_17086082, EPI_ISL_17086083, EPI_ISL_17086084, EPI_ISL_17086086, EPI_ISL_17086087, EPI_ISL_17086088, EPI_ISL_17086089, EPI_ISL_17086090, EPI_ISL_17086091, EPI_ISL_17086093, EPI_ISL_17086095, EPI_ISL_17086096, EPI_ISL_17086097, EPI_ISL_17086098, EPI_ISL_17086101, EPI_ISL_17086102, EPI_ISL_17086103, EPI_ISL_17086104, EPI_ISL_17086105, EPI_ISL_17086106, EPI_ISL_17086107, EPI_ISL_17086108, EPI_ISL_17086109, EPI_ISL_17086110, EPI_ISL_17086111, EPI_ISL_17086112, EPI_ISL_17086113, EPI_ISL_17086114, EPI_ISL_17086115, EPI_ISL_17086116, EPI_ISL_17086117, EPI_ISL_17086118, EPI_ISL_17086119, EPI_ISL_17086120, EPI_ISL_17086121, EPI_ISL_17086122, EPI_ISL_17086123, EPI_ISL_17086124, EPI_ISL_17086125, EPI_ISL_17086126, EPI_ISL_17086127, EPI_ISL_17086128, EPI_ISL_17086129, EPI_ISL_17086130, EPI_ISL_17086131, EPI_ISL_17086132, EPI_ISL_17086133, EPI_ISL_17086134, EPI_ISL_17086135, EPI_ISL_17086136, EPI_ISL_17086137, EPI_ISL_17086138, EPI_ISL_17086139, EPI_ISL_17086140, EPI_ISL_17086141, EPI_ISL_17086142, EPI_ISL_17086143, EPI_ISL_17086144, EPI_ISL_17086145, EPI_ISL_17086146, EPI_ISL_17086147, EPI_ISL_17086148, EPI_ISL_17086149, EPI_ISL_17086150, EPI_ISL_17086151, EPI_ISL_17086152, EPI_ISL_17086153, EPI_ISL_17086154, EPI_ISL_17086155, EPI_ISL_17086156, EPI_ISL_17086157, EPI_ISL_17086158, EPI_ISL_17086159, EPI_ISL_17086160, EPI_ISL_17086161, EPI_ISL_17086162, EPI_ISL_17086163, EPI_ISL_17086164, EPI_ISL_17086165, EPI_ISL_17086166, EPI_ISL_17086167, EPI_ISL_17086168, EPI_ISL_17086169, EPI_ISL_17086170, EPI_ISL_17086171, EPI_ISL_17086172, EPI_ISL_17086173, EPI_ISL_17086174, EPI_ISL_17086175, EPI_ISL_17086176, EPI_ISL_17086177, EPI_ISL_17086178, EPI_ISL_17086179, EPI_ISL_17086180, EPI_ISL_17086181, EPI_ISL_17086182, EPI_ISL_17086183, EPI_ISL_17086184, EPI_ISL_17086185, EPI_ISL_17086186, EPI_ISL_17086187, EPI_ISL_17086188, EPI_ISL_17086189, EPI_ISL_17086190, EPI_ISL_17086191, EPI_ISL_17086192, EPI_ISL_17086193, EPI_ISL_17086194, EPI_ISL_17086195, EPI_ISL_17086196, EPI_ISL_17086197, EPI_ISL_17086198, EPI_ISL_17086199, EPI_ISL_17086200, EPI_ISL_17086201, EPI_ISL_17086202, EPI_ISL_17086203, EPI_ISL_17086204, EPI_ISL_17086205, EPI_ISL_17086206, EPI_ISL_17086207, EPI_ISL_17086208, EPI_ISL_17086209, EPI_ISL_17086210, EPI_ISL_17086211, EPI_ISL_17086212, EPI_ISL_17086213, EPI_ISL_17086214, EPI_ISL_17086215, EPI_ISL_17086216, EPI_ISL_17086217, EPI_ISL_17086218, EPI_ISL_17086219, EPI_ISL_17086232, EPI_ISL_17086233, EPI_ISL_17086234, EPI_ISL_17086235, EPI_ISL_17086236, EPI_ISL_17086237, EPI_ISL_17086238, EPI_ISL_17086239, EPI_ISL_17086240, EPI_ISL_17086241, EPI_ISL_17086242, EPI_ISL_17086243, EPI_ISL_17086244, EPI_ISL_17086245, EPI_ISL_17086246, EPI_ISL_17086247, EPI_ISL_17086248, EPI_ISL_17086249, EPI_ISL_17086250, EPI_ISL_17086251, EPI_ISL_17086252, EPI_ISL_17086253, EPI_ISL_17086254, EPI_ISL_17086255, EPI_ISL_17086256, EPI_ISL_17086257, EPI_ISL_17086258, EPI_ISL_17086259, EPI_ISL_17086260, EPI_ISL_17086261, EPI_ISL_17086262, EPI_ISL_17086263, EPI_ISL_17086264, EPI_ISL_17086265, EPI_ISL_17086266, EPI_ISL_17086267, EPI_ISL_17086268, EPI_ISL_17086269, EPI_ISL_17086270, EPI_ISL_17086271, EPI_ISL_17086272			
see above	Public Health Laboratory, NYC Department of Health and Mental Hygiene	Public Health Laboratory, NYC Department of Health and Mental Hygiene	Wang,J.C., Amin,H.S., Clabby,T.T., Taki,F., Su,M., Rahat,A., De La Cruz,N., Olsen,A., Thi,C., Silver,S., Akther,S., Chowdhury,M., Omoregie,E. and Hughes,S.
EPI_ISL_17086274, EPI_ISL_17086275, EPI_ISL_17086276, EPI_ISL_17086277, EPI_ISL_17086278, EPI_ISL_17086279, EPI_ISL_17086280, EPI_ISL_17086281, EPI_ISL_17086282, EPI_ISL_17086283, EPI_ISL_17086284, EPI_ISL_17086285, EPI_ISL_17086286, EPI_ISL_17086287, EPI_ISL_17086288, EPI_ISL_17086289, EPI_ISL_17086290, EPI_ISL_17086291, EPI_ISL_17086292, EPI_ISL_17086293, EPI_ISL_17086294, EPI_ISL_17086295, EPI_ISL_17086296, EPI_ISL_17086297, EPI_ISL_17086298, EPI_ISL_17086299, EPI_ISL_17086300, EPI_ISL_17086301, EPI_ISL_17086302, EPI_ISL_17086304, EPI_ISL_17086305, EPI_ISL_17086306, EPI_ISL_17086307, EPI_ISL_17086308, EPI_ISL_17086309, EPI_ISL_17086310, EPI_ISL_17086311, EPI_ISL_17086312, EPI_ISL_17086313, EPI_ISL_17086314, EPI_ISL_17086315, EPI_ISL_17086316, EPI_ISL_17086317, EPI_ISL_17086318, EPI_ISL_17086319, EPI_ISL_17086320, EPI_ISL_17086321, EPI_ISL_17086322, EPI_ISL_17086323, EPI_ISL_17086324, EPI_ISL_17086325, EPI_ISL_17086326, EPI_ISL_17086327, EPI_ISL_17086328, EPI_ISL_17086329, EPI_ISL_17086330, EPI_ISL_17086331, EPI_ISL_17086332, EPI_ISL_17104181, EPI_ISL_17104182, EPI_ISL_17104183, EPI_ISL_17104184, EPI_ISL_17104185, EPI_ISL_17104186, EPI_ISL_17104188, EPI_ISL_17104189, EPI_ISL_17104190, EPI_ISL_17104191, EPI_ISL_17104192, EPI_ISL_17104193, EPI_ISL_17104194, EPI_ISL_17104195, EPI_ISL_17104196, EPI_ISL_17104197, EPI_ISL_17104198, EPI_ISL_17104200, EPI_ISL_17104201, EPI_ISL_17104203, EPI_ISL_17104204, EPI_ISL_17104205, EPI_ISL_17104206, EPI_ISL_17104207, EPI_ISL_17104208, EPI_ISL_17104209, EPI_ISL_17104210, EPI_ISL_17104212, EPI_ISL_17104213, EPI_ISL_17104214, EPI_ISL_17104215, EPI_ISL_17104216, EPI_ISL_17104217, EPI_ISL_17104218, EPI_ISL_17104219, EPI_ISL_17104220, EPI_ISL_17104221, EPI_ISL_17104222, EPI_ISL_17104224, EPI_ISL_17104225, EPI_ISL_17104226, EPI_ISL_17104227, EPI_ISL_17104228, EPI_ISL_17104229, EPI_ISL_17104230, EPI_ISL_17104231, EPI_ISL_17104232, EPI_ISL_17104233, EPI_ISL_17104236, EPI_ISL_17104237, EPI_ISL_17104238, EPI_ISL_17104239, EPI_ISL_17104240, EPI_ISL_17104241, EPI_ISL_17104242, EPI_ISL_17104243, EPI_ISL_17104244, EPI_ISL_17104245, EPI_ISL_17104247, EPI_ISL_17104248, EPI_ISL_17104249, EPI_ISL_17104250, EPI_ISL_17104251, EPI_ISL_17104252, EPI_ISL_17104253, EPI_ISL_17104254, EPI_ISL_17104255, EPI_ISL_17104256, EPI_ISL_17104257, EPI_ISL_17104259, EPI_ISL_17104260, EPI_ISL_17104261, EPI_ISL_17104262, EPI_ISL_17104263, EPI_ISL_17104264, EPI_ISL_17104265, EPI_ISL_17104266, EPI_ISL_17104267, EPI_ISL_17104268, EPI_ISL_17104269, EPI_ISL_17104271, EPI_ISL_17104272, EPI_ISL_17104273, EPI_ISL_17104274, EPI_ISL_17104275, EPI_ISL_17104276, EPI_ISL_17104277, EPI_ISL_17104278, EPI_ISL_17104279, EPI_ISL_17104280, EPI_ISL_17104281, EPI_ISL_17104282, EPI_ISL_17104284, EPI_ISL_17104285, EPI_ISL_17104286, EPI_ISL_17104287, EPI_ISL_17104288, EPI_ISL_17104350, EPI_ISL_17104352, EPI_ISL_17104353, EPI_ISL_17104354, EPI_ISL_17104355, EPI_ISL_17104356, EPI_ISL_17104357, EPI_ISL_17104358, EPI_ISL_17104359, EPI_ISL_17104360, EPI_ISL_17104361, EPI_ISL_17104362, EPI_ISL_17104364, EPI_ISL_17104365, EPI_ISL_17104366, EPI_ISL_17104368, EPI_ISL_17104369, EPI_ISL_17104370, EPI_ISL_17104371, EPI_ISL_17104372, EPI_ISL_17104373, EPI_ISL_17104374, EPI_ISL_17104375, EPI_ISL_17104377, EPI_ISL_17104378, EPI_ISL_17104379, EPI_ISL_17104380, EPI_ISL_17104381, EPI_ISL_17104382, EPI_ISL_17104383, EPI_ISL_17104384, EPI_ISL_17104385, EPI_ISL_17104386, EPI_ISL_17104387, EPI_ISL_17104389, EPI_ISL_17104390, EPI_ISL_17104391, EPI_ISL_17104392, EPI_ISL_17104393, EPI_ISL_17104394, EPI_ISL_17104395, EPI_ISL_17104396, EPI_ISL_17104397, EPI_ISL_17104398, EPI_ISL_17104399, EPI_ISL_17104401, EPI_ISL_17104402, EPI_ISL_17104403, EPI_ISL_17104404, EPI_ISL_17104405, EPI_ISL_17104406, EPI_ISL_17104407, EPI_ISL_17104409, EPI_ISL_17104410, EPI_ISL_17104412, EPI_ISL_17104413, EPI_ISL_17104414, EPI_ISL_17104415, EPI_ISL_17104416, EPI_ISL_17104417, EPI_ISL_17104418, EPI_ISL_17104419, EPI_ISL_17104421, EPI_ISL_17104422, EPI_ISL_17104423, EPI_ISL_17104425, EPI_ISL_17104426, EPI_ISL_17104427, EPI_ISL_17104428, EPI_ISL_17104429, EPI_ISL_17104430, EPI_ISL_17104431, EPI_ISL_17104432, EPI_ISL_17104433, EPI_ISL_17104434, EPI_ISL_17104435, EPI_ISL_17104437, EPI_ISL_17104438, EPI_ISL_17104439, EPI_ISL_17104440, EPI_ISL_17104441, EPI_ISL_17104442, EPI_ISL_17104443, EPI_ISL_17104444, EPI_ISL_17104445, EPI_ISL_17104446, EPI_ISL_17104447, EPI_ISL_17104448, EPI_ISL_17104450, EPI_ISL_17104451, EPI_ISL_17104452, EPI_ISL_17104453, EPI_ISL_17104454, EPI_ISL_17104455, EPI_ISL_17104456, EPI_ISL_17104457, EPI_ISL_17104458, EPI_ISL_17104490, EPI_ISL_17104491, EPI_ISL_17104492, EPI_ISL_17104493, EPI_ISL_17104494, EPI_ISL_17104496, EPI_ISL_17104497, EPI_ISL_17104498, EPI_ISL_17104499, EPI_ISL_17104500, EPI_ISL_17104501, EPI_ISL_17104502, EPI_ISL_17104503, EPI_ISL_17104504, EPI_ISL_17104506, EPI_ISL_17104507, EPI_ISL_17104508, EPI_ISL_17104509, EPI_ISL_17104510, EPI_ISL_17104511, EPI_ISL_17104512, EPI_ISL_17104513, EPI_ISL_17104514, EPI_ISL_17104515, EPI_ISL_17104516, EPI_ISL_17104517, EPI_ISL_17104518, EPI_ISL_17104521, EPI_ISL_17104522, EPI_ISL_17104523, EPI_ISL_17104524, EPI_ISL_17104525, EPI_ISL_17104526, EPI_ISL_17104527, EPI_ISL_17104528, EPI_ISL_17104529, EPI_ISL_17104530, EPI_ISL_17104531, EPI_ISL_17104533, EPI_ISL_17104534, EPI_ISL_17104535, EPI_ISL_17104536, EPI_ISL_17104537, EPI_ISL_17104538, EPI_ISL_17104539, EPI_ISL_17104540, EPI_ISL_17104541, EPI_ISL_17104542, EPI_ISL_17104543, EPI_ISL_17104545, EPI_ISL_17104546, EPI_ISL_17104547, EPI_ISL_17104548, EPI_ISL_17104549, EPI_ISL_17104551, EPI_ISL_17104552, EPI_ISL_17104553, EPI_ISL_17104554, EPI_ISL_17104555, EPI_ISL_17104556, EPI_ISL_17104557, EPI_ISL_17104559, EPI_ISL_17104560, EPI_ISL_17104561, EPI_ISL_17104562, EPI_ISL_17104563, EPI_ISL_17104564, EPI_ISL_17104565, EPI_ISL_17104566, EPI_ISL_17104567, EPI_ISL_17104568, EPI_ISL_17104569, EPI_ISL_17104570, EPI_ISL_17104572, EPI_ISL_17104573, EPI_ISL_17104574, EPI_ISL_17104575, EPI_ISL_17104576, EPI_ISL_17104577, EPI_ISL_17104578, EPI_ISL_17104579, EPI_ISL_17104580, EPI_ISL_17104581, EPI_ISL_17104582, EPI_ISL_17104583, EPI_ISL_17104585, EPI_ISL_17104586, EPI_ISL_17104587, EPI_ISL_17104588, EPI_ISL_17104589, EPI_ISL_17104590, EPI_ISL_17104591, EPI_ISL_17104592, EPI_ISL_17104593, EPI_ISL_17104594, EPI_ISL_17104595, EPI_ISL_17104597, EPI_ISL_17104598, EPI_ISL_17104599, EPI_ISL_17104613, EPI_ISL_17104614, EPI_ISL_17104615, EPI_ISL_17104616, EPI_ISL_17104617, EPI_ISL_17104618, EPI_ISL_17104619, EPI_ISL_17104620, EPI_ISL_17104621, EPI_ISL_17104622, EPI_ISL_17104623, EPI_ISL_17104625, EPI_ISL_17104626, EPI_ISL_17104627, EPI_ISL_17104628, EPI_ISL_17104629, EPI_ISL_17104630, EPI_ISL_17104631, EPI_ISL_17104632, EPI_ISL_17104633, EPI_ISL_17104634, EPI_ISL_17104635, EPI_ISL_17104637, EPI_ISL_17104638, EPI_ISL_17104639, EPI_ISL_17104641, EPI_ISL_17104642, EPI_ISL_17104643, EPI_ISL_17104644, EPI_ISL_17104645, EPI_ISL_17104646, EPI_ISL_17104647, EPI_ISL_17104648, EPI_ISL_17104649, EPI_ISL_17104650, EPI_ISL_17104651, EPI_ISL_17104652, EPI_ISL_17104653, EPI_ISL_17104655, EPI_ISL_17104656, EPI_ISL_17104657			
see above	Public Health Laboratory, NYC Department of Health and Mental Hygiene	Public Health Laboratory, NYC Department of Health and Mental Hygiene	Clabby,T.T., Amin,H.S., Wang,J.C., Taki,F., Su,M., Rahat,A., De La Cruz,N., Olsen,A., Thi,C., Silver,S., Akther,S., Chowdhury,M., Omoregie,E. and Hughes,S.

EPI_ISL_17118598, EPI_ISL_17118599, EPI_ISL_17118601, EPI_ISL_17118602, EPI_ISL_17118603, EPI_ISL_17118604, EPI_ISL_17118605, EPI_ISL_17118606, EPI_ISL_17118607, EPI_ISL_17118608, EPI_ISL_17118609, EPI_ISL_17118610, EPI_ISL_17118611, EPI_ISL_17118612, EPI_ISL_17118613, EPI_ISL_17118614, EPI_ISL_17118615, EPI_ISL_17118616, EPI_ISL_17118617, EPI_ISL_17118618, EPI_ISL_17118619, EPI_ISL_17118620, EPI_ISL_17118621, EPI_ISL_17118622, EPI_ISL_17118623, EPI_ISL_17118624, EPI_ISL_17118625, EPI_ISL_17118626, EPI_ISL_17118627, EPI_ISL_17118628, EPI_ISL_17118629, EPI_ISL_17118630, EPI_ISL_17118631, EPI_ISL_17118632, EPI_ISL_17118633, EPI_ISL_17118634, EPI_ISL_17118635, EPI_ISL_17118636, EPI_ISL_17118637, EPI_ISL_17118638, EPI_ISL_17118639, EPI_ISL_17118640, EPI_ISL_17118641, EPI_ISL_17118642, EPI_ISL_17118643, EPI_ISL_17118644, EPI_ISL_17118645, EPI_ISL_17118646, EPI_ISL_17118647, EPI_ISL_17118648, EPI_ISL_17118649, EPI_ISL_17118650, EPI_ISL_17118651, EPI_ISL_17118652, EPI_ISL_17118653, EPI_ISL_17118654, EPI_ISL_17118655, EPI_ISL_17118656, EPI_ISL_17118657, EPI_ISL_17118659, EPI_ISL_17118660, EPI_ISL_17118661, EPI_ISL_17118662, EPI_ISL_17118663, EPI_ISL_17118664, EPI_ISL_17118665, EPI_ISL_17118666, EPI_ISL_17118667, EPI_ISL_17118668, EPI_ISL_17118669, EPI_ISL_17118670, EPI_ISL_17118671, EPI_ISL_17118672, EPI_ISL_17118673, EPI_ISL_17118674, EPI_ISL_17118675, EPI_ISL_17118676, EPI_ISL_17118677, EPI_ISL_17118678, EPI_ISL_17118679, EPI_ISL_17118680, EPI_ISL_17118681, EPI_ISL_17118682, EPI_ISL_17118683, EPI_ISL_17118684, EPI_ISL_17118685, EPI_ISL_17118686, EPI_ISL_17118687, EPI_ISL_17118688, EPI_ISL_17118689, EPI_ISL_17118690, EPI_ISL_17118691, EPI_ISL_17118692, EPI_ISL_17118693, EPI_ISL_17118694, EPI_ISL_17118695, EPI_ISL_17118696, EPI_ISL_17118697			
see above	Public Health Laboratory, NYC Department of Health and Mental Hygiene	Public Health Laboratory, NYC Department of Health and Mental Hygiene	Wang,J.C., Amin,H.S., Clabby,T.T., Taki,F., Su,M., Rahat,A., De La Cruz,N., Olsen,A., Thi,C., Silver,S., Akther,S., Chowdhury,M., Omoregie,E. and Hughes,S.
EPI_ISL_17118698, EPI_ISL_17118699, EPI_ISL_17118700, EPI_ISL_17118701, EPI_ISL_17118702, EPI_ISL_17118703, EPI_ISL_17118704, EPI_ISL_17118705, EPI_ISL_17118706, EPI_ISL_17118707, EPI_ISL_17118708, EPI_ISL_17118709, EPI_ISL_17118710, EPI_ISL_17118711, EPI_ISL_17118712, EPI_ISL_17118713, EPI_ISL_17118714, EPI_ISL_17118715, EPI_ISL_17118716, EPI_ISL_17118717, EPI_ISL_17118718, EPI_ISL_17118719, EPI_ISL_17118720, EPI_ISL_17118721, EPI_ISL_17118722, EPI_ISL_17118723, EPI_ISL_17118724, EPI_ISL_17118725, EPI_ISL_17118726, EPI_ISL_17118727, EPI_ISL_17118728, EPI_ISL_17118729, EPI_ISL_17118730, EPI_ISL_17118731, EPI_ISL_17118732, EPI_ISL_17118733			
see above	Laboratory Medicine, UW Virology	Laboratory Medicine, UW Virology	Sereewit,J., Xie,H., Roychoudhury,P. and Greninger,A.L.
EPI_ISL_17118734, EPI_ISL_17118735, EPI_ISL_17118736, EPI_ISL_17118737	Environmental, Agricultural, and Occupational Health, University of Nebraska Medical Center	Environmental, Agricultural, and Occupational Health, University of Nebraska Medical Center	Gigante,C., Wang,Y., Zhao,H., Batra,D., Hetrick,E., Howard,D., Kovar,L., Seabolt,M., Knipe,K., Burroughs,M.S., Lee,J., Wilkins,K., McCollum,A., Hutson,C., Davidson,W., Rao,A., Stone,J. and Li,Y.
EPI_ISL_17118738, EPI_ISL_17118739	Environmental, Agricultural, and Occupational Health, University of Nebraska Medical Center	Environmental, Agricultural, and Occupational Health, University of Nebraska Medical Center	Pentella,M., Chapman,R.C., Stapleton,J., Meier,J., Xiang,J., Li,M., Reeb,V., Benfer,J., Eveland,K., Wiley,M.R., Hottel,W. and Cross,S.T.
EPI_ISL_17152764	Centers for Disease Control, R.O.C. (Taiwan)	Centers for Disease Control, R.O.C. (Taiwan)	Lin JH, Chiu SC, Huang HI, Huang WL, Li TY, Fann WB, Hsieh PY, Yang JY
EPI_ISL_17152842	Centers for Disease Control, R.O.C. (Taiwan)	Centers for Disease Control, R.O.C. (Taiwan)	Lin JH, Chiu SC, Huang HI, Huang LW, Li TY, Fann WB, Hsieh PY, Yang JY
EPI_ISL_17152843	Centers for Disease Control, R.O.C. (Taiwan)	Centers for Disease Control, R.O.C. (Taiwan)	Lin JH, Chiu SC, Huang HI, Huang WL, Li TY, Fann WB, Hsieh PY, Yang JY
EPI_ISL_17156262, EPI_ISL_17156263, EPI_ISL_17156264, EPI_ISL_17156265, EPI_ISL_17156266, EPI_ISL_17156267, EPI_ISL_17156268, EPI_ISL_17156269, EPI_ISL_17156270, EPI_ISL_17156271, EPI_ISL_17156272, EPI_ISL_17156273, EPI_ISL_17156274, EPI_ISL_17156275, EPI_ISL_17156276, EPI_ISL_17156277, EPI_ISL_17156278, EPI_ISL_17156279, EPI_ISL_17156280, EPI_ISL_17156281, EPI_ISL_17156282, EPI_ISL_17156283, EPI_ISL_17156284, EPI_ISL_17156285, EPI_ISL_17156286, EPI_ISL_17156287, EPI_ISL_17156288, EPI_ISL_17156289, EPI_ISL_17156290, EPI_ISL_17156291			
see above	Centre for Biological Threats, Highly Pathogenic Viruses, Robert Koch Institute	Centre for Biological Threats, Highly Pathogenic Viruses, Robert Koch Institute	Brinkmann,A., Kohl,C., Pape,K., Schrick,L., Michel,J., Schaade,L. and Nitsche,A.
EPI_ISL_17165661	Division of High Consequence Pathogens and Pathology (DHCPP) - Poxvirus and Rabies Branch (PRB), CDC	Division of High Consequence Pathogens and Pathology (DHCPP) - Poxvirus and Rabies Branch (PRB), CDC	Gigante,C., Kubin,G., Zhao,H., Batra,D., Hetrick,E., Howard,D., Kovar,L., Seabolt,M., Morrison,S., Desch,M., Knipe,K., Weigand,M., Cintron,R., Sheth,M., Burgin,A., Burroughs,M., Lee,J., Wilkins,K., McCollum,A., Hutson,C., Davidson,W., Rao,A., White,S. and Li,Y.
EPI_ISL_17165662	Division of High Consequence Pathogens and Pathology (DHCPP) - Poxvirus and Rabies Branch (PRB), CDC	Division of High Consequence Pathogens and Pathology (DHCPP) - Poxvirus and Rabies Branch (PRB), CDC	Gigante,C., Ventura,J., Zhao,H., Batra,D., Hetrick,E., Howard,D., Kovar,L., Seabolt,M., Morrison,S., Desch,M., Knipe,K., Weigand,M., Cintron,R., Sheth,M., Burgin,A., Burroughs,M., Lee,J., Wilkins,K., McCollum,A., Hutson,C., Davidson,W., Rao,A., Nash,J. and Li,Y.
EPI_ISL_17165663	Division of High Consequence Pathogens and Pathology (DHCPP) - Poxvirus and Rabies Branch (PRB), CDC	Division of High Consequence Pathogens and Pathology (DHCPP) - Poxvirus and Rabies Branch (PRB), CDC	Gigante,C., Steidley,B., Zhao,H., Batra,D., Hetrick,E., Howard,D., Kovar,L., Seabolt,M., Morrison,S., Desch,M., Knipe,K., Weigand,M., Cintron,R., Sheth,M., Burgin,A., Burroughs,M., Lee,J., Wilkins,K., McCollum,A., Hutson,C., Davidson,W., Rao,A., Davizon,E. and Li,Y.
EPI_ISL_17165664, EPI_ISL_17165665	Division of High Consequence Pathogens and Pathology (DHCPP) - Poxvirus and Rabies Branch (PRB), CDC	Division of High Consequence Pathogens and Pathology (DHCPP) - Poxvirus and Rabies Branch (PRB), CDC	Gigante,C., Alexander,S., Zhao,H., Batra,D., Hetrick,E., Howard,D., Kovar,L., Seabolt,M., Morrison,S., Desch,M., Knipe,K., Weigand,M., Cintron,R., Sheth,M., Burgin,A., Burroughs,M., Lee,J., Wilkins,K., McCollum,A., Hutson,C., Davidson,W., Rao,A., Pinnick,D. and Li,Y.
EPI_ISL_17165666, EPI_ISL_17165667	Division of High Consequence Pathogens and Pathology (DHCPP) - Poxvirus and Rabies Branch (PRB), CDC	Division of High Consequence Pathogens and Pathology (DHCPP) - Poxvirus and Rabies Branch (PRB), CDC	Gigante,C.M., Benton,C., Zhao,H., Batra,D., Hetrick,E.E., Howard,D.T., Kovar,L., Seabolt,M.H., Morrison,S.S., Desch,M., Knipe,K., Weigand,M.R., Cintron,R., Sheth,M., Burgin,A., Burroughs,M., Lee,J., Wilkins,K., McCollum,A., Hutson,C., Davidson,W., Rao,A., Bonner,K.O. and Li,Y.
EPI_ISL_17165668, EPI_ISL_17165669, EPI_ISL_17165670	Division of High Consequence Pathogens and Pathology (DHCPP) - Poxvirus and Rabies Branch (PRB), CDC	Division of High Consequence Pathogens and Pathology (DHCPP) - Poxvirus and Rabies Branch (PRB), CDC	Gigante,C., Ruiz,V., Zhao,H., Batra,D., Hetrick,E., Howard,D., Kovar,L., Seabolt,M., Morrison,S., Desch,M., Knipe,K., Weigand,M., Cintron,R., Burgin,A., Burroughs,M., Lee,J., Wilkins,K., McCollum,A., Hutson,C., Davidson,W., Rao,A., Wang,J. and Li,Y.
EPI_ISL_17165671, EPI_ISL_17165672, EPI_ISL_17165673, EPI_ISL_17165674, EPI_ISL_17165675, EPI_ISL_17165676, EPI_ISL_17165677, EPI_ISL_17165678	Division of High Consequence Pathogens and Pathology (DHCPP) - Poxvirus and Rabies Branch (PRB), CDC	Division of High Consequence Pathogens and Pathology (DHCPP) - Poxvirus and Rabies Branch (PRB), CDC	Gigante,C., Kubin,G., Zhao,H., Batra,D., Hetrick,E., Howard,D., Kovar,L., Seabolt,M., Morrison,S., Desch,M., Knipe,K., Weigand,M., Cintron,R., Sheth,M., Burgin,A., Burroughs,M., Lee,J., Wilkins,K., McCollum,A., Hutson,C., Davidson,W., Rao,A., White,S. and Li,Y.
EPI_ISL_17165679, EPI_ISL_17165680, EPI_ISL_17165681, EPI_ISL_17165683, EPI_ISL_17165684, EPI_ISL_17165685, EPI_ISL_17165686, EPI_ISL_17165687, EPI_ISL_17165688, EPI_ISL_17165689, EPI_ISL_17165690, EPI_ISL_17165691, EPI_ISL_17165692, EPI_ISL_17165693, EPI_ISL_17165694, EPI_ISL_17165695, EPI_ISL_17165696, EPI_ISL_17165697, EPI_ISL_17165698, EPI_ISL_17165699, EPI_ISL_17165700, EPI_ISL_17165701, EPI_ISL_17165702, EPI_ISL_17165703, EPI_ISL_17165704, EPI_ISL_17165705, EPI_ISL_17165707, EPI_ISL_17165708, EPI_ISL_17165709, EPI_ISL_17165710, EPI_ISL_17165711, EPI_ISL_17165712, EPI_ISL_17165713, EPI_ISL_17165714, EPI_ISL_17165715, EPI_ISL_17165716, EPI_ISL_17165717, EPI_ISL_17165718, EPI_ISL_17165719, EPI_ISL_17165720, EPI_ISL_17165721, EPI_ISL_17165722, EPI_ISL_17165723, EPI_ISL_17165724, EPI_ISL_17165725, EPI_ISL_17165726, EPI_ISL_17165727, EPI_ISL_17165728, EPI_ISL_17165729, EPI_ISL_17165730, EPI_ISL_17165731, EPI_ISL_17165732, EPI_ISL_17165733, EPI_ISL_17165734, EPI_ISL_17165735, EPI_ISL_17165736, EPI_ISL_17165737, EPI_ISL_17165738, EPI_ISL_17165739, EPI_ISL_17165740, EPI_ISL_17165741, EPI_ISL_17165742, EPI_ISL_17165743, EPI_ISL_17165744, EPI_ISL_17165745, EPI_ISL_17165746, EPI_ISL_17165747, EPI_ISL_17165748, EPI_ISL_17165749, EPI_ISL_17165750, EPI_ISL_17165751, EPI_ISL_17165752, EPI_ISL_17165753, EPI_ISL_17165754, EPI_ISL_17165755, EPI_ISL_17165756, EPI_ISL_17165757, EPI_ISL_17165758, EPI_ISL_17165759, EPI_ISL_17165760, EPI_ISL_17165761, EPI_ISL_17165762, EPI_ISL_17165763, EPI_ISL_17165764, EPI_ISL_17165765, EPI_ISL_17165766, EPI_ISL_17165767, EPI_ISL_17165768, EPI_ISL_17165769, EPI_ISL_17165770, EPI_ISL_17165771, EPI_ISL_17165772, EPI_ISL_17165773, EPI_ISL_17165774, EPI_ISL_17165775, EPI_ISL_17165776, EPI_ISL_17165777, EPI_ISL_17165778			
see above	Public Health Laboratory, NYC Department of Health and Mental Hygiene (DOHMH)	Public Health Laboratory, NYC Department of Health and Mental Hygiene (DOHMH)	Clabby,T.T., Amin,H.S., Wang,J.C., Taki,F., Su,M., Rahat,A., De La Cruz,N., Olsen,A., Thi,C., Silver,S., Akther,S., Chowdhury,M., Omoregie,E. and Hughes,S.
EPI_ISL_17170656, EPI_ISL_17170657, EPI_ISL_17170658, EPI_ISL_17170659, EPI_ISL_17170660, EPI_ISL_17170661, EPI_ISL_17170662, EPI_ISL_17170663, EPI_ISL_17170664, EPI_ISL_17170665, EPI_ISL_17170666, EPI_ISL_17170667, EPI_ISL_17170668, EPI_ISL_17170669, EPI_ISL_17170670, EPI_ISL_17170671			
see above	California Department of Public Health	California Department of Public Health	Haw, M. , Kath, C., Espinosa, A., O'Neil, R., and Hacker, J.
EPI_ISL_17179627, EPI_ISL_17179628	St Jame's Hospital, Virology Department	National Virus Reference Laboratory	Patrice Keane, Yvonne Lynagh, Brendan Crowley, Gabriel Gonzalez, Michael Carr, Emer O'Byrne, Weronika Banka, Brian Keogan, Jose Maria Urtasun Elizari, Jonathan Dean, Daniel Hare, Cillian F De Gascun
EPI_ISL_17179629, EPI_ISL_17179630, EPI_ISL_17179631, EPI_ISL_17179632	National Virus Reference Laboratory	National Virus Reference Laboratory	Gabriel Gonzalez, Michael Carr, Emer O'Byrne, Weronika Banka, Brian Keogan, Jose Maria Urtasun Elizari, Jonathan Dean, Daniel Hare, Cillian F De Gascun
EPI_ISL_17179633	St Jame's Hospital, Virology Department	National Virus Reference Laboratory	Patrice Keane, Yvonne Lynagh, Brendan Crowley, Gabriel Gonzalez, Michael Carr, Emer O'Byrne, Weronika Banka, Brian Keogan, Jose Maria Urtasun Elizari, Jonathan Dean, Daniel Hare, Cillian F De Gascun
EPI_ISL_17179634, EPI_ISL_17179635, EPI_ISL_17179636, EPI_ISL_17179637	National Virus Reference Laboratory	National Virus Reference Laboratory	Gabriel Gonzalez, Michael Carr, Emer O'Byrne, Weronika Banka, Brian Keogan, Jose Maria Urtasun Elizari, Jonathan Dean, Daniel Hare, Cillian F De Gascun
EPI_ISL_17179638, EPI_ISL_17179639, EPI_ISL_17179640, EPI_ISL_17179641, EPI_ISL_17179642, EPI_ISL_17179643	St Jame's Hospital, Virology Department	National Virus Reference Laboratory	Patrice Keane, Yvonne Lynagh, Brendan Crowley, Gabriel Gonzalez, Michael Carr, Emer O'Byrne, Weronika Banka, Brian Keogan, Jose Maria Urtasun Elizari, Jonathan Dean, Daniel Hare, Cillian F De Gascun
EPI_ISL_17187497, EPI_ISL_17187498	Vajira Hospital	Thai Red Cross Emerging Infectious Diseases Clinical Center and Faculty of Medicine, Chulalongkorn University	Suppasit srisaeng, Praepoly Ruekmuang, Kusuma Swangpun, Arriya Panchaiyaphum, Pakita Salaeh, Natpusda Kongmaung, Pornsiri Limwattanawong, Noree Pholprasert, Montriya Unteamsom, Kanjana Jeknok, Withak Withaksabut, Sunisa Nilda, Artorn Niakul, Sopon Iamsirithaworn, Thitipong Yingyong, Rossaporn Kittiyaowamarn, Rome Buathong, Ratanaporn Tangwangvivat, Supaporn Wacharapluesadee, Sininat Petcharat, Ananporn Supataragul, Stefan Fernandez, Achawin Rojanaviwat, Chonticha Klungthong, Pilailuk Okada, Khajohn Joonlasak, Chakkarat Pitayawonganon, Opass Putcharoen
EPI_ISL_17187499	Department of Disease Control, Ministry of Public Health	Thai Red Cross Emerging Infectious Diseases Clinical Center and Faculty of Medicine, Chulalongkorn University	Suppasit srisaeng, Praepoly Ruekmuang, Kusuma Swangpun, Arriya Panchaiyaphum, Pakita Salaeh, Natpusda Kongmaung, Pornsiri Limwattanawong, Noree Pholprasert, Montriya Unteamsom, Kanjana Jeknok, Withak Withaksabut, Sunisa Nilda, Artorn Niakul, Sopon Iamsirithaworn, Thitipong Yingyong, Rossaporn Kittiyaowamarn, Rome Buathong, Ratanaporn Tangwangvivat, Supaporn Wacharapluesadee, Sininat Petcharat, Ananporn Supataragul, Stefan Fernandez, Achawin Rojanaviwat, Chonticha Klungthong, Pilailuk Okada, Khajohn Joonlasak, Chakkarat Pitayawonganon, Opass Putcharoen
EPI_ISL_17187500, EPI_ISL_17187501	Bangkok Hospital Phuket	Thai Red Cross Emerging Infectious Diseases Clinical Center and Faculty of Medicine, Chulalongkorn University	Nungrathai Srisong, Praepoly Ruekmuang, Kusuma Swangpun, Arriya Panchaiyaphum, Pakita Salaeh, Natpusda Kongmaung, Pornsiri Limwattanawong, Noree Pholprasert, Montriya Unteamsom, Kanjana Jeknok, Withak Withaksabut, Sunisa Nilda, Artorn Niakul, Sopon Iamsirithaworn, Thitipong Yingyong, Rossaporn Kittiyaowamarn, Rome Buathong, Ratanaporn Tangwangvivat, Supaporn Wacharapluesadee, Sininat Petcharat, Ananporn Supataragul, Stefan Fernandez, Achawin Rojanaviwat, Chonticha Klungthong, Pilailuk Okada, Khajohn Joonlasak, Chakkarat Pitayawonganon, Opass Putcharoen
EPI_ISL_17187502	Department of Disease Control, Ministry of Public Health	Thai Red Cross Emerging Infectious Diseases Clinical Center and Faculty of Medicine, Chulalongkorn University	Supanut Chotichavalrattanakul, , Praepoly Ruekmuang, Kusuma Swangpun, Arriya Panchaiyaphum, Pakita Salaeh, Natpusda Kongmaung, Pornsiri Limwattanawong, Noree Pholprasert, Montriya Unteamsom, Kanjana Jeknok, Withak Withaksabut, Sunisa Nilda, Artorn Niakul, Sopon Iamsirithaworn, Thitipong Yingyong, Rossaporn Kittiyaowamarn, Rome Buathong, Ratanaporn Tangwangvivat, Supaporn Wacharapluesadee, Sininat Petcharat, Ananporn Supataragul, Stefan Fernandez, Achawin Rojanaviwat, Chonticha Klungthong, Pilailuk Okada, Khajohn Joonlasak, Chakkarat Pitayawonganon, Opass Putcharoen
EPI_ISL_17187503, EPI_ISL_17187504	Suvarnabhumi Airport	Thai Red Cross Emerging Infectious Diseases Clinical Center and Faculty of Medicine, Chulalongkorn University	Phawinee Montri, Praepoly Ruekmuang, Kusuma Swangpun, Arriya Panchaiyaphum, Pakita Salaeh, Natpusda Kongmaung, Pornsiri Limwattanawong, Noree Pholprasert, Montriya Unteamsom, Kanjana Jeknok, Withak Withaksabut, Sunisa Nilda, Artorn Niakul, Sopon Iamsirithaworn, Thitipong Yingyong, Rossaporn Kittiyaowamarn, Rome Buathong, Ratanaporn Tangwangvivat, Supaporn Wacharapluesadee, Sininat Petcharat, Ananporn Supataragul, Stefan Fernandez, Achawin Rojanaviwat, Chonticha Klungthong, Pilailuk Okada, Khajohn Joonlasak, Chakkarat Pitayawonganon, Opass Putcharoen
EPI_ISL_17187505	Phuket Provincial Public Health Office	Thai Red Cross Emerging Infectious Diseases Clinical Center and Faculty of Medicine, Chulalongkorn University	Nungrathai Srisong, Praepoly Ruekmuang, Kusuma Swangpun, Arriya Panchaiyaphum, Pakita Salaeh, Natpusda Kongmaung, Pornsiri Limwattanawong, Noree Pholprasert, Montriya Unteamsom, Kanjana Jeknok, Withak Withaksabut, Sunisa Nilda, Artorn Niakul, Sopon Iamsirithaworn, Thitipong Yingyong, Rossaporn Kittiyaowamarn, Rome Buathong, Ratanaporn Tangwangvivat, Supaporn Wacharapluesadee, Sininat Petcharat, Ananporn Supataragul, Stefan Fernandez, Achawin Rojanaviwat, Chonticha Klungthong, Pilailuk Okada, Khajohn Joonlasak, Chakkarat Pitayawonganon, Opass Putcharoen
EPI_ISL_17201439, EPI_ISL_17201440, EPI_ISL_17201441	Genomics Division, Instituto Tecnologico y de Energias Renovables (ITER)	Genomics Division, Instituto Tecnologico y de Energias Renovables (ITER)	Munoz-Barrera,A., Ciuffreda,L., Alcoba-Florez,J., Rubio-Rodriguez,L.A., Rodriguez-Perez,H., Gil-Campesino,H., Garcia-Martinez de Artola,D., Salas-Hernandez,J., Rodriguez-Nunez,J., Inigo-Campos,A., Garcia-Olivares,V., Diez-Gil,O., Gonzalez-Montelongo,R., Valenzuela-Fernandez,A., Lorenzo-Salazar,J.M. and Flores,C.
EPI_ISL_17201443	Genomics Division, Instituto Tecnologico y de Energias Renovables (ITER)	Genomics Division, Instituto Tecnologico y de Energias Renovables (ITER)	Munoz Barrera,A., Ciuffreda,L., Alcoba-Florez,J., Rubio-Rodriguez,L.A., Rodriguez-Perez,H., Gil-Campesino,H., Garcia-Martinez de Artola,D., Salas-Hernandez,J., Rodriguez-Nunez,J., Inigo-Campos,A., Garcia-Olivares,V., Diez-Gil,O., Gonzalez-Montelongo,R., Valenzuela-Fernandez,A., Lorenzo-Salazar,J.M. and Flores,C.
EPI_ISL_17201444	Genomics Division, Instituto Tecnologico y de Energias Renovables (ITER)	Genomics Division, Instituto Tecnologico y de Energias Renovables (ITER)	Munoz-Barrera,A., Ciuffreda,L., Alcoba-Florez,J., Rubio-Rodriguez,L.A., Rodriguez-Perez,H., Gil-Campesino,H., Garcia-Martinez de Artola,D., Salas-Hernandez,J., Rodriguez-Nunez,J., Inigo-Campos,A., Garcia-Olivares,V., Diez-Gil,O., Gonzalez-Montelongo,R., Valenzuela-Fernandez,A., Lorenzo-Salazar,J.M. and Flores,C.
EPI_ISL_17206607, EPI_ISL_17206608, EPI_ISL_17206609, EPI_ISL_17206610, EPI_ISL_17206611, EPI_ISL_17206612, EPI_ISL_17206613, EPI_ISL_17206614, EPI_ISL_17206615, EPI_ISL_17206616, EPI_ISL_17206617, EPI_ISL_17206618, EPI_ISL_17206619, EPI_ISL_17206620, EPI_ISL_17206621, EPI_ISL_17206622			
see above	California Department of Public Health	California Department of Public Health	Haw,M., Kath,C., Espinosa,A., O'Neil,R., and Hacker,J.
EPI_ISL_17211323	Los Angeles County Public Health Laboratories	Los Angeles County Public Health Laboratories	P. Hemarajata et al.
EPI_ISL_17211324, EPI_ISL_17211325, EPI_ISL_17211326, EPI_ISL_17211327, EPI_ISL_17211328, EPI_ISL_17211329, EPI_ISL_17211330	Kaiser Permanente Chino Hills Regional Reference Laboratories	Los Angeles County Public Health Laboratories	P. Hemarajata et al.
EPI_ISL_17211331	Los Angeles County Public Health Laboratories	Los Angeles County Public Health Laboratories	P. Hemarajata et al.
EPI_ISL_17211332	Quest Diagnostics Nichols Institute	Los Angeles County Public Health Laboratories	P. Hemarajata et al.
EPI_ISL_17211333	Los Angeles County Public Health Laboratories	Los Angeles County Public Health Laboratories	P. Hemarajata et al.

EPI_ISL_17211335	Laboratory Corporation of America	Los Angeles County Public Health Laboratories	P. Hemarajata et al.
EPI_ISL_17222811, EPI_ISL_17222812, EPI_ISL_17222813, EPI_ISL_17222814, EPI_ISL_17222816, EPI_ISL_17222817, EPI_ISL_17222818, EPI_ISL_17222819, EPI_ISL_17222820, EPI_ISL_17222822, EPI_ISL_17222823, EPI_ISL_17222824, EPI_ISL_17222825, EPI_ISL_17222827, EPI_ISL_17222828, EPI_ISL_17222829			
see above	Viral and Rickettsial Disease Laboratory (VRDL) California Department of Public Health (CDPH)	Viral and Rickettsial Disease Laboratory (VRDL) California Department of Public Health (CDPH)	Haw,M., Kath,C., Espinosa,A., O'Neil,R. and Hacker,J.
EPI_ISL_17246657, EPI_ISL_17246659	Fumi Kasuya Tokyo Metropolitan Institute of Public Health, Department of Microbiology	Fumi Kasuya Tokyo Metropolitan Institute of Public Health, Department of Microbiology	Kasuya,F., Negishi,A., Kumagai,R., Hasegawa,M., Fujiwara,T., Miyake,H., Nagashima,M. and Sadamasu,K.
EPI_ISL_17269833, EPI_ISL_17269834, EPI_ISL_17269835, EPI_ISL_17269836, EPI_ISL_17269837, EPI_ISL_17269838, EPI_ISL_17269839	Environmental, Agricultural, and Occupational Health, University of Nebraska Medical Center	Environmental, Agricultural, and Occupational Health, University of Nebraska Medical Center	Tegomoh,B., Cross,S.T., Chapman,R.C., Bernhard,K., McCutchen,E.L., Fauver,J.R., Pratt,C.B., Warden,D.E., Iwen,P.C., Donahue,M. and Wiley,M.R.
EPI_ISL_17271956, EPI_ISL_17271957	Rhode Island State Health Laboratory	Rhode Island State Health Laboratory	Kristin Carpenter-Azevedo, Sean Sierra-Patev, Richard C. Huard
EPI_ISL_17319546, EPI_ISL_17319547, EPI_ISL_17319548, EPI_ISL_17319549, EPI_ISL_17319550, EPI_ISL_17319551, EPI_ISL_17319554, EPI_ISL_17319555	Department of Clinical Sciences, Institute of Tropical Medicine	Department of Clinical Sciences, Institute of Tropical Medicine	Mertes,H., Rezende,A.M., Naesens,R., de Block,T., Michiels,J., Coppens,J., Van Dijck,C., Bomans,P., Arien,K., Bottieau,E., Van Esbroeck,M., Liesenborghs,L. and Vercauteren,K.
EPI_ISL_17319556	Center of Diagnostics and Vaccine Development, Centers for Disease Control	Center of Diagnostics and Vaccine Development, Centers for Disease Control	Lin,J.-H., Chiu,S.-C., Huang,H.-I., Huang,W.-L., Li,T.-Y., Fann,W.-B., Hsieh,P.-Y. and Yang,J.-Y.
EPI_ISL_17383630, EPI_ISL_17383632, EPI_ISL_17383634, EPI_ISL_17383635, EPI_ISL_17383636, EPI_ISL_17383637, EPI_ISL_17383639, EPI_ISL_17383641, EPI_ISL_17383645	Laboratorio Departamental de Salud Publica de Antioquia	Laboratorio Departamental de Salud Publica de Antioquia	Betancur,I.I.B., Velarde Hoyos,C.A.C.V., Gomez,R.R.G. and Mercado-Reyes,M.M.R.
EPI_ISL_17390796, EPI_ISL_17390797, EPI_ISL_17390799, EPI_ISL_17390801, EPI_ISL_17390803, EPI_ISL_17390804	Antioquia, Laboratorio Departamental de Salud Publica de Antioquia	Antioquia, Laboratorio Departamental de Salud Publica de Antioquia	Betancur,I.I.B., Velarde Hoyos,C.A.C.V., Gomez,R.R.G. and Mercado-Reyes,M.M.R.
EPI_ISL_17394091, EPI_ISL_17394092	Division of High-Consequence Pathogens and Pathology - Poxvirus and Rabies Branch (DHCPP-PRB), Centers for Disease Control and Prevention (CDC)	Division of High-Consequence Pathogens and Pathology - Poxvirus and Rabies Branch (DHCPP-PRB), Centers for Disease Control and Prevention (CDC)	Gigante,C., Ostadkar,R., Zhao,H., Batra,D., Hetrick,E., Howard,D., Kovar,L., Seabolt,M., Morrison,S., Desch,M., Knipe,K., Weigand,M., Cintron,R., Burgin,A., Burroughs,M., Lee,J., Wilkins,K., McCollum,A., Hutson,C., Davidson,W., Rao,A., Wang,X. and Li,Y.
EPI_ISL_17394093	Division of High-Consequence Pathogens and Pathology - Poxvirus and Rabies Branch (DHCPP-PRB), Centers for Disease Control and Prevention (CDC)	Division of High-Consequence Pathogens and Pathology - Poxvirus and Rabies Branch (DHCPP-PRB), Centers for Disease Control and Prevention (CDC)	Gigante,C., Wang,Y., Zhao,H., Batra,D., Hetrick,E., Howard,D., Kovar,L., Seabolt,M., Morrison,S., Desch,M., Knipe,K., Weigand,M., Cintron,R., Burgin,A., Burroughs,M., Lee,J., Wilkins,K., McCollum,A., Hutson,C., Davidson,W., Rao,A., O'Dell,J. and Li,Y.
EPI_ISL_17394094	Division of High-Consequence Pathogens and Pathology - Poxvirus and Rabies Branch (DHCPP-PRB), Centers for Disease Control and Prevention (CDC)	Division of High-Consequence Pathogens and Pathology - Poxvirus and Rabies Branch (DHCPP-PRB), Centers for Disease Control and Prevention (CDC)	Gigante,C., Cogswell,K., Zhao,H., Batra,D., Hetrick,E., Howard,D., Kovar,L., Seabolt,M., Morrison,S., Desch,M., Knipe,K., Weigand,M., Cintron,R., Burgin,A., Burroughs,M., Lee,J., Wilkins,K., McCollum,A., Hutson,C., Davidson,W., Rao,A., Grenz,L. and Li,Y.
EPI_ISL_17394095	Division of High-Consequence Pathogens and Pathology - Poxvirus and Rabies Branch (DHCPP-PRB), Centers for Disease Control and Prevention (CDC)	Division of High-Consequence Pathogens and Pathology - Poxvirus and Rabies Branch (DHCPP-PRB), Centers for Disease Control and Prevention (CDC)	Gigante,C., Xia,D., Zhao,H., Batra,D., Hetrick,E., Howard,D., Kovar,L., Seabolt,M., Morrison,S., Desch,M., Knipe,K., Weigand,M., Cintron,R., Burgin,A., Burroughs,M., Lee,J., Wilkins,K., McCollum,A., Hutson,C., Davidson,W., Rao,A., Plipat,N. and Li,Y.
EPI_ISL_17394096, EPI_ISL_17394097, EPI_ISL_17394098	Division of High-Consequence Pathogens and Pathology - Poxvirus and Rabies Branch (DHCPP-PRB), Centers for Disease Control and Prevention (CDC)	Division of High-Consequence Pathogens and Pathology - Poxvirus and Rabies Branch (DHCPP-PRB), Centers for Disease Control and Prevention (CDC)	Gigante,C., Berns,A., Zhao,H., Batra,D., Hetrick,E., Howard,D., Kovar,L., Seabolt,M., Morrison,S., Desch,M., Knipe,K., Weigand,M., Cintron,R., Burgin,A., Burroughs,M., Lee,J., Wilkins,K., McCollum,A., Hutson,C., Davidson,W., Rao,A., Carpenter-Azevedo,K. and Li,Y.
EPI_ISL_17394099, EPI_ISL_17394100	Division of High-Consequence Pathogens and Pathology - Poxvirus and Rabies Branch (DHCPP-PRB), Centers for Disease Control and Prevention (CDC)	Division of High-Consequence Pathogens and Pathology - Poxvirus and Rabies Branch (DHCPP-PRB), Centers for Disease Control and Prevention (CDC)	Gigante,C., Kubin,G., Zhao,H., Batra,D., Hetrick,E., Howard,D., Kovar,L., Seabolt,M., Morrison,S., Desch,M., Knipe,K., Weigand,M., Cintron,R., Burgin,A., Burroughs,M., Lee,J., Wilkins,K., McCollum,A., Hutson,C., Davidson,W., Rao,A., White,S. and Li,Y.
EPI_ISL_17394101, EPI_ISL_17394102, EPI_ISL_17394103, EPI_ISL_17394104, EPI_ISL_17394105	Division of High-Consequence Pathogens and Pathology - Poxvirus and Rabies Branch (DHCPP-PRB), Centers for Disease Control and Prevention (CDC)	Division of High-Consequence Pathogens and Pathology - Poxvirus and Rabies Branch (DHCPP-PRB), Centers for Disease Control and Prevention (CDC)	Gigante,C., Haydel,D., Zhao,H., Batra,D., Hetrick,E., Howard,D., Kovar,L., Seabolt,M., Morrison,S., Desch,M., Knipe,K., Weigand,M., Cintron,R., Burgin,A., Burroughs,M., Lee,J., Wilkins,K., McCollum,A., Hutson,C., Davidson,W., Rao,A., Salinas,A. and Li,Y.
EPI_ISL_17394106, EPI_ISL_17394107	Division of High-Consequence Pathogens and Pathology - Poxvirus and Rabies Branch (DHCPP-PRB), Centers for Disease Control and Prevention (CDC)	Division of High-Consequence Pathogens and Pathology - Poxvirus and Rabies Branch (DHCPP-PRB), Centers for Disease Control and Prevention (CDC)	Gigante,C., Ostadkar,R., Zhao,H., Batra,D., Hetrick,E., Howard,D., Kovar,L., Seabolt,M., Morrison,S., Desch,M., Knipe,K., Weigand,M., Cintron,R., Burgin,A., Burroughs,M., Lee,J., Wilkins,K., McCollum,A., Hutson,C., Davidson,W., Rao,A., Wang,X. and Li,Y.
EPI_ISL_17406093, EPI_ISL_17406094, EPI_ISL_17406095, EPI_ISL_17406096, EPI_ISL_17406097, EPI_ISL_17406098, EPI_ISL_17406099, EPI_ISL_17406100, EPI_ISL_17406101, EPI_ISL_17406102, EPI_ISL_17406103, EPI_ISL_17406104, EPI_ISL_17406105, EPI_ISL_17406106, EPI_ISL_17406107, EPI_ISL_17406108, EPI_ISL_17406109, EPI_ISL_17406110, EPI_ISL_17406111, EPI_ISL_17406112, EPI_ISL_17406113, EPI_ISL_17406114, EPI_ISL_17406115, EPI_ISL_17406116, EPI_ISL_17406117, EPI_ISL_17406118, EPI_ISL_17406119, EPI_ISL_17406120, EPI_ISL_17406121, EPI_ISL_17406122, EPI_ISL_17406123, EPI_ISL_17406124			
see above	CDCT/CEVS/SES-RS	CDCT/CEVS/SES-RS	Richard Steiner Salvato, Fernanda Marques Godinho, Regina Bones Barcellos, Patricia Sesterheim, Amanda Pellenz Ruivo, Viviane Horn de Melo, Júlio Augusto Schroder
EPI_ISL_17424657, EPI_ISL_17424658, EPI_ISL_17424659, EPI_ISL_17424660, EPI_ISL_17424661, EPI_ISL_17424662, EPI_ISL_17424663, EPI_ISL_17424664, EPI_ISL_17424665, EPI_ISL_17424666, EPI_ISL_17424667, EPI_ISL_17424668, EPI_ISL_17424669, EPI_ISL_17424670, EPI_ISL_17424671, EPI_ISL_17424673, EPI_ISL_17424674, EPI_ISL_17424675, EPI_ISL_17424676, EPI_ISL_17424677, EPI_ISL_17424678			
see above	Molecular Microbiology Laboratory, Department of Pathology, Molecular and Cell-Based Medicine, Icahn School of Medicine at Mount Sinai,	Molecular Microbiology Laboratory, Department of Pathology, Molecular and Cell-Based Medicine, Icahn School of Medicine at Mount Sinai,	Luz H. Patiño, Susana Guerra, Marina Muñoz, Nicolas Luna , Keith Farrugia, Adriana van de Guchte, Zain Khalil , Ana Silvia Gonzalez-Reiche, Matthew M. Hernandez ,Radhika Banu, Paras Shrestha, Bernadette Liggayu, Adolfo Firpo Betancourt, David Reich, Carlos Cordon-Cardo, Randy Albrechtf, Rebecca Pearlf, Viviana Simona, Aria Rookera, Emilia Mia Sordillo, Harm van Bakeld, Adolfo García-Sastre, Dusan Bogunovic, Gustavo Palacios, Alberto Paniz Mondolfi, Juan David Ramírez
EPI_ISL_17428282, EPI_ISL_17428283, EPI_ISL_17428284, EPI_ISL_17428286	Center of Diagnostics and Vaccine Development, Centers for Disease Control	Center of Diagnostics and Vaccine Development, Centers for Disease Control	Lin,J.-H., Chiu,S.-C., Huang,H.-I., Huang,W.-L., Li,T.-Y., Fann,W.-B., Hsieh,P.-Y. and Yang,J.-Y.
EPI_ISL_17428287	Centers for Disease Control and Prevention DHCPP-PRB, CDC	Centers for Disease Control and Prevention DHCPP-PRB, CDC	Gigante,C., Francis,D., Zhao,H., Batra,D., Hetrick,E., Howard,D., Kovar,L., Seabolt,M., Morrison,S., Desch,M., Knipe,K., Weigand,M., Cintron,R., Burgin,A., Burroughs,M., Lee,J., Wilkins,K., McCollum,A., Hutson,C., Davidson,W., Rao,A., Escobar,J. and Li,Y.
EPI_ISL_17428289	Centers for Disease Control and Prevention DHCPP-PRB, CDC	Centers for Disease Control and Prevention DHCPP-PRB, CDC	Gigante,C., Vang,K., Zhao,H., Batra,D., Hetrick,E., Howard,D., Kovar,L., Seabolt,M., Morrison,S., Desch,M., Knipe,K., Weigand,M., Cintron,R., Burgin,A., Burroughs,M., Lee,J., Wilkins,K., McCollum,A., Hutson,C., Davidson,W., Rao,A., Seely,K. and Li,Y.
EPI_ISL_17428290, EPI_ISL_17428291	Centers for Disease Control and Prevention DHCPP-PRB, CDC	Centers for Disease Control and Prevention DHCPP-PRB, CDC	Gigante,C., Ghinai,I., Zhao,H., Batra,D., Hetrick,E., Howard,D., Kovar,L., Seabolt,M., Morrison,S., Desch,M., Knipe,K., Weigand,M., Cintron,R., Burgin,A., Burroughs,M., Lee,J., Wilkins,K., McCollum,A., Hutson,C., Davidson,W., Rao,A., Kerins,J. and Li,Y.
EPI_ISL_17428292, EPI_ISL_17428293	Centers for Disease Control and Prevention DHCPP-PRB, CDC	Centers for Disease Control and Prevention DHCPP-PRB, CDC	Gigante,C., Bradley,A., Zhao,H., Batra,D., Hetrick,E., Howard,D., Kovar,L., Seabolt,M., Morrison,S., Desch,M., Knipe,K., Weigand,M., Cintron,R., Burgin,A., Burroughs,M., Lee,J., Wilkins,K., McCollum,A., Hutson,C., Davidson,W., Rao,A., Anderson,J. and Li,Y.
EPI_ISL_17428294, EPI_ISL_17428295	Centers for Disease Control and Prevention DHCPP-PRB, CDC	Centers for Disease Control and Prevention DHCPP-PRB, CDC	Gigante,C., Johnson,S., Zhao,H., Batra,D., Hetrick,E., Howard,D., Kovar,L., Seabolt,M., Morrison,S., Desch,M., Knipe,K., Weigand,M., Cintron,R., Burgin,A., Burroughs,M., Lee,J., Wilkins,K., McCollum,A., Hutson,C., Davidson,W., Rao,A., Riner,D. and Li,Y.
EPI_ISL_17428297	Centers for Disease Control and Prevention DHCPP-PRB, CDC	Centers for Disease Control and Prevention DHCPP-PRB, CDC	Gigante,C., Ostadkar,R., Zhao,H., Batra,D., Hetrick,E., Howard,D., Kovar,L., Seabolt,M., Morrison,S., Desch,M., Knipe,K., Weigand,M., Cintron,R., Burgin,A., Burroughs,M., Lee,J., Wilkins,K., McCollum,A., Hutson,C., Davidson,W., Rao,A., Wang,X. and Li,Y.
EPI_ISL_17428298, EPI_ISL_17428300, EPI_ISL_17428302	Centers for Disease Control and Prevention DHCPP-PRB, CDC	Centers for Disease Control and Prevention DHCPP-PRB, CDC	Gigante,C., Cleavinger,K., Zhao,H., Batra,D., Hetrick,E., Howard,D., Kovar,L., Seabolt,M., Morrison,S., Desch,M., Knipe,K., Weigand,M., Cintron,R., Burgin,A., Burroughs,M., Lee,J., Wilkins,K., McCollum,A., Hutson,C., Davidson,W., Rao,A., Sinn,M. and Li,Y.
EPI_ISL_17428303, EPI_ISL_17428305	Centers for Disease Control and Prevention DHCPP-PRB, CDC	Centers for Disease Control and Prevention DHCPP-PRB, CDC	Gigante,C., Fisher,S., Zhao,H., Batra,D., Hetrick,E., Howard,D., Kovar,L., Seabolt,M., Morrison,S., Desch,M., Knipe,K., Weigand,M., Cintron,R., Burgin,A., Burroughs,M., Lee,J., Wilkins,K., McCollum,A., Hutson,C., Davidson,W., Rao,A., Siebert,M. and Li,Y.
EPI_ISL_17428306, EPI_ISL_17428307	Centers for Disease Control and Prevention DHCPP-PRB, CDC	Centers for Disease Control and Prevention DHCPP-PRB, CDC	Gigante,C., Ruiz,V., Zhao,H., Batra,D., Hetrick,E., Howard,D., Kovar,L., Seabolt,M., Morrison,S., Desch,M., Knipe,K., Weigand,M., Cintron,R., Burgin,A., Burroughs,M., Lee,J., Wilkins,K., McCollum,A., Hutson,C., Davidson,W., Rao,A., Wang,J. and Li,Y.
EPI_ISL_17428308, EPI_ISL_17428309, EPI_ISL_17428311	Centers for Disease Control and Prevention DHCPP-PRB, CDC	Centers for Disease Control and Prevention DHCPP-PRB, CDC	Gigante,C., Lee,B., Zhao,H., Batra,D., Hetrick,E., Howard,D., Kovar,L., Seabolt,M., Morrison,S., Desch,M., Knipe,K., Weigand,M., Cintron,R., Burgin,A., Burroughs,M., Lee,J., Wilkins,K., McCollum,A., Hutson,C., Davidson,W., Rao,A., Salehi,E. and Li,Y.
EPI_ISL_17428313, EPI_ISL_17428315, EPI_ISL_17428316	Centers for Disease Control and Prevention DHCPP-PRB, CDC	Centers for Disease Control and Prevention DHCPP-PRB, CDC	Gigante,C., Epie,N., Zhao,H., Batra,D., Hetrick,E., Howard,D., Kovar,L., Seabolt,M., Morrison,S., Desch,M., Knipe,K., Weigand,M., Cintron,R., Burgin,A., Burroughs,M., Lee,J., Wilkins,K., McCollum,A., Hutson,C., Davidson,W., Rao,A., Perez,T. and Li,Y.
EPI_ISL_17428317, EPI_ISL_17428319, EPI_ISL_17428321	Centers for Disease Control and Prevention DHCPP-PRB, CDC	Centers for Disease Control and Prevention DHCPP-PRB, CDC	Gigante,C., Thomas,L., Zhao,H., Batra,D., Hetrick,E., Howard,D., Kovar,L., Seabolt,M., Morrison,S., Desch,M., Knipe,K., Weigand,M., Cintron,R., Burgin,A., Burroughs,M., Lee,J., Wilkins,K., McCollum,A., Hutson,C., Davidson,W., Rao,A., Dunn,J. and Li,Y.
EPI_ISL_17428323	Centers for Disease Control and Prevention DHCPP-PRB, CDC	Centers for Disease Control and Prevention DHCPP-PRB, CDC	Gigante,C., Kubin,G., Zhao,H., Batra,D., Hetrick,E., Howard,D., Kovar,L., Seabolt,M., Morrison,S., Desch,M., Knipe,K., Weigand,M., Cintron,R., Burgin,A., Burroughs,M., Lee,J., Wilkins,K., McCollum,A., Hutson,C., Davidson,W., Rao,A., White,S. and Li,Y.
EPI_ISL_17428325, EPI_ISL_17428326, EPI_ISL_17428328	Centers for Disease Control and Prevention DHCPP-PRB, CDC	Centers for Disease Control and Prevention DHCPP-PRB, CDC	Gigante,C., Segaloff,H., Zhao,H., Batra,D., Hetrick,E., Howard,D., Kovar,L., Seabolt,M., Morrison,S., Desch,M., Knipe,K., Weigand,M., Cintron,R., Burgin,A., Burroughs,M., Lee,J., Wilkins,K., McCollum,A., Hutson,C., Davidson,W., Rao,A., Florek,K. and Li,Y.
EPI_ISL_17445514, EPI_ISL_17445515, EPI_ISL_17445516, EPI_ISL_17445517, EPI_ISL_17445518, EPI_ISL_17445519	Tokyo Metropolitan Institute of Public Health	Tokyo Metropolitan Institute of Public Health	Fumi Kasuya, Wakaba Okada, Ryota Kumagai, Sachiko Harada, Arisa Amano, Michiya Hasegawa, Mami Nagashima, Kenji Sadamasu
EPI_ISL_17471100, EPI_ISL_17471101, EPI_ISL_17471102, EPI_ISL_17471103, EPI_ISL_17471104, EPI_ISL_17471105, EPI_ISL_17471106, EPI_ISL_17471107, EPI_ISL_17471108, EPI_ISL_17471109, EPI_ISL_17471110			
see above	Laboratorio de Enterovirus, Instituto Oswaldo Cruz, Fiocruz	Laboratory of Respiratory Viruses and Measles, Oswaldo Cruz Institute, FIOCRUZ	Paola Resende, Elisa Cavalcante Pereira, Bruna Mendonça da Silva, Jéssica Graça Macedo de Carvalho, Larissa Macedo Pinto, Victor Guimaraes, Marilda Siqueira, Renan da Silva Faustino, Marilia Santini, Beatriz Grinsztejn, Mayara Secco Torres da Silva, Edson Elias da Silva on behalf of the Fiocruz Genomic Surveillance Network
EPI_ISL_17472037	Division of Infectious Disease Vaccine Research, Korea National Institute of Health	Division of Infectious Disease Vaccine Research, Korea National Institute of Health	Lee,T.Y., Hwang,Y.-H., Yun,M.-R., Kim,Y.-J. and Kim,D.
EPI_ISL_17472041, EPI_ISL_17472042, EPI_ISL_17472043, EPI_ISL_17472044, EPI_ISL_17472045, EPI_ISL_17472046,	DPH, Massachusetts State Public Health Laboratory	DPH, Massachusetts State Public Health Laboratory	Doucette,M., Gagne,L. and Smole,S.

EPI_ISL_17472047			
EPI_ISL_17485343	Laboratorio de Enterovirus, Instituto Oswaldo Cruz, Fiocruz	Instituto Oswaldo Cruz FIOCRUZ - Laboratory of Respiratory Viruses and Measles (LVRS)	Paola Resende, Elisa Cavalcante Pereira, Bruna Mendonça da Silva, Jéssica Graça Macedo de Carvalho, Larissa Macedo Pinto, Victor Guimaraes, Marilda Siqueira, Renan da Silva Faustino, Marilia Santini, Beatriz Grinsztejn, Mayara Secco Torres da Silva, Edson Elias da Silva on behalf of the Fiocruz Genomic Surveillance Network
EPI_ISL_17485440, EPI_ISL_17485441, EPI_ISL_17485442, EPI_ISL_17485443, EPI_ISL_17485444, EPI_ISL_17485445, EPI_ISL_17485446, EPI_ISL_17485447, EPI_ISL_17485448, EPI_ISL_17485449, EPI_ISL_17485450, EPI_ISL_17485451, EPI_ISL_17485452, EPI_ISL_17485453, EPI_ISL_17485454, EPI_ISL_17485455, EPI_ISL_17485456, EPI_ISL_17485457, EPI_ISL_17485458, EPI_ISL_17485459, EPI_ISL_17485460, EPI_ISL_17485461, EPI_ISL_17485462, EPI_ISL_17485463, EPI_ISL_17485464, EPI_ISL_17485465, EPI_ISL_17485466, EPI_ISL_17485467, EPI_ISL_17485468, EPI_ISL_17485469, EPI_ISL_17485470, EPI_ISL_17485471, EPI_ISL_17485472, EPI_ISL_17485473, EPI_ISL_17485474, EPI_ISL_17485475, EPI_ISL_17485476, EPI_ISL_17485477, EPI_ISL_17485478, EPI_ISL_17485479, EPI_ISL_17485480, EPI_ISL_17485481, EPI_ISL_17485482, EPI_ISL_17485483, EPI_ISL_17485484, EPI_ISL_17485485, EPI_ISL_17485486, EPI_ISL_17485487, EPI_ISL_17485488, EPI_ISL_17485489, EPI_ISL_17485490, EPI_ISL_17485491, EPI_ISL_17485492, EPI_ISL_17485493, EPI_ISL_17485494, EPI_ISL_17485495, EPI_ISL_17485496, EPI_ISL_17485497, EPI_ISL_17485498, EPI_ISL_17485499, EPI_ISL_17485500, EPI_ISL_17485501, EPI_ISL_17485502, EPI_ISL_17485503, EPI_ISL_17485504, EPI_ISL_17485505, EPI_ISL_17485506, EPI_ISL_17485507, EPI_ISL_17485508, EPI_ISL_17485509, EPI_ISL_17485510, EPI_ISL_17485511, EPI_ISL_17485512, EPI_ISL_17485513, EPI_ISL_17485514, EPI_ISL_17485515, EPI_ISL_17485516, EPI_ISL_17485517, EPI_ISL_17485518, EPI_ISL_17485519, EPI_ISL_17485520, EPI_ISL_17485521, EPI_ISL_17485522, EPI_ISL_17485523, EPI_ISL_17485524, EPI_ISL_17485525, EPI_ISL_17485526, EPI_ISL_17485527, EPI_ISL_17485528, EPI_ISL_17485529, EPI_ISL_17485530, EPI_ISL_17485531, EPI_ISL_17485532, EPI_ISL_17485533, EPI_ISL_17485534, EPI_ISL_17485535, EPI_ISL_17485536, EPI_ISL_17485537, EPI_ISL_17485538, EPI_ISL_17485539, EPI_ISL_17485540, EPI_ISL_17485541, EPI_ISL_17485542			
see above	Centre for Biological Threats, Highly Pathogenic Viruses, Robert Koch Institute	Centre for Biological Threats, Highly Pathogenic Viruses, Robert Koch Institute	Brinkmann,A., Kohl,C., Schrick,L., Michel,J., Schaade,L. and Nitsche,A.
EPI_ISL_17502583	Public Health Laboratory, Public Health Service Amsterdam, The Netherlands	Department of Medical Microbiology & Infection prevention, Amsterdam University Medical Centers location AMC	Matthijs Welkers, Jelle Koopsen, Robin van Houdt, Marcel Jonges, Sebastien Matamoros, Sjoerd Rebers, Fokla Zorgdrager, Sylvia Bruisten, Akke Cornelissen, Janke Schinkel, Ewout Fanoy, Roisin Bavalia, Menno de Jong and Mariken van der Lubben on behalf of the Amsterdam Regional Genomic epidemiology and Outbreak Surveillance (ARGOS) consortium
EPI_ISL_17518107	Virology Section, Division of Microbiology,Osaka Institute of Public Health	Virology Section, Division of Microbiology,Osaka Institute of Public Health	Daiki Kanbayashi, Takako Kurata, Takuya Kawahata, Fumiya Bannno, Minami Hama, Kazushi Motomuta
EPI_ISL_17525484	Division de Microbiologia, Hospital Nacional de Niños Carlos Saenz Herrera	Inciensa, Investigación y Enseñanza en Nutrición y Salud Centro Nacional de Referencia de Virología	Cristian Perez Corrales, Christopher Mairena Acuña, Diana Cantillo, Hillary Serrano, Ana Isela Ruiz, Gustavo Vega, Claudio Soto-Garita, Adriana Godinez, Estela Cordero, Melany Calderon, Francisco Duarte
EPI_ISL_17529368	Laboratorio de Virus Exantematicos, Gastroentéricos y Otros Transmitidos por Vectores	Centro de Referencia Nacional de Genomica, Secuenciacion y Bioinformatica GENSBIO, INSPI-CZ9	Andrés Carrazco*, SIlvia Salgado, Diana Gutiérrez, Damaris Alarcón, Andrés Tinizaray, Ruth Gómez, Martha Sánchez, Johanna Parrales, Eva Nicola, Jorge Bejarano, Leandro Patiño.
EPI_ISL_17536780	Department of Virology, National Institute of Health, Islamabad, Pakistan	Department of Virology, National Institute of Health, Islamabad, Pakistan	Massab Umair, Muhammad Ammar, Syed Adnan Haider, Rabia Hakim, Qasim Malik, Muhammad Salman, Ghazala Parveen, and Naseem Akhtar
EPI_ISL_17536782, EPI_ISL_17536783, EPI_ISL_17536784, EPI_ISL_17536785	Laboratorio de Enterovirus, Instituto Oswaldo Cruz, Fiocruz	Instituto Oswaldo Cruz FIOCRUZ - Laboratory of Respiratory Viruses and Measles (LVRS)	Paola Resende, Elisa Cavalcante Pereira, Bruna Mendonça da Silva, Jéssica Graça Macedo de Carvalho, Larissa Macedo Pinto, Victor Guimaraes, Marilda Siqueira, Renan da Silva Faustino, Marilia Santini, Edson Elias da Silva on behalf of the Fiocruz Genomic Surveillance Network
EPI_ISL_17582853	Quest Diagnostics Nichols Institute	Los Angeles County Public Health Laboratories	P. Hemarajata et al.
EPI_ISL_17584292	Centro Medico ABC	Instituto Nacional de Medicina Genomica	Cedro Tanda Alberto, Roxana Trejo González, Laura Gomez-Romero, Alfredo Mendoza-Vargas, Dora Garnica-Lopez, Alfredo Hidalgo-Miranda, Luis A Herrera.
EPI_ISL_17592665, EPI_ISL_17592666, EPI_ISL_17592667, EPI_ISL_17592668, EPI_ISL_17592669, EPI_ISL_17592670	Tokyo Metropolitan Institute of Public Health	Tokyo Metropolitan Institute of Public Health	Fumi Kasuya, Wakaba Okada, Ryota Kumagai, Sachiko Harada, Arisa Amano, Michiya Hasegawa, Mami Nagashima, Kenji Sadamasu
EPI_ISL_17595302, EPI_ISL_17595303	Quest Diagnostics Nichols Institute	Los Angeles County Public Health Laboratories	P. Hemarajata et al.
EPI_ISL_17595304, EPI_ISL_17595305, EPI_ISL_17595306, EPI_ISL_17595307, EPI_ISL_17595308	Kaiser Permanente Chino Hills Regional Reference Laboratories	Los Angeles County Public Health Laboratories	P. Hemarajata et al.
EPI_ISL_17614017, EPI_ISL_17614018, EPI_ISL_17614019, EPI_ISL_17614020, EPI_ISL_17614021, EPI_ISL_17614022, EPI_ISL_17614023, EPI_ISL_17614024, EPI_ISL_17614025, EPI_ISL_17614026, EPI_ISL_17614027, EPI_ISL_17614028, EPI_ISL_17614029, EPI_ISL_17614030, EPI_ISL_17614031, EPI_ISL_17614032, EPI_ISL_17614033, EPI_ISL_17614034, EPI_ISL_17614035, EPI_ISL_17614036, EPI_ISL_17614037, EPI_ISL_17614038, EPI_ISL_17614039, EPI_ISL_17614040, EPI_ISL_17614041, EPI_ISL_17614042, EPI_ISL_17614043, EPI_ISL_17614044, EPI_ISL_17614045, EPI_ISL_17614046, EPI_ISL_17614047, EPI_ISL_17614048, EPI_ISL_17614049			
see above	Laboratorio de Enterovirus, Instituto Oswaldo Cruz, Fiocruz	Instituto Oswaldo Cruz FIOCRUZ - Laboratory of Respiratory Viruses and Measles (LVRS)	Paola Resende, Elisa Cavalcante Pereira, Bruna Mendonça da Silva, Jéssica Graça Macedo de Carvalho, Larissa Macedo Pinto, Victor Guimaraes, Marilda Siqueira, Renan da Silva Faustino, Marilia Santini, Edson Elias da Silva on behalf of the Fiocruz Genomic Surveillance Network
EPI_ISL_17665624, EPI_ISL_17665625, EPI_ISL_17665626, EPI_ISL_17665627	Tokyo Metropolitan Institute of Public Health	Tokyo Metropolitan Institute of Public Health	Fumi Kasuya, Wakaba Okada, Ryota Kumagai, Sachiko Harada, Arisa Amano, Michiya Hasegawa, Mami Nagashima, Kenji Sadamasu
EPI_ISL_17672206	LESP State of Mexico	Instituto de Diagnostico y Referencia Epidemiologicos (INDRE)	Abril Rodríguez-Maldonado; Claudia Wong-Arámbula; Silvia Rivero-Arredondo; Ruth Madera-Sandoval; Joaquín Quiroz-Mercado; Fernando González-Domínguez; Lucia Hernández-Rivas, Irma López-Martínez; Ernesto Ramírez-González; Maribel González-Villa
EPI_ISL_17672207	LESP Jalisco	Instituto de Diagnostico y Referencia Epidemiologicos (INDRE)	Abril Rodríguez-Maldonado; Claudia Wong-Arámbula; Silvia Rivero-Arredondo; Ruth Madera-Sandoval; Joaquín Quiroz-Mercado; Fernando González-Domínguez; Lucia Hernández-Rivas, Irma López-Martínez; Ernesto Ramírez-González; Maribel González-Villa
EPI_ISL_17672208	LESP Queretaro	Instituto de Diagnostico y Referencia Epidemiologicos (INDRE)	Abril Rodríguez-Maldonado; Claudia Wong-Arámbula; Silvia Rivero-Arredondo; Ruth Madera-Sandoval; Joaquín Quiroz-Mercado; Fernando González-Domínguez; Lucia Hernández-Rivas, Irma López-Martínez; Ernesto Ramírez-González; Maribel González-Villa
EPI_ISL_17672209	LESP Yucatan	Instituto de Diagnostico y Referencia Epidemiologicos (INDRE)	Abril Rodríguez-Maldonado; Claudia Wong-Arámbula; Silvia Rivero-Arredondo; Ruth Madera-Sandoval; Joaquín Quiroz-Mercado; Fernando González-Domínguez; Lucia Hernández-Rivas, Irma López-Martínez; Ernesto Ramírez-González; Maribel González-Villa
EPI_ISL_17672210	LESP Quintana Roo	Instituto de Diagnostico y Referencia Epidemiologicos (INDRE)	Abril Rodríguez-Maldonado; Claudia Wong-Arámbula; Silvia Rivero-Arredondo; Ruth Madera-Sandoval; Joaquín Quiroz-Mercado; Fernando González-Domínguez; Lucia Hernández-Rivas, Irma López-Martínez; Ernesto Ramírez-González; Maribel González-Villa
EPI_ISL_17672211	LESP Mexico City	Instituto de Diagnostico y Referencia Epidemiologicos (INDRE)	Abril Rodríguez-Maldonado; Claudia Wong-Arámbula; Silvia Rivero-Arredondo; Ruth Madera-Sandoval; Joaquín Quiroz-Mercado; Fernando González-Domínguez; Lucia Hernández-Rivas, Irma López-Martínez; Ernesto Ramírez-González; Maribel González-Villa
EPI_ISL_17672212	LESP Tamaulipas	Instituto de Diagnostico y Referencia Epidemiologicos (INDRE)	Abril Rodríguez-Maldonado; Claudia Wong-Arámbula; Silvia Rivero-Arredondo; Ruth Madera-Sandoval; Joaquín Quiroz-Mercado; Fernando González-Domínguez; Lucia Hernández-Rivas, Irma López-Martínez; Ernesto Ramírez-González; Maribel González-Villa
EPI_ISL_17672213	LESP Puebla	Instituto de Diagnostico y Referencia Epidemiologicos (INDRE)	Abril Rodríguez-Maldonado; Claudia Wong-Arámbula; Silvia Rivero-Arredondo; Ruth Madera-Sandoval; Joaquín Quiroz-Mercado; Fernando González-Domínguez; Lucia Hernández-Rivas, Irma López-Martínez; Ernesto Ramírez-González; Maribel González-Villa
EPI_ISL_17672214	LESP Guerrero	Instituto de Diagnostico y Referencia Epidemiologicos (INDRE)	Abril Rodríguez-Maldonado; Claudia Wong-Arámbula; Silvia Rivero-Arredondo; Ruth Madera-Sandoval; Joaquín Quiroz-Mercado; Fernando González-Domínguez; Lucia Hernández-Rivas, Irma López-Martínez; Ernesto Ramírez-González; Maribel González-Villa
EPI_ISL_17672215	LESP Oaxaca	Instituto de Diagnostico y Referencia Epidemiologicos (INDRE)	Abril Rodríguez-Maldonado; Claudia Wong-Arámbula; Silvia Rivero-Arredondo; Ruth Madera-Sandoval; Joaquín Quiroz-Mercado; Fernando González-Domínguez; Lucia Hernández-Rivas, Irma López-Martínez; Ernesto Ramírez-González; Maribel González-Villa
EPI_ISL_17672216	LESP Hidalgo	Instituto de Diagnostico y Referencia Epidemiologicos (INDRE)	Abril Rodríguez-Maldonado; Claudia Wong-Arámbula; Silvia Rivero-Arredondo; Ruth Madera-Sandoval; Joaquín Quiroz-Mercado; Fernando González-Domínguez; Lucia Hernández-Rivas, Irma López-Martínez; Ernesto Ramírez-González; Maribel González-Villa
EPI_ISL_17672217	LESP Zacatecas	Instituto de Diagnostico y Referencia Epidemiologicos (INDRE)	Abril Rodríguez-Maldonado; Claudia Wong-Arámbula; Silvia Rivero-Arredondo; Ruth Madera-Sandoval; Joaquín Quiroz-Mercado; Fernando González-Domínguez; Lucia Hernández-Rivas, Irma López-Martínez; Ernesto Ramírez-González; Maribel González-Villa
EPI_ISL_17672218	LESP Tabasco	Instituto de Diagnostico y Referencia Epidemiologicos (INDRE)	Abril Rodríguez-Maldonado; Claudia Wong-Arámbula; Silvia Rivero-Arredondo; Ruth Madera-Sandoval; Joaquín Quiroz-Mercado; Fernando González-Domínguez; Lucia Hernández-Rivas, Irma López-Martínez; Ernesto Ramírez-González; Maribel González-Villa
EPI_ISL_17672219	LESP Aguascalientes	Instituto de Diagnostico y Referencia Epidemiologicos (INDRE)	Abril Rodríguez-Maldonado; Claudia Wong-Arámbula; Silvia Rivero-Arredondo; Ruth Madera-Sandoval; Joaquín Quiroz-Mercado; Fernando González-Domínguez; Lucia Hernández-Rivas, Irma López-Martínez; Ernesto Ramírez-González; Maribel González-Villa
EPI_ISL_17672220	LESP Tlaxcala	Instituto de Diagnostico y Referencia Epidemiologicos (INDRE)	Abril Rodríguez-Maldonado; Claudia Wong-Arámbula; Silvia Rivero-Arredondo; Ruth Madera-Sandoval; Joaquín Quiroz-Mercado; Fernando González-Domínguez; Lucia Hernández-Rivas, Irma López-Martínez; Ernesto Ramírez-González; Maribel González-Villa
EPI_ISL_17672221	LESP Chihuahua	Instituto de Diagnostico y Referencia Epidemiologicos (INDRE)	Abril Rodríguez-Maldonado; Claudia Wong-Arámbula; Silvia Rivero-Arredondo; Ruth Madera-Sandoval; Joaquín Quiroz-Mercado; Fernando González-Domínguez; Lucia Hernández-Rivas, Irma López-Martínez; Ernesto Ramírez-González; Maribel González-Villa
EPI_ISL_17672222	LESP Veracruz	Instituto de Diagnostico y Referencia Epidemiologicos (INDRE)	Abril Rodríguez-Maldonado; Claudia Wong-Arámbula; Silvia Rivero-Arredondo; Ruth Madera-Sandoval; Joaquín Quiroz-Mercado; Fernando González-Domínguez; Lucia Hernández-Rivas, Irma López-Martínez; Ernesto Ramírez-González; Maribel González-Villa
EPI_ISL_17672223	LESP Baja California	Instituto de Diagnostico y Referencia Epidemiologicos (INDRE)	Abril Rodríguez-Maldonado; Claudia Wong-Arámbula; Silvia Rivero-Arredondo; Ruth Madera-Sandoval; Joaquín Quiroz-Mercado; Fernando González-Domínguez; Lucia Hernández-Rivas, Irma López-Martínez; Ernesto Ramírez-González; Maribel González-Villa
EPI_ISL_17672224	LESP Tamaulipas	Instituto de Diagnostico y Referencia Epidemiologicos (INDRE)	Abril Rodríguez-Maldonado; Claudia Wong-Arámbula; Silvia Rivero-Arredondo; Ruth Madera-Sandoval; Joaquín Quiroz-Mercado; Fernando González-Domínguez; Lucia Hernández-Rivas, Irma López-Martínez; Ernesto Ramírez-González; Maribel González-Villa
EPI_ISL_17672225	LESP Campeche	Instituto de Diagnostico y Referencia Epidemiologicos (INDRE)	Abril Rodríguez-Maldonado; Claudia Wong-Arámbula; Silvia Rivero-Arredondo; Ruth Madera-Sandoval; Joaquín Quiroz-Mercado; Fernando González-Domínguez; Lucia Hernández-Rivas, Irma López-Martínez; Ernesto Ramírez-González; Maribel González-Villa
EPI_ISL_17672226	LESP Hidalgo	Instituto de Diagnostico y Referencia Epidemiologicos (INDRE)	Abril Rodríguez-Maldonado; Claudia Wong-Arámbula; Silvia Rivero-Arredondo; Ruth Madera-Sandoval; Joaquín Quiroz-Mercado; Fernando González-Domínguez; Lucia Hernández-Rivas, Irma López-Martínez; Ernesto Ramírez-González; Maribel González-Villa
EPI_ISL_17672227	LESP Mexico City	Instituto de Diagnostico y Referencia Epidemiologicos (INDRE)	Abril Rodríguez-Maldonado; Claudia Wong-Arámbula; Silvia Rivero-Arredondo; Ruth Madera-Sandoval; Joaquín Quiroz-Mercado; Fernando González-Domínguez; Lucia Hernández-Rivas, Irma López-Martínez; Ernesto Ramírez-González; Maribel González-Villa
EPI_ISL_17672228	LESP Guerrero	Instituto de Diagnostico y Referencia Epidemiologicos (INDRE)	Abril Rodríguez-Maldonado; Claudia Wong-Arámbula; Silvia Rivero-Arredondo; Ruth Madera-Sandoval; Joaquín Quiroz-Mercado; Fernando González-Domínguez; Lucia Hernández-Rivas, Irma López-Martínez; Ernesto Ramírez-González; Maribel González-Villa
EPI_ISL_17672229	LESP Coahuila	Instituto de Diagnostico y Referencia Epidemiologicos (INDRE)	Abril Rodríguez-Maldonado; Claudia Wong-Arámbula; Silvia Rivero-Arredondo; Ruth Madera-Sandoval; Joaquín Quiroz-Mercado; Fernando González-Domínguez; Lucia Hernández-Rivas, Irma López-Martínez; Ernesto Ramírez-González; Maribel González-Villa
EPI_ISL_17672230	LESP Tabasco	Instituto de Diagnostico y Referencia Epidemiologicos (INDRE)	Abril Rodríguez-Maldonado; Claudia Wong-Arámbula; Silvia Rivero-Arredondo; Ruth Madera-Sandoval; Joaquín Quiroz-Mercado; Fernando González-Domínguez; Lucia Hernández-Rivas, Irma López-Martínez; Ernesto Ramírez-González; Maribel González-Villa
EPI_ISL_17672231	LESP Chiapas	Instituto de Diagnostico y Referencia Epidemiologicos (INDRE)	Abril Rodríguez-Maldonado; Claudia Wong-Arámbula; Silvia Rivero-Arredondo; Ruth Madera-Sandoval; Joaquín Quiroz-Mercado; Fernando González-Domínguez; Lucia Hernández-Rivas, Irma López-Martínez; Ernesto Ramírez-González; Maribel González-Villa
EPI_ISL_17672232	LESP Yucatan	Instituto de Diagnostico y Referencia Epidemiologicos	Abril Rodríguez-Maldonado; Claudia Wong-Arámbula; Silvia Rivero-Arredondo; Ruth Madera-Sandoval; Joaquín Quiroz-Mercado; Fernando González-Domínguez; Lucia Hernández-Rivas, Irma López-Martínez; Ernesto

		(INDRE)	Ramírez-González; Maribel González-Villa
EPI_ISL_17672233	LESP Quintana Roo	Instituto de Diagnostico y Referencia Epidemiologicos (INDRE)	Abril Rodríguez-Maldonado; Claudia Wong-Arámbula; Silvia Rivero-Arredondo; Ruth Madera-Sandoval; Joaquín Quiroz-Mercado; Fernando González-Domínguez; Lucia Hernández-Rivas, Irma López-Martínez; Ernesto Ramírez-González; Maribel González-Villa
EPI_ISL_17672234	LESP Jalisco	Instituto de Diagnostico y Referencia Epidemiologicos (INDRE)	Abril Rodríguez-Maldonado; Claudia Wong-Arámbula; Silvia Rivero-Arredondo; Ruth Madera-Sandoval; Joaquín Quiroz-Mercado; Fernando González-Domínguez; Lucia Hernández-Rivas, Irma López-Martínez; Ernesto Ramírez-González; Maribel González-Villa
EPI_ISL_17672235	LESP State of Mexico	Instituto de Diagnostico y Referencia Epidemiologicos (INDRE)	Abril Rodríguez-Maldonado; Claudia Wong-Arámbula; Silvia Rivero-Arredondo; Ruth Madera-Sandoval; Joaquín Quiroz-Mercado; Fernando González-Domínguez; Lucia Hernández-Rivas, Irma López-Martínez; Ernesto Ramírez-González; Maribel González-Villa
EPI_ISL_17672236	Laboratorio Estatal de Salud Pública Puebla	Instituto de Diagnostico y Referencia Epidemiologicos (INDRE)	Abril Rodríguez-Maldonado; Claudia Wong-Arámbula; Silvia Rivero-Arredondo; Ruth Madera-Sandoval; Joaquín Quiroz-Mercado; Fernando González-Domínguez; Lucia Hernández-Rivas, Irma López-Martínez; Ernesto Ramírez-González; Maribel González-Villa
EPI_ISL_17672237	LESP Campeche	Instituto de Diagnostico y Referencia Epidemiologicos (INDRE)	Abril Rodríguez-Maldonado; Claudia Wong-Arámbula; Silvia Rivero-Arredondo; Ruth Madera-Sandoval; Joaquín Quiroz-Mercado; Fernando González-Domínguez; Lucia Hernández-Rivas, Irma López-Martínez; Ernesto Ramírez-González; Maribel González-Villa
EPI_ISL_17672238	LESP State of Mexico	Instituto de Diagnostico y Referencia Epidemiologicos (INDRE)	Abril Rodríguez-Maldonado; Claudia Wong-Arámbula; Silvia Rivero-Arredondo; Ruth Madera-Sandoval; Joaquín Quiroz-Mercado; Fernando González-Domínguez; Lucia Hernández-Rivas, Irma López-Martínez; Ernesto Ramírez-González; Maribel González-Villa
EPI_ISL_17672239	LESP Chiapas	Instituto de Diagnostico y Referencia Epidemiologicos (INDRE)	Abril Rodríguez-Maldonado; Claudia Wong-Arámbula; Silvia Rivero-Arredondo; Ruth Madera-Sandoval; Joaquín Quiroz-Mercado; Fernando González-Domínguez; Lucia Hernández-Rivas, Irma López-Martínez; Ernesto Ramírez-González; Maribel González-Villa
EPI_ISL_17672240	LESP Coahuila	Instituto de Diagnostico y Referencia Epidemiologicos (INDRE)	Abril Rodríguez-Maldonado; Claudia Wong-Arámbula; Silvia Rivero-Arredondo; Ruth Madera-Sandoval; Joaquín Quiroz-Mercado; Fernando González-Domínguez; Lucia Hernández-Rivas, Irma López-Martínez; Ernesto Ramírez-González; Maribel González-Villa
EPI_ISL_17672241	LESP Jalisco	Instituto de Diagnostico y Referencia Epidemiologicos (INDRE)	Abril Rodríguez-Maldonado; Claudia Wong-Arámbula; Silvia Rivero-Arredondo; Ruth Madera-Sandoval; Joaquín Quiroz-Mercado; Fernando González-Domínguez; Lucia Hernández-Rivas, Irma López-Martínez; Ernesto Ramírez-González; Maribel González-Villa
EPI_ISL_17672242	LESP Nuevo Leon	Instituto de Diagnostico y Referencia Epidemiologicos (INDRE)	Abril Rodríguez-Maldonado; Claudia Wong-Arámbula; Silvia Rivero-Arredondo; Ruth Madera-Sandoval; Joaquín Quiroz-Mercado; Fernando González-Domínguez; Lucia Hernández-Rivas, Irma López-Martínez; Ernesto Ramírez-González; Maribel González-Villa
EPI_ISL_17672243	LESP Queretaro	Instituto de Diagnostico y Referencia Epidemiologicos (INDRE)	Abril Rodríguez-Maldonado; Claudia Wong-Arámbula; Silvia Rivero-Arredondo; Ruth Madera-Sandoval; Joaquín Quiroz-Mercado; Fernando González-Domínguez; Lucia Hernández-Rivas, Irma López-Martínez; Ernesto Ramírez-González; Maribel González-Villa
EPI_ISL_17672244	LESP Hidalgo	Instituto de Diagnostico y Referencia Epidemiologicos (INDRE)	Abril Rodríguez-Maldonado; Claudia Wong-Arámbula; Silvia Rivero-Arredondo; Ruth Madera-Sandoval; Joaquín Quiroz-Mercado; Fernando González-Domínguez; Lucia Hernández-Rivas, Irma López-Martínez; Ernesto Ramírez-González; Maribel González-Villa
EPI_ISL_17672245	LESP Tamaulipas	Instituto de Diagnostico y Referencia Epidemiologicos (INDRE)	Abril Rodríguez-Maldonado; Claudia Wong-Arámbula; Silvia Rivero-Arredondo; Ruth Madera-Sandoval; Joaquín Quiroz-Mercado; Fernando González-Domínguez; Lucia Hernández-Rivas, Irma López-Martínez; Ernesto Ramírez-González; Maribel González-Villa
EPI_ISL_17672246	LESP Veracruz	Instituto de Diagnostico y Referencia Epidemiologicos (INDRE)	Abril Rodríguez-Maldonado; Claudia Wong-Arámbula; Silvia Rivero-Arredondo; Ruth Madera-Sandoval; Joaquín Quiroz-Mercado; Fernando González-Domínguez; Lucia Hernández-Rivas, Irma López-Martínez; Ernesto Ramírez-González; Maribel González-Villa
EPI_ISL_17672247	LESP Quintana Roo	Instituto de Diagnostico y Referencia Epidemiologicos (INDRE)	Abril Rodríguez-Maldonado; Claudia Wong-Arámbula; Silvia Rivero-Arredondo; Ruth Madera-Sandoval; Joaquín Quiroz-Mercado; Fernando González-Domínguez; Lucia Hernández-Rivas, Irma López-Martínez; Ernesto Ramírez-González; Maribel González-Villa
EPI_ISL_17672248	LESP Queretaro	Instituto de Diagnostico y Referencia Epidemiologicos (INDRE)	Abril Rodríguez-Maldonado; Claudia Wong-Arámbula; Silvia Rivero-Arredondo; Ruth Madera-Sandoval; Joaquín Quiroz-Mercado; Fernando González-Domínguez; Lucia Hernández-Rivas, Irma López-Martínez; Ernesto Ramírez-González; Maribel González-Villa
EPI_ISL_17672249	LESP Oaxaca	Instituto de Diagnostico y Referencia Epidemiologicos (INDRE)	Abril Rodríguez-Maldonado; Claudia Wong-Arámbula; Silvia Rivero-Arredondo; Ruth Madera-Sandoval; Joaquín Quiroz-Mercado; Fernando González-Domínguez; Lucia Hernández-Rivas, Irma López-Martínez; Ernesto Ramírez-González; Maribel González-Villa
EPI_ISL_17672250	LESP Yucatan	Instituto de Diagnostico y Referencia Epidemiologicos (INDRE)	Abril Rodríguez-Maldonado; Claudia Wong-Arámbula; Silvia Rivero-Arredondo; Ruth Madera-Sandoval; Joaquín Quiroz-Mercado; Fernando González-Domínguez; Lucia Hernández-Rivas, Irma López-Martínez; Ernesto Ramírez-González; Maribel González-Villa
EPI_ISL_17672251	LESP Guanajuato	Instituto de Diagnostico y Referencia Epidemiologicos (INDRE)	Abril Rodríguez-Maldonado; Claudia Wong-Arámbula; Silvia Rivero-Arredondo; Ruth Madera-Sandoval; Joaquín Quiroz-Mercado; Fernando González-Domínguez; Lucia Hernández-Rivas, Irma López-Martínez; Ernesto Ramírez-González; Maribel González-Villa
EPI_ISL_17672252	LESP Veracruz	Instituto de Diagnostico y Referencia Epidemiologicos (INDRE)	Abril Rodríguez-Maldonado; Claudia Wong-Arámbula; Silvia Rivero-Arredondo; Ruth Madera-Sandoval; Joaquín Quiroz-Mercado; Fernando González-Domínguez; Lucia Hernández-Rivas, Irma López-Martínez; Ernesto Ramírez-González; Maribel González-Villa
EPI_ISL_17672253	LESP Mexico City	Instituto de Diagnostico y Referencia Epidemiologicos (INDRE)	Abril Rodríguez-Maldonado; Claudia Wong-Arámbula; Silvia Rivero-Arredondo; Ruth Madera-Sandoval; Joaquín Quiroz-Mercado; Fernando González-Domínguez; Lucia Hernández-Rivas, Irma López-Martínez; Ernesto Ramírez-González; Maribel González-Villa
EPI_ISL_17672254	LESP Durango	Instituto de Diagnostico y Referencia Epidemiologicos (INDRE)	Abril Rodríguez-Maldonado; Claudia Wong-Arámbula; Silvia Rivero-Arredondo; Ruth Madera-Sandoval; Joaquín Quiroz-Mercado; Fernando González-Domínguez; Lucia Hernández-Rivas, Irma López-Martínez; Ernesto Ramírez-González; Maribel González-Villa
EPI_ISL_17672255	LESP San Luis Potosi	Instituto de Diagnostico y Referencia Epidemiologicos (INDRE)	Abril Rodríguez-Maldonado; Claudia Wong-Arámbula; Silvia Rivero-Arredondo; Ruth Madera-Sandoval; Joaquín Quiroz-Mercado; Fernando González-Domínguez; Lucia Hernández-Rivas, Irma López-Martínez; Ernesto Ramírez-González; Maribel González-Villa
EPI_ISL_17672256	LESP Nuevo Leon	Instituto de Diagnostico y Referencia Epidemiologicos (INDRE)	Abril Rodríguez-Maldonado; Claudia Wong-Arámbula; Silvia Rivero-Arredondo; Ruth Madera-Sandoval; Joaquín Quiroz-Mercado; Fernando González-Domínguez; Lucia Hernández-Rivas, Irma López-Martínez; Ernesto Ramírez-González; Maribel González-Villa
EPI_ISL_17672257, EPI_ISL_17672258	LESP Quintana Roo	Instituto de Diagnostico y Referencia Epidemiologicos (INDRE)	Abril Rodríguez-Maldonado; Claudia Wong-Arámbula; Silvia Rivero-Arredondo; Ruth Madera-Sandoval; Joaquín Quiroz-Mercado; Fernando González-Domínguez; Lucia Hernández-Rivas, Irma López-Martínez; Ernesto Ramírez-González; Maribel González-Villa
EPI_ISL_17672259	LESP Mexico City	Instituto de Diagnostico y Referencia Epidemiologicos (INDRE)	Abril Rodríguez-Maldonado; Claudia Wong-Arámbula; Silvia Rivero-Arredondo; Ruth Madera-Sandoval; Joaquín Quiroz-Mercado; Fernando González-Domínguez; Lucia Hernández-Rivas, Irma López-Martínez; Ernesto Ramírez-González; Maribel González-Villa
EPI_ISL_17672260	LESP Veracruz	Instituto de Diagnostico y Referencia Epidemiologicos (INDRE)	Abril Rodríguez-Maldonado; Claudia Wong-Arámbula; Silvia Rivero-Arredondo; Ruth Madera-Sandoval; Joaquín Quiroz-Mercado; Fernando González-Domínguez; Lucia Hernández-Rivas, Irma López-Martínez; Ernesto Ramírez-González; Maribel González-Villa
EPI_ISL_17692268, EPI_ISL_17692269	Tokyo Metropolitan Institute of Public Health	Tokyo Metropolitan Institute of Public Health	Fumi Kasuya, Wakaba Okada, Ryota Kumagai, Sachiko Harada, Arisa Amano, Michiya Hasegawa, Mami Nagashima, Kenji Sadamasu
EPI_ISL_17703564, EPI_ISL_17703565	LESP State of Mexico	Instituto de Diagnostico y Referencia Epidemiologicos (INDRE)	Abril Rodríguez-Maldonado; Claudia Wong-Arámbula; Felipe Arguijo-Perez; Helios Cárdenas-Hernández; Carmen Castro-Méndez; Lidia García-Torres; Ruth Madera-Sandoval; América Mandujano-Martínez; Nancy Martínez-Velázquez; Mireya Mederos-Michel; Angélica Pedraza-Meléndez; Joaquín Quiroz-Mercado; Daniel Regalado-Santiago; Silvia Rivero-Arredondo; Erika Sierra-Atanacio; Fernando González-Domínguez; Lucia Hernández-Rivas, Irma López-Martínez; Ernesto Ramírez-González; Maribel González-Villa
EPI_ISL_17703566	LESP Jalisco	Instituto de Diagnostico y Referencia Epidemiologicos (INDRE)	Abril Rodríguez-Maldonado; Claudia Wong-Arámbula; Felipe Arguijo-Perez; Helios Cárdenas-Hernández; Carmen Castro-Méndez; Lidia García-Torres; Ruth Madera-Sandoval; América Mandujano-Martínez; Nancy Martínez-Velázquez; Mireya Mederos-Michel; Angélica Pedraza-Meléndez; Joaquín Quiroz-Mercado; Daniel Regalado-Santiago; Silvia Rivero-Arredondo; Erika Sierra-Atanacio; Fernando González-Domínguez; Lucia Hernández-Rivas, Irma López-Martínez; Ernesto Ramírez-González; Maribel González-Villa
EPI_ISL_17703567	LESP State of Mexico	Instituto de Diagnostico y Referencia Epidemiologicos (INDRE)	Abril Rodríguez-Maldonado; Claudia Wong-Arámbula; Felipe Arguijo-Perez; Helios Cárdenas-Hernández; Carmen Castro-Méndez; Lidia García-Torres; Ruth Madera-Sandoval; América Mandujano-Martínez; Nancy Martínez-Velázquez; Mireya Mederos-Michel; Angélica Pedraza-Meléndez; Joaquín Quiroz-Mercado; Daniel Regalado-Santiago; Silvia Rivero-Arredondo; Erika Sierra-Atanacio; Fernando González-Domínguez; Lucia Hernández-Rivas, Irma López-Martínez; Ernesto Ramírez-González; Maribel González-Villa
EPI_ISL_17703568	LESP Quintana Roo	Instituto de Diagnostico y Referencia Epidemiologicos (INDRE)	Abril Rodríguez-Maldonado; Claudia Wong-Arámbula; Felipe Arguijo-Perez; Helios Cárdenas-Hernández; Carmen Castro-Méndez; Lidia García-Torres; Ruth Madera-Sandoval; América Mandujano-Martínez; Nancy Martínez-Velázquez; Mireya Mederos-Michel; Angélica Pedraza-Meléndez; Joaquín Quiroz-Mercado; Daniel Regalado-Santiago; Silvia Rivero-Arredondo; Erika Sierra-Atanacio; Fernando González-Domínguez; Lucia Hernández-Rivas, Irma López-Martínez; Ernesto Ramírez-González; Maribel González-Villa
EPI_ISL_17703569, EPI_ISL_17703570	LESP Mexico City	Instituto de Diagnostico y Referencia Epidemiologicos (INDRE)	Abril Rodríguez-Maldonado; Claudia Wong-Arámbula; Felipe Arguijo-Perez; Helios Cárdenas-Hernández; Carmen Castro-Méndez; Lidia García-Torres; Ruth Madera-Sandoval; América Mandujano-Martínez; Nancy Martínez-Velázquez; Mireya Mederos-Michel; Angélica Pedraza-Meléndez; Joaquín Quiroz-Mercado; Daniel Regalado-Santiago; Silvia Rivero-Arredondo; Erika Sierra-Atanacio; Fernando González-Domínguez; Lucia Hernández-Rivas, Irma López-Martínez; Ernesto Ramírez-González; Maribel González-Villa
EPI_ISL_17703571	LESP Sinaloa	Instituto de Diagnostico y Referencia Epidemiologicos (INDRE)	Abril Rodríguez-Maldonado; Claudia Wong-Arámbula; Felipe Arguijo-Perez; Helios Cárdenas-Hernández; Carmen Castro-Méndez; Lidia García-Torres; Ruth Madera-Sandoval; América Mandujano-Martínez; Nancy Martínez-Velázquez; Mireya Mederos-Michel; Angélica Pedraza-Meléndez; Joaquín Quiroz-Mercado; Daniel Regalado-Santiago; Silvia Rivero-Arredondo; Erika Sierra-Atanacio; Fernando González-Domínguez; Lucia Hernández-Rivas, Irma López-Martínez; Ernesto Ramírez-González; Maribel González-Villa
EPI_ISL_17703572	LESP State of Mexico	Instituto de Diagnostico y Referencia Epidemiologicos (INDRE)	Abril Rodríguez-Maldonado; Claudia Wong-Arámbula; Felipe Arguijo-Perez; Helios Cárdenas-Hernández; Carmen Castro-Méndez; Lidia García-Torres; Ruth Madera-Sandoval; América Mandujano-Martínez; Nancy Martínez-Velázquez; Mireya Mederos-Michel; Angélica Pedraza-Meléndez; Joaquín Quiroz-Mercado; Daniel Regalado-Santiago; Silvia Rivero-Arredondo; Erika Sierra-Atanacio; Fernando González-Domínguez; Lucia Hernández-Rivas, Irma López-Martínez; Ernesto Ramírez-González; Maribel González-Villa
EPI_ISL_17703573	LESP Mexico City	Instituto de Diagnostico y Referencia Epidemiologicos (INDRE)	Abril Rodríguez-Maldonado; Claudia Wong-Arámbula; Felipe Arguijo-Perez; Helios Cárdenas-Hernández; Carmen Castro-Méndez; Lidia García-Torres; Ruth Madera-Sandoval; América Mandujano-Martínez; Nancy Martínez-Velázquez; Mireya Mederos-Michel; Angélica Pedraza-Meléndez; Joaquín Quiroz-Mercado; Daniel Regalado-Santiago; Silvia Rivero-Arredondo; Erika Sierra-Atanacio; Fernando González-Domínguez; Lucia Hernández-Rivas, Irma López-Martínez; Ernesto Ramírez-González; Maribel González-Villa
EPI_ISL_17703574	LESP State of Mexico	Instituto de Diagnostico y Referencia Epidemiologicos (INDRE)	Abril Rodríguez-Maldonado; Claudia Wong-Arámbula; Felipe Arguijo-Perez; Helios Cárdenas-Hernández; Carmen Castro-Méndez; Lidia García-Torres; Ruth Madera-Sandoval; América Mandujano-Martínez; Nancy Martínez-Velázquez; Mireya Mederos-Michel; Angélica Pedraza-Meléndez; Joaquín Quiroz-Mercado; Daniel Regalado-Santiago; Silvia Rivero-Arredondo; Erika Sierra-Atanacio; Fernando González-Domínguez; Lucia Hernández-Rivas, Irma López-Martínez; Ernesto Ramírez-González; Maribel González-Villa
EPI_ISL_17703575	LESP Mexico City	Instituto de Diagnostico y Referencia Epidemiologicos (INDRE)	Abril Rodríguez-Maldonado; Claudia Wong-Arámbula; Felipe Arguijo-Perez; Helios Cárdenas-Hernández; Carmen Castro-Méndez; Lidia García-Torres; Ruth Madera-Sandoval; América Mandujano-Martínez; Nancy Martínez-Velázquez; Mireya Mederos-Michel; Angélica Pedraza-Meléndez; Joaquín Quiroz-Mercado; Daniel Regalado-Santiago; Silvia Rivero-Arredondo; Erika Sierra-Atanacio; Fernando González-Domínguez; Lucia Hernández-Rivas, Irma López-Martínez; Ernesto Ramírez-González; Maribel González-Villa
EPI_ISL_17703576	LESP Quintana Roo	Instituto de Diagnostico y Referencia Epidemiologicos (INDRE)	Abril Rodríguez-Maldonado; Claudia Wong-Arámbula; Felipe Arguijo-Perez; Helios Cárdenas-Hernández; Carmen Castro-Méndez; Lidia García-Torres; Ruth Madera-Sandoval; América Mandujano-Martínez; Nancy Martínez-Velázquez; Mireya Mederos-Michel; Angélica Pedraza-Meléndez; Joaquín Quiroz-Mercado; Daniel Regalado-Santiago; Silvia Rivero-Arredondo; Erika Sierra-Atanacio; Fernando González-Domínguez; Lucia Hernández-Rivas, Irma López-Martínez; Ernesto Ramírez-González; Maribel González-Villa
EPI_ISL_17703577	LESP Mexico City	Instituto de Diagnostico y Referencia Epidemiologicos (INDRE)	Abril Rodríguez-Maldonado; Claudia Wong-Arámbula; Felipe Arguijo-Perez; Helios Cárdenas-Hernández; Carmen Castro-Méndez; Lidia García-Torres; Ruth Madera-Sandoval; América Mandujano-Martínez; Nancy Martínez-Velázquez; Mireya Mederos-Michel; Angélica Pedraza-Meléndez; Joaquín Quiroz-Mercado; Daniel Regalado-Santiago; Silvia Rivero-Arredondo; Erika Sierra-Atanacio; Fernando González-Domínguez; Lucia Hernández-Rivas, Irma López-Martínez; Ernesto Ramírez-González; Maribel González-Villa

EPI_ISL_17703578	LESP Yucatan	Instituto de Diagnostico y Referencia Epidemiologicos (INDRE)	Abril Rodríguez-Maldonado; Claudia Wong-Arámbula; Felipe Arguijo-Perez; Helios Cárdenas-Hernández; Carmen Castro-Méndez; Lidia García-Torres; Ruth Madera-Sandoval; América Mandujano-Martínez; Nancy Martínez-Velázquez; Mireya Mederos-Michel; Angélica Pedraza-Meléndez; Joaquín Quiroz-Mercado; Daniel Regalado-Santiago; Silvia Rivero-Arredondo; Erika Sierra-Atanacio; Fernando González-Domínguez; Lucia Hernández-Rivas, Irma López-Martínez; Ernesto Ramírez-González; Maribel González-Villa
EPI_ISL_17703579	LESP Quintana Roo	Instituto de Diagnostico y Referencia Epidemiologicos (INDRE)	Abril Rodríguez-Maldonado; Claudia Wong-Arámbula; Felipe Arguijo-Perez; Helios Cárdenas-Hernández; Carmen Castro-Méndez; Lidia García-Torres; Ruth Madera-Sandoval; América Mandujano-Martínez; Nancy Martínez-Velázquez; Mireya Mederos-Michel; Angélica Pedraza-Meléndez; Joaquín Quiroz-Mercado; Daniel Regalado-Santiago; Silvia Rivero-Arredondo; Erika Sierra-Atanacio; Fernando González-Domínguez; Lucia Hernández-Rivas, Irma López-Martínez; Ernesto Ramírez-González; Maribel González-Villa
EPI_ISL_17703580	LESP Hidalgo	Instituto de Diagnostico y Referencia Epidemiologicos (INDRE)	Abril Rodríguez-Maldonado; Claudia Wong-Arámbula; Felipe Arguijo-Perez; Helios Cárdenas-Hernández; Carmen Castro-Méndez; Lidia García-Torres; Ruth Madera-Sandoval; América Mandujano-Martínez; Nancy Martínez-Velázquez; Mireya Mederos-Michel; Angélica Pedraza-Meléndez; Joaquín Quiroz-Mercado; Daniel Regalado-Santiago; Silvia Rivero-Arredondo; Erika Sierra-Atanacio; Fernando González-Domínguez; Lucia Hernández-Rivas, Irma López-Martínez; Ernesto Ramírez-González; Maribel González-Villa
EPI_ISL_17703581	LESP Yucatan	Instituto de Diagnostico y Referencia Epidemiologicos (INDRE)	Abril Rodríguez-Maldonado; Claudia Wong-Arámbula; Felipe Arguijo-Perez; Helios Cárdenas-Hernández; Carmen Castro-Méndez; Lidia García-Torres; Ruth Madera-Sandoval; América Mandujano-Martínez; Nancy Martínez-Velázquez; Mireya Mederos-Michel; Angélica Pedraza-Meléndez; Joaquín Quiroz-Mercado; Daniel Regalado-Santiago; Silvia Rivero-Arredondo; Erika Sierra-Atanacio; Fernando González-Domínguez; Lucia Hernández-Rivas, Irma López-Martínez; Ernesto Ramírez-González; Maribel González-Villa
EPI_ISL_17703582	LESP Nuevo Leon	Instituto de Diagnostico y Referencia Epidemiologicos (INDRE)	Abril Rodríguez-Maldonado; Claudia Wong-Arámbula; Felipe Arguijo-Perez; Helios Cárdenas-Hernández; Carmen Castro-Méndez; Lidia García-Torres; Ruth Madera-Sandoval; América Mandujano-Martínez; Nancy Martínez-Velázquez; Mireya Mederos-Michel; Angélica Pedraza-Meléndez; Joaquín Quiroz-Mercado; Daniel Regalado-Santiago; Silvia Rivero-Arredondo; Erika Sierra-Atanacio; Fernando González-Domínguez; Lucia Hernández-Rivas, Irma López-Martínez; Ernesto Ramírez-González; Maribel González-Villa
EPI_ISL_17703583	LESP Quintana Roo	Instituto de Diagnostico y Referencia Epidemiologicos (INDRE)	Abril Rodríguez-Maldonado; Claudia Wong-Arámbula; Felipe Arguijo-Perez; Helios Cárdenas-Hernández; Carmen Castro-Méndez; Lidia García-Torres; Ruth Madera-Sandoval; América Mandujano-Martínez; Nancy Martínez-Velázquez; Mireya Mederos-Michel; Angélica Pedraza-Meléndez; Joaquín Quiroz-Mercado; Daniel Regalado-Santiago; Silvia Rivero-Arredondo; Erika Sierra-Atanacio; Fernando González-Domínguez; Lucia Hernández-Rivas, Irma López-Martínez; Ernesto Ramírez-González; Maribel González-Villa
EPI_ISL_17703584	LESP Chihuahua	Instituto de Diagnostico y Referencia Epidemiologicos (INDRE)	Abril Rodríguez-Maldonado; Claudia Wong-Arámbula; Felipe Arguijo-Perez; Helios Cárdenas-Hernández; Carmen Castro-Méndez; Lidia García-Torres; Ruth Madera-Sandoval; América Mandujano-Martínez; Nancy Martínez-Velázquez; Mireya Mederos-Michel; Angélica Pedraza-Meléndez; Joaquín Quiroz-Mercado; Daniel Regalado-Santiago; Silvia Rivero-Arredondo; Erika Sierra-Atanacio; Fernando González-Domínguez; Lucia Hernández-Rivas, Irma López-Martínez; Ernesto Ramírez-González; Maribel González-Villa
EPI_ISL_17703585	LESP Yucatan	Instituto de Diagnostico y Referencia Epidemiologicos (INDRE)	Abril Rodríguez-Maldonado; Claudia Wong-Arámbula; Felipe Arguijo-Perez; Helios Cárdenas-Hernández; Carmen Castro-Méndez; Lidia García-Torres; Ruth Madera-Sandoval; América Mandujano-Martínez; Nancy Martínez-Velázquez; Mireya Mederos-Michel; Angélica Pedraza-Meléndez; Joaquín Quiroz-Mercado; Daniel Regalado-Santiago; Silvia Rivero-Arredondo; Erika Sierra-Atanacio; Fernando González-Domínguez; Lucia Hernández-Rivas, Irma López-Martínez; Ernesto Ramírez-González; Maribel González-Villa
EPI_ISL_17703586, EPI_ISL_17703587	LESP Nuevo Leon	Instituto de Diagnostico y Referencia Epidemiologicos (INDRE)	Abril Rodríguez-Maldonado; Claudia Wong-Arámbula; Felipe Arguijo-Perez; Helios Cárdenas-Hernández; Carmen Castro-Méndez; Lidia García-Torres; Ruth Madera-Sandoval; América Mandujano-Martínez; Nancy Martínez-Velázquez; Mireya Mederos-Michel; Angélica Pedraza-Meléndez; Joaquín Quiroz-Mercado; Daniel Regalado-Santiago; Silvia Rivero-Arredondo; Erika Sierra-Atanacio; Fernando González-Domínguez; Lucia Hernández-Rivas, Irma López-Martínez; Ernesto Ramírez-González; Maribel González-Villa
EPI_ISL_17703588, EPI_ISL_17703589	LESP Quintana Roo	Instituto de Diagnostico y Referencia Epidemiologicos (INDRE)	Abril Rodríguez-Maldonado; Claudia Wong-Arámbula; Felipe Arguijo-Perez; Helios Cárdenas-Hernández; Carmen Castro-Méndez; Lidia García-Torres; Ruth Madera-Sandoval; América Mandujano-Martínez; Nancy Martínez-Velázquez; Mireya Mederos-Michel; Angélica Pedraza-Meléndez; Joaquín Quiroz-Mercado; Daniel Regalado-Santiago; Silvia Rivero-Arredondo; Erika Sierra-Atanacio; Fernando González-Domínguez; Lucia Hernández-Rivas, Irma López-Martínez; Ernesto Ramírez-González; Maribel González-Villa
EPI_ISL_17703590	LESP State of Mexico	Instituto de Diagnostico y Referencia Epidemiologicos (INDRE)	Abril Rodríguez-Maldonado; Claudia Wong-Arámbula; Felipe Arguijo-Perez; Helios Cárdenas-Hernández; Carmen Castro-Méndez; Lidia García-Torres; Ruth Madera-Sandoval; América Mandujano-Martínez; Nancy Martínez-Velázquez; Mireya Mederos-Michel; Angélica Pedraza-Meléndez; Joaquín Quiroz-Mercado; Daniel Regalado-Santiago; Silvia Rivero-Arredondo; Erika Sierra-Atanacio; Fernando González-Domínguez; Lucia Hernández-Rivas, Irma López-Martínez; Ernesto Ramírez-González; Maribel González-Villa
EPI_ISL_17703591	LESP Campeche	Instituto de Diagnostico y Referencia Epidemiologicos (INDRE)	Abril Rodríguez-Maldonado; Claudia Wong-Arámbula; Felipe Arguijo-Perez; Helios Cárdenas-Hernández; Carmen Castro-Méndez; Lidia García-Torres; Ruth Madera-Sandoval; América Mandujano-Martínez; Nancy Martínez-Velázquez; Mireya Mederos-Michel; Angélica Pedraza-Meléndez; Joaquín Quiroz-Mercado; Daniel Regalado-Santiago; Silvia Rivero-Arredondo; Erika Sierra-Atanacio; Fernando González-Domínguez; Lucia Hernández-Rivas, Irma López-Martínez; Ernesto Ramírez-González; Maribel González-Villa
EPI_ISL_17703592	LESP Nuevo Leon	Instituto de Diagnostico y Referencia Epidemiologicos (INDRE)	Abril Rodríguez-Maldonado; Claudia Wong-Arámbula; Felipe Arguijo-Perez; Helios Cárdenas-Hernández; Carmen Castro-Méndez; Lidia García-Torres; Ruth Madera-Sandoval; América Mandujano-Martínez; Nancy Martínez-Velázquez; Mireya Mederos-Michel; Angélica Pedraza-Meléndez; Joaquín Quiroz-Mercado; Daniel Regalado-Santiago; Silvia Rivero-Arredondo; Erika Sierra-Atanacio; Fernando González-Domínguez; Lucia Hernández-Rivas, Irma López-Martínez; Ernesto Ramírez-González; Maribel González-Villa
EPI_ISL_17703593, EPI_ISL_17703594	LESP Jalisco	Instituto de Diagnostico y Referencia Epidemiologicos (INDRE)	Abril Rodríguez-Maldonado; Claudia Wong-Arámbula; Felipe Arguijo-Perez; Helios Cárdenas-Hernández; Carmen Castro-Méndez; Lidia García-Torres; Ruth Madera-Sandoval; América Mandujano-Martínez; Nancy Martínez-Velázquez; Mireya Mederos-Michel; Angélica Pedraza-Meléndez; Joaquín Quiroz-Mercado; Daniel Regalado-Santiago; Silvia Rivero-Arredondo; Erika Sierra-Atanacio; Fernando González-Domínguez; Lucia Hernández-Rivas, Irma López-Martínez; Ernesto Ramírez-González; Maribel González-Villa
EPI_ISL_17703595, EPI_ISL_17703596	LESP Nuevo Leon	Instituto de Diagnostico y Referencia Epidemiologicos (INDRE)	Abril Rodríguez-Maldonado; Claudia Wong-Arámbula; Felipe Arguijo-Perez; Helios Cárdenas-Hernández; Carmen Castro-Méndez; Lidia García-Torres; Ruth Madera-Sandoval; América Mandujano-Martínez; Nancy Martínez-Velázquez; Mireya Mederos-Michel; Angélica Pedraza-Meléndez; Joaquín Quiroz-Mercado; Daniel Regalado-Santiago; Silvia Rivero-Arredondo; Erika Sierra-Atanacio; Fernando González-Domínguez; Lucia Hernández-Rivas, Irma López-Martínez; Ernesto Ramírez-González; Maribel González-Villa
EPI_ISL_17703597	Laboratorio Estatal de Salud Pública Nuevo Leon	Instituto de Diagnostico y Referencia Epidemiologicos (INDRE)	Abril Rodríguez-Maldonado; Claudia Wong-Arámbula; Felipe Arguijo-Perez; Helios Cárdenas-Hernández; Carmen Castro-Méndez; Lidia García-Torres; Ruth Madera-Sandoval; América Mandujano-Martínez; Nancy Martínez-Velázquez; Mireya Mederos-Michel; Angélica Pedraza-Meléndez; Joaquín Quiroz-Mercado; Daniel Regalado-Santiago; Silvia Rivero-Arredondo; Erika Sierra-Atanacio; Fernando González-Domínguez; Lucia Hernández-Rivas, Irma López-Martínez; Ernesto Ramírez-González; Maribel González-Villa
EPI_ISL_17703598	LESP Quintana Roo	Instituto de Diagnostico y Referencia Epidemiologicos (INDRE)	Abril Rodríguez-Maldonado; Claudia Wong-Arámbula; Felipe Arguijo-Perez; Helios Cárdenas-Hernández; Carmen Castro-Méndez; Lidia García-Torres; Ruth Madera-Sandoval; América Mandujano-Martínez; Nancy Martínez-Velázquez; Mireya Mederos-Michel; Angélica Pedraza-Meléndez; Joaquín Quiroz-Mercado; Daniel Regalado-Santiago; Silvia Rivero-Arredondo; Erika Sierra-Atanacio; Fernando González-Domínguez; Lucia Hernández-Rivas, Irma López-Martínez; Ernesto Ramírez-González; Maribel González-Villa
EPI_ISL_17703599, EPI_ISL_17703600, EPI_ISL_17703601, EPI_ISL_17703602, EPI_ISL_17703603, EPI_ISL_17703604	LESP Nuevo Leon	Instituto de Diagnostico y Referencia Epidemiologicos (INDRE)	Abril Rodríguez-Maldonado; Claudia Wong-Arámbula; Felipe Arguijo-Perez; Helios Cárdenas-Hernández; Carmen Castro-Méndez; Lidia García-Torres; Ruth Madera-Sandoval; América Mandujano-Martínez; Nancy Martínez-Velázquez; Mireya Mederos-Michel; Angélica Pedraza-Meléndez; Joaquín Quiroz-Mercado; Daniel Regalado-Santiago; Silvia Rivero-Arredondo; Erika Sierra-Atanacio; Fernando González-Domínguez; Lucia Hernández-Rivas, Irma López-Martínez; Ernesto Ramírez-González; Maribel González-Villa
EPI_ISL_17703605	LESP Chihuahua	Instituto de Diagnostico y Referencia Epidemiologicos (INDRE)	Abril Rodríguez-Maldonado; Claudia Wong-Arámbula; Felipe Arguijo-Perez; Helios Cárdenas-Hernández; Carmen Castro-Méndez; Lidia García-Torres; Ruth Madera-Sandoval; América Mandujano-Martínez; Nancy Martínez-Velázquez; Mireya Mederos-Michel; Angélica Pedraza-Meléndez; Joaquín Quiroz-Mercado; Daniel Regalado-Santiago; Silvia Rivero-Arredondo; Erika Sierra-Atanacio; Fernando González-Domínguez; Lucia Hernández-Rivas, Irma López-Martínez; Ernesto Ramírez-González; Maribel González-Villa
EPI_ISL_17703606	LESP Nuevo Leon	Instituto de Diagnostico y Referencia Epidemiologicos (INDRE)	Abril Rodríguez-Maldonado; Claudia Wong-Arámbula; Felipe Arguijo-Perez; Helios Cárdenas-Hernández; Carmen Castro-Méndez; Lidia García-Torres; Ruth Madera-Sandoval; América Mandujano-Martínez; Nancy Martínez-Velázquez; Mireya Mederos-Michel; Angélica Pedraza-Meléndez; Joaquín Quiroz-Mercado; Daniel Regalado-Santiago; Silvia Rivero-Arredondo; Erika Sierra-Atanacio; Fernando González-Domínguez; Lucia Hernández-Rivas, Irma López-Martínez; Ernesto Ramírez-González; Maribel González-Villa
EPI_ISL_17703607, EPI_ISL_17703608, EPI_ISL_17703609, EPI_ISL_17703610	LESP Mexico City	Instituto de Diagnostico y Referencia Epidemiologicos (INDRE)	Abril Rodríguez-Maldonado; Claudia Wong-Arámbula; Felipe Arguijo-Perez; Helios Cárdenas-Hernández; Carmen Castro-Méndez; Lidia García-Torres; Ruth Madera-Sandoval; América Mandujano-Martínez; Nancy Martínez-Velázquez; Mireya Mederos-Michel; Angélica Pedraza-Meléndez; Joaquín Quiroz-Mercado; Daniel Regalado-Santiago; Silvia Rivero-Arredondo; Erika Sierra-Atanacio; Fernando González-Domínguez; Lucia Hernández-Rivas, Irma López-Martínez; Ernesto Ramírez-González; Maribel González-Villa
EPI_ISL_17703611	LESP Tlaxcala	Instituto de Diagnostico y Referencia Epidemiologicos (INDRE)	Abril Rodríguez-Maldonado; Claudia Wong-Arámbula; Felipe Arguijo-Perez; Helios Cárdenas-Hernández; Carmen Castro-Méndez; Lidia García-Torres; Ruth Madera-Sandoval; América Mandujano-Martínez; Nancy Martínez-Velázquez; Mireya Mederos-Michel; Angélica Pedraza-Meléndez; Joaquín Quiroz-Mercado; Daniel Regalado-Santiago; Silvia Rivero-Arredondo; Erika Sierra-Atanacio; Fernando González-Domínguez; Lucia Hernández-Rivas, Irma López-Martínez; Ernesto Ramírez-González; Maribel González-Villa
EPI_ISL_17703612	LESP Nuevo Leon	Instituto de Diagnostico y Referencia Epidemiologicos (INDRE)	Abril Rodríguez-Maldonado; Claudia Wong-Arámbula; Felipe Arguijo-Perez; Helios Cárdenas-Hernández; Carmen Castro-Méndez; Lidia García-Torres; Ruth Madera-Sandoval; América Mandujano-Martínez; Nancy Martínez-Velázquez; Mireya Mederos-Michel; Angélica Pedraza-Meléndez; Joaquín Quiroz-Mercado; Daniel Regalado-Santiago; Silvia Rivero-Arredondo; Erika Sierra-Atanacio; Fernando González-Domínguez; Lucia Hernández-Rivas, Irma López-Martínez; Ernesto Ramírez-González; Maribel González-Villa
EPI_ISL_17703743, EPI_ISL_17703744	Parasitology Laboratory, Institute of Tropical Medicine of Sao Paulo, School of Medicine, University of Sao Paulo	Parasitology Laboratory	Raissa Heloisa de Araujo Eliodoro, Ingra Claro Morales, Ester Cerdeira Sabino
EPI_ISL_17703745	Parasitology Laboratory, Institute of Tropical Medicine of Sao Paulo, School of Medicine, University of Sao Paulo	Parasitology Laboratory	Raissa Heloisa de Araujo Eliodoro, Ingra Morales Claro, Ester Cerdeira Sabino
EPI_ISL_17703746	Parasitology Laboratory, Institute of Tropical Medicine of Sao Paulo, School of Medicine, University of Sao Paulo	Parasitology Laboratory	Raissa Heloisa de Araujo Eliodoro, Ingra Claro Morales, Ester Cerdeira Sabino
EPI_ISL_17718458	Department of Virology, National Institute of Health, Islamabad, Pakistan	Department of Virology, National Institute of Health, Islamabad, Pakistan	Massab Umair, Muhammad Ammar, Syed Adnan Haider, Rabia Hakim, Qasim Malik, Muhammad Salman, Ghazala Parveen, and Naseem Akhtar
EPI_ISL_17722468	Tokyo Metropolitan Institute of Public Health	Tokyo Metropolitan Institute of Public Health	Fumi Kasuya, Wakaba Okada, Ryota Kumagai, Sachiko Harada, Arisa Amano, Michiya Hasegawa, Mami Nagashima, Kenji Sadamasu
EPI_ISL_17736865, EPI_ISL_17736866, EPI_ISL_17736867, EPI_ISL_17736868, EPI_ISL_17736869, EPI_ISL_17736870, EPI_ISL_17736871, EPI_ISL_17736872, EPI_ISL_17736873, EPI_ISL_17736874, EPI_ISL_17736875, EPI_ISL_17736876, EPI_ISL_17736877, EPI_ISL_17736878, EPI_ISL_17736879, EPI_ISL_17736880, EPI_ISL_17736881, EPI_ISL_17736882, EPI_ISL_17736883, EPI_ISL_17736884, EPI_ISL_17736885, EPI_ISL_17736886, EPI_ISL_17736887, EPI_ISL_17736888, EPI_ISL_17736889, EPI_ISL_17736890, EPI_ISL_17736891			
see above	Charité Universitätsmedizin Berlin, Institute for Virology/Laboratory Berlin	Charité Universitätsmedizin Berlin, Institute for Virology	Terry C. Jones, Julia Melchert, Barbara Mühlemann, Talitha Veith, Jörn Beheim-Schwarzbach, Julia Tesch, Marie Luisa Schmidt, Felix Walper, Tobias Bleicker, Caroline Isner, Frieder Pfäfflin, Ricardo Niklas Werner, Victor M. Corman, Christian Drosten
EPI_ISL_17737466, EPI_ISL_17737467, EPI_ISL_17737468, EPI_ISL_17737469, EPI_ISL_17737470, EPI_ISL_17737471, EPI_ISL_17737472, EPI_ISL_17737473, EPI_ISL_17737474, EPI_ISL_17737475, EPI_ISL_17737476, EPI_ISL_17737477, EPI_ISL_17737478, EPI_ISL_17737479, EPI_ISL_17737480, EPI_ISL_17737481, EPI_ISL_17737482, EPI_ISL_17737483, EPI_ISL_17737484, EPI_ISL_17737485, EPI_ISL_17737486, EPI_ISL_17737488, EPI_ISL_17737489, EPI_ISL_17737490, EPI_ISL_17737491, EPI_ISL_17737492, EPI_ISL_17737493, EPI_ISL_17737495, EPI_ISL_17737496, EPI_ISL_17737497, EPI_ISL_17737498, EPI_ISL_17737499, EPI_ISL_17737500, EPI_ISL_17737501, EPI_ISL_17737502, EPI_ISL_17737503, EPI_ISL_17737504, EPI_ISL_17737505, EPI_ISL_17737506, EPI_ISL_17737507, EPI_ISL_17737508, EPI_ISL_17737509, EPI_ISL_17737510, EPI_ISL_17737511, EPI_ISL_17737512, EPI_ISL_17737513, EPI_ISL_17737514, EPI_ISL_17737515, EPI_ISL_17737516, EPI_ISL_17737517, EPI_ISL_17737518, EPI_ISL_17737519, EPI_ISL_17737520, EPI_ISL_17737521, EPI_ISL_17737522, EPI_ISL_17737523, EPI_ISL_17737524, EPI_ISL_17737525, EPI_ISL_17737526, EPI_ISL_17737527, EPI_ISL_17737528, EPI_ISL_17737529, EPI_ISL_17737530, EPI_ISL_17737531, EPI_ISL_17737532, EPI_ISL_17737533, EPI_ISL_17737534, EPI_ISL_17737535, EPI_ISL_17737536, EPI_ISL_17737537, EPI_ISL_17737538, EPI_ISL_17737539, EPI_ISL_17737540, EPI_ISL_17737541, EPI_ISL_17737542, EPI_ISL_17737543, EPI_ISL_17737544, EPI_ISL_17737545, EPI_ISL_17737546, EPI_ISL_17737547, EPI_ISL_17737548, EPI_ISL_17737549, EPI_ISL_17737550, EPI_ISL_17737551, EPI_ISL_17737553, EPI_ISL_17737555, EPI_ISL_17737556			
see above	Public Health Ontario	Public Health Ontario	Isabel S, Eshaghi A, Duvvuri VR, Gubbay JB, Cronin K, Li A, Hasso M, Clark ST, Hopkins JP, Patel SN, Braukmann TWA
EPI_ISL_17762484, EPI_ISL_17762485	Tokyo Metropolitan Institute of Public Health	Tokyo Metropolitan Institute of Public Health	Fumi Kasuya, Wakaba Okada, Ryota Kumagai, Sachiko Harada, Arisa Amano, Michiya Hasegawa, Mami Nagashima, Kenji Sadamasu
EPI_ISL_17779992	Servicio de Microbiologia Hospital Ramon y Cajal	Servicio de Microbiologia Hospital Ramon y Cajal	Ponce-Alonso M, Martinez-García L, Olavarrieta L, Galan JC
EPI_ISL_17789831	Delaware Public Health Lab	Delaware Public Health Lab	Moneeb Bajwa & Holly Miller

EPI_ISL_17793219	Microbiology, Immunology and Transplantation, KU Leuven	Microbiology, Immunology and Transplantation, KU Leuven	Wawina-Bokalanga,T., Vanmechelen,B., Logist,A.-S., Bloemen,M. and Maes,P.
EPI_ISL_17793220	Microbiology, Immunology and Transplantation, KU Leuven	Microbiology, Immunology and Transplantation, KU Leuven	Vanmechelen,B., Wawina-Bokalanga,T., Logist,A.-S., Bloemen,M. and Maes,P.
EPI_ISL_17793221, EPI_ISL_17793222	Microbiology, Immunology and Transplantation, KU Leuven	Microbiology, Immunology and Transplantation, KU Leuven	Wawina-Bokalanga,T., Vanmechelen,B., Logist,A.-S., Bloemen,M. and Maes,P.
EPI_ISL_17793223, EPI_ISL_17793224, EPI_ISL_17793225	Microbiology, Immunology and Transplantation, KU Leuven	Microbiology, Immunology and Transplantation, KU Leuven	Vanmechelen,B., Wawina-Bokalanga,T., Logist,A.-S., Bloemen,M. and Maes,P.
EPI_ISL_17793226	Microbiology, Immunology and Transplantation, KU Leuven	Microbiology, Immunology and Transplantation, KU Leuven	Wawina-Bokalanga,T., Vanmechelen,B., Logist,A.-S., Bloemen,M. and Maes,P.
EPI_ISL_17793227	Microbiology, Immunology and Transplantation, KU Leuven	Microbiology, Immunology and Transplantation, KU Leuven	Vanmechelen,B., Wawina-Bokalanga,T., Logist,A.-S., Bloemen,M. and Maes,P.
EPI_ISL_17793229, EPI_ISL_17793230	Microbiology, Immunology and Transplantation, KU Leuven	Microbiology, Immunology and Transplantation, KU Leuven	Wawina-Bokalanga,T., Vanmechelen,B., Logist,A.-S., Sinnesael,R., Ysebaert,L., Verlinden,J., Van Holm,B., Bloemen,M. and Maes,P.
EPI_ISL_17793231	Microbiology, Immunology and Transplantation, KU Leuven	Microbiology, Immunology and Transplantation, KU Leuven	Vanmechelen,B., Wawina-Bokalanga,T., Logist,A.-S., Bloemen,M. and Maes,P.
EPI_ISL_17793232	Microbiology, Immunology and Transplantation, KU Leuven	Microbiology, Immunology and Transplantation, KU Leuven	Wawina-Bokalanga,T., Vanmechelen,B., Logist,A.-S., Bloemen,M. and Maes,P.
EPI_ISL_17793233, EPI_ISL_17793234	Microbiology, Immunology and Transplantation, KU Leuven	Microbiology, Immunology and Transplantation, KU Leuven	Vanmechelen,B., Wawina-Bokalanga,T., Logist,A.-S., Bloemen,M. and Maes,P.
EPI_ISL_17793235	Microbiology, Immunology and Transplantation, KU Leuven	Microbiology, Immunology and Transplantation, KU Leuven	Wawina-Bokalanga,T., Vanmechelen,B., Logist,A.-S., Bloemen,M. and Maes,P.
EPI_ISL_17793236, EPI_ISL_17793238, EPI_ISL_17793239	Microbiology, Immunology and Transplantation, KU Leuven	Microbiology, Immunology and Transplantation, KU Leuven	Vanmechelen,B., Wawina-Bokalanga,T., Logist,A.-S., Bloemen,M. and Maes,P.
EPI_ISL_17793240	Microbiology, Immunology and Transplantation, KU Leuven	Microbiology, Immunology and Transplantation, KU Leuven	Vanmechelen,B., Wawina-Bokalanga,T., Logist,A.-S., Sinnesael,R., Ysebaert,L., Verlinden,J., Van Holm,B., Bloemen,M. and Maes,P.
EPI_ISL_17793241	Microbiology, Immunology and Transplantation, KU Leuven	Microbiology, Immunology and Transplantation, KU Leuven	Wawina-Bokalanga,T., Vanmechelen,B., Logist,A.-S., Bloemen,M. and Maes,P.
EPI_ISL_17793242, EPI_ISL_17793243	Microbiology, Immunology and Transplantation, KU Leuven	Microbiology, Immunology and Transplantation, KU Leuven	Vanmechelen,B., Wawina-Bokalanga,T., Logist,A.-S., Bloemen,M. and Maes,P.
EPI_ISL_17793244, EPI_ISL_17793245	Microbiology, Immunology and Transplantation, KU Leuven	Microbiology, Immunology and Transplantation, KU Leuven	Wawina-Bokalanga,T., Vanmechelen,B., Logist,A.-S., Bloemen,M. and Maes,P.
EPI_ISL_17793246	Microbiology, Immunology and Transplantation, KU Leuven	Microbiology, Immunology and Transplantation, KU Leuven	Wawina-Bokalanga,T., Vanmechelen,B., Logist,A.-S., Horemans,M., Ysebaert,L., Verlinden,J., Van Holm,B., Bloemen,M. and Maes,P.
EPI_ISL_17793247, EPI_ISL_17793248, EPI_ISL_17793250	Microbiology, Immunology and Transplantation, KU Leuven	Microbiology, Immunology and Transplantation, KU Leuven	Vanmechelen,B., Wawina-Bokalanga,T., Logist,A.-S., Bloemen,M. and Maes,P.
EPI_ISL_17793251	Microbiology, Immunology and Transplantation, KU Leuven	Microbiology, Immunology and Transplantation, KU Leuven	Wawina-Bokalanga,T., Vanmechelen,B., Logist,A.-S., Bloemen,M. and Maes,P.
EPI_ISL_17793252, EPI_ISL_17793253	Microbiology, Immunology and Transplantation, KU Leuven	Microbiology, Immunology and Transplantation, KU Leuven	Vanmechelen,B., Wawina-Bokalanga,T., Logist,A.-S., Bloemen,M. and Maes,P.
EPI_ISL_17793254	Microbiology, Immunology and Transplantation, KU Leuven	Microbiology, Immunology and Transplantation, KU Leuven	Wawina-Bokalanga,T., Vanmechelen,B., Logist,A.-S., Ysebaert,L., Horemans,M., Verlinden,J., Van Holm,B., Bloemen,M. and Maes,P.
EPI_ISL_17793255	Microbiology, Immunology and Transplantation, KU Leuven	Microbiology, Immunology and Transplantation, KU Leuven	Wawina-Bokalanga,T., Vanmechelen,B., Logist,A.-S., Sinnesael,R., Ysebaert,L., Verlinden,J., Van Holm,B., Bloemen,M. and Maes,P.
EPI_ISL_17793256	Microbiology, Immunology and Transplantation, KU Leuven	Microbiology, Immunology and Transplantation, KU Leuven	Vanmechelen,B., Wawina-Bokalanga,T., Logist,A.-S., Bloemen,M. and Maes,P.
EPI_ISL_17793257	Microbiology, Immunology and Transplantation, KU Leuven	Microbiology, Immunology and Transplantation, KU Leuven	Wawina-Bokalanga,T., Vanmechelen,B., Logist,A.-S., Bloemen,M. and Maes,P.
EPI_ISL_17793258	Microbiology, Immunology and Transplantation, KU Leuven	Microbiology, Immunology and Transplantation, KU Leuven	Vanmechelen,B., Wawina-Bokalanga,T., Logist,A.-S., Bloemen,M. and Maes,P.
EPI_ISL_17793259, EPI_ISL_17793260, EPI_ISL_17793261	Microbiology, Immunology and Transplantation, KU Leuven	Microbiology, Immunology and Transplantation, KU Leuven	Wawina-Bokalanga,T., Vanmechelen,B., Logist,A.-S., Bloemen,M. and Maes,P.
EPI_ISL_17793262, EPI_ISL_17793263, EPI_ISL_17793264, EPI_ISL_17793265, EPI_ISL_17793266, EPI_ISL_17793267	Microbiology, Immunology and Transplantation, KU Leuven	Microbiology, Immunology and Transplantation, KU Leuven	Vanmechelen,B., Wawina-Bokalanga,T., Logist,A.-S., Bloemen,M. and Maes,P.
EPI_ISL_17793268	Microbiology, Immunology and Transplantation, KU Leuven	Microbiology, Immunology and Transplantation, KU Leuven	Wawina-Bokalanga,T., Vanmechelen,B., Logist,A.-S., Ysebaert,L., Verlinden,J., Sinnesael,R., Van Holm,B., Bloemen,M. and Maes,P.
EPI_ISL_17793269	Microbiology, Immunology and Transplantation, KU Leuven	Microbiology, Immunology and Transplantation, KU Leuven	Wawina-Bokalanga,T., Vanmechelen,B., Logist,A.-S., Bloemen,M. and Maes,P.
EPI_ISL_17793270	Microbiology, Immunology and Transplantation, KU Leuven	Microbiology, Immunology and Transplantation, KU Leuven	Vanmechelen,B., Wawina-Bokalanga,T., Logist,A.-S., Bloemen,M. and Maes,P.
EPI_ISL_17793271	Microbiology, Immunology and Transplantation, KU Leuven	Microbiology, Immunology and Transplantation, KU Leuven	Wawina-Bokalanga,T., Vanmechelen,B., Logist,A.-S., Bloemen,M., Van Holm,B. and Maes,P.
EPI_ISL_17793272, EPI_ISL_17793273	Microbiology, Immunology and Transplantation, KU Leuven	Microbiology, Immunology and Transplantation, KU Leuven	Wawina-Bokalanga,T., Vanmechelen,B., Logist,A.-S., Bloemen,M. and Maes,P.
EPI_ISL_17793274	Microbiology, Immunology and Transplantation, KU Leuven	Microbiology, Immunology and Transplantation, KU Leuven	Vanmechelen,B., Wawina-Bokalanga,T., Logist,A.-S., Bloemen,M. and Maes,P.
EPI_ISL_17793275	Microbiology, Immunology and Transplantation, KU Leuven	Microbiology, Immunology and Transplantation, KU Leuven	Wawina-Bokalanga,T., Vanmechelen,B., Logist,A.-S., Bloemen,M. and Maes,P.
EPI_ISL_17793276, EPI_ISL_17793277	Microbiology, Immunology and Transplantation, KU Leuven	Microbiology, Immunology and Transplantation, KU Leuven	Vanmechelen,B., Wawina-Bokalanga,T., Logist,A.-S., Bloemen,M. and Maes,P.
EPI_ISL_17793278	Microbiology, Immunology and Transplantation, KU Leuven	Microbiology, Immunology and Transplantation, KU Leuven	Wawina-Bokalanga,T., Mechelen,B., Logist,A.-S., Bloemen,M. and Maes,P.
EPI_ISL_17793279	Microbiology, Immunology and Transplantation, KU Leuven	Microbiology, Immunology and Transplantation, KU Leuven	Wawina-Bokalanga,T., Vanmechelen,B., Logist,A.-S., Bloemen,M. and Maes,P.
EPI_ISL_17793280, EPI_ISL_17793281	Microbiology, Immunology and Transplantation, KU Leuven	Microbiology, Immunology and Transplantation, KU Leuven	Vanmechelen,B., Wawina-Bokalanga,T., Logist,A.-S., Bloemen,M. and Maes,P.
EPI_ISL_17793282	Microbiology, Immunology and Transplantation, KU Leuven	Microbiology, Immunology and Transplantation, KU Leuven	Vanmechelen,B., Wawina-Bokalanga,T., Logist,A.-S., Van Holm,B., Bloemen,M. and Maes,P.
EPI_ISL_17793283	Microbiology, Immunology and Transplantation, KU Leuven	Microbiology, Immunology and Transplantation, KU Leuven	Wawina-Bokalanga,T., Vanmechelen,B., Logist,A.-S., Bloemen,M. and Maes,P.
EPI_ISL_17793284	Microbiology, Immunology and Transplantation, KU Leuven	Microbiology, Immunology and Transplantation, KU Leuven	Vanmechelen,B., Wawina-Bokalanga,T., Logist,A.-S., Bloemen,M. and Maes,P.
EPI_ISL_17793285	Microbiology, Immunology and Transplantation, KU Leuven	Microbiology, Immunology and Transplantation, KU Leuven	Wawina-Bokalanga,T., Vanmechelen,B., Logist,A.-S., Bloemen,M. and Maes,P.
EPI_ISL_17793286	Microbiology, Immunology and Transplantation, KU Leuven	Microbiology, Immunology and Transplantation, KU Leuven	Vanmechelen,B., Wawina-Bokalanga,T., Logist,A.-S., Bloemen,M. and Maes,P.
EPI_ISL_17793287	Microbiology, Immunology and Transplantation, KU Leuven	Microbiology, Immunology and Transplantation, KU Leuven	Vanmechelen,B., Wawona-Bokalanga,T., Logist,A.-S., Bloemen,M. and Maes,P.
EPI_ISL_17793288, EPI_ISL_17793289	Microbiology, Immunology and Transplantation, KU Leuven	Microbiology, Immunology and Transplantation, KU Leuven	Wawina-Bokalanga,T., Vanmechelen,B., Logist,A.-S., Bloemen,M. and Maes,P.

EPI_ISL_17793290, EPI_ISL_17793291	Microbiology, Immunology and Transplantation, KU Leuven	Microbiology, Immunology and Transplantation, KU Leuven	Vanmechelen,B., Wawina-Bokalanga,T., Logist,A.-S., Bloemen,M. and Maes,P.
EPI_ISL_17793292, EPI_ISL_17793293, EPI_ISL_17793294, EPI_ISL_17793295	Microbiology, Immunology and Transplantation, KU Leuven	Microbiology, Immunology and Transplantation, KU Leuven	Wawina-Bokalanga,T., Vanmechelen,B., Logist,A.-S., Bloemen,M. and Maes,P.
EPI_ISL_17797712	Microbiology, Immunology and Transplantation, KU Leuven	Microbiology, Immunology and Transplantation, KU Leuven	Vanmechelen,B., Wawina-Bokalanga,T., Logis,A.-S., Bloemen,M., Van Holm,B. and Maes,P.
EPI_ISL_17797713	Microbiology, Immunology and Transplantation, KU Leuven	Microbiology, Immunology and Transplantation, KU Leuven	Vanmechelen,B., Wawina-Bokalanga,T., Logist,A.-S., Van Holm,B., Bloemen,M. and Maes,P.
EPI_ISL_17797714	Microbiology, Immunology and Transplantation, KU Leuven	Microbiology, Immunology and Transplantation, KU Leuven	Wawina-Bokalanga,T., Vanmechelen,B., Logist,A.-S., Van Holm,B., Bloemen,M. and Maes,P.
EPI_ISL_17797715	Microbiology, Immunology and Transplantation, KU Leuven	Microbiology, Immunology and Transplantation, KU Leuven	Vanmechelen,B., Wawina-Bokalanga,T., Logist,A.-S., Bloemen,M. and Maes,P.
EPI_ISL_17797716	Microbiology, Immunology and Transplantation, KU Leuven	Microbiolgy, Immunology and Transplantation, KU Leuven	Wawina-Bokalanga,T., Vanmechelen,B., Logist,A.-S., Bloemen,M. and Maes,P.
EPI_ISL_17797717	Microbiology, Immunology and Transplantation, KU Leuven	Microbiology, Immunology and Transplantation, KU Leuven	Vanmechelen,B., Wawina-Bokalanga,T., Logist,A.-S., Van Holm,B., Bloemen,M. and Maes,P.
EPI_ISL_17797718	Microbiology, Immunology and Transplantation, KU Leuven	Microbiology, Immunology and Transplantation, KU Leuven	Wawina-Bokalanga,T., Vanmechelen,B., Logist,A.-S., Bloemen,M. and Maes,P.
EPI_ISL_17797719	Microbiology, Immunology and Transplantation, KU Leuven	Microbiology, Immunology and Transplantation, KU Leuven	Vanmechelen,B., Wawina-Bokalanga,T., Logist,A.-S., Van Holm,B., Bloemen,M. and Maes,P.
EPI_ISL_17797720, EPI_ISL_17797721	Microbiology, Immunology and Transplantation, KU Leuven	Microbiology, Immunology and Transplantation, KU Leuven	Wawina-Bokalanga,T., Vanmechelen,B., Logist,A.-S., Bloemen,M. and Maes,P.
EPI_ISL_17797722	Microbiology, Immunology and Transplantation, KU Leuven	Microbiology, Immunology and Transplantation, KU Leuven	Vanmechelen,B., Wawina-Bokalanga,T., Logist,A.-S., Bloemen,M., Van Holm,B. and Maes,P.
EPI_ISL_17797723	Microbiology, Immunology and Transplantation, KU Leuven	Microbiology, Immunology and Transplantation, KU Leuven	Vanmechelen,B., Wawina-Bokalanga,T., Logist,A.-S., Bloemen,M. and Maes,P.
EPI_ISL_17797724, EPI_ISL_17797725	Microbiology, Immunology and Transplantation, KU Leuven	Microbiology, Immunology and Transplantation, KU Leuven	Wawina-Bokalanga,T., Vanmechelen,B., Logist,A.-S., Bloemen,M. and Maes,P.
EPI_ISL_17797726, EPI_ISL_17797727	Microbiology, Immunology and Transplantation, KU Leuven	Microbiology, Immunology and Transplantation, KU Leuven	Wawina-Bokalanga,T., Vanmechelen,B., Logist,A.-S., Van Holm,B., Bloemen,M. and Maes,P.
EPI_ISL_17797728	Microbiology, Immunology and Transplantation, KU Leuven	Microbiology, Immunology and Transplantation, KU Leuven	Wawina-Bokalanga,T., Vanmechelen,B., Logist,A.-s., Van Holm,B., Bloemen,M. and Maes,P.
EPI_ISL_17797729, EPI_ISL_17797730, EPI_ISL_17797731	Microbiology, Immunology and Transplantation, KU Leuven	Microbiology, Immunology and Transplantation, KU Leuven	Wawina-Bokalanga,T., Vanmechelen,B., Logist,A.-S., Bloemen,M. and Maes,P.
EPI_ISL_17797732	Microbiology, Immunology and Transplantation, KU Leuven	Microbiology, Immunology and Transplantation, KU Leuven	Vanmechelen,B., Wawina-Bokalanga,T., Logist,A.-S., Van Holm,B., Bloemen,M. and Maes,P.
EPI_ISL_17797733	Microbiology, Immunology and Transplantation, KU Leuven	Microbiology, Immunology and Transplantation, KU Leuven	Wawina-Bokalanga,T., Vanmechelen,B., Logist,A.-S., Van Holm,B., Bloemen,M. and Maes,P.
EPI_ISL_17797734, EPI_ISL_17797735, EPI_ISL_17797736	Microbiology, Immunology and Transplantation, KU Leuven	Microbiology, Immunology and Transplantation, KU Leuven	Wawina-Bokalanga,T., Vanmechelen,B., Logist,A.-S., Bloemen,M. and Maes,P.
EPI_ISL_17797737, EPI_ISL_17797738	Microbiology, Immunology and Transplantation, KU Leuven	Microbiology, Immunology and Transplantation, KU Leuven	Wawina-Bokalanga,T., Vanmechelen,B., Logist,A.-S., Van Holm,B., Bloemen,M. and Maes,P.
EPI_ISL_17797739	Microbiology, Immunology and Transplantation, KU Leuven	Microbiology, Immunology and Transplantation, KU Leuven	Vanmechelen,B., Wawina-Bokalanga,T., Logist,A.-S., Van Holm,B., Bloemen,M. and Maes,P.
EPI_ISL_17797740	Microbiology, Immunology and Transplantation, KU Leuven	Microbiology, Immunology and Transplantation, KU Leuven	Vanmechelen,B., Wawina-Bokalanga,T., Logist,A.-S., Bloemen,M. and Maes,P.
EPI_ISL_17797741	Microbiology, Immunology and Transplantation, KU Leuven	Microbiology, Immunology and Transplantation, KU Leuven	Vanmechelen,B., Wawina-Bokalanga,T., Logist,A.-S., Van Holm,B., Bloemen,M. and Maes,P.
EPI_ISL_17797742	Microbiology, Immunology and Transplantation, KU Leuven	Microbiology, Immunology and Transplantation, KU Leuven	Wawina-Bokalanga,T., Vanmechelen,B., Logist,A.-S., Bloemen,M. and Maes,P.
EPI_ISL_17797743	Microbiology, Immunology and Transplantation, KU Leuven	Microbiology, Immunology and Transplantation, KU Leuven	Vanmechelen,B., Wawina-Bokalanga,T., Logist,A.-S., Van Holm,B., Bloemen,M. and Maes,P.
EPI_ISL_17797744	Microbiology, Immunology and Transplantation, KU Leuven	Microbiology, Immunology and Transplantation, KU Leuven	Vanmechelen,B., Wawina-Bokalanga,T., Logist,A.-S., Bloemen,M., Van Holm,B. and Maes,P.
EPI_ISL_17797745, EPI_ISL_17797746	Microbiology, Immunology and Transplantation, KU Leuven	Microbiology, Immunology and Transplantation, KU Leuven	Wawina-Bokalanga,T., Vanmechelen,B., Logist,A.-S., Bloemen,M. and Maes,P.
EPI_ISL_17797747	Microbiology, Immunology and Transplantation, KU Leuven	Microbiology, Immunology and Transplantation, KU Leuven	Wawina-Bokalanga,T., Vanmechelen,B., Logist,A.-S., Van Holm,B., Bloemen,M. and Maes,P.
EPI_ISL_17797748	Microbiology, Immunology and Transplantation, KU Leuven	Microbiology, Immunology and Transplantation, KU Leuven	Vanmechelen,B., Wawina-Bokalanga,T., Logist,A.-S., Bloemen,M., Van Holm,B. and Maes,P.
EPI_ISL_17797749	Microbiology, Immunology and Transplantation, KU Leuven	Microbiology, Immunology and Transplantation, KU Leuven	Vanmechelen,B., Wawina-Bokalanga,T., Logist,A.-S., Bloemen,M. and Maes,P.
EPI_ISL_17797750	Microbiology, Immunology and Transplantation, KU Leuven	Microbiology, Immunology and Transplantation, KU Leuven	Vanmechelen,B., Wawina-Bokalanga,T., Logist,A.-S., Van Holm,B., Bloemen,M. and Maes,P.
EPI_ISL_17797751	Microbiology, Immunology and Transplantation, KU Leuven	Microbiology, Immunology and Transplantation, KU Leuven	Wawina-Bokalanga,T., Vanmechelen,B., Logist,A.-S., Van Holm,B., Bloemen,M. and Maes,P.
EPI_ISL_17797752	Microbiology, Immunology and Transplantation, KU Leuven	Microbiology, Immunology and Transplantation, KU Leuven	Vanmechelen,B., Wawina-Bokalanga,T., Logist,A.-S., Van Holm,B., Bloemen,M. and Maes,P.
EPI_ISL_17797753	Microbiology, Immunology and Transplantation, KU Leuven	Microbiology, Immunology and Transplantation, KU Leuven	Wawina-Bokalanga,T., Vanmechelen,B., Logist,A.-S., Van Holm,B., Bloemen,M. and Maes,P.
EPI_ISL_17797754	Microbiology, Immunology and Transplantation, KU Leuven	Microbiology, Immunology and Transplantation, KU Leuven	Wawina-Bokalanga,T., Vanmechelen,B., Logist,A.-S., Bloemen,M. and Maes,P.
EPI_ISL_17804477	Center for Virology, Medical University of Vienna	Center for Virology, Medical University of Vienna	Camp,J.V., Redlberger-Fritz,M. and Aberle,S.W.
EPI_ISL_17809521	Hangzhou Center for Disease Control and Prevention	Hangzhou Center for Disease Control and Prevention	Lijiao Ao , Jun Li , Yue Yu
EPI_ISL_17817239, EPI_ISL_17817240, EPI_ISL_17817241	Tokyo Metropolitan Institute of Public Health	Tokyo Metropolitan Institute of Public Health	Fumi Kasuya, Wakaba Okada, Ryota Kumagai, Sachiko Harada, Arisa Amano, Michiya Hasegawa, Mami Nagashima, Kenji Sadamasu
EPI_ISL_17821080, EPI_ISL_17821081, EPI_ISL_17821082, EPI_ISL_17821083, EPI_ISL_17821084, EPI_ISL_17821085, EPI_ISL_17821086, EPI_ISL_17821087, EPI_ISL_17821088	National Institute for Infectious Diseases "Matei Bals"	National Institute for Infectious Diseases "Matei Bals"	Robert Hohan, Ovidiu Vlaicu, Marius Surleac, Leontina Banica, Andreea Tudor, Simona Paraschiv
EPI_ISL_17821096, EPI_ISL_17821097, EPI_ISL_17821098	ACL Laboratories	RIPHL at Rush University Medical Center	Stefan Green, Kevin Kunstman, Hannah Barbian, Felix Araujo Perez, Edith Perez, Sofiya Bobrovska, Alyse Kittner, Cecilia Chau, Giancarlo Balangue, Lok Yiu Ashley Wu
EPI_ISL_17821099, EPI_ISL_17821100, EPI_ISL_17821101	Quest Diagnostics	RIPHL at Rush University Medical Center	Stefan Green, Kevin Kunstman, Hannah Barbian, Felix Araujo Perez, Edith Perez, Sofiya Bobrovska, Alyse Kittner, Cecilia Chau, Giancarlo Balangue, Lok Yiu Ashley Wu
EPI_ISL_17831608	Delaware Public Health Lab	Delaware Public Health Lab	Miller,H. and Bajwa,M.
EPI_ISL_17834476	California Department of Public Health	California Department of Public Health	Kath, C., Haw, M., Espinosa, A., and Hacker, J.
EPI_ISL_17837266, EPI_ISL_17837267, EPI_ISL_17837268, EPI_ISL_17959214, EPI_ISL_17959215, EPI_ISL_17959216	Tokyo Metropolitan Institute of Public Health	Tokyo Metropolitan Institute of Public Health	Fumi Kasuya, Wakaba Okada, Ryota Kumagai, Sachiko Harada, Arisa Amano, Michiya Hasegawa, Mami Nagashima, Kenji Sadamasu
EPI_ISL_17960863, EPI_ISL_17960864, EPI_ISL_17960865, EPI_ISL_17960866	Laboratorio Nacional de Salud Pública Dr. Defilló	Laboratorio Nacional de Salud Pública Dr. Defilló	Isaac Miguel Sánchez, Carlos Vergara Castillo, Edwin Féliz, Anny Peña, Pedro Martinez, Yeny E. Lara Perez, Robinson Agramonte
EPI_ISL_17972012, EPI_ISL_17972014, EPI_ISL_17972015	California Department of Public Health (CDPH)	California Department of Public Health (CDPH)	Kath,C., Haw,M., Espinosa,A. and Hacker,J.
EPI_ISL_17972019, EPI_ISL_17972020, EPI_ISL_17972021	Centers for Disease Control and Prevention DHCPP-PRB, CDC	Centers for Disease Control and Prevention DHCPP-PRB, CDC	Li,C.M.

EPI_ISL_17972022	Centers for Disease Control and Prevention DHCPP-PRB, CDC	Centers for Disease Control and Prevention DHCPP-PRB, CDC	Gigante,C., Johnson,S., Zhao,H., Batra,D., Hetrick,E., Howard,D., Kovar,L., Seabolt,M., Morrison,S., Desch,M., Knipe,K., Weigand,M., Cintron,R., Burgin,A., Burroughs,M., Lee,J., Wilkins,K., McCollum,A., Hutson,C., Davidson,W., Rao,A., Riner,D. and Li,Y.
EPI_ISL_17972023, EPI_ISL_17972024, EPI_ISL_17972025, EPI_ISL_17972026, EPI_ISL_17972027	Centers for Disease Control and Prevention DHCPP-PRB, CDC	Centers for Disease Control and Prevention DHCPP-PRB, CDC	Li,C.M.
EPI_ISL_17972028	Centers for Disease Control and Prevention DHCPP-PRB, CDC	Centers for Disease Control and Prevention DHCPP-PRB, CDC	Gigante,C., Thomas,L., Zhao,H., Batra,D., Hetrick,E., Howard,D., Kovar,L., Seabolt,M., Morrison,S., Desch,M., Knipe,K., Weigand,M., Cintron,R., Burgin,A., Burroughs,M., Lee,J., Wilkins,K., McCollum,A., Hutson,C., Davidson,W., Rao,A., Dunn,J. and Li,Y.
EPI_ISL_17972029, EPI_ISL_17972030	Centers for Disease Control and Prevention DHCPP-PRB, CDC	Centers for Disease Control and Prevention DHCPP-PRB, CDC	Li,C.M.
EPI_ISL_17972031	Centers for Disease Control and Prevention DHCPP-PRB, CDC	Centers for Disease Control and Prevention DHCPP-PRB, CDC	Gigante,C., Kubin,G., Zhao,H., Batra,D., Hetrick,E., Howard,D., Kovar,L., Seabolt,M., Morrison,S., Desch,M., Knipe,K., Weigand,M., Cintron,R., Burgin,A., Burroughs,M., Lee,J., Wilkins,K., McCollum,A., Hutson,C., Davidson,W., Rao,A., White,S. and Li,Y.
EPI_ISL_17972032	Centers for Disease Control and Prevention DHCPP-PRB, CDC	Centers for Disease Control and Prevention DHCPP-PRB, CDC	Li,C.M.
EPI_ISL_17972033	Centers for Disease Control and Prevention DHCPP-PRB, CDC	Centers for Disease Control and Prevention DHCPP-PRB, CDC	Gigante,C., Ostadkar,R., Zhao,H., Batra,D., Hetrick,E., Howard,D., Kovar,L., Seabolt,M., Morrison,S., Desch,M., Knipe,K., Weigand,M., Cintron,R., Burgin,A., Burroughs,M., Lee,J., Wilkins,K., McCollum,A., Hutson,C., Davidson,W., Rao,A., Wang,X. and Li,Y.
EPI_ISL_17972034	Centers for Disease Control and Prevention DHCPP-PRB, CDC	Centers for Disease Control and Prevention DHCPP-PRB, CDC	Gigante,C., Johnson,S., Zhao,H., Batra,D., Hetrick,E., Howard,D., Kovar,L., Seabolt,M., Morrison,S., Desch,M., Knipe,K., Weigand,M., Cintron,R., Burgin,A., Burroughs,M., Lee,J., Wilkins,K., McCollum,A., Hutson,C., Davidson,W., Rao,A., Riner,D. and Li,Y.
EPI_ISL_17972035	Centers for Disease Control and Prevention DHCPP-PRB, CDC	Centers for Disease Control and Prevention DHCPP-PRB, CDC	Gigante,C., Ostadkar,R., Zhao,H., Batra,D., Hetrick,E., Howard,D., Kovar,L., Seabolt,M., Morrison,S., Desch,M., Knipe,K., Weigand,M., Cintron,R., Burgin,A., Burroughs,M., Lee,J., Wilkins,K., McCollum,A., Hutson,C., Davidson,W., Rao,A., Wang,X. and Li,Y.
EPI_ISL_17972036, EPI_ISL_17972037, EPI_ISL_17972038	Centers for Disease Control and Prevention DHCPP-PRB, CDC	Centers for Disease Control and Prevention DHCPP-PRB, CDC	Li,C.M.
EPI_ISL_17972039	Centers for Disease Control and Prevention DHCPP-PRB, CDC	Centers for Disease Control and Prevention DHCPP-PRB, CDC	Gigante,C., Kubin,G., Zhao,H., Batra,D., Hetrick,E., Howard,D., Kovar,L., Seabolt,M., Morrison,S., Desch,M., Knipe,K., Weigand,M., Cintron,R., Burgin,A., Burroughs,M., Lee,J., Wilkins,K., McCollum,A., Hutson,C., Davidson,W., Rao,A., White,S. and Li,Y.
EPI_ISL_17972040	Centers for Disease Control and Prevention DHCPP-PRB, CDC	Centers for Disease Control and Prevention DHCPP-PRB, CDC	Li,C.M.
EPI_ISL_17977751	Centro Medico ABC	Instituto Nacional de Medicina Genomica	Cedro Tanda Alberto, Roxana Trejo Gonzalez, Laura Gomez-Romero, Alfredo Mendoza-Vargas, Dora Garnica-Lopez, Alfredo Hidalgo-Miranda.
EPI_ISL_17979000, EPI_ISL_17979001, EPI_ISL_17979002	Quest Diagnostics	Regional Innovative Public Health Laboratory at Rush University Medical Center	Stefan Green, Kevin Kunstman, Hannah Barbian, Sofiya Bobrovska, Felix Araujo Perez, Edith Perez, Cecilia Chau, Giancarlo Balangue, Lok Yiu Ashley Wu, Trisha Jeon, Marisol Dominguez, Latifah Boyd
EPI_ISL_17980808	Hospital Ramon y Cajal	Hospital Ramon y Cajal	Ponce-Alonso,M., Martinez-Garcia,L., Olavarrieta,L. and Galan,J.C.
EPI_ISL_17988349, EPI_ISL_17988350, EPI_ISL_17988351, EPI_ISL_17988352, EPI_ISL_17988353, EPI_ISL_17988354, EPI_ISL_17988355, EPI_ISL_17988356, EPI_ISL_17988357, EPI_ISL_17988358, EPI_ISL_17988359, EPI_ISL_17988360, EPI_ISL_17988361, EPI_ISL_17988362, EPI_ISL_17988363, EPI_ISL_17988364, EPI_ISL_17988365, EPI_ISL_17988366, EPI_ISL_17988367, EPI_ISL_17988368, EPI_ISL_17988369, EPI_ISL_17988370, EPI_ISL_17988371, EPI_ISL_17988372, EPI_ISL_17988373, EPI_ISL_17988374, EPI_ISL_17988375, EPI_ISL_17988376, EPI_ISL_17988377, EPI_ISL_17988378			
see above	Laboratorio Central de Salud Publica	Laboratorio Central de Salud Publica	Cynthia Vazquez, Vagner Fonseca, Andrea Gomez de la Fuente, Sandra Gonzalez, Fatima Fleitas, Mauricio Lima, Natalia R. Guimaraes, Felipe C. M. Iani, Analia Rojas, Tania Alfonso, Cesar Cantero, Julio Barrios, Shirley Villalba, Maria Jose Ortega, Juan Torales, Maria Liz Gamarra, Carolina Aquino, Jairo Mendez Rico, Luiz Carlos Junior Alcantara, Marta Giovanetti
EPI_ISL_18044981, EPI_ISL_18044982, EPI_ISL_18044983, EPI_ISL_18044984, EPI_ISL_18044987	Center for Vectors and Infectious Diseases Research (CEVDI), National Health Institute Doutor Ricardo Jorge, IP (INSA),	Center for Vectors and Infectious Diseases Research (CEVDI), National Health Institute Doutor Ricardo Jorge, IP (INSA),	Isidro,J., Borges,V., Pinto,M., Sobral,D., Santos,J., Nunes,A., Mixao,V., Ferreira,R., Santos,D., Duarte,S., Vieira,L., Borrego,M.J., Nuncio,S., Lopes de Carvalho,I., Pelerito,A., Cordeiro,R. and Gomes,J.P.
EPI_ISL_18055899, EPI_ISL_18055900	Tokyo Metropolitan Institute of Public Health	Tokyo Metropolitan Institute of Public Health	Fumi Kasuya, Wakaba Okada, Ryota Kumagai, Sachiko Harada, Arisa Amano, Michiya Hasegawa, Mami Nagashima, Kenji Sadamasu
EPI_ISL_18059182, EPI_ISL_18059183, EPI_ISL_18059184	Department of Acute Infectious Diseases Control and Prevention, Yunnan Center for Disease Control and Prevention	Department of Acute Infectious Diseases Control and Prevention, Yunnan Center for Disease Control and Prevention	Meiling Zhang, Ruize Ni, Xiaoqing Fu
EPI_ISL_18064640, EPI_ISL_18064641, EPI_ISL_18064642, EPI_ISL_18064643, EPI_ISL_18064644, EPI_ISL_18064645, EPI_ISL_18064648	Laboratory of Microbiology and Virology, Ospedale Amedeo di Savoia, ASL "Città di Torino"	Laboratory of Microbiology and Virology, Ospedale Amedeo di Savoia, ASL "Città di Torino"	Francesco Cerutti, Tiziano Allice, Maria Grazia Milia, Gabriella Gregori, Elisa Burdino, Sara Monteleone, Marisa Cazzadore, Valeria Ghisetti
EPI_ISL_18075506, EPI_ISL_18075507, EPI_ISL_18075508	Tokyo Metropolitan Institute of Public Health	Tokyo Metropolitan Institute of Public Health	Fumi Kasuya, Wakaba Okada, Ryota Kumagai, Sachiko Harada, Arisa Amano, Michiya Hasegawa, Mami Nagashima, Kenji Sadamasu
EPI_ISL_18076378, EPI_ISL_18076379, EPI_ISL_18076380, EPI_ISL_18076382, EPI_ISL_18076383, EPI_ISL_18076384, EPI_ISL_18076385, EPI_ISL_18076386, EPI_ISL_18076388, EPI_ISL_18076389	Center for Vectors and Infectious Diseases Research (CEVDI), National Health Institute Doutor Ricardo Jorge, IP (INSA)	Center for Vectors and Infectious Diseases Research (CEVDI), National Health Institute Doutor Ricardo Jorge, IP (INSA)	Isidro,J., Borges,V., Pinto,M., Sobral,D., Santos,J., Nunes,A., Mixao,V., Ferreira,R., Santos,D., Duarte,S., Vieira,L., Borrego,M.J., Nuncio,S., Lopes de Carvalho,I., Pelerito,A., Cordeiro,R. and Gomes,J.P.
EPI_ISL_18097375	Tokyo Metropolitan Institute of Public Health	Tokyo Metropolitan Institute of Public Health	Fumi Kasuya, Wakaba Okada, Ryota Kumagai, Sachiko Harada, Arisa Amano, Michiya Hasegawa, Mami Nagashima, Kenji Sadamasu
EPI_ISL_18125028, EPI_ISL_18125029, EPI_ISL_18125030, EPI_ISL_18125031, EPI_ISL_18125033, EPI_ISL_18125034, EPI_ISL_18125035, EPI_ISL_18125036, EPI_ISL_18125037, EPI_ISL_18125038, EPI_ISL_18125039, EPI_ISL_18125040, EPI_ISL_18125041, EPI_ISL_18125042, EPI_ISL_18125043, EPI_ISL_18125044, EPI_ISL_18125045, EPI_ISL_18125046			
see above	University of Washington, Department of Laboratory Medicine	University of Washington, Department of Laboratory Medicine	Sereewit,J., Xie,H., Roychoudhury,P. and Greninger,A.L.
EPI_ISL_18128768	Bichat-Claude Bernard Hospital, Paris France	Institut Pasteur	Aurelia Kwasiborski, Véronique Hourdel, Charlotte Balière, Damien Hoinard, Quentin Grassin, Maxence Feher, Clémentine De La Porte Des Vaux, Mélanie Cresta, Jessica Vanhomwegen, Jean-Claude Manuguerra, Christophe Batéjat, Valérie Caro
EPI_ISL_18131371, EPI_ISL_18131372, EPI_ISL_18131373, EPI_ISL_18131374, EPI_ISL_18131375, EPI_ISL_18131376, EPI_ISL_18131377, EPI_ISL_18131378, EPI_ISL_18131379, EPI_ISL_18131380, EPI_ISL_18131381, EPI_ISL_18131382, EPI_ISL_18131383, EPI_ISL_18131384, EPI_ISL_18131385, EPI_ISL_18131386, EPI_ISL_18131387, EPI_ISL_18131388, EPI_ISL_18131389, EPI_ISL_18131391, EPI_ISL_18131392, EPI_ISL_18131393, EPI_ISL_18131395, EPI_ISL_18131396, EPI_ISL_18131397, EPI_ISL_18131398, EPI_ISL_18131400, EPI_ISL_18131401, EPI_ISL_18131402, EPI_ISL_18131403, EPI_ISL_18131404, EPI_ISL_18131405, EPI_ISL_18131406, EPI_ISL_18131407, EPI_ISL_18131408, EPI_ISL_18131409, EPI_ISL_18131410, EPI_ISL_18131411, EPI_ISL_18131412, EPI_ISL_18131413, EPI_ISL_18131414, EPI_ISL_18131415, EPI_ISL_18131416, EPI_ISL_18131417, EPI_ISL_18131418, EPI_ISL_18131419, EPI_ISL_18131420, EPI_ISL_18131421, EPI_ISL_18131422, EPI_ISL_18131423, EPI_ISL_18131424, EPI_ISL_18131426, EPI_ISL_18131427, EPI_ISL_18131428, EPI_ISL_18131429, EPI_ISL_18131430, EPI_ISL_18131431, EPI_ISL_18131432, EPI_ISL_18131433, EPI_ISL_18131434, EPI_ISL_18131435, EPI_ISL_18131436, EPI_ISL_18131437, EPI_ISL_18131438, EPI_ISL_18131439, EPI_ISL_18131440, EPI_ISL_18131441, EPI_ISL_18131442, EPI_ISL_18131443, EPI_ISL_18131444, EPI_ISL_18131445, EPI_ISL_18131446, EPI_ISL_18131447, EPI_ISL_18131448, EPI_ISL_18131449, EPI_ISL_18131450, EPI_ISL_18131451, EPI_ISL_18131452, EPI_ISL_18131453, EPI_ISL_18131454, EPI_ISL_18131455, EPI_ISL_18131456, EPI_ISL_18131457, EPI_ISL_18131458, EPI_ISL_18131459, EPI_ISL_18131460, EPI_ISL_18131461, EPI_ISL_18131462, EPI_ISL_18131463, EPI_ISL_18131464, EPI_ISL_18131465, EPI_ISL_18131466, EPI_ISL_18131467, EPI_ISL_18131468, EPI_ISL_18131469, EPI_ISL_18131470, EPI_ISL_18131471, EPI_ISL_18131472, EPI_ISL_18131473, EPI_ISL_18131474, EPI_ISL_18131475, EPI_ISL_18131476, EPI_ISL_18131477, EPI_ISL_18131478, EPI_ISL_18131479, EPI_ISL_18131480, EPI_ISL_18131481, EPI_ISL_18131482, EPI_ISL_18131483, EPI_ISL_18131484, EPI_ISL_18131485, EPI_ISL_18131486, EPI_ISL_18131487, EPI_ISL_18131488, EPI_ISL_18131489, EPI_ISL_18131490, EPI_ISL_18131491			
see above	Robert Koch Institute	Robert Koch Institute	Brinkmann,A., Pape,K., Kohl,C., Schrick,L., Michel,J., Schaade,L. and Nitsche,A.
EPI_ISL_18137801, EPI_ISL_18137802, EPI_ISL_18137803	Northwestern Medicine	RIPHL at Rush University Medical Center	Stefan Green, Kevin Kunstman, Hannah Barbian, Sofiya Bobrovska, Felix Araujo Perez, Edith Perez, Cecilia Chau, Giancarlo Balangue, Lok Yiu Ashley Wu, Trisha Jeon, Marisol Dominguez, Latifah Boyd, Lacy Simons
EPI_ISL_18137804	Quest Diagnostics	RIPHL at Rush University Medical Center	Stefan Green, Kevin Kunstman, Hannah Barbian, Sofiya Bobrovska, Felix Araujo Perez, Edith Perez, Cecilia Chau, Giancarlo Balangue, Lok Yiu Ashley Wu, Trisha Jeon, Marisol Dominguez, Latifah Boyd
EPI_ISL_18137805	ACL Laboratories	RIPHL at Rush University Medical Center	Stefan Green, Kevin Kunstman, Hannah Barbian, Sofiya Bobrovska, Felix Araujo Perez, Edith Perez, Cecilia Chau, Giancarlo Balangue, Lok Yiu Ashley Wu, Trisha Jeon, Marisol Dominguez, Latifah Boyd
EPI_ISL_18137806	Quest Diagnostics	RIPHL at Rush University Medical Center	Stefan Green, Kevin Kunstman, Hannah Barbian, Sofiya Bobrovska, Felix Araujo Perez, Edith Perez, Cecilia Chau, Giancarlo Balangue, Lok Yiu Ashley Wu, Trisha Jeon, Marisol Dominguez, Latifah Boyd
EPI_ISL_18137807, EPI_ISL_18137808	Northwestern Medicine	RIPHL at Rush University Medical Center	Stefan Green, Kevin Kunstman, Hannah Barbian, Sofiya Bobrovska, Felix Araujo Perez, Edith Perez, Cecilia Chau, Giancarlo Balangue, Lok Yiu Ashley Wu, Trisha Jeon, Marisol Dominguez, Latifah Boyd, Lacy Simons
EPI_ISL_18137809, EPI_ISL_18137810, EPI_ISL_18137811, EPI_ISL_18137812, EPI_ISL_18137813	Quest Diagnostics	RIPHL at Rush University Medical Center	Stefan Green, Kevin Kunstman, Hannah Barbian, Sofiya Bobrovska, Felix Araujo Perez, Edith Perez, Cecilia Chau, Giancarlo Balangue, Lok Yiu Ashley Wu, Trisha Jeon, Marisol Dominguez, Latifah Boyd
EPI_ISL_18137814, EPI_ISL_18137815, EPI_ISL_18137816, EPI_ISL_18137817, EPI_ISL_18137818, EPI_ISL_18137819, EPI_ISL_18137820	Northwestern Medicine	RIPHL at Rush University Medical Center	Stefan Green, Kevin Kunstman, Hannah Barbian, Sofiya Bobrovska, Felix Araujo Perez, Edith Perez, Cecilia Chau, Giancarlo Balangue, Lok Yiu Ashley Wu, Trisha Jeon, Marisol Dominguez, Latifah Boyd, Lacy Simons
EPI_ISL_18137821, EPI_ISL_18137822, EPI_ISL_18137823, EPI_ISL_18137824, EPI_ISL_18137825, EPI_ISL_18137826, EPI_ISL_18137827, EPI_ISL_18137828, EPI_ISL_18137829	Universidad de Chile	Universidad de Chile	Ampuero,M.M.A.
EPI_ISL_18137830	University of Washington, Department of Laboratory Medicine	University of Washington, Department of Laboratory Medicine	Sereewit,J., Xie,H., Roychoudhury,P. and Greninger,A.L.
EPI_ISL_18147334, EPI_ISL_18147335, EPI_ISL_18147336, EPI_ISL_18147337, EPI_ISL_18147338, EPI_ISL_18147339, EPI_ISL_18147340, EPI_ISL_18147341, EPI_ISL_18147342, EPI_ISL_18147343, EPI_ISL_18147344, EPI_ISL_18147345, EPI_ISL_18147346, EPI_ISL_18147347, EPI_ISL_18147348, EPI_ISL_18147349, EPI_ISL_18147350, EPI_ISL_18147351, EPI_ISL_18147352, EPI_ISL_18147353, EPI_ISL_18147354, EPI_ISL_18147355, EPI_ISL_18147356, EPI_ISL_18147357, EPI_ISL_18147358, EPI_ISL_18147359, EPI_ISL_18147360, EPI_ISL_18147361, EPI_ISL_18147362, EPI_ISL_18147363			
see above	Korea Disease Control and Prevention Agency	Korea Disease Control and Prevention Agency	Chung,Y.-S., Yi,H., Choi,M.-M., Kim,J.-W., Lee,M., Lee,S., Sim,G., Lee,J.H., Shin,H. and Choi,C.
EPI_ISL_18147364, EPI_ISL_18147365, EPI_ISL_18147366, EPI_ISL_18147367, EPI_ISL_18147368, EPI_ISL_18147369, EPI_ISL_18147370, EPI_ISL_18147371, EPI_ISL_18147372, EPI_ISL_18147373, EPI_ISL_18147374, EPI_ISL_18147375, EPI_ISL_18147376, EPI_ISL_18147377, EPI_ISL_18147378, EPI_ISL_18147379, EPI_ISL_18147380, EPI_ISL_18147381, EPI_ISL_18147382, EPI_ISL_18147383, EPI_ISL_18147384, EPI_ISL_18147385, EPI_ISL_18147386, EPI_ISL_18147387, EPI_ISL_18147388, EPI_ISL_18147389, EPI_ISL_18147390, EPI_ISL_18147391, EPI_ISL_18147392, EPI_ISL_18147393, EPI_ISL_18147394, EPI_ISL_18147395, EPI_ISL_18147396, EPI_ISL_18147397, EPI_ISL_18147398, EPI_ISL_18147399, EPI_ISL_18147400, EPI_ISL_18147401, EPI_ISL_18147402, EPI_ISL_18147403, EPI_ISL_18147404, EPI_ISL_18147405, EPI_ISL_18147406, EPI_ISL_18147407, EPI_ISL_18147408, EPI_ISL_18147409, EPI_ISL_18147410, EPI_ISL_18147411, EPI_ISL_18147412, EPI_ISL_18147413, EPI_ISL_18147414, EPI_ISL_18147415, EPI_ISL_18147416, EPI_ISL_18147417, EPI_ISL_18147418, EPI_ISL_18147419, EPI_ISL_18147420			
see above	Centre for Biological Threats, Highly Pathogenic Viruses, Robert Koch Institute	Centre for Biological Threats, Highly Pathogenic Viruses, Robert Koch Institute	Brinkmann,A., Pape,K., Kohl,C., Schrick,L., Michel,J., Schaade,L. and Nitsche,A.
EPI_ISL_18147421	Division of High-Consequence Pathogens & Pathology (DHCPP) - Poxvirus and Rabies Branch (PRB) - Centers for Disease Control and Prevention (CDC)	Division of High-Consequence Pathogens & Pathology (DHCPP) - Poxvirus and Rabies Branch (PRB) - Centers for Disease Control and Prevention (CDC)	Gigante,C., Kubin,G., Zhao,H., Batra,D., Hetrick,E., Howard,D., Kovar,L., Seabolt,M., Morrison,S., Desch,M., Knipe,K., Weigand,M., Mcgrath,D., Takakuwa,J., Burgin,A., Burroughs,M., Lee,J., Wilkins,K., Mccollum,A., Hutson,C., Davidson,W., Rao,A., White,S. and Li,Y.
EPI_ISL_18147422	Division of High-Consequence Pathogens & Pathology (DHCPP) - Poxvirus and Rabies Branch (PRB) - Centers for Disease Control and Prevention (CDC)	Division of High-Consequence Pathogens & Pathology (DHCPP) - Poxvirus and Rabies Branch (PRB) - Centers for Disease Control and Prevention (CDC)	Gigante,C., Smole,S., Zhao,H., Batra,D., Hetrick,E., Howard,D., Kovar,L., Seabolt,M., Morrison,S., Desch,M., Knipe,K., Weigand,M., Mcgrath,D., Takakuwa,J., Burgin,A., Burroughs,M., Lee,J., Wilkins,K., Mccollum,A., Hutson,C., Davidson,W., Rao,A., Brown,C. and Li,Y.
EPI_ISL_18147423	Division of High-Consequence Pathogens & Pathology	Division of High-Consequence Pathogens & Pathology	Gigante,C., Ruiz,V., Zhao,H., Batra,D., Hetrick,E., Howard,D., Kovar,L., Seabolt,M., Morrison,S., Desch,M., Knipe,K., Weigand,M., Mcgrath,D., Takakuwa,J., Burgin,A., Burroughs,M., Lee,J., Wilkins,K., Mccollum,A., Hutson,C.,

	(DHCPP) - Poxvirus and Rabies Branch (PRB) - Centers for Disease Control and Prevention (CDC)	(DHCPP) - Poxvirus and Rabies Branch (PRB) - Centers for Disease Control and Prevention (CDC)	Davidson,W., Rao,A., Wang,J. and Li,Y.
EPI_ISL_18147424	Division of High-Consequence Pathogens & Pathology (DHCPP) - Poxvirus and Rabies Branch (PRB) - Centers for Disease Control and Prevention (CDC)	Division of High-Consequence Pathogens & Pathology (DHCPP) - Poxvirus and Rabies Branch (PRB) - Centers for Disease Control and Prevention (CDC)	Gigante,C., Johnson,S., Zhao,H., Batra,D., Hetrick,E., Howard,D., Kovar,L., Seabolt,M., Morrison,S., Desch,M., Knipe,K., Weigand,M., Mcgrath,D., Takakuwa,J., Burgin,A., Burroughs,M., Lee,J., Wilkins,K., Mccollum,A., Hutson,C., Davidson,W., Rao,A., Riner,D. and Li,Y.
EPI_ISL_18147425	Division of High-Consequence Pathogens & Pathology (DHCPP) - Poxvirus and Rabies Branch (PRB) - Centers for Disease Control and Prevention (CDC)	Division of High-Consequence Pathogens & Pathology (DHCPP) - Poxvirus and Rabies Branch (PRB) - Centers for Disease Control and Prevention (CDC)	Gigante,C., Kubin,G., Zhao,H., Batra,D., Hetrick,E., Howard,D., Kovar,L., Seabolt,M., Morrison,S., Desch,M., Knipe,K., Weigand,M., Mcgrath,D., Takakuwa,J., Burgin,A., Burroughs,M., Lee,J., Wilkins,K., Mccollum,A., Hutson,C., Davidson,W., Rao,A., White,S. and Li,Y.
EPI_ISL_18147426	Division of High-Consequence Pathogens & Pathology (DHCPP) - Poxvirus and Rabies Branch (PRB) - Centers for Disease Control and Prevention (CDC)	Division of High-Consequence Pathogens & Pathology (DHCPP) - Poxvirus and Rabies Branch (PRB) - Centers for Disease Control and Prevention (CDC)	Gigante,C., Hauser,J., Zhao,H., Batra,D., Hetrick,E., Howard,D., Kovar,L., Seabolt,M., Morrison,S., Desch,M., Knipe,K., Weigand,M., Mcgrath,D., Takakuwa,J., Burgin,A., Burroughs,M., Lee,J., Wilkins,K., Mccollum,A., Hutson,C., Davidson,W., Rao,A., Mangla,A. and Li,Y.
EPI_ISL_18147427	Division of High-Consequence Pathogens & Pathology (DHCPP) - Poxvirus and Rabies Branch (PRB) - Centers for Disease Control and Prevention (CDC)	Division of High-Consequence Pathogens & Pathology (DHCPP) - Poxvirus and Rabies Branch (PRB) - Centers for Disease Control and Prevention (CDC)	Gigante,C., Ventura,J., Zhao,H., Batra,D., Hetrick,E., Howard,D., Kovar,L., Seabolt,M., Morrison,S., Desch,M., Knipe,K., Weigand,M., Mcgrath,D., Takakuwa,J., Burgin,A., Burroughs,M., Lee,J., Wilkins,K., Mccollum,A., Hutson,C., Davidson,W., Rao,A., Nash,J. and Li,Y.
EPI_ISL_18147428, EPI_ISL_18147429, EPI_ISL_18147430, EPI_ISL_18147431	Division of High-Consequence Pathogens & Pathology (DHCPP) - Poxvirus and Rabies Branch (PRB) - Centers for Disease Control and Prevention (CDC)	Division of High-Consequence Pathogens & Pathology (DHCPP) - Poxvirus and Rabies Branch (PRB) - Centers for Disease Control and Prevention (CDC)	Gigante,C., Ruiz,V., Zhao,H., Batra,D., Hetrick,E., Howard,D., Kovar,L., Seabolt,M., Morrison,S., Desch,M., Knipe,K., Weigand,M., Mcgrath,D., Takakuwa,J., Burgin,A., Burroughs,M., Lee,J., Wilkins,K., Mccollum,A., Hutson,C., Davidson,W., Rao,A., Wang,J. and Li,Y.
EPI_ISL_18147432	Division of High-Consequence Pathogens & Pathology (DHCPP) - Poxvirus and Rabies Branch (PRB) - Centers for Disease Control and Prevention (CDC)	Division of High-Consequence Pathogens & Pathology (DHCPP) - Poxvirus and Rabies Branch (PRB) - Centers for Disease Control and Prevention (CDC)	Gigante,C., Pettit,D., Zhao,H., Batra,D., Hetrick,E., Howard,D., Kovar,L., Seabolt,M., Morrison,S., Desch,M., Knipe,K., Weigand,M., Mcgrath,D., Takakuwa,J., Burgin,A., Burroughs,M., Lee,J., Wilkins,K., Mccollum,A., Hutson,C., Davidson,W., Rao,A., Deutsch-Feldman,M. and Li,Y.
EPI_ISL_18147433	Division of High-Consequence Pathogens & Pathology (DHCPP) - Poxvirus and Rabies Branch (PRB) - Centers for Disease Control and Prevention (CDC)	Division of High-Consequence Pathogens & Pathology (DHCPP) - Poxvirus and Rabies Branch (PRB) - Centers for Disease Control and Prevention (CDC)	Gigante,C., Kubin,G., Zhao,H., Batra,D., Hetrick,E., Howard,D., Kovar,L., Seabolt,M., Morrison,S., Desch,M., Knipe,K., Weigand,M., Mcgrath,D., Takakuwa,J., Burgin,A., Burroughs,M., Lee,J., Wilkins,K., Mccollum,A., Hutson,C., Davidson,W., Rao,A., White,S. and Li,Y.
EPI_ISL_18147434	Division of High-Consequence Pathogens & Pathology (DHCPP) - Poxvirus and Rabies Branch (PRB) - Centers for Disease Control and Prevention (CDC)	Division of High-Consequence Pathogens & Pathology (DHCPP) - Poxvirus and Rabies Branch (PRB) - Centers for Disease Control and Prevention (CDC)	Gigante,C., Thomas,L., Zhao,H., Batra,D., Hetrick,E., Howard,D., Kovar,L., Seabolt,M., Morrison,S., Desch,M., Knipe,K., Weigand,M., Mcgrath,D., Takakuwa,J., Burgin,A., Burroughs,M., Lee,J., Wilkins,K., Mccollum,A., Hutson,C., Davidson,W., Rao,A., Dunn,J. and Li,Y.
EPI_ISL_18147435	Division of High-Consequence Pathogens & Pathology (DHCPP) - Poxvirus and Rabies Branch (PRB) - Centers for Disease Control and Prevention (CDC)	Division of High-Consequence Pathogens & Pathology (DHCPP) - Poxvirus and Rabies Branch (PRB) - Centers for Disease Control and Prevention (CDC)	Gigante,C., Ventura,J., Zhao,H., Batra,D., Hetrick,E., Howard,D., Kovar,L., Seabolt,M., Morrison,S., Desch,M., Knipe,K., Weigand,M., Mcgrath,D., Takakuwa,J., Burgin,A., Burroughs,M., Lee,J., Wilkins,K., Mccollum,A., Hutson,C., Davidson,W., Rao,A., Nash,J. and Li,Y.
EPI_ISL_18147436, EPI_ISL_18147437	Division of High-Consequence Pathogens & Pathology (DHCPP) - Poxvirus and Rabies Branch (PRB) - Centers for Disease Control and Prevention (CDC)	Division of High-Consequence Pathogens & Pathology (DHCPP) - Poxvirus and Rabies Branch (PRB) - Centers for Disease Control and Prevention (CDC)	Gigante,C., Ruiz,V., Zhao,H., Batra,D., Hetrick,E., Howard,D., Kovar,L., Seabolt,M., Morrison,S., Desch,M., Knipe,K., Weigand,M., Mcgrath,D., Takakuwa,J., Burgin,A., Burroughs,M., Lee,J., Wilkins,K., Mccollum,A., Hutson,C., Davidson,W., Rao,A., Wang,J. and Li,Y.
EPI_ISL_18147438	Division of High-Consequence Pathogens & Pathology (DHCPP) - Poxvirus and Rabies Branch (PRB) - Centers for Disease Control and Prevention (CDC)	Division of High-Consequence Pathogens & Pathology (DHCPP) - Poxvirus and Rabies Branch (PRB) - Centers for Disease Control and Prevention (CDC)	Gigante,C., Johnson,S., Zhao,H., Batra,D., Hetrick,E., Howard,D., Kovar,L., Seabolt,M., Morrison,S., Desch,M., Knipe,K., Weigand,M., Mcgrath,D., Takakuwa,J., Burgin,A., Burroughs,M., Lee,J., Wilkins,K., Mccollum,A., Hutson,C., Davidson,W., Rao,A., Riner,D. and Li,Y.
EPI_ISL_18147439	Division of High-Consequence Pathogens & Pathology (DHCPP) - Poxvirus and Rabies Branch (PRB) - Centers for Disease Control and Prevention (CDC)	Division of High-Consequence Pathogens & Pathology (DHCPP) - Poxvirus and Rabies Branch (PRB) - Centers for Disease Control and Prevention (CDC)	Gigante,C., Pettit,D., Zhao,H., Batra,D., Hetrick,E., Howard,D., Kovar,L., Seabolt,M., Morrison,S., Desch,M., Knipe,K., Weigand,M., Mcgrath,D., Takakuwa,J., Burgin,A., Burroughs,M., Lee,J., Wilkins,K., Mccollum,A., Hutson,C., Davidson,W., Rao,A., Deutsch-Feldman,M. and Li,Y.
EPI_ISL_18147440, EPI_ISL_18147441	Division of High-Consequence Pathogens & Pathology (DHCPP) - Poxvirus and Rabies Branch (PRB) - Centers for Disease Control and Prevention (CDC)	Division of High-Consequence Pathogens & Pathology (DHCPP) - Poxvirus and Rabies Branch (PRB) - Centers for Disease Control and Prevention (CDC)	Gigante,C., Ruiz,V., Zhao,H., Batra,D., Hetrick,E., Howard,D., Kovar,L., Seabolt,M., Morrison,S., Desch,M., Knipe,K., Weigand,M., Mcgrath,D., Takakuwa,J., Burgin,A., Burroughs,M., Lee,J., Wilkins,K., Mccollum,A., Hutson,C., Davidson,W., Rao,A., Wang,J. and Li,Y.
EPI_ISL_18147442	Division of High-Consequence Pathogens & Pathology (DHCPP) - Poxvirus and Rabies Branch (PRB) - Centers for Disease Control and Prevention (CDC)	Division of High-Consequence Pathogens & Pathology (DHCPP) - Poxvirus and Rabies Branch (PRB) - Centers for Disease Control and Prevention (CDC)	Gigante,C., Bradley,A., Zhao,H., Batra,D., Hetrick,E., Howard,D., Kovar,L., Seabolt,M., Morrison,S., Desch,M., Knipe,K., Weigand,M., Mcgrath,D., Takakuwa,J., Burgin,A., Burroughs,M., Lee,J., Wilkins,K., Mccollum,A., Hutson,C., Davidson,W., Rao,A., Anderson,J. and Li,Y.
EPI_ISL_18147443	Division of High-Consequence Pathogens & Pathology (DHCPP) - Poxvirus and Rabies Branch (PRB) - Centers for Disease Control and Prevention (CDC)	Division of High-Consequence Pathogens & Pathology (DHCPP) - Poxvirus and Rabies Branch (PRB) - Centers for Disease Control and Prevention (CDC)	Gigante,C., Ruiz,V., Zhao,H., Batra,D., Hetrick,E., Howard,D., Kovar,L., Seabolt,M., Morrison,S., Desch,M., Knipe,K., Weigand,M., Mcgrath,D., Takakuwa,J., Burgin,A., Burroughs,M., Lee,J., Wilkins,K., Mccollum,A., Hutson,C., Davidson,W., Rao,A., Wang,J. and Li,Y.
EPI_ISL_18147444	Division of High-Consequence Pathogens & Pathology (DHCPP) - Poxvirus and Rabies Branch (PRB) - Centers for Disease Control and Prevention (CDC)	Division of High-Consequence Pathogens & Pathology (DHCPP) - Poxvirus and Rabies Branch (PRB) - Centers for Disease Control and Prevention (CDC)	Gigante,C., Lee,B., Zhao,H., Batra,D., Hetrick,E., Howard,D., Kovar,L., Seabolt,M., Morrison,S., Desch,M., Knipe,K., Weigand,M., Mcgrath,D., Takakuwa,J., Burgin,A., Burroughs,M., Lee,J., Wilkins,K., Mccollum,A., Hutson,C., Davidson,W., Rao,A., Salehi,E. and Li,Y.
EPI_ISL_18147445	Division of High-Consequence Pathogens & Pathology (DHCPP) - Poxvirus and Rabies Branch (PRB) - Centers for Disease Control and Prevention (CDC)	Division of High-Consequence Pathogens & Pathology (DHCPP) - Poxvirus and Rabies Branch (PRB) - Centers for Disease Control and Prevention (CDC)	Gigante,C., Kubin,G., Zhao,H., Batra,D., Hetrick,E., Howard,D., Kovar,L., Seabolt,M., Morrison,S., Desch,M., Knipe,K., Weigand,M., Mcgrath,D., Takakuwa,J., Burgin,A., Burroughs,M., Lee,J., Wilkins,K., Mccollum,A., Hutson,C., Davidson,W., Rao,A., White,S. and Li,Y.
EPI_ISL_18147446	Division of High-Consequence Pathogens & Pathology (DHCPP) - Poxvirus and Rabies Branch (PRB) - Centers for Disease Control and Prevention (CDC)	Division of High-Consequence Pathogens & Pathology (DHCPP) - Poxvirus and Rabies Branch (PRB) - Centers for Disease Control and Prevention (CDC)	Gigante,C., Ruiz,V., Zhao,H., Batra,D., Hetrick,E., Howard,D., Kovar,L., Seabolt,M., Morrison,S., Desch,M., Knipe,K., Weigand,M., Mcgrath,D., Takakuwa,J., Burgin,A., Burroughs,M., Lee,J., Wilkins,K., Mccollum,A., Hutson,C., Davidson,W., Rao,A., Wang,J. and Li,Y.
EPI_ISL_18147447	Division of High-Consequence Pathogens & Pathology (DHCPP) - Poxvirus and Rabies Branch (PRB) - Centers for Disease Control and Prevention (CDC)	Division of High-Consequence Pathogens & Pathology (DHCPP) - Poxvirus and Rabies Branch (PRB) - Centers for Disease Control and Prevention (CDC)	Gigante,C., Xia,D., Zhao,H., Batra,D., Hetrick,E., Howard,D., Kovar,L., Seabolt,M., Morrison,S., Desch,M., Knipe,K., Weigand,M., Mcgrath,D., Takakuwa,J., Burgin,A., Burroughs,M., Lee,J., Wilkins,K., Mccollum,A., Hutson,C., Davidson,W., Rao,A., Plipat,N. and Li,Y.
EPI_ISL_18147448	Division of High-Consequence Pathogens & Pathology (DHCPP) - Poxvirus and Rabies Branch (PRB) - Centers for Disease Control and Prevention (CDC)	Division of High-Consequence Pathogens & Pathology (DHCPP) - Poxvirus and Rabies Branch (PRB) - Centers for Disease Control and Prevention (CDC)	Gigante,C., Goldoft,M., Zhao,H., Batra,D., Hetrick,E., Howard,D., Kovar,L., Seabolt,M., Morrison,S., Desch,M., Knipe,K., Weigand,M., Mcgrath,D., Takakuwa,J., Burgin,A., Burroughs,M., Lee,J., Wilkins,K., Mccollum,A., Hutson,C., Davidson,W., Rao,A., Holshue,M. and Li,Y.
EPI_ISL_18147449	Division of High-Consequence Pathogens & Pathology (DHCPP) - Poxvirus and Rabies Branch (PRB) - Centers for Disease Control and Prevention (CDC)	Division of High-Consequence Pathogens & Pathology (DHCPP) - Poxvirus and Rabies Branch (PRB) - Centers for Disease Control and Prevention (CDC)	Gigante,C., Kubin,G., Zhao,H., Batra,D., Hetrick,E., Howard,D., Kovar,L., Seabolt,M., Morrison,S., Desch,M., Knipe,K., Weigand,M., Mcgrath,D., Takakuwa,J., Burgin,A., Burroughs,M., Lee,J., Wilkins,K., Mccollum,A., Hutson,C., Davidson,W., Rao,A., White,S. and Li,Y.
EPI_ISL_18161269, EPI_ISL_18161270, EPI_ISL_18161271, EPI_ISL_18161272, EPI_ISL_18161273, EPI_ISL_18161274, EPI_ISL_18161275, EPI_ISL_18161276, EPI_ISL_18161277, EPI_ISL_18161279	Quest Diagnostics Nichols Institute	Los Angeles County Public Health Laboratories	J. Garrigues et al.
EPI_ISL_18161280	UCLA Clinical Micro Lab	Los Angeles County Public Health Laboratories	J. Garrigues et al.
EPI_ISL_18161281, EPI_ISL_18161282, EPI_ISL_18161284, EPI_ISL_18161285, EPI_ISL_18161286	Quest Diagnostics Nichols Institute	Los Angeles County Public Health Laboratories	J. Garrigues et al.
EPI_ISL_18161289, EPI_ISL_18161290, EPI_ISL_18161291	Los Angeles County Public Health Laboratories	Los Angeles County Public Health Laboratories	J. Garrigues et al.
EPI_ISL_18161292	Quest Diagnostics Nichols Institute	Los Angeles County Public Health Laboratories	J. Garrigues et al.
EPI_ISL_18161293, EPI_ISL_18161294, EPI_ISL_18161295, EPI_ISL_18161296	Laboratory Corporation of America	Los Angeles County Public Health Laboratories	J. Garrigues et al.
EPI_ISL_18161297, EPI_ISL_18161298, EPI_ISL_18161299, EPI_ISL_18161300, EPI_ISL_18161301, EPI_ISL_18161302, EPI_ISL_18161303, EPI_ISL_18161304, EPI_ISL_18161305	Quest Diagnostics Nichols Institute	Los Angeles County Public Health Laboratories	J. Garrigues et al.
EPI_ISL_18161306	Los Angeles County Public Health Laboratories	Los Angeles County Public Health Laboratories	J. Garrigues et al.
EPI_ISL_18161307, EPI_ISL_18161308, EPI_ISL_18161309, EPI_ISL_18161310, EPI_ISL_18161312, EPI_ISL_18161314	Quest Diagnostics Nichols Institute	Los Angeles County Public Health Laboratories	J. Garrigues et al.
EPI_ISL_18161315	ARUP Laboratories	Los Angeles County Public Health Laboratories	J. Garrigues et al.
EPI_ISL_18161316, EPI_ISL_18161317, EPI_ISL_18161318, EPI_ISL_18161319, EPI_ISL_18161320, EPI_ISL_18161321, EPI_ISL_18161322, EPI_ISL_18161324	Laboratory Corporation of America	Los Angeles County Public Health Laboratories	J. Garrigues et al.
EPI_ISL_18161325	Los Angeles County Public Health Laboratories	Los Angeles County Public Health Laboratories	J. Garrigues et al.
EPI_ISL_18161326	Quest Diagnostics Nichols Institute	Los Angeles County Public Health Laboratories	J. Garrigues et al.
EPI_ISL_18168621, EPI_ISL_18168622, EPI_ISL_18168623	Quest Diagnostics	RIPHL at Rush University Medical Center	Stefan Green, Kevin Kunstman, Hannah Barbian, Sofiya Bobrovska, Felix Araujo Perez, Edith Perez, Cecilia Chau, Giancarlo Balangue, Lok Yiu Ashley Wu, Trisha Jeon, Marisol Dominguez, Latifah Boyd
EPI_ISL_18168624	ACL Laboratories	RIPHL at Rush University Medical Center	Stefan Green, Kevin Kunstman, Hannah Barbian, Sofiya Bobrovska, Felix Araujo Perez, Edith Perez, Cecilia Chau, Giancarlo Balangue, Lok Yiu Ashley Wu, Trisha Jeon, Marisol Dominguez, Latifah Boyd
EPI_ISL_18213374, EPI_ISL_18213375	Institute for Hepatology,Shenzhen Third People's Hospital	Institute for Hepatology,Shenzhen Third People's Hospital	Lin Cheng,Zheng Zhang

EPI_ISL_18228619, EPI_ISL_18228621, EPI_ISL_18228622, EPI_ISL_18228624, EPI_ISL_18228625, EPI_ISL_18228626, EPI_ISL_18228629, EPI_ISL_18228630, EPI_ISL_18228631, EPI_ISL_18228632, EPI_ISL_18228633, EPI_ISL_18228634, EPI_ISL_18228637, EPI_ISL_18228638, EPI_ISL_18228640, EPI_ISL_18228641, EPI_ISL_18228643, EPI_ISL_18228645

see above	Center for Vectors and Infectious Diseases Research (CEVDI), National Health Institute Doutor Ricardo Jorge, IP (INSA)	Center for Vectors and Infectious Diseases Research (CEVDI), National Health Institute Doutor Ricardo Jorge, IP (INSA)	Isidro,J., Borges,V., Pinto,M., Sobral,D., Santos,J., Nunes,A., Mixao,V., Ferreira,R., Santos,D., Duarte,S., Vieira,L., Borrego,M.J., Nuncio,S., Lopes de Carvalho,I., Pelerito,A., Cordeiro,R. and Gomes,J.P.
EPI_ISL_18238278, EPI_ISL_18238279, EPI_ISL_18238280, EPI_ISL_18238281, EPI_ISL_18238282, EPI_ISL_18238283, EPI_ISL_18238284, EPI_ISL_18238285, EPI_ISL_18238286, EPI_ISL_18238287, EPI_ISL_18238291, EPI_ISL_18238292, EPI_ISL_18238293, EPI_ISL_18238294, EPI_ISL_18238295, EPI_ISL_18238296, EPI_ISL_18238297, EPI_ISL_18238298, EPI_ISL_18238299, EPI_ISL_18238300, EPI_ISL_18238301			
see above	Centre for Biological Threats, Highly Pathogenic Viruses, Robert Koch Institute	Centre for Biological Threats, Highly Pathogenic Viruses, Robert Koch Institute	Brinkmann,A., Kohl,C., Pape,K., Schrick,L., Michel,J., Schaade,L. and Nitsche,A.
EPI_ISL_18238302, EPI_ISL_18238303, EPI_ISL_18238304, EPI_ISL_18238305, EPI_ISL_18238306, EPI_ISL_18238307, EPI_ISL_18238308, EPI_ISL_18238309, EPI_ISL_18238310, EPI_ISL_18238312, EPI_ISL_18238313, EPI_ISL_18238314, EPI_ISL_18238315, EPI_ISL_18238316, EPI_ISL_18238317, EPI_ISL_18238319, EPI_ISL_18238320, EPI_ISL_18238321, EPI_ISL_18238322, EPI_ISL_18238323, EPI_ISL_18238324, EPI_ISL_18238325, EPI_ISL_18238326, EPI_ISL_18238327			
see above	DPH, Massachusetts State Public Health Laboratory	DPH, Massachusetts State Public Health Laboratory	Doucette,M., Gagne,L. and Smole,S.C.
EPI_ISL_18241786, EPI_ISL_18241787, EPI_ISL_18241788, EPI_ISL_18241789, EPI_ISL_18241790, EPI_ISL_18241791, EPI_ISL_18241792	Unidade de Genômica - UFRJ	Unidade de Genômica - UFRJ	Carolina Moreira Voloch, Filipe Romero Rebello Moreira, Diana Mariani, Rafael Mello Galliez, Debora Souza Faffe, Terezinha Marta Pereira Pinto Castiñeiras, Clarissa Damaso, Amílcar Tanuri.
EPI_ISL_18245407, EPI_ISL_18245408, EPI_ISL_18245409, EPI_ISL_18245410, EPI_ISL_18245411, EPI_ISL_18245412, EPI_ISL_18245413	Indian Council of Medical Research-National Institute of Virology	Indian Council of Medical Research-National Institute of Virology	Pragya Yadav, Rima Sahay, Anita Aich Shete, Sreelekshmy Mohandas
EPI_ISL_18257122	Haidian CDC	Haidian District Center for Disease Control and Prevention Microbiological Laboratory	Fangyao Liu, Lifei Shi,Feng Liu, Heng Zhang
EPI_ISL_18285959	UCLA Clinical Micro Lab	Los Angeles County Public Health Laboratories	J. Garrigues et al.
EPI_ISL_18285960, EPI_ISL_18285961, EPI_ISL_18285962, EPI_ISL_18285963, EPI_ISL_18285964, EPI_ISL_18285965, EPI_ISL_18285966, EPI_ISL_18285967, EPI_ISL_18285968	Quest Diagnostics Nichols Institute	Los Angeles County Public Health Laboratories	J. Garrigues et al.
EPI_ISL_18285969	Laboratory Corporation of America	Los Angeles County Public Health Laboratories	J. Garrigues et al.
EPI_ISL_18285970	Quest Diagnostics Nichols Institute	Los Angeles County Public Health Laboratories	J. Garrigues et al.
EPI_ISL_18285971, EPI_ISL_18285972	Cedars-Sinai Medical Center	Los Angeles County Public Health Laboratories	J. Garrigues et al.
EPI_ISL_18285973, EPI_ISL_18285974, EPI_ISL_18285975, EPI_ISL_18285976, EPI_ISL_18285977, EPI_ISL_18285978, EPI_ISL_18285979, EPI_ISL_18285980, EPI_ISL_18285981, EPI_ISL_18285982, EPI_ISL_18285983, EPI_ISL_18285984, EPI_ISL_18285985, EPI_ISL_18285986, EPI_ISL_18285987			
see above	Quest Diagnostics	RIPHL at Rush University Medical Center	Stefan Green, Kevin Kunstman, Hannah Barbian, Sofiya Bobrovska, Felix Araujo Perez, Edith Perez, Cecilia Chau, Giancarlo Balangue, Lok Yiu Ashley Wu, Trisha Jeon, Marisol Dominguez, Latifah Boyd
EPI_ISL_18299473, EPI_ISL_18299474	Centre for Biological Threats, Highly Pathogenic Viruses, Robert Koch Institute	Centre for Biological Threats, Highly Pathogenic Viruses, Robert Koch Institute	Brinkmann,A., Kohl,C., Schrick,L., Michel,J., Schaade,L. and Nitsche,A.
EPI_ISL_18308395, EPI_ISL_18308396, EPI_ISL_18308397, EPI_ISL_18308398, EPI_ISL_18308399	National Virus Reference Laboratory	National Virus Reference Laboratory	Gabriel Gonzalez, Michael Carr, Emer O'Byrne, Weronika Banka, Brian Keogan, Jonathan Dean, Daniel Hare, Cillian F De Gascun
EPI_ISL_18323779, EPI_ISL_18323780, EPI_ISL_18323781, EPI_ISL_18323782, EPI_ISL_18323783, EPI_ISL_18323784, EPI_ISL_18323785, EPI_ISL_18323786, EPI_ISL_18323787, EPI_ISL_18323788, EPI_ISL_18323789, EPI_ISL_18323790, EPI_ISL_18323791, EPI_ISL_18323792, EPI_ISL_18323793, EPI_ISL_18323794, EPI_ISL_18324980, EPI_ISL_18324981, EPI_ISL_18324982, EPI_ISL_18324983, EPI_ISL_18324984, EPI_ISL_18324985, EPI_ISL_18324986, EPI_ISL_18324987, EPI_ISL_18324988, EPI_ISL_18324989, EPI_ISL_18324990, EPI_ISL_18324991, EPI_ISL_18324992, EPI_ISL_18324993, EPI_ISL_18324994, EPI_ISL_18324995, EPI_ISL_18324996, EPI_ISL_18324997, EPI_ISL_18324998, EPI_ISL_18324999, EPI_ISL_18325000, EPI_ISL_18325001, EPI_ISL_18325002, EPI_ISL_18325003, EPI_ISL_18325004, EPI_ISL_18325005, EPI_ISL_18325006, EPI_ISL_18325007, EPI_ISL_18325008, EPI_ISL_18325010, EPI_ISL_18325011			
see above	California Department of Public Health	California Department of Public Health	Kath, C., Haw, M., Espinosa, A., and Hacker, J.
EPI_ISL_18352302, EPI_ISL_18352303, EPI_ISL_18352304, EPI_ISL_18352305, EPI_ISL_18352306	Tokyo Metropolitan Institute of Public Health	Tokyo Metropolitan Institute of Public Health	Fumi Kasuya, Wakaba Okada, Ryota Kumagai, Sachiko Harada, Arisa Amano, Michiya Hasegawa, Mami Nagashima, Kenji Sadamasu
EPI_ISL_18354483	Shenzhen Key Laboratory of Pathogen and Immunity	Shenzhen Key Laboratory of Pathogen and Immunity	Yang Yang, Shengjie Zhang, Yun Peng, Fuxiang Wang, Yingxia Liu, Hongzhou Lu
EPI_ISL_18360394	Haidian District Center for Disease Control and Prevention Microbiological Laboratory	Haidian District Center for Disease Control and Prevention Microbiological Laboratory	Fangyao Liu, Lifei Shi,Feng Liu, Heng Zhang
EPI_ISL_18361186	University of Washington, Department of Laboratory Medicine	University of Washington, Department of Laboratory Medicine	Sereewit,J., Xie,H., Roychoudhury,P. and Greninger,A.L.
EPI_ISL_18386999, EPI_ISL_18387000, EPI_ISL_18387001, EPI_ISL_18387002, EPI_ISL_18387003, EPI_ISL_18387004, EPI_ISL_18387005, EPI_ISL_18387006, EPI_ISL_18387008, EPI_ISL_18387009, EPI_ISL_18387010, EPI_ISL_18387011, EPI_ISL_18387012, EPI_ISL_18387013			
see above	NC - Los Angeles County Public Health Laboratories	NC - Los Angeles County Public Health Laboratories	Garrigues,J.M. and Green,N.M.
EPI_ISL_18387015	Institut Pasteur de Dakar, Virology Unit	Institut Pasteur de Dakar, Virology Unit	Martin,F., Anges,Y., Benjamin,H., Amadou,S.A. and Ousmane,F.
EPI_ISL_18399134, EPI_ISL_18399135, EPI_ISL_18399136, EPI_ISL_18399137, EPI_ISL_18399138, EPI_ISL_18399139, EPI_ISL_18399140, EPI_ISL_18399141, EPI_ISL_18399142, EPI_ISL_18399143, EPI_ISL_18399144, EPI_ISL_18399145, EPI_ISL_18399146, EPI_ISL_18399147			
see above	California Department of Public Health	California Department of Public Health	Kath, C., Haw, M., Espinosa, A., and Hacker, J.
EPI_ISL_18414668, EPI_ISL_18414669, EPI_ISL_18414671, EPI_ISL_18414672	Laboratory Medicine, UW Virology	Laboratory Medicine, UW Virology	Sereewit,J., Xie,H., Roychoudhury,P. and Greninger,A.L.
EPI_ISL_18427686, EPI_ISL_18427687, EPI_ISL_18427688, EPI_ISL_18427689, EPI_ISL_18427690, EPI_ISL_18427691	Quest Diagnostics	RIPHL at Rush University Medical Center	Stefan Green, Kevin Kunstman, Hannah Barbian, Sofiya Bobrovska, Felix Araujo Perez, Edith Perez, Cecilia Chau, Giancarlo Balangue, Lok Yiu Ashley Wu, Trisha Jeon, Marisol Dominguez, Latifah Boyd
EPI_ISL_18427692	ACL Laboratories	RIPHL at Rush University Medical Center	Stefan Green, Kevin Kunstman, Hannah Barbian, Sofiya Bobrovska, Felix Araujo Perez, Edith Perez, Cecilia Chau, Giancarlo Balangue, Lok Yiu Ashley Wu, Trisha Jeon, Marisol Dominguez, Latifah Boyd
EPI_ISL_18436040	PKC Mampang Prapatan	National Institute of Health Research and Development	Fajar Nur Sulistyahadi, Arie Ardiansyah Nugraha, Hana Apsari Pawestri, Kartika Dewi Puspa, Herna, Subangkit, IGM Wirabrata
EPI_ISL_18436041	PKC Jatinegara	National Institute of Health Research and Development	Fajar Nur Sulistyahadi, Arie Ardiansyah Nugraha, Hana Apsari Pawestri, Kartika Dewi Puspa, Herna, Subangkit, IGM Wirabrata
EPI_ISL_18443030, EPI_ISL_18443031, EPI_ISL_18443032, EPI_ISL_18443033, EPI_ISL_18443034, EPI_ISL_18443035, EPI_ISL_18443036, EPI_ISL_18443037, EPI_ISL_18443038, EPI_ISL_18443039, EPI_ISL_18443040, EPI_ISL_18443041, EPI_ISL_18443042, EPI_ISL_18443043, EPI_ISL_18443044, EPI_ISL_18443045, EPI_ISL_18452332, EPI_ISL_18452334, EPI_ISL_18452335, EPI_ISL_18452336, EPI_ISL_18452337, EPI_ISL_18452338, EPI_ISL_18452339, EPI_ISL_18452340, EPI_ISL_18452341, EPI_ISL_18452342, EPI_ISL_18452343, EPI_ISL_18452344, EPI_ISL_18452345, EPI_ISL_18452346, EPI_ISL_18452347			
see above	California Department of Public Health	California Department of Public Health	Kath, C., Haw, M., Espinosa, A., and Hacker, J.
